# National-level and state-level prevalence of overweight and obesity among children, adolescents, and adults in the USA, 1990–2021, and forecasts up to 2050

**DOI:** 10.1016/S0140-6736(24)01548-4

**Published:** 2024-12-07

**Authors:** GBD 2021 US Obesity Forecasting Collaborators, Marie Ng, Xiaochen Dai, Rebecca M Cogen, Michael Abdelmasseh, Arash Abdollahi, Auwal Abdullahi, Richard Gyan Aboagye, Hana J Abukhadijah, Temitayo Esther Adeyeoluwa, Aanuoluwapo Adeyimika Afolabi, Danish Ahmad, Noah Ahmad, Ayman Ahmed, Syed Anees Ahmed, Mohammed Ahmed Akkaif, Ashley E Akrami, Syed Mahfuz Al Hasan, Omar Al Ta'ani, Fares Alahdab, Ziyad Al-Aly, Wafa A Aldhaleei, Abdelazeem M Algammal, Waad Ali, Akram Al-Ibraheem, Saleh A Alqahatni, Rami H Al-Rifai, Najim Z Alshahrani, Mohammad Al-Wardat, Hany Aly, Walid A Al-Zyoud, Sohrab Amiri, Abhishek Anil, Jalal Arabloo, Aleksandr Y Aravkin, Ali Ardekani, Demelash Areda, Mubarek Yesse Ashemo, Alok Atreya, Sina Azadnajafabad, Shahkaar Aziz, Peter S Azzopardi, Giridhara Rathnaiah Babu, Atif Amin Baig, Abdulaziz T Bako, Kannu Bansal, Till Winfried Bärnighausen, Mohammad-Mahdi Bastan, Maryam Bemanalizadeh, Azizullah Beran, Habtamu B Beyene, Sonu Bhaskar, Cem Bilgin, Archie Bleyer, Hamed Borhany, Edward J Boyko, Dejana Braithwaite, Dana Bryazka, Raffaele Bugiardini, Yasser Bustanji, Zahid A Butt, Mehtap Çakmak Barsbay, Ismael Campos-Nonato, Francieli Cembranel, Ester Cerin, Pamela Roxana Chacón-Uscamaita, Eeshwar K Chandrasekar, Vijay Kumar Chattu, An-Tian Chen, Guangjin Chen, Gerald Chi, Patrick R Ching, So Mi Jemma Cho, Dong-Woo Choi, Bryan Chong, Sheng-Chia Chung, Zinhle Cindi, Karly I Cini, Alyssa Columbus, Rosa A S Couto, Michael H Criqui, Natalia Cruz-Martins, Omar B Da'ar, Omid Dadras, Zhaoli Dai, Samuel Demissie Darcho, Nihar Ranjan Dash, Hardik Dineshbhai Desai, Samath Dhamminda Dharmaratne, Daniel Diaz, Michael J Diaz, Thanh Chi Do, Mahsa Dolatshahi, Mario D'Oria, Ojas Prakashbhai Doshi, Rajkumar Prakashbhai Doshi, Robert Kokou Dowou, John Dube, Dorothea Dumuid, Arkadiusz Marian Dziedzic, Abdel Rahman E'mar, Rabie Adel El Arab, Ibrahim Farahat El Bayoumy, Muhammed Elhadi, Chadi Eltaha, Luca Falzone, Hossein Farrokhpour, Patrick Fazeli, Valery L Feigin, Ginenus Fekadu, Nuno Ferreira, Florian Fischer, Kate Louise Francis, Muktar A Gadanya, Miglas Welay Gebregergis, Delaram J Ghadimi, Ehsan Gholami, Mahaveer Golechha, Davide Golinelli, Philimon N Gona, Mahdi Gouravani, Ayman Grada, Ashna Grover, Avirup Guha, Rahul Gupta, Parham Habibzadeh, Nils Haep, Aram Halimi, Md. Kamrul Hasan, Md Saquib Hasnain, Simon I Hay, Wen-Qiang He, Jeffrey J Hebert, Mehdi Hemmati, Yuta Hiraike, Nguyen Quoc Hoan, Sorin Hostiuc, Chengxi Hu, Junjie Huang, Hong-Han Huynh, Md. Rabiul Islam, Sheikh Mohammed Shariful Islam, Louis Jacob, Abel Joseph, Sivesh Kathir Kamarajah, Kehinde Kazeem Kanmodi, Rami S Kantar, Yeganeh Karimi, Sina Kazemian, Mohammad Jobair Khan, Muhammad Shahzeb Khan, Praval Khanal, Shaghayegh Khanmohammadi, Khaled Khatab, Moawiah Mohammad Khatatbeh, Moein Khormali, Jagdish Khubchandani, Sylvia Kiconco, Min Seo Kim, Ruth W Kimokoti, Adnan Kisa, Mukhtar Kulimbet, Vijay Kumar, Satyajit Kundu, Om P Kurmi, Hanpeng Lai, Nhi Huu Hanh Le, Munjae Lee, Seung Won Lee, Wei-Chen Lee, An Li, Wei Li, Stephen S Lim, Jialing Lin, Paulina A Lindstedt, Xiaofeng Liu, Justin Lo, José Francisco López-Gil, Giancarlo Lucchetti, Lisha Luo, Jay B Lusk, Elham Mahmoudi, Elaheh Malakan Rad, Yosef Manla, Ramon Martinez-Piedra, Yasith Mathangasinghe, Fernanda Penido Matozinhos, Steven M McPhail, Hadush Negash Meles, George A Mensah, Sultan Ayoub Meo, Tomislav Mestrovic, Irmina Maria Michalek, GK Mini, Mohammad Mirza-Aghazadeh-Attari, Gabriele Mocciaro, Jama Mohamed, Mouhand F H Mohamed, Nouh Saad Mohamed, Ameen Mosa Mohammad, Shafiu Mohammed, Ali H Mokdad, Kaveh Momenzadeh, Sara Momtazmanesh, Fateme Montazeri, Maziar Moradi-Lakeh, Shane Douglas Morrison, Rohith Motappa, Erin C Mullany, Christopher J L Murray, Pirouz Naghavi, Soroush Najdaghi, Delaram Narimani Davani, Gustavo G Nascimento, Zuhair S Natto, Dang H Nguyen, Hau Thi Hien Nguyen, Phat Tuan Nguyen, Van Thanh Nguyen, Yeshambel T Nigatu, Nasrin Nikravangolsefid, Syed Toukir Ahmed Noor, Fred Nugen, Ogochukwu Janet Nzoputam, Bogdan Oancea, Erin M O'Connell, Sylvester Reuben Okeke, Andrew T Olagunju, Omotola O Olasupo, Abdulhakeem Abayomi Olorukooba, Samuel M Ostroff, Abderrahim Oulhaj, Mayowa O Owolabi, Mahesh Padukudru P A, Romil R Parikh, Seoyeon Park, Sungchul Park, Ava Pashaei, Gavin Pereira, Hoang Nhat Pham, Tom Pham, Anil K Philip, Jalandhar Pradhan, Pranil Man Singh Pradhan, Nicolaas P Pronk, Jagadeesh Puvvula, Seyedeh Niloufar Rafiei Alavi, Catalina Raggi, Muhammad Aziz Rahman, Bita Rahmani, Mohammad Rahmanian, Shakthi Kumaran Ramasamy, Chhabi Lal Ranabhat, Sowmya J Rao, Sina Rashedi, Ahmed Mustafa Rashid, Elrashdy Moustafa Mohamed Redwan, Taeho Gregory Rhee, Monica Rodrigues, Jefferson Antonio Buendia Rodriguez, Cameron John Sabet, Siamak Sabour, Umar Saeed, Dominic Sagoe, Mohamed A Saleh, Vijaya Paul Samuel, Abdallah M Samy, Aswini Saravanan, Monika Sawhney, Susan M M Sawyer, Nikolaos Scarmeas, Markus P Schlaich, Art Schuermans, Sadaf G Sepanlou, Allen Seylani, Mahan Shafie, Nilay S Shah, Muhammad Aaqib Shamim, Mohammad Ali Shamshirgaran, Sadaf Sharfaei, Amin Sharifan, Anupam Sharma, Manoj Sharma, Aziz Sheikh, Rekha Raghuveer Shenoy, Premalatha K Shetty, Kenji Shibuya, Aminu Shittu, Kerem Shuval, Emmanuel Edwar Siddig, Diego Augusto Santos Silva, Jasvinder A Singh, Amanda E Smith, Ranjan Solanki, Sameh S M Soliman, Yi Song, Soroush Soraneh, Kurt Straif, Lukasz Szarpak, Seyyed Mohammad Tabatabaei, Celine Tabche, Manoj Tanwar, Nathan Y Tat, Mohamad-Hani Temsah, Aravind Thavamani, Thang Huu Tran, Domenico Trico, Thien Tan Tri Tai Truyen, Stefanos Tyrovolas, Arit Udoh, Sana Ullah, Seyed Mohammad Vahabi, Sanaz Vahdati, Asokan Govindaraj Vaithinathan, Azin Vakilpour, Jef Van den Eynde, Manish Vinayak, Kosala Gayan Weerakoon, Nuwan Darshana Wickramasinghe, Asrat Arja Wolde, Tewodros Eshete Wonde, Suowen Xu, Lin Yang, Yuichiro Yano, Arzu Yiğit, Dong Keon Yon, Chuanhua Yu, Chun-Wei Yuan, Michael Zastrozhin, Mohammed G M Zeariya, Claire Chenwen Zhong, Bin Zhu, Abzal Zhumagaliuly, Magdalena Zielińska, Sa'ed H Zyoud, Jessica A Kerr, Stein Emil Vollset, Emmanuela Gakidou

## Abstract

**Background:**

Over the past several decades, the overweight and obesity epidemic in the USA has resulted in a significant health and economic burden. Understanding current trends and future trajectories at both national and state levels is crucial for assessing the success of existing interventions and informing future health policy changes. We estimated the prevalence of overweight and obesity from 1990 to 2021 with forecasts to 2050 for children and adolescents (aged 5–24 years) and adults (aged ≥25 years) at the national level. Additionally, we derived state-specific estimates and projections for older adolescents (aged 15–24 years) and adults for all 50 states and Washington, DC.

**Methods:**

In this analysis, self-reported and measured anthropometric data were extracted from 134 unique sources, which included all major national surveillance survey data. Adjustments were made to correct for self-reporting bias. For individuals older than 18 years, overweight was defined as having a BMI of 25 kg/m^2^ to less than 30 kg/m^2^ and obesity was defined as a BMI of 30 kg/m^2^ or higher, and for individuals younger than 18 years definitions were based on International Obesity Task Force criteria. Historical trends of overweight and obesity prevalence from 1990 to 2021 were estimated using spatiotemporal Gaussian process regression models. A generalised ensemble modelling approach was then used to derive projected estimates up to 2050, assuming continuation of past trends and patterns. All estimates were calculated by age and sex at the national level, with estimates for older adolescents (aged 15–24 years) and adults aged (≥25 years) also calculated for 50 states and Washington, DC. 95% uncertainty intervals (UIs) were derived from the 2·5th and 97·5th percentiles of the posterior distributions of the respective estimates.

**Findings:**

In 2021, an estimated 15·1 million (95% UI 13·5–16·8) children and young adolescents (aged 5–14 years), 21·4 million (20·2–22·6) older adolescents (aged 15–24 years), and 172 million (169–174) adults (aged ≥25 years) had overweight or obesity in the USA. Texas had the highest age-standardised prevalence of overweight or obesity for male adolescents (aged 15–24 years), at 52·4% (47·4–57·6), whereas Mississippi had the highest for female adolescents (aged 15–24 years), at 63·0% (57·0–68·5). Among adults, the prevalence of overweight or obesity was highest in North Dakota for males, estimated at 80·6% (78·5–82·6), and in Mississippi for females at 79·9% (77·8–81·8). The prevalence of obesity has outpaced the increase in overweight over time, especially among adolescents. Between 1990 and 2021, the percentage change in the age-standardised prevalence of obesity increased by 158·4% (123·9–197·4) among male adolescents and 185·9% (139·4–237·1) among female adolescents (15–24 years). For adults, the percentage change in prevalence of obesity was 123·6% (112·4–136·4) in males and 99·9% (88·8–111·1) in females. Forecast results suggest that if past trends and patterns continue, an additional 3·33 million children and young adolescents (aged 5–14 years), 3·41 million older adolescents (aged 15–24 years), and 41·4 million adults (aged ≥25 years) will have overweight or obesity by 2050. By 2050, the total number of children and adolescents with overweight and obesity will reach 43·1 million (37·2–47·4) and the total number of adults with overweight and obesity will reach 213 million (202–221). In 2050, in most states, a projected one in three adolescents (aged 15–24 years) and two in three adults (≥25 years) will have obesity. Although southern states, such as Oklahoma, Mississippi, Alabama, Arkansas, West Virginia, and Kentucky, are forecast to continue to have a high prevalence of obesity, the highest percentage changes from 2021 are projected in states such as Utah for adolescents and Colorado for adults.

**Interpretation:**

Existing policies have failed to address overweight and obesity. Without major reform, the forecasted trends will be devastating at the individual and population level, and the associated disease burden and economic costs will continue to escalate. Stronger governance is needed to support and implement a multifaceted whole-system approach to disrupt the structural drivers of overweight and obesity at both national and local levels. Although clinical innovations should be leveraged to treat and manage existing obesity equitably, population-level prevention remains central to any intervention strategies, particularly for children and adolescents.

**Funding:**

Bill & Melinda Gates Foundation.

**Research in context**

**Evidence before this study**

Propelled by complex and interacting social and structural drivers, obesity is at a crisis point throughout the USA. The disease burden associated with overweight and obesity is acute, and this burden has pervasive social and economic consequences. Two of the most important considerations for the US Government to plan an effective response to this crisis include accessing contemporary estimates of overweight and obesity across the life course and understanding the timing and speed with which future increases in prevalence will arise. To review the literature focused on past and future epidemiology of overweight and obesity in the USA, we searched Ovid MEDLINE and PubMed for articles published from database inception up to April 30, 2024, using the terms “obese” AND “prevalence” AND “forecasting” AND “United States” (and synonyms for each, including state names) with no language or year restrictions. We also searched the grey literature and the reference lists of relevant systematic reviews and meta-analyses. In addition to the US-specific estimates provided within previous global publications (eg, Global Burden of Disease studies and NCD Risk Factor Collaboration), there have been many national-level estimates of overweight and obesity for the period 1990–2021, with most disaggregated by sex and race. These studies show a consistent upward trajectory in mean BMI and obesity prevalence across all age groups. We found comparatively fewer forecasting studies (total 26), most of which forecast prevalence only to 2030. Most focused on national estimates for adults, with six studies reporting national-level forecasts among age groups younger than 20 years. Studies consistently suggested that, at a national level, one in two adults were estimated to be obese and four in five adults were estimated to be overweight or obese by 2030. Available national-level 2030 forecasts for children and adolescents are more heterogeneous, with overweight and obesity prevalence estimated to increase to 29–33% for children (aged 6–11 years) and 31–50% for adolescents (age 12–19 years). At the state level, forecasts among children or adolescents largely focused on individual US states or populations (eg, one from Georgia and another from Pennsylvania). No study contained national and state-level forecasts for all states, among all age groups.

**Added value of this study**

To our knowledge, our study is the first to report the historical and projected trends in overweight and obesity for older adolescents (aged 15–24 years) and adults (aged ≥25 years) from 1990 to 2021, with forecasts to 2050 for total number and prevalence at the national level and across all 50 states and Washington, DC. Additionally, we provide past, current, and forecasted national-level prevalence for children and younger adolescents (aged 5–14 years). In our analysis, we used all available national and subnational data in the USA and applied systematic adjustments to reconcile differences between self-reported and measured anthropometric data. We examined the differential surges of prevalences of overweight and obesity across age, sex, and state-level geography in the past three decades, and analysed how, if the current pattern holds, the future trajectory will affect the US population across the country.

**Implications of all the available evidence**

Our study highlights the need for greater investment in obesity prevention. The national and subnational analysis of historical trends highlights decades-long failure in tackling the epidemic. If the current pattern continues, more than 250 million people living in the USA will have overweight or obesity by 2050. Given that obesity is caused by numerous complex factors (eg, urbanisation, flawed food and agricultural systems, food insecurity, and wealth inequality), a whole-of-government, Health in All Policies approach is required to impose multisectoral structural changes. Such structural changes might include legislative amendments to promote access to healthy foods, social welfare interventions, and improved regulation of the food, agricultural, and marketing sectors. Moreover, new-generation clinical treatments, including anti-obesity medications, will probably become a key option for obesity management. However, current access to these treatments is inequitable and their efficacy varies widely among individuals. Although they have a place within personalised comprehensive management plans, clinical treatments alone will not solve the current and future obesity epidemic. The next administration must urgently focus on population-level prevention and intervention.

## Introduction

The USA has one of the largest populations with overweight and obesity in the world. The persistently high and continuously rising obesity trend has resulted in a profound slowing in health improvements.^1^ The US population does not experience the same level of health gain as their high-income counterparts in other countries. Over the past three decades, both life expectancy and healthy life expectancy in the USA have declined in global rankings.^2^ Obesity and overweight have contributed to substantial morbidity and mortality. In 2021 in the USA, 335 000 deaths and 11·6 million disability-adjusted life-years were attributed to overweight and obesity, making them one of the top and fastest-growing risk factors.^3^, ^4^ Obesity and overconsumption not only trigger substantial environmental change,^5^ but the economic costs are substantial,^6^ with the direct health-care costs attributable to obesity in the USA in 2016 estimated to be between US$261 billion and $481 billion.^7^, ^8^ Complications of obesity (eg, diabetes) have increased in prevalence by more than 140% in the past 30 years^9^ and have become one of the leading causes of health-care spending.^10^

The increase in overweight and obesity among children and adolescents is particularly concerning.^11^, ^12^, ^13^ Data from the National Health and Nutrition Examination Survey (NHANES) found that nearly 20% of children and adolescents in the USA aged 2–19 years lived with obesity.^14^ Obesity during childhood and adolescence directly affects mental health, social interactions, and physical functioning (eg, sports participation and sleep quality), and can trigger serious diseases before reaching young adulthood.^10^, ^15^, ^16^, ^17^ The effect of obesity among the younger population in the USA is becoming evident, with the prevalence of cardiovascular disease risk factors (eg, dyslipidaemia and hypertension) having increased over the past three decades,^15^ despite appearing steady in recent years.^16^, ^17^, ^18^ Moreover, the prevalence of type 2 diabetes has nearly doubled over the past two decades.^19^ Because obesity in childhood or adolescence is intergenerational and rarely resolves,^20^, ^21^, ^22^, ^23^ it is a key predictor of adult obesity.^24^ Appropriate monitoring of the prevalence of overweight and obesity at the population level is crucial for anticipating future disease burden and managing an effective prevention-focused response to rising levels of obesity.

Considerable geographical disparities in the prevalence of overweight and obesity in the USA have been documented.^25^, ^26^, ^27^, ^28^ Some of the disparities are structurally determined and driven by variations in area-based demographic characteristics, socioeconomics, and environmental factors.^29^, ^30^, ^31^, ^32^, ^33^ For instance, the concentration of so-called food deserts and food swamps,^34^ and the absence of safe open spaces in some areas, that are conducive to physical activity^35^ drive inequities in local obesity trends.^36^ Moreover, genetic predisposition exacerbates the susceptibility of some populations to environmental risk factors,^37^ causing significant racial and ethnic disparities in obesity.^38^ These systematic differences underscore the need for tailored policies to address these disparities across geographical areas.

To support urgent policy change and implementation, timely monitoring and forecasting of the prevalence of overweight and obesity are essential. Several studies have been published on historical trends of overweight and obesity at the national and state levels in the USA,^11^, ^39^, ^40^, ^41^ and a few have provided forecasts up to 2030.^42^, ^43^, ^44^

## Methods

### Overview

In this comprehensive study, we provide estimates of the prevalence of overweight and obesity for children and young adolescents (aged 5–14 years), older adolescents (aged 15–24 years),^45^ and adults (aged ≥25 years), by sex, from 1990 to 2021, with forecasts extending to 2050 for the absolute number of individuals with overweight and obesity among older adolescents and adults and for prevalence at both a national level and for all 50 states and Washington, DC. Additional forecasts for children and younger adolescents aged 5–14 years are provided at the national level. This manuscript was produced as part of the Global Burden of Diseases, Injuries, and Risk Factors Study (GBD) Collaborator Network and in accordance with the GBD Protocol and complies with GATHER (appendix 1 pp 34–35).

### Definition of overweight and obesity

Overweight and obesity are defined using BMI, calculated as bodyweight in kg divided by the square of height in m (kg/m^2^). For individuals aged 18 years and older, a BMI of 25 kg/m^2^ to less than 30 kg/m^2^ is defined as overweight, and a BMI of 30 kg/m^2^ or higher is defined as obese. The classifications for children and adolescents (younger than 18 years) were based on International Obesity Task Force (IOTF) criteria.^46^ The US Centers for Disease Control and Prevention (CDC) growth chart is a common alternative for assessing childhood obesity in the USA. In this study, we adopted the IOTF criteria in alignment with the approach from GBD 2021.^3^ Based on published validation studies, IOTF criteria seem to be more conservative than CDC criteria but the two remain generally consistent with each other.^47^, ^48^

### Data sources

For this analysis, national and state-representative data on overweight and obesity were identified through a systematic review and literature search. Full information on the search strategy, inclusion criteria, and data extraction methods has been published previously.^49^, ^50^ Self-reported and directly measured heights, weights, and BMI data were included in our analysis. Studies were excluded if the samples were limited to specific subpopulations that were likely to be unrepresentative of the general population. Additionally, studies reporting overweight and obesity on the basis of alternative measures, such as waist circumference and waist-to-hip ratio, were excluded because there is no reliable method for accurate conversion of measurement to equivalent BMI-based prevalence estimates, and studies using BMI were by far the most numerous in all time periods.

134 data sources covering the period from 1980 to 2021 were included. These sources included all major national US CDC surveys, such as the NHANES from 1991 to 2019, the Behavioral Risk Factor Surveillance System from 1984 to 2021, the Gallup Daily Survey from 2008 to 2016, the National Health Interview Survey from 1980 to 2015, the National Youth Risk Behavior Surveillance System from 1999 to 2015, and the Study of Women's Health Across the Nation from 1996 to 2006. Individual-level microdata were extracted from these surveys for all ages. Any data of a specific sex and age group with sample sizes smaller than ten individuals were excluded. After data extraction, we did rigorous quality checks to eliminate any duplications, inconsistencies, or implausible data entries. A list of data sources is in appendix 1 (pp 3–30) and is accessible via the Global Health Data Exchange.

### Data standardisation

BMI calculated from measured height and bodyweight was used as the reference for our analyses because it is generally unbiased. To ensure consistency with this standard, we made adjustments to self-reported data to correct for potential biases. Details of the bias correction method are in appendix 1 (pp 30–31). Briefly, using available US datasets with self-reported information and measured NHANES data, statistical models based on meta-regression—Bayesian, regularised, trimmed (MR-BRT)^51^ were developed to estimate bias correction coefficients specific to each sex (male and female), 5-year age group (from age 5 years to age ≥80 years), and decade (1990–2000, 2000–10, 2010–21). The bias correction coefficients were applied to self-reported prevalence data from individuals aged 15 years and older. Corrections were not made for children's prevalence data (aged 5–14 years), because these data were directly measured.

### Estimation of the prevalence of overweight and obesity from 1990 to 2021

Spatiotemporal Gaussian process regression (ST-GPR) was used to generate a complete time series for the prevalence of overweight and obesity and the proportion of individuals with obesity among the population with overweight and obesity by age, sex, and year at the national level and for each state and Washington, DC, following a similar approach used in previous studies.^49^, ^50^ Briefly, we used a linear regression model to estimate the mean function of ST-GPR on the basis of covariates including age-standardised educational attainment level, the proportion of the population living in urban areas, and the proportion of the population working in agriculture at the state level. These covariates help capture some of the association between socioeconomic development and overweight and obesity.^30^, ^52^, ^53^, ^54^ Detailed descriptions of the models are in appendix 1 (p 32). We then calculated the prevalence of obesity by multiplying the prevalence of overweight and obesity by the estimated proportion of individuals with obesity among the population with overweight and obesity. Throughout this process, we carefully propagated the uncertainty in the estimates by using draws for the calculation. The 95% uncertainty intervals (UIs) for the final estimates were derived from the 2·5th and 97·5th percentiles of 1000 draws from the posterior distribution of ST-GPR and from the calculation. Further details are in previous publications.^49^, ^50^

### Forecast of the prevalence of overweight and obesity from 2022 to 2050

Forecasts were produced for a reference scenario that assumes the continuation of past trends and associations. Using prevalence estimates from 1990 to 2021 as inputs, we used a generalised ensemble modelling approach to forecast the prevalence of overweight and obesity, as well as the proportion of individuals with obesity among the population with overweight and obesity from 2022 to 2050.^55^ This approach integrated 12 submodels to leverage their combined predictive strengths. Six of these submodels utilised annualised rate of change models with different recency weights, placing varying emphasis on recent year-over-year trends. The remaining six submodels used a two-stage MR-BRT spline model with different statistical models and fitting procedures, accounting for the Socio-demographic Index.^55^, ^56^, ^57^ The forecasted prevalence of obesity was then calculated by multiplying the forecasted prevalence of overweight and obesity by the forecasted proportion of individuals with obesity among the population with overweight and obesity, using the draws to derive 95% UIs. Further details are in appendix 1 (pp 32–33). In addition to presenting the forecasted trend of prevalence over time and geography, we combined the forecasted 5-year age group prevalence from 2022 to 2050 with the estimates from 1990 to 2022 to examine the age-cohort pattern. Specifically, we converted the age-period data to age-cohort data. For instance, the cohort aged 5–9 years in 1990 would reflect the 1981–85 cohort. Leveraging the forecast data, we presented the prevalence of overweight and obesity in this birth cohort until they reached the age of 65–69 years in 2050. Examining the age-cohort pattern enables us to detect changes in age patterns and shifts in the onset age by cohort.

Analyses were completed with R (version 4.4.0) and Python (version 3.10.6).

### Role of the funding source

The funders of this study had no role in study design, data collection, data analysis, data interpretation, or the writing of the report.

## Results

### Prevalence of overweight and obesity in 2021

In 2021, an estimated 15·1 million (95% UI 13·5–16·8) children and young adolescents aged 5–14 years, 21·4 million (20·2–22·6) adolescents aged 15–24 years, and 172 million (169–174) adults aged 25 years and older had overweight or obesity in the USA. The age-standardised prevalence among children (aged 5–14 years) was estimated to be 36·2% (31·1–41·6) in males and 37·2% (31·3–43·5) in females. The prevalence among adolescents (aged 15–24 years) was estimated to be 46·7% (43·3–50·2) in males and 50·8% (46·7–54·9) in females. More adolescent females than males had obesity, with an estimated prevalence of 28·8% (25·4–32·5) and 22·7% (20·3–25·1), respectively. The age-standardised overweight and obesity prevalence among individuals aged 25 years and older was estimated at 75·9% (74·6–77·2) in males and 72·6% (70·8–74·3) in females. Similar to the sex difference among adolescents, more adult females than males had obesity, with an estimated prevalence of 45·6% (43·7–47·5) in females and 41·5% (40·1–43·2) in males.

**Figure 1:**
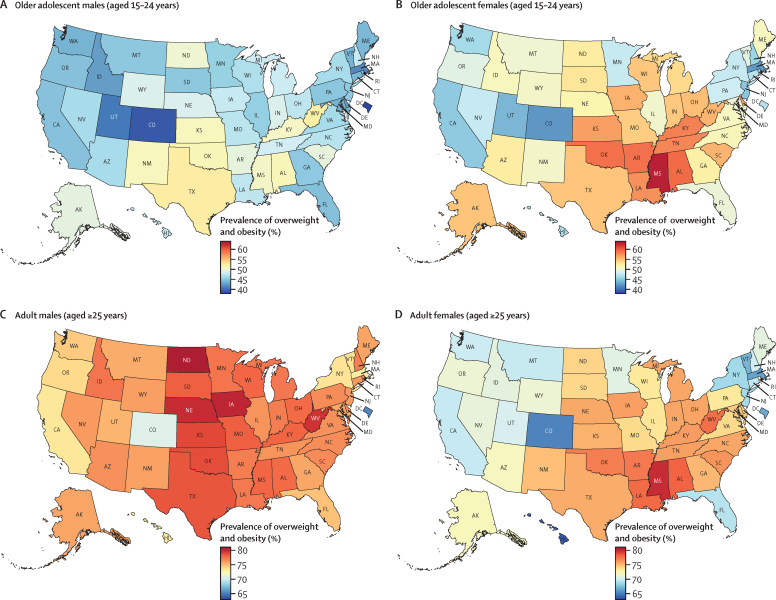
Estimated age-standardised prevalence of overweight and obesity in 50 US states and Washington, DC, in 2021, for adolescents and adults, by sex

In 2021, for all 50 states and Washington, DC, the prevalence of overweight and obesity was over 40% in both sexes combined (appendix 2 p 3) and was generally higher in female adolescents than in male adolescents (aged 15–24 years; figure 1A, B). Among adolescent females, the highest prevalence was observed in Mississippi at 63·0% (95% UI 57·0–68·5), followed by Alabama at 59·4% (53·5–65·3) and Oklahoma at 59·0% (52·9–65·0). Among males, the highest prevalence was observed in Texas at 52·4% (47·4–57·6), followed by West Virginia at 52·2% (46·9–57·9) and Oklahoma at 51·4% (45·6–56·9). Prevalence of obesity among female adolescents was above 20% for all states and Washington, DC, and above 30% for 20 states, with the highest prevalence observed in Mississippi (40·9% [35·1–46·5]; appendix 2 p 6). Among male adolescents, prevalence of obesity was above 20% in 46 states, with the highest prevalence observed in Oklahoma at 29·6% (25·0–34·5), followed by Mississippi and West Virginia, at 28·5% (24·0–33·8) and 27·7% (23·3–33·0), respectively (appendix 2 p 5).

In 2021, among adults, the estimated prevalence of overweight and obesity was high across all states (figure 1C, D). In males, prevalence ranged from 70·6% (95% UI 68·4–72·5) in Colorado to 80·6% (78·5–82·6) in North Dakota, and was lowest in Washington, DC, at 65·3% (62·7–68·0). Among females, prevalence ranged from 63·7% (61·2–66·4) in Hawaii to 79·9% (77·8–81·8) in Mississippi. Broader geographical variations were observed in terms of estimated prevalence of obesity (appendix 2 pp 7–8). Among males, the prevalence of obesity ranged from 30·4% (27·8–33·2) in Washington, DC, to 50·5% (47·9–53·1) in West Virginia. Prevalence was over 40% in 39 states and over 45% in 14 states (West Virginia, Kentucky, Iowa, North Dakota, Oklahoma, Arkansas, Alabama, Nebraska, Mississippi, Kansas, Ohio, Louisiana, Missouri, and Texas). Among females, the prevalence of obesity ranged from 36·0% (33·3–38·7) in Hawaii to 55·9% (53·2–58·5) in Mississippi. Prevalence was over 40% in 48 states and over 50% in 13 states (Mississippi, Louisiana, West Virginia, Alabama, Arkansas, South Carolina, Oklahoma, Indiana, Kentucky, Ohio, Iowa, Kansas, and Michigan).

### Prevalence of obesity by age

**Figure 2:**
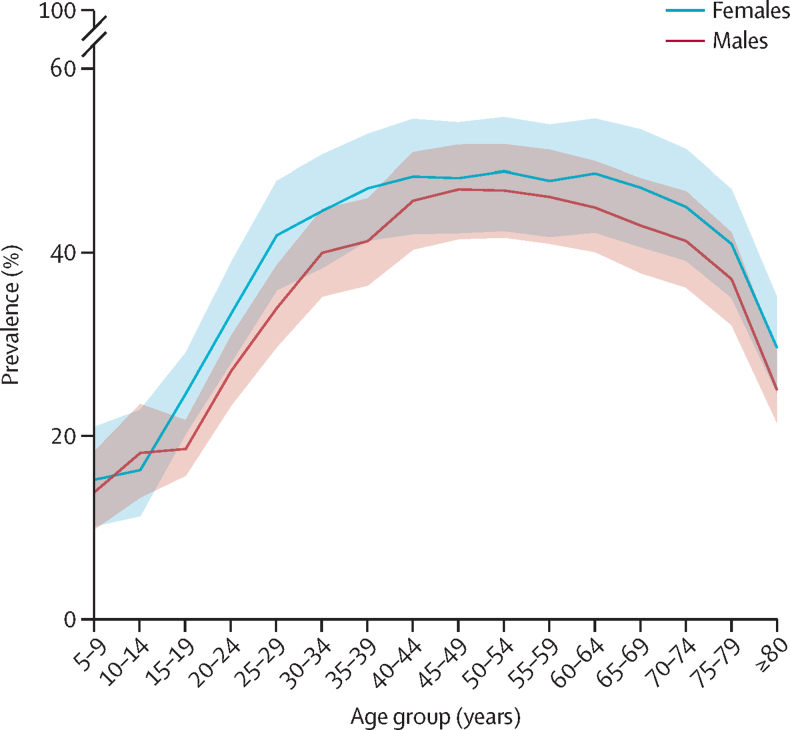
Sex-specific prevalence of obesity, by age group, in 2021 in the USA Shaded areas indicate 95% uncertainty intervals.

National levels of obesity across age groups in 2021 are shown by sex in figure 2. At ages 5–9 years, the estimated prevalence of obesity among males was 13·9% (9·9–18·3) and that among females was 15·2% (10·2–21·0). These sex differences widened during adolescence. Among females, substantial increases were observed in mid and late adolescence, with the prevalence increasing from 16·3% (11·3–22·8) in those aged 10–14 years to 24·6% (20·2–29·0) in those aged 15–19 years, and increasing again to 33·3% (28·0–38·9) in those aged 20–24 years. For males, estimated prevalence of obesity remained relatively stable between the ages of 10 years and 19 years but began to rise thereafter, increasing from 18·6% (15·7–21·7) among those aged 15–19 years to 27·1% (23·3–30·9) in those aged 20–24 years.

Among adults in 2021, by age 25 years, an estimated 41·8% (95% UI 35·9–47·7) of females had obesity compared with 33·9% (29·7–38·5) of males. The prevalence of obesity rose steadily with age, peaking at 48·7% (42·3–54·6) for females aged 50–54 years. For males, obesity prevalence peaked slightly earlier, at 46·8% (41·4–51·6) among those aged 45–49 years. The prevalence of obesity steadily declined from its peak, with the steepest drop observed around age 75 years for both sexes. Across states and Washington, DC, some variation was observed. The youngest peak among males was observed at age 40–44 years in seven states: Alabama, Indiana, Massachusetts, Montana, New Hampshire, North Dakota, and Wisconsin, where the estimated prevalence ranged from 43·8% (36·2–51·3) to 56·2% (47·8–64·4). The oldest peak was observed in North Carolina, with an estimated prevalence of 49·7% (42·3–57·4) at age 60–64 years. Among females, the youngest peak was observed at age 30–34 years in Rhode Island, with an estimated prevalence of 45·9% (36·7–55·6). Arizona and North Dakota observed the oldest peaks at ages 65–69 years, with estimated prevalence rates of 47·8% (38·5–57·1) and 50·9% (42·0–59·6), respectively (appendix 2 pp 70–93).

### Changes in the prevalence of overweight and obesity, 1990–2021

**Figure 3:**
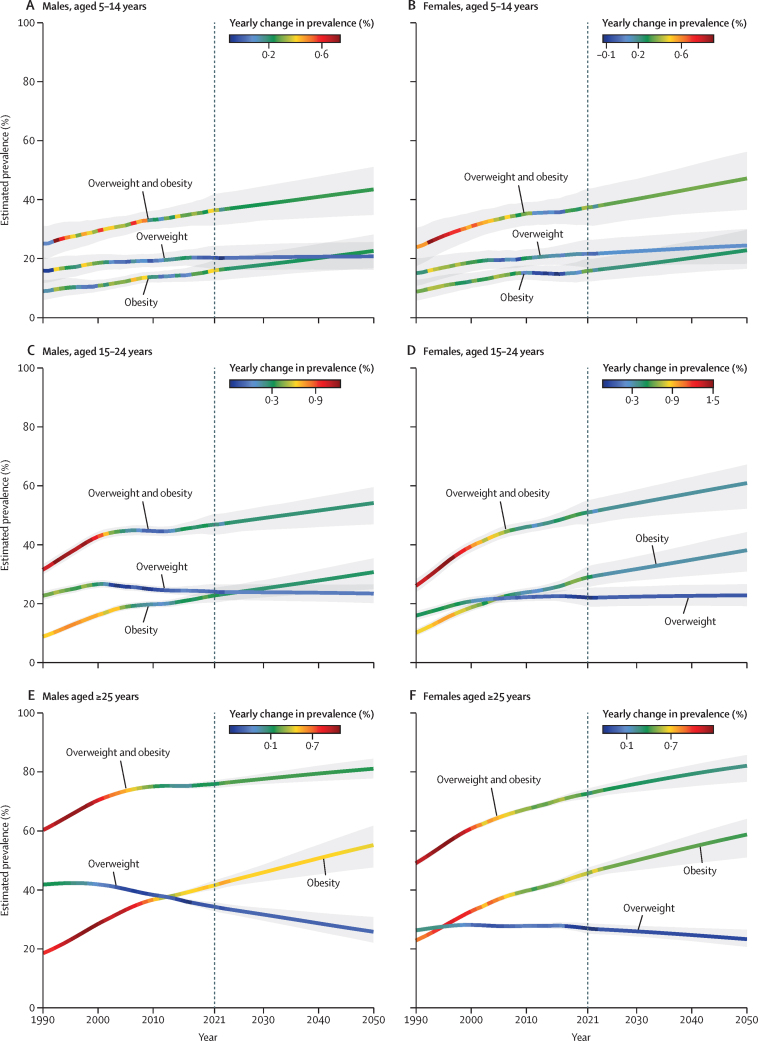
Estimated and projected prevalence of overweight and obesity combined, prevalence of overweight, and prevalence of obesity in children and young adolescents, older adolescents, and adults, by sex, from 1990 to 2050 in the USA Grey shaded areas show 95% uncertainty intervals, and the dotted vertical line indicates the point from which prevalence estimates start to be forecasts.

**Figure 4:**
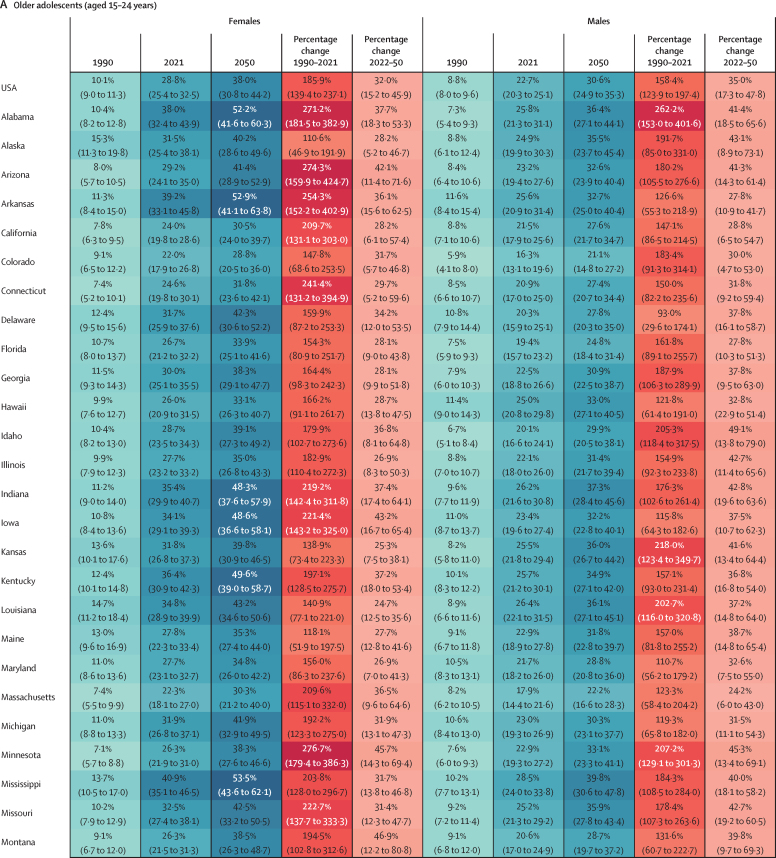

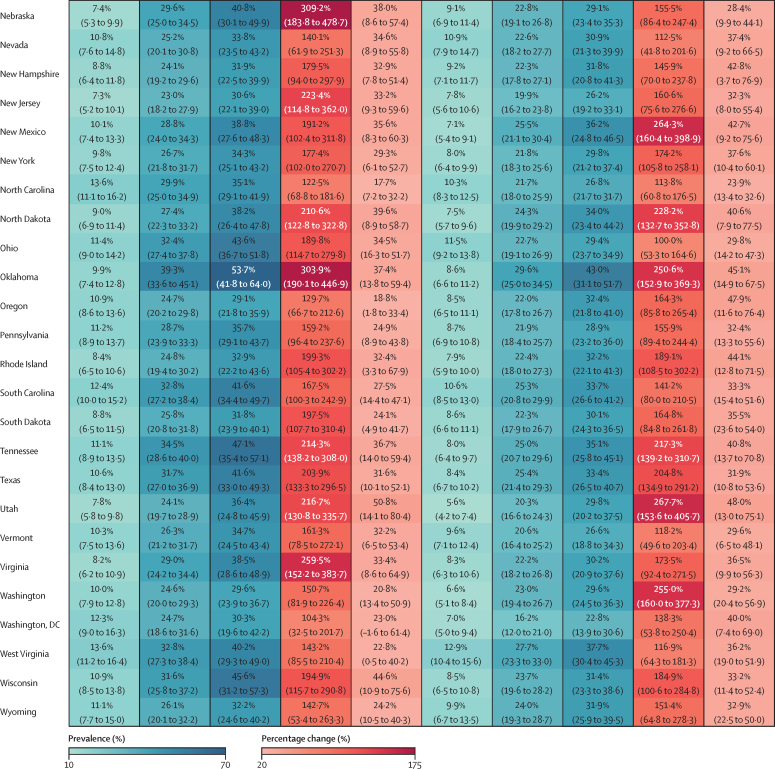

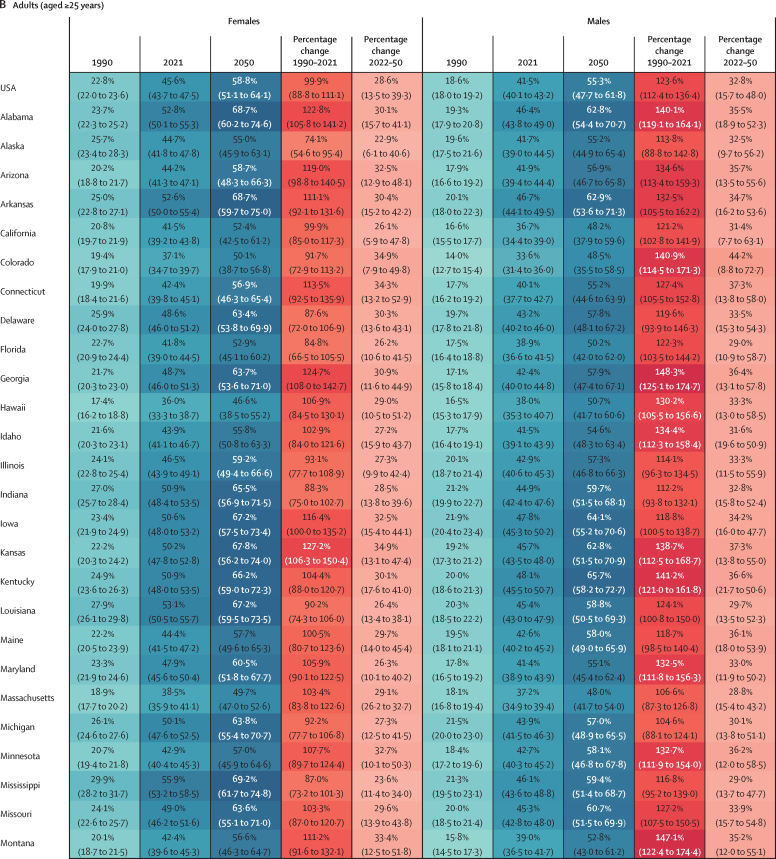

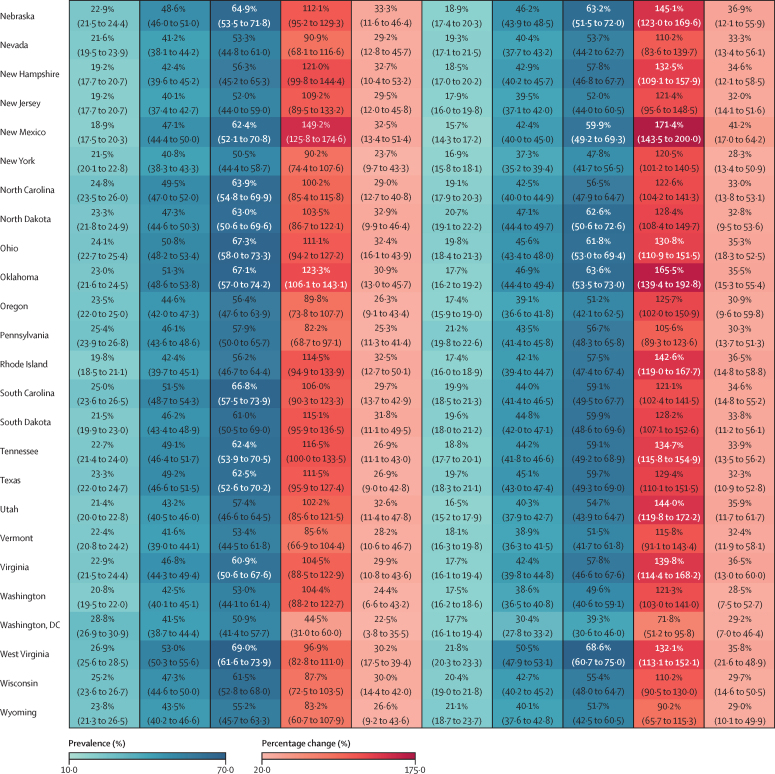
Estimated and projected age-standardised prevalence and percentage change in prevalence of obesity among older adolescents (aged 15–24 years; A) and adults (aged ≥25 years; B), by sex, for 1990, 2021, and 2050 in the USA at the national level and across 50 states and Washington, DC

At the national level, the estimated percentage change in prevalence of overweight and obesity combined increased linearly between 1990 and 2021 among children and adolescents aged 5–14 years (figure 3A, B). Between 1990 and 2021, the percentage change in the prevalence of overweight and obesity was 46·7% (95% UI 10·7–87·7) in males and 59·6% (14·0–117·5) in females aged 5–14 years, with the prevalence of obesity rising at a much more rapid pace than the prevalence of overweight alone. The increases in prevalence among adolescents aged 15–24 years were even greater (figure 3C, D; appendix 2 pp 9, 11, 13, 24). At the national level, between 1990 and 2021, the percentage change in the prevalence of overweight and obesity in adolescents aged 15–24 years was 48·6% (35·7–63·0) among males and 95·9% (74·5–119·6) among females (appendix 2 p 24), with much of the increase being driven by a sharp rise in obesity (mean change in prevalence of 158·4% [123·9–197·4] among males and 185·9% [139·4–237·1] among females). By state, the percentage change in prevalence of overweight and obesity was more than 50% among male adolescents in 20 states, with the most prominent increases observed in Utah, New Mexico, Texas, Alabama, and Arizona (appendix 2 pp 9, 24). The increase was much more substantial among female adolescents, with percentage changes of over 50% in all states, and Washington, DC, over 100% in 21 states, and over 145% in two states, Arizona and Alabama. The increase in the prevalence of obesity outpaced that of overweight in all states. For adolescent males, the percentage change in prevalence was more than 100% in 49 states and Washington, DC, with the highest increase observed in Utah (267·7% [153·6–405·7]), followed by New Mexico (264·3% [160·4–398·9]) and Alabama (262·2% [153·0–401·6]). For female adolescents, increases in the prevalence of obesity were even larger, with every state and Washington, DC recording an at least 100% increase, and the largest surge was in Nebraska (309·2% [183·8–478·7]), followed by Oklahoma (303·9% [190·1–446·9]) and Minnesota (276·7% [179·4–386·3]; figure 4A).

Among adults, at the national level, the estimated percentage change in prevalence of overweight and obesity was 25·6% (95% UI 22·7–28·6) for males and 47·9% (43·2–52·4) for females between 1990 and 2021 (figure 3E, F; appendix 2 p 25). By state, the largest percentage changes in the prevalence of overweight and obesity among males were in New Mexico (38·7% [32·8–44·9]), Arizona (38·5% [33·0–44·5]), and Hawaii (37·9% [31·3–45·7]). Among females, the largest percentage changes in the prevalence of overweight and obesity were in New Mexico (63·7% [55·1–73·0]), Rhode Island (60·4% [51·8–69·5]), and Arizona (59·9% [50·8–69·0]; appendix 2 p 25). Between 1990 and 2021, the prevalence of obesity increased at a much steeper rate, with a percentage change in prevalence of 123·6% (112·4–136·4) among males and 99·9% (88·8–111·1) among females (figure 4B; appendix 2 pp 15–20). For males, the percentage change in prevalence was more than 100% in 49 states, with the greatest increases observed in New Mexico (171·4% [143·5–200·0]), followed by Oklahoma (165·5% [139·4–192·8]) and Georgia (148·3% [125·1–174·7]). For females, the percentage change in prevalence was more than 100% in 32 states, with New Mexico having the greatest increase of 149·2% (125·8–174·6), followed by Kansas at 127·2% (106·3–150·4) and Georgia at 124·7% (108·0–142·7).

### Forecasts of overweight and obesity to 2050

Assuming a reference scenario where past trends and patterns persist, we forecast that, by 2050, an additional 3·33 million children and early adolescents (age 5–14 years) and 3·41 million adolescents (age 15–24 years) will have overweight or obesity. This will result in a total of 43·1 million (95% UI 37·2–47·4) children and adolescents (ages 5–24 years) with overweight or obesity, of whom 24·0 million (19·7–27·2) will be classified as having obesity (appendix 2 pp 21, 94). By 2050, the prevalence of overweight and obesity in children (aged 5–14 years) is projected to reach 45·1% (37·1–51·7), and the prevalence of obesity is projected to reach 22·6% (17·4–27·4). For adolescents (aged 15–24 years), the prevalence of overweight and obesity is projected to reach 57·3% (50·0–62·6), with obesity alone projected to reach 34·2% (28·5–38·7). Obesity among females aged 5–24 years is forecast to continue to dominate the trend, with a wider sex gap projected in older adolescents (aged 15–24 years). Among children (aged 5–14 years), percentage change in the prevalence of obesity is projected to be 41·2% (18·6–57·5) in males and 45·2% (20·1–67·8) in females compared with 2021 levels. By 2050, the prevalence of obesity in this age group is projected to be 22·5% (16·8–28·1) in males and 22·7% (16·5–29·6) in females. Among male adolescents (aged 15–24 years), the percentage change in the prevalence of obesity between 2021 and 2050 is projected to be 35·0% (17·3–47·8), rising to a prevalence of 30·6% (24·9–35·3) by 2050. In female adolescents (aged 15–24 years), the estimated percentage change in prevalence of obesity between 2022 and 2050 is 32·0% (15·2–45·9), reaching a projected prevalence of 38·0% (30·8–44·2) by 2050, resulting in a more than 7% difference in prevalence between males and females (figure 4A).

By 2050, prevalence of obesity among males aged 15–24 years is projected to be over 30% in 32 states, with forecasts as high as 43·0% (95% UI 31·1–51·7) for Oklahoma (figure 4A), with the percentage changes in the prevalence of obesity between 2021 and 2050 projected to be over 45% in Idaho, Utah, Oregon, Minnesota, and Oklahoma. Among females aged 15–24 years, 47 states and Washington, DC, are predicted to have a prevalence of obesity over 30%, with the highest rates expected in Oklahoma, Mississippi, Arkansas, and Alabama (all exceeding 50%; figure 4A). Because percentage changes in the prevalence of obesity in 1990–2021 were greater among adolescent females than among adolescent males, the forecasted rise in obesity among females is less rapid than among males, with the largest percentage change projected to be in Utah (50·8% [14·1–80·4]), followed by Montana (46·9% [12·2–80·8]; Figure 3, Figure 4). The largest numbers of adolescents with obesity will continue to be in California and Texas, with an estimated 1·53 million (1·25–1·89) and 1·49 million (1·20–1·75) adolescents being obese by 2050, respectively (appendix 2 p 22). However, New York is projected to surpass Florida to become the state with the third largest number of adolescents with obesity in the nation (appendix 2 p 94).

The number of adults with overweight and obesity is also forecast to increase substantially from 2021 to 2050. With an additional 41·4 million individuals, the total number of people with overweight and obesity aged 25 years and older is forecast to be 213 million (95% UI 202–221) in 2050, of whom 146 million (127–161) will have obesity (appendix 2 p 94). The projected prevalence of overweight and obesity in 2050 is estimated to be 81·1% (77·9–84·5) in adult males and 82·1% (76·7–85·7) in adult females (appendix 2 p 25). The prevalence of obesity is projected to increase at a more rapid rate than overweight, and faster among males than females (figure 3E, F). In males, the percentage change in the prevalence of obesity from 2021 to 2050 is estimated to be 32·8% (15·7–48·0), with a prevalence of 55·3% (47·7–61·8) in 2050 (figure 4B). For females, the percentage change in the prevalence of obesity from 2021 to 2050 is estimated to be 28·6% (13·5–39·3), with a prevalence of 58·8% (51·1–64·1) in 2050. Among males in 2050, 45 states are expected to have a prevalence of obesity of over 50%, with 11 states exceeding 60% (figure 4B). The highest prevalence of obesity is projected to be in West Virginia, estimated at 68·6% (60·7–75·0), followed by Kentucky at 65·7% (58·2–72·7) and Iowa at 64·1% (55·2–70·6). In terms of relative change, the largest increases are expected in Colorado and New Mexico, where the percentage change in the prevalence of obesity is predicted to be 44·2% (8·8–72·7) and 41·2% (17·0–64·2), respectively. Among females, except for Massachusetts and Hawaii, all 48 states and Washington, DC, are projected to have a prevalence of obesity of over 50%, with 12 states exceeding 65% (figure 4B). The highest prevalence is expected in Mississippi (69·2% [61·7–74·8]), followed by West Virginia (69·0% [61·6–73·9]), and Alabama (68·7% [60·2–74·6]). The largest percentage changes are expected in Kansas (34·9% [13·1–47·4]) and Colorado (34·9% [7·9–49·8]). The highest numbers of adults with obesity will continue to be in California (16·4 million [13·3–19·7]) and Texas (14·4 million [12·1–16·4]; appendix 2 pp 22).

### Cohort trajectories

**Figure 5:**
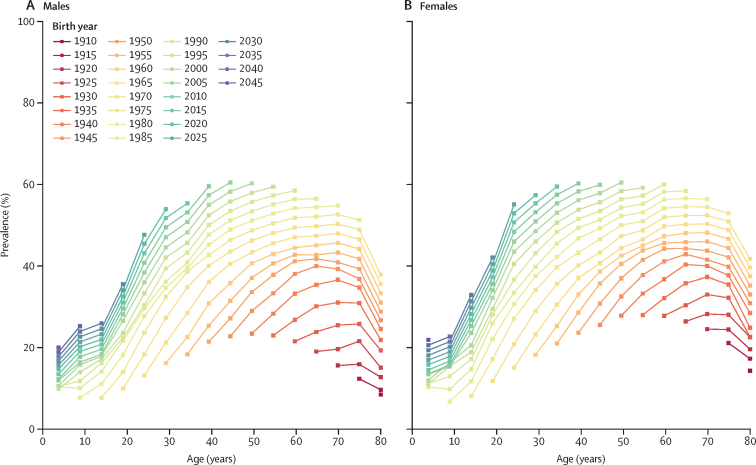
Prevalence of obesity by age across birth cohorts for males (A) and females (B)

Combining both historical estimates and forecast projections, trends in the prevalence of obesity across successive cohorts at the national level are presented in figure 5 and appendix 2 (p 23). For both males and females across birth cohorts the prevalence of obesity increased most rapidly between the ages of 20 years and 30 years, and it started to decline around the age of 70 years. At any given age, each successive birth cohort had a progressively higher prevalence of obesity. For example, at age 50 years, the 1945 birth cohort of females had a prevalence of 33·2% (95% UI 30·6–35·9). In each subsequent 5-year cohort, the prevalence increased by an average of approximately 6%. The 1970 birth cohort, which reached age 50 years in 2020, had a prevalence of obesity in 2020 of 48·2% (42·4–53·5). Forecast results suggest that by the time the 2000 birth cohort reaches age 50 years in 2050, the projected obesity prevalence will be 61·5% (51·0–69·4).

Comparing select cohorts across three different generations of females—those born in 1960, 1980, and 2020—showed that the onset of obesity has become earlier over subsequent generations. For the 1960 females' birth cohort, at the age of 45 years, the prevalence of obesity was estimated to be 39·3% (95% UI 36·5–42·0). In contrast, a similar level of prevalence was reached at age 30 years in the 1980 birth cohort and is projected to be reached at age 20 years in the 2020 birth cohort. A similar pattern was observed in males. For the 1960 males' birth cohort, at age 45 years the prevalence of obesity was estimated to be 35·9% (33·7–38·0). Conversely, the same level of prevalence was reached at age 30 years in the 1980 birth cohort and is projected to be surpassed at age 25 years in the 2020 birth cohort.

## Discussion

In this comprehensive analysis of the prevalence of overweight and obesity in the USA at national and state levels from 1990 to 2021, with projections to 2050, we found that nearly three-quarters of the adult population (aged ≥25 years) had overweight or obesity in 2021. The prevalence of obesity rose especially rapidly, doubling in the past three decades in both adult males and females. Our forecasts suggest that without immediate action, by 2050, the prevalence of overweight and obesity in adults will exceed 80% nationwide. The rate of increase in the prevalence of obesity among males will continue to overtake that among females and the high prevalence of obesity will continue to be concentrated in the southern states, such as Alabama, Arkansas, Kentucky, Louisiana, Mississippi, Oklahoma, South Carolina, and West Virginia. However, other states, including Colorado and New Mexico, that are forecast to have some of the largest relative increases, might soon catch up.

The prevalence of child and adolescent (aged 5–24 years) overweight and obesity has changed substantially in the past 30 years, with the highest percentage increase of 95·9% observed among female adolescents aged 15–24 years between 1990 and 2021. Despite some past indications that rates among children and adolescents had plateaued in most high-income countries,^58^, ^59^, ^60^, ^61^ including in the USA,^60^, ^61^ our forecasts show little evidence of stabilisation. By 2050, we project that an additional 6·73 million children and adolescents (aged 5–24 years) will have overweight or obesity compared with 2021. The increase in obesity is expected to outpace the increase in overweight, with more than one in five children and younger adolescents (aged 5–14 years) and at least one in three older adolescents (aged 15–24 years) experiencing obesity in 2050. Moreover, sex inequities are forecast to widen over time. Although a high prevalence of obesity is forecast for adolescent males (>35% prevalence in those aged 15–24 years) in Oklahoma, Mississippi, West Virginia, Indiana, Alabama, New Mexico, Louisiana, Kansas, Missouri, Alaska, and Tennessee, given the intergenerational risks of obesity,^21^ it is particularly concerning that more than 50% of adolescent females (aged 15–24 years) are forecast to have obesity in Mississippi, Arkansas, Oklahoma, and Alabama in 2050. Although these patterns in late adolescence are consistent with puberty-related sex differences in fat mass, the magnitude of the difference in prevalence signals that interventions need to be tailored by sex.^62^ Population-wide obesity in the USA is the result of excessive energy intake and limited physical activity, both of which have become socially normal.^63^ An alternative view purports that obesity is a complex disease that can be caused by various factors triggering energy imbalance.^64^, ^65^ As outlined in the 2019 *Lancet* Commission on the global syndemic of obesity, undernutrition, and climate change^5^ this energy imbalance reflects repeated exposure to intense and multiplying commercial, social, and structural drivers across interacting systems (eg, food and agriculture, urban design, land use, and transportation).^66^ In the past decade increasing attention has been paid to the commercial determinants of obesity, including the role that industry (eg, transnational food and beverage manufacturers) plays in manipulating the food environment, consumer behaviours (eg, via marketing and pricing), and fiscal policy via highly resourced lobbying forces.^5^, ^67^, ^68^ Indeed the resulting commercial profits associated with obesity are substantial for many industries (eg, the food industry and pharmaceutical industry).^68^, ^69^, ^70^ Aligning with the 2019 Commission report,^5^ the 2023 American Academy of Pediatrics (AAP) clinical practice guideline for the evaluation and treatment of children and adolescents with obesity^71^ now acknowledges that obesity is an embedded social issue, with complex drivers interacting across public policy, society, community, and built environments.^71^ In the past three decades, the USA has undergone extensive economic, demographic, and technological transitions that have triggered profound changes to agriculture practices, food security, food supply systems, commercialisation, urbanisation, densification, neighbourhood liveability, sedentariness, and wealth and educational inequalities, coupled with underlying structural racism and gender inequalities, that all interact to drive population-wide obesity.^36^, ^63^, ^72^, ^73^

Effective policy has not kept up with these substantial transitions in bodyweight and body composition,^74^ which are most concerning in the young. Because obesity in adolescence rarely resolves,^20^, ^28^, ^75^ this cohort will likely increasingly garner health comorbidities as they age, including diabetes, cardiovascular disease, kidney disease, cancer, infertility, mental health disorders, and premature death.^50^, ^76^, ^77^ Beyond individuals, health system and economic costs will be equally striking among a population where over half of children and adolescents aged 5–24 years are forecast to have overweight or obesity in 2050.^7^, ^8^, ^78^ Finally, the intergenerational effects of obesity indicate that urgent investments are needed to change these trajectories for the benefit of the current population of children and adolescents, the future adult population, and the next generation.^21^, ^22^

Given the chronicity of obesity once established,^20^ along with the substantial social and health system costs,^6^ prevention needs to become a much more dominant focus of obesity control. Preventing new cases of childhood obesity can be achieved by investing in preconception and perinatal interventions to circumvent intergenerational transmission.^22^, ^79^ The ideal prevention target age group would be adolescents entering their reproductive years (aged 15–24 years),^80^ of whom 21 million were estimated to have overweight or obesity in 2021—and without immediate action, this number will increase by another 3·41 million by 2050. To prevent the next generation from following a similar trajectory, actions are particularly urgent in states where close to 60% of female adolescents of childbearing age already have established overweight or obesity (eg, Mississippi and Alabama). Government policy priorities should be directed to funding-controlled intervention studies, including for maternal preconception obesity, given the scarcity of current evidence and investment.^22^

Most historical intervention efforts targeting children and adolescents have emphasised lifestyle-based behavioural interventions, such as current recommendations by the AAP and by the US Preventive Services Task Force.^60^, ^71^, ^81^ These individual-level programmes typically cannot address the complex drivers of obesity (eg, commercial determinants),^82^ require high contact time with specialised tertiary health professionals (≥26 h over 1 year) and only have moderate and unsustained success.^83^ Families whose children have obesity often do not have the insurance coverage required to access this level of care, and programmes are only available in select urban areas of the USA,^84^ notwithstanding efforts to improve coverage.^84^, ^85^ Maintaining healthy bodyweight during childhood and adolescence is also hindered by genetic predisposition for obesity,^86^ and by the incomplete maturation of executive functions, which heightens impulsivity in the face of commercial exploitations.^86^ Additionally, by adulthood, obesity becomes increasingly difficult to resolve because isolated attempts at lifestyle modification are likely to be overwhelmed by the body's physiological defence to store, rather than to lose, adipose tissue (eg, bodyweight set point).^87^, ^88^

Therefore, given the complexity of the drivers of overweight and obesity in children and adolescents, expecting behavioural interventions alone to produce sustainable and sufficient reductions in adiposity is quite unrealistic. Structural determinants need to be addressed with structural interventions (ie, legislative changes to address commercial determinants^67^) if we are to avoid the currently forecasted 2050 rates of overweight and obesity. According to the 2019 *Lancet* Commission on the global syndemic of obesity, undernutrition, and climate change,^5^ radical political and social movement is required to create sustainable and pro-ecological solutions. Rather than relying on individual agency,^89^ intervention efforts should be redirected, coordinated, intersectoral, policy-based, equity-focused, and population-wide.^58^, ^90^, ^91^ The WHO Global Action Plan for the Prevention and Control of Noncommunicable Diseases 2013–2020 recommends whole-of-government approaches to policy^92^ with the World Obesity Federation encouraging political leaders to safeguard regulations and policy making from commercial industry interference and lobbying.^93^ In the USA, some federal and state-level efforts have been made in the past, such as the Childhood Obesity Task Forces established in 2010 by state legislature,^94^ sugar-sweetened beverage taxes, school physical activity policy,^95^, ^96^ and the 2022 White House Conference on Hunger, Nutrition, and Health and associated 2030 targets.^97^ Investments from federal agencies, such as the US Department of Agriculture, the CDC, and the National Collaborative on Childhood Obesity Research, have been speculated to have curbed what otherwise could have been epidemic growth in obesity.^44^ Yet, despite these efforts, the prevalence of obesity has not substantially reduced.^74^ Investment should now extend beyond government to industry,^5^ and federal policy makers must look beyond short-term political goals to organise actions beyond the health sector to include education, social welfare, food and agricultural systems, and marketing sectors at national and community levels, and within domestic households.^5^, ^66^, ^98^

Examples of promising multifaceted, whole system approaches are emerging, with the most successful obesity interventions targeting multiple drivers across different sectors.^98^, ^99^, ^100^, ^101^ Recent evaluations of obesity policy in the USA^95^ and other high-income countries^102^, ^103^ should be harnessed, noting that other high-income countries have more comprehensive national-level multisectoral obesity policies.^104^ Urgent action is needed due to the lag between policy implementation and effect.^74^ Interventions and policies need to be age-appropriate,^71^, ^98^ including interventions adapted for pregnant women and early feeding practices.^80^ While school-aged children and young adolescents (aged 5–14 years) attend health services with their parents, who often direct their health and lifestyle-based decisions, from mid-adolescence, policies and interventions should harness the power of peer approval, autonomy, and social norms.^98^ Interventions with population-level sustainable and multisectoral benefits that could occur across various government sectors focused on education (eg, primary school meals and physical education), health (eg, opportunistic obesity screening), food systems and consumer environments (eg, subsidies and beverage taxes^95^), household resources (eg, cash transfers), and regulation (eg, policy to encourage safe and active transport and fast food legislation within and around schools) have been summarised elsewhere.^98^, ^105^, ^106^ Above all else, government levers need to be used to promote physical activity (eg, safe and walkable neighbourhoods), guarantee the availability of healthy foods to children and adolescents at school and at home, and regulate the food and marketing industries, and to reform food systems to be healthy, environmentally sustainable, and equitable.^5^

In addition to these much-needed public health interventions, the extremely high forecasts for overweight and obesity indicate many US adults, and some children and adolescents, will require more urgent treatment. For adults and post-pubertal adolescents, options might include anti-obesity medications, intensive dietary modification (eg, very low-energy diets), or bariatric surgeries.^58^, ^71^, ^106^, ^107^, ^108^ Many new-generation anti-obesity medications have shown clinically significant treatment efficacy.^109^, ^110^ The clinical trial on semaglutide in adolescents with obesity, for instance, has found an average of 16·1% reduction in BMI among treated participants after 68 weeks.^111^ However, these treatments require medical supervision and are not recommended for those who do not have obesity, for pre-pubertal children, or for adolescents younger than 12 years.^71^ As with other chronic diseases (eg, depression and diabetes), any medical treatment needs to be part of a comprehensive management plan that includes lifestyle interventions that have been shown to achieve greater results.^58^, ^112^ However, we must emphasise that the effectiveness of anti-obesity treatments varies widely across individuals, and some treatments are associated with serious side-effects.^113^, ^114^ Many unknowns remain in terms of the long-term effects of new-generation clinical interventions.^108^, ^112^, ^115^ Equitable access is an additional crucial consideration. The current pricing of the latest GLP-1 anti-obesity medications in the USA is prohibitive. A 2024 report from the US Senate Health, Education, Labor, and Pensions Committee estimated that if half of US adults with obesity took semaglutide, it could cost the health-care system $411 billion per year.^69^ This estimate is in sharp contrast with implementing population-wide smart food policies, such as taxation on sugar-sweetened beverages, which is estimated to cost between $430 million over 10 years and $1·7 billion over a lifetime.^116^, ^117^ Although the price of anti-obesity medications might decrease with the end of market exclusivity for some treatments,^118^ uncertainty remains regarding the extent of price negotiations, the availability of generic options, and the entry of new and more effective medications.^78^, ^119^, ^120^ The past adoption trends of treatments for other metabolic diseases, such as statins for dyslipidaemia, suggest that although prescription volumes increased after introducing cheaper generic options,^121^ considerable underutilisation remains in susceptible populations.^122^ Without addressing access disparities, the use of such treatments will simply widen current inequalities in obesity and associated disease burden, further exacerbating the life expectancy gaps across the USA.^123^ Therefore, anti-obesity medications and clinical treatments for obesity should not be viewed as a cure for the obesity epidemic. Prevention and treatment are both indispensable in providing holistic care for people at risk of and living with obesity. Along with enhancing treatment coverage, addressing the structural drivers of population obesity and emphasising prevention remain a central part of any comprehensive strategy.

The findings of this study should be interpreted while taking into account its limitations. First, the definition of overweight and obesity is based on BMI, which might not account for variations in body structure across the population.^124^ Despite its limitations, BMI has been found to correlate with other alternatives, such as waist circumference, waist-stature ratios, and dual x-ray absorptiometry, and is predictive of metabolic risks;^125^, ^126^, ^127^ hence, it continues to be a practical tool for population-level surveillance.^128^ Second, due to considerations of data quality and availability, we did not report the prevalence of overweight and obesity for individuals aged 5–14 years at the US state level. State-level measured child anthropometrics data are scarce. Although surveys such as the National Survey of Children's Health are representative of the state they are conducted in and provide data on children's height and bodyweight, the data are based on parents' or guardians' reports, which were found to be biased and unsuitable for assessing overweight prevalence.^129^ Alternatively, data from the Nutrition Program for Women, Infants, and Children and well-child visit records available from electronic medical records have sampling bias and do not have state representativeness. This limitation highlights the existing gap in monitoring child and adolescent obesity status and underscores the necessity to enhance screening methods. Third, we did not examine disparities across race and ethnicity. Given that policy decisions and implementation blueprints are typically formulated at the state level, we chose to focus on describing overweight and obesity status by state. However, we recognise the presence of racial and ethnic disparities^130^ and acknowledge the need to consider population characteristics in intervention planning. Fourth, our recommendations are US-specific and might only generalise to other high-income countries, rather than low-income or middle-income countries. Fifth, our analysis focused on overweight and obesity and did not differentiate between moderate and severe obesity categories. Sixth, to maximise data volume, self-reported data were included alongside biometric measures, but self-reported data are subject to systematic biases that differ by sex and change over time. Although we used an updated bias correction model, some imperfections might remain. Finally, our current forecasts only considered a reference scenario that assumed the continuation of trends based on historical data ending in 2021. Therefore, our projections do not capture the effect of the recent surge in the use of GLP-1 anti-obesity medications.^131^ With updated evidence on the long-term effects of new-generation anti-obesity medications and additional insights into the changing market access landscape, future forecast studies should explore how pharmacotherapies will shift obesity trends.

In conclusion, based on the current trajectory, overweight and obesity will continue to increase in the USA up to 2050. Existing policies have not shown adequate effectiveness. Future strategies must involve a multifaceted, whole-system approach, taking into consideration the complex drivers of obesity. Stronger governance is necessary to enact meaningful policy changes. Prevention remains the primary focus given the chronicity of the condition, with emphasis on children and adolescents. Meanwhile, regulations need to be put in place to eliminate barriers to accessing new-generation obesity clinical treatments, ensuring the availability and affordability of these options to the broader population. To protect population health, avoid overwhelming the health-care system, and mitigate mounting health-care costs, deliberate concerted action is needed to disrupt the epidemic of overweight and obesity.

### GBD 2021 US Obesity Forecasting Collaborators

### Affiliations

### Contributors

### Data sharing

To download GBD data used and estimates generated in these analyses, please visit the Global Health Data Exchange GBD 2021 website. Codes used for prevalence estimation and forecast are available through Github

## Declaration of interests
