# Supplementary material for: National-level and state-level prevalence of overweight and obesity among children, adolescents, and adults in the USA, 1990–2021, and forecasts up to 2050
Source: Lancet. 2024 Dec 7;404(10469):2278–98. doi: 10.1016/S0140-6736(24)01548-4 (PMC11694015; doi:10.1016/S0140-6736(24)01548-4)
Supplement: Supplementary appendix 1 [file mmc1.pdf]

THE LANCET

Supplementary appendix 1

This appendix formed part of the original submission and has been peer reviewed. We post it as supplied by the authors.

Supplement to: GBD 2021 US Obesity Forecasting Collaborators. National-level and state-level prevalence of overweight and obesity among children, adolescents, and adults in the USA, 1990–2021, and forecasts up to 2050. *Lancet* 2024; published online Nov 14. https://doi.org/10.1016/S0140-6736(24)01548-4.

Supplementary appendix 1

Methods appendix to:

National and state-level prevalence of overweight and obesity among children, adolescents, and adults in the USA, 1990–2021, and forecasts up to 2050

Table of Contents

Supplementary appendix 1 1
Data Sources Used for Overweight and Obesity Prevalence Estimation 3
Definition of overweight and obesity 30
Data Extraction 30
Data Standardisation and Adjustment 30
Self-report bias adjustment 30
Prevalence estimation for overweight and obesity 32
Forecast Modelling 32
GATHER Checklist 34
References 36

Data Sources Used for Overweight and Obesity Prevalence Estimation

A total of 134 data sources, including all major health surveillance databases, were included for the period from 1980 to 2021. Among these sources, 31 provided measured anthropometric information for adults, and 103 provided self-reported data. For children, 15 sources were included, all of which are measured data. Self-reported or caregiver-reported data were excluded for children due to quality considerations. SM Table 1 lists the data sources included in this study.

SM Table 1 Data Sources included in the estimation of overweight and obesity.

NID	Citation	Country	Representative	Sample Size	Years	Data type
507696	Centers for Disease Control and Prevention (CDC). United States Behavioral Risk Factor Surveillance System 2021. Atlanta, United States of America: Centers for Disease Control and Prevention (CDC), 2022.	USA	State + National	767915	2021-2021	self-report
507657	Centers for Disease Control and Prevention (CDC). United States Behavioral Risk Factor Surveillance System 2020. Atlanta, United States of America: Centers for Disease Control and Prevention (CDC), 2021.	USA	State + National	706076	2020-2020	self-report
465101	Centers for Disease Control and Prevention (CDC). United States Behavioral Risk Factor Surveillance System 2019. Atlanta, United States of America: Centers for Disease Control and Prevention (CDC), 2020.	USA	State + National	750582	2019-2019	self-report
498429	National Center for Health Statistics, Centers for Disease Control and Prevention. United States National Health and Nutrition Examination Survey Pre-Pandemic 2017-2020. Hyattsville, United States of America: National Center for Health Statistics, Centers for	USA	National	13116	2019-2019	measured

	Disease Control and Prevention, 2022.					
424067	Centers for Disease Control and Prevention (CDC). United States Behavioral Risk Factor Surveillance System 2018. Atlanta, United States of America: Centers for Disease Control and Prevention (CDC), 2019.	USA	State + National	791264	2018-2018	self-report
373675	Centers for Disease Control and Prevention (CDC). United States Behavioral Risk Factor Surveillance System 2017. Atlanta, United States of America: Centers for Disease Control and Prevention (CDC), 2018.	USA	State + National	815671	2017-2017	self-report
337617	Gallup. United States - Gallup Daily 2016. Washington, D.C., United States of America: Gallup.	USA	State + National	332883	2016-2016	self-report
411299	National Center for Health Statistics, Centers for Disease Control and Prevention. United States National Ambulatory Medical Care Survey 2016. Hyattsville, United States of America: National Center for Health Statistics, Centers for Disease Control and Prevention.	USA	National	7574	2016-2016	measured
205347	Centers for Disease Control and Prevention (CDC). United States National Youth Risk Behavior Survey 2015. Atlanta, United States of America: Centers for Disease Control and Prevention (CDC), 2016.	USA	National	10834	2015-2015	self-report
237986	National Center for Health Statistics, Centers for Disease Control and Prevention. United States National Health and Nutrition Examination Survey 2015-2016. Hyattsville, United States of America: National Center for Health Statistics,	USA	National	8753	2015-2015	measured

	Centers for Disease Control and Prevention, 2017.					
238008	National Center for Health Statistics, Centers for Disease Control and Prevention. United States National Health Interview Survey 2015. Hyattsville, United States of America: National Center for Health Statistics, Centers for Disease Control and Prevention, 2016.	USA	National	30421	2015-2015	self-report
264957	Centers for Disease Control and Prevention (CDC). United States Behavioral Risk Factor Surveillance System 2015. Atlanta, United States of America: Centers for Disease Control and Prevention (CDC), 2016.	USA	State + National	800962	2015-2015	self-report
337616	Gallup. United States - Gallup Daily 2015. Washington, D.C., United States of America: Gallup.	USA	State + National	332366	2015-2015	self-report
218067	National Center for Health Statistics, Centers for Disease Control and Prevention. United States National Health Interview Survey 2014. Hyattsville, United States of America: National Center for Health Statistics, Centers for Disease Control and Prevention, 2015.	USA	National	35352	2014-2014	self-report
225638	Centers for Disease Control and Prevention (CDC). United States Behavioral Risk Factor Surveillance System 2014. Atlanta, United States of America: Centers for Disease Control and Prevention (CDC), 2015.	USA	State + National	855455	2014-2014	self-report
337615	Gallup. United States - Gallup Daily 2014. Washington, D.C., United States of America: Gallup.	USA	State + National	329881	2014-2014	self-report

151813	National Center for Health Statistics, Centers for Disease Control and Prevention, United States Census Bureau (USCB). United States National Health Interview Survey 2013. Hyattsville, United States of America: National Center for Health Statistics, Centers for Disease Control and Prevention, 2014.	USA	National	31303	2013-2013	self-report
152390	Centers for Disease Control and Prevention (CDC). United States Behavioral Risk Factor Surveillance System 2013. Atlanta, United States of America: Centers for Disease Control and Prevention (CDC), 2014.	USA	State + National	918232	2013-2013	self-report
163917	Centers for Disease Control and Prevention (CDC). United States National Youth Risk Behavior Survey 2013. Atlanta, United States of America: Centers for Disease Control and Prevention (CDC), 2014.	USA	National	11307	2013-2013	self-report
165892	National Center for Health Statistics, Centers for Disease Control and Prevention. United States National Health and Nutrition Examination Survey 2013-2014. Hyattsville, United States of America: National Center for Health Statistics, Centers for Disease Control and Prevention.	USA	National	9045	2013-2013	measured
337614	Gallup. United States - Gallup Daily 2013. Washington, D.C., United States of America: Gallup.	USA	State + National	334067	2013-2013	self-report
104825	Centers for Disease Control and Prevention (CDC). United States Behavioral Risk Factor Surveillance System 2012. Atlanta, Georgia: CDC, US Department of Health and Human Services, 2013.	USA	State + National	883850	2012-2012	self-report

130846	Health and Retirement Study, (Biennial 2012) public use dataset. Produced and distributed by the University of Michigan with funding from the National Institute on Aging (grant number NIA U01AG009740). Ann Arbor, MI, (2020).	USA	National	14701	2012-2012	measured
142803	ISSP Research Group (2009): International Social Survey Programme: Health and Health Care - ISSP 2011. GESIS Data Archive, Cologne. ZA5800 Data file version 3.0.0, doi:10.4232/1.12252.	USA	National	1514	2012-2012	self-report
337605	Gallup. United States - Gallup Daily 2012. Washington, D.C., United States of America: Gallup.	USA	State + National	668811	2012-2012	self-report
83633	Centers for Disease Control and Prevention (CDC). United States Behavioral Risk Factor Surveillance System 2011. Atlanta, Georgia: CDC, US Department of Health and Human Services.	USA	State + National	939934	2011-2011	self-report
91569	National Center for Health Statistics (NCHS), Centers for Disease Control and Prevention (CDC), US Census Bureau. United States National Health Interview Survey 2011. Hyattsville, United States: National Center for Health Statistics (NCHS), Centers for Disease Control and Prevention (CDC).	USA	National	30144	2011-2011	self-report
110300	National Center for Health Statistics, Centers for Disease Control and Prevention. United States National Health and Nutrition Examination Survey 2011-2012. Hyattsville, United States of America: National Center for Health Statistics,	USA	National	8591	2011-2011	measured

	Centers for Disease Control and Prevention, 2013.					
163916	Centers for Disease Control and Prevention (CDC). United States National Youth Risk Behavior Survey 2011. Atlanta, United States of America: Centers for Disease Control and Prevention (CDC), 2012.	USA	National	12824	2011-2011	self-report
337604	Gallup. United States - Gallup Daily 2011. Washington, D.C., United States of America: Gallup.	USA	State + National	664198	2011-2011	self-report
42609	National Center for Health Statistics (NCHS), Centers for Disease Control and Prevention (CDC), US Census Bureau. United States National Health Interview Survey 2010. Hyattsville, United States: National Center for Health Statistics (NCHS), Centers for Disease Control and Prevention (CDC).	USA	National	24605	2010-2010	self-report
83627	Centers for Disease Control and Prevention (CDC). United States Behavioral Risk Factor Surveillance System 2010. Atlanta, United States of America: Centers for Disease Control and Prevention (CDC).	USA	State + National	842851	2010-2010	self-report
114636	National Center for Health Statistics, Centers for Disease Control and Prevention, SRA International, Inc., United States Census Bureau (USCB). United States National Hospital Ambulatory Medical Care Survey 2010. Hyattsville, United States of America: National Center for Health Statistics, Centers for Disease Control and Prevention.	USA	National	15615	2010-2010	measured
337603	Gallup. United States - Gallup Daily 2010. Washington, D.C., United States of America: Gallup.	USA	State + National	660613	2010-2010	self-report

30018	Centers for Disease Control and Prevention (CDC). United States Behavioral Risk Factor Surveillance System 2009. Atlanta, Georgia: CDC, US Department of Health and Human Services.	USA	State + National	807048	2009-2009	self-report
42525	National Center for Health Statistics (NCHS), Centers for Disease Control and Prevention (CDC), US Census Bureau. United States National Health Interview Survey 2009. Hyattsville, United States: National Center for Health Statistics (NCHS), Centers for Disease Control and Prevention (CDC).	USA	National	25163	2009-2009	self-report
48332	National Center for Health Statistics (NCHS), Centers for Disease Control and Prevention (CDC). United States National Health and Nutrition Examination Survey 2009-2010. Hyattsville, United States: National Center for Health Statistics (NCHS), Centers for Disease Control and Prevention (CDC), 2011.	USA	National	9404	2009-2009	measured
163915	Centers for Disease Control and Prevention (CDC). United States National Youth Risk Behavior Survey 2009. Atlanta, United States of America: Centers for Disease Control and Prevention (CDC), 2010.	USA	National	13685	2009-2009	self-report
191581	World Health Organization Regional Office for Europe (EURO-WHO). Health Behaviour in School-aged Children: WHO Collaborative Cross-National survey/study (HBSC) 2009-2010.	USA	National	1763	2009-2009	self-report
337602	Gallup. United States - Gallup Daily 2009. Washington, D.C., United States of America: Gallup.	USA	State + National	669233	2009-2009	self-report

30008	Centers for Disease Control and Prevention (CDC). United States Behavioral Risk Factor Surveillance System 2008. Atlanta, Georgia: CDC, US Department of Health and Human Services.	USA	State + National	773032	2008-2008	self-report
42517	National Center for Health Statistics (NCHS), Centers for Disease Control and Prevention (CDC), US Census Bureau. United States National Health Interview Survey 2008. Hyattsville, United States: National Center for Health Statistics (NCHS), Centers for Disease Control and Prevention (CDC).	USA	National	19617	2008-2008	self-report
120195	Harris, Kathleen Mullan, and J. Richard Udry. National Longitudinal Study of Adolescent to Adult Health (Add Health), 1994-2008. ICPSR21600-v12. Ann Arbor, MI: Inter-university Consortium for Political and Social Research [distributor], 2013-03-08. doi:10.3886/ICPSR21600.v12	USA	National	5032	2008-2008	measured
337601	Gallup. United States - Gallup Daily 2008. Washington, D.C., United States of America: Gallup.	USA	State + National	671650	2008-2008	self-report
25914	National Center for Health Statistics (NCHS), Centers for Disease Control and Prevention (CDC). United States National Health and Nutrition Examination Survey 2007-2008. Hyattsville, United States: National Center for Health Statistics (NCHS), Centers for Disease Control and Prevention (CDC), 2009.	USA	National	8854	2007-2007	measured
30000	Centers for Disease Control and Prevention (CDC). United States Behavioral Risk Factor	USA	State + National	804172	2007-2007	self-report

	Surveillance System 2007. Atlanta, Georgia: CDC, US Department of Health and Human Services.					
42427	National Center for Health Statistics (NCHS), Centers for Disease Control and Prevention (CDC), US Census Bureau. United States National Health Interview Survey 2007. Hyattsville, United States: National Center for Health Statistics (NCHS), Centers for Disease Control and Prevention (CDC).	USA	National	20847	2007-2007	self-report
86807	Panel Study of Income Dynamics, 2007 public use dataset. Produced and distributed by the University of Michigan with primary funding from the National Science Foundation, the National Institute of Aging, and the National Institute of Child Health and Human Development. Ann Arbor, MI, (2011).	USA	National	8134	2007-2007	self-report
154230	Sutton-Tyrrell, Kim, Faith Selzer, MaryFran Sowers, Joel Finkelstein, Lynda Powell, Ellen Gold, Gail David, Gerson Weiss, and Karen Matthews. Study of Women Across the Nation (SWAN), 2006-2008: Visit 10 Dataset. ICPSR32961-v1. Ann Arbor, MI: Inter-university Consortium for Political and Social Research [distributor], 2014-10-08. http://doi.org/10.3886/ICPSR32961.v1	USA	National	1964	2007-2007	measured
163914	Centers for Disease Control and Prevention (CDC). United States National Youth Risk Behavior Survey 2007. Atlanta, United States of America: Centers for Disease Control and Prevention (CDC), 2008.	USA	National	11803	2007-2007	self-report

29993	Centers for Disease Control and Prevention (CDC). United States Behavioral Risk Factor Surveillance System 2006. Atlanta, Georgia: CDC, US Department of Health and Human Services.	USA	State + National	658640	2006-2006	self-report
42357	National Center for Health Statistics (NCHS), Centers for Disease Control and Prevention (CDC), US Census Bureau. United States National Health Interview Survey 2006. Hyattsville, United States: National Center for Health Statistics (NCHS), Centers for Disease Control and Prevention (CDC).	USA	National	21714	2006-2006	self-report
153640	Sutton-Tyrell, Kim, Faith Selzer, MaryFran Sowers, Joel Finkelstein, Lynda Powell, Ellen Gold, Gail Greendale, Gerson Weiss, and Karen Matthews. Study of Women's Health Across the Nation (SWAN), 2005-2007: Visit 09 Dataset. ICPSR32721-v1. Ann Arbor, MI: Inter-university Consortium for Political and Social Research [distributor], 2014-09-30. http://doi.org/10.3886/ICPSR32721.v1	USA	National	1933	2006-2006	measured
29983	Centers for Disease Control and Prevention (CDC). United States Behavioral Risk Factor Surveillance System 2005. Atlanta, Georgia: CDC, US Department of Health and Human Services.	USA	State + National	662676	2005-2005	self-report
41635	National Center for Health Statistics (NCHS), Centers for Disease Control and Prevention (CDC), US Census Bureau. United States National Health Interview Survey 2005. Hyattsville, United States: National Center for Health Statistics (NCHS), Centers for	USA	National	28178	2005-2005	self-report

	Disease Control and Prevention (CDC).					
47478	National Center for Health Statistics (NCHS), Centers for Disease Control and Prevention (CDC). United States National Health and Nutrition Examination Survey 2005-2006. Hyattsville, United States: National Center for Health Statistics (NCHS), Centers for Disease Control and Prevention (CDC), 2007.	USA	National	8936	2005-2005	measured
86818	Panel Study of Income Dynamics, 2005 public use dataset. Produced and distributed by the University of Michigan with primary funding from the National Science Foundation, the National Institute of Aging, and the National Institute of Child Health and Human Development. Ann Arbor, MI, (2011).	USA	National	7835	2005-2005	self-report
153632	Sutton-Tyrell, Kim, Faith Selzer, MaryFran Sowers, Joel Finkelstein, Lynda Powell, Ellen Gold, Gail Greendale, Gerson Weiss, and Karen Matthews. Study of Women's Health Across the Nation (SWAN), 2004-2006: Visit 08 Dataset. ICPSR32122-v1. Ann Arbor, MI: Inter-university Consortium for Political and Social Research [distributor], 2014-09-30. http://doi.org/10.3886/ICPSR32122.v1	USA	National	1681	2005-2005	measured
163913	Centers for Disease Control and Prevention (CDC). United States National Youth Risk Behavior Survey 2005. Atlanta, United States of America: Centers for Disease Control and Prevention (CDC), 2006.	USA	National	12017	2005-2005	self-report

191508	World Health Organization Regional Office for Europe (EURO-WHO). Health Behaviour in School-aged Children: WHO Collaborative Cross-National survey/study (HBSC) 2005-2006.	USA	National	1236	2005-2005	self-report
29978	Centers for Disease Control and Prevention (CDC). United States Behavioral Risk Factor Surveillance System 2004. Atlanta, Georgia: CDC, US Department of Health and Human Services.	USA	State + National	563951	2004-2004	self-report
124091	National Institute on Alcohol Abuse and Alcoholism (NIAAA), National Institutes of Health (NIH), U.S. Department of Health and Human Services. United States National Epidemiologic Survey on Alcohol and Related Conditions 2004-2005.	USA	National	34316	2004-2004	self-report
153631	Sutton-Tyrell, Kim, Faith Selzer, MaryFran Sowers, Joel Finkelstein, Lynda Powell, Ellen Gold, Gail Greendale, Gerson Weiss, and Karen Matthews. Study of Women's Health Across the Nation (SWAN), 2003-2005: Visit 07 Dataset. ICPSR31901-v1. Ann Arbor, MI: Inter-university Consortium for Political and Social Research [distributor], 2014-09-30. http://doi.org/10.3886/ICPSR31901.v1	USA	National	2060	2004-2004	measured
29973	Centers for Disease Control and Prevention (CDC). United States Behavioral Risk Factor Surveillance System 2003. Atlanta, Georgia: CDC, US Department of Health and Human Services.	USA	State + National	489182	2003-2003	self-report
41522	National Center for Health Statistics (NCHS), Centers for Disease Control and Prevention	USA	National	27645	2003-2003	self-report

	(CDC), US Census Bureau. United States National Health Interview Survey 2003. Hyattsville, United States: National Center for Health Statistics (NCHS), Centers for Disease Control and Prevention (CDC).					
47962	National Center for Health Statistics (NCHS), Centers for Disease Control and Prevention (CDC). United States National Health and Nutrition Examination Survey 2003-2004. Hyattsville, United States: National Center for Health Statistics (NCHS), Centers for Disease Control and Prevention (CDC).	USA	National	8676	2003-2003	measured
86830	Panel Study of Income Dynamics, 2003 public use dataset. Produced and distributed by the University of Michigan with primary funding from the National Science Foundation, the National Institute of Aging, and the National Institute of Child Health and Human Development. Ann Arbor, MI, (2011).	USA	National	7378	2003-2003	self-report
153627	Sutton-Tyrell, Kim, Faith Selzer, MaryFran Sowers, Joel Finkelstein, Lynda Powell, Ellen Gold, Gail Greendale, Gerson Weiss, and Karen Matthews. Study of Women's Health Across the Nation (SWAN), 2002-2004: Visit 06 Dataset. ICPSR31181-v1. Ann Arbor, MI: Inter-university Consortium for Political and Social Research [distributor], 2014-09-24. http://doi.org/10.3886/ICPSR31181.v1	USA	National	2139	2003-2003	measured
163912	Centers for Disease Control and Prevention (CDC). United States National Youth Risk	USA	National	12604	2003-2003	self-report

	Behavior Survey 2003. Atlanta, United States of America: Centers for Disease Control and Prevention (CDC), 2004.					
13328	Alegria, Margarita, James S. Jackson, Ronald C. Kessler, and David Takeuchi. United States National Comorbidity Survey: Replication 2001-2003 [United States]. ICPSR20240-v7. Ann Arbor, MI: Inter-university Consortium for Political and Social Research [distributor], 2015-05-28. http://doi.org/10.3886/ICPSR20240.v7	USA	National	5086	2002-2002	measured
29968	Centers for Disease Control and Prevention (CDC). United States Behavioral Risk Factor Surveillance System 2002. Atlanta, Georgia: CDC, US Department of Health and Human Services.	USA	State + National	457939	2002-2002	self-report
41257	National Center for Health Statistics (NCHS), Centers for Disease Control and Prevention (CDC), US Census Bureau. United States National Health Interview Survey 2002. Hyattsville, United States: National Center for Health Statistics (NCHS), Centers for Disease Control and Prevention (CDC).	USA	National	27882	2002-2002	self-report
152994	Sutton-Tyrell, Kim, Faith Selzer, MaryFran Sowers, Robert Neer, Lynda Powell, Ellen Gold, Gail Greendale, Gerson Weiss, Karen Matthews, and Sonja McKinlay. Study of Women's Health Across the Nation (SWAN), 2001-2003: Visit 05 Dataset. ICPSR30501-v1. Ann Arbor, MI: Inter-university Consortium for Political and Social Research [distributor], 2014-09-02.	USA	National	2286	2002-2002	measured

	http://doi.org/10.3886/ICPSR30501.v1					
212526	Margarita Algeria, James S. Jackson, Ronald C. Kessler, and David Takeuchi. Collaborative Psychiatric Epidemiology Surveys (CPES), 2001-2003 [United States]. ICPSR20240-v7. Ann Arbor, MI: Inter-university Consortium for Political and Social Research [distributor], 2015-05-28. http://doi.org/10.3886/ICPSR20240.v7	USA	National	15379	2002-2002	measured
29963	Centers for Disease Control and Prevention (CDC). United States Behavioral Risk Factor Surveillance System 2001. Atlanta, Georgia: CDC, US Department of Health and Human Services.	USA	State + National	389870	2001-2001	self-report
41213	National Center for Health Statistics (NCHS), Centers for Disease Control and Prevention (CDC), US Census Bureau. United States National Health Interview Survey 2001. Hyattsville, United States: National Center for Health Statistics (NCHS), Centers for Disease Control and Prevention (CDC).	USA	National	30094	2001-2001	self-report
49205	National Center for Health Statistics (NCHS), Centers for Disease Control and Prevention (CDC). United States National Health and Nutrition Examination Survey 2001-2002. Hyattsville, United States: National Center for Health Statistics (NCHS), Centers for Disease Control and Prevention (CDC).	USA	National	9003	2001-2001	measured
86840	Panel Study of Income Dynamics, 2001 public use dataset. Produced and distributed by the University of	USA	National	7007	2001-2001	self-report

	Michigan with primary funding from the National Science Foundation, the National Institute of Aging, and the National Institute of Child Health and Human Development. Ann Arbor, MI, (2011).					
120179	Harris, Kathleen Mullan, and J. Richard Udry. National Longitudinal Study of Adolescent to Adult Health (Add Health), 1994-2008. ICPSR21600-v12. Ann Arbor, MI: Inter-university Consortium for Political and Social Research [distributor], 2013-03-08. doi:10.3886/ICPSR21600.v12	USA	National	9143	2001-2001	measured
124090	National Institute on Alcohol Abuse and Alcoholism (NIAAA), National Institutes of Health (NIH), U.S. Department of Health and Human Services. United States National Epidemiologic Survey on Alcohol and Related Conditions 2001-2002.	USA	National	41649	2001-2001	self-report
152993	Sutton-Tyrell, Kim, Faith Selzer, MaryFran Sowers, Robert Neer, Lynda Powell, Ellen Gold, Gail Greendale, Gerson Weiss, Karen Matthews, and Sonja McKinlay. Study of Women's Health Across the Nation (SWAN), 2000-2002: Visit 04 Dataset. ICPSR30142-v1. Ann Arbor, MI: Inter-university Consortium for Political and Social Research[distributor], 2014-02-13. http://doi.org/10.3886/ICPSR30142.v1	USA	National	2441	2001-2001	measured
163911	Centers for Disease Control and Prevention (CDC). United States National Youth Risk Behavior Survey 2001. Atlanta, United States of America:	USA	National	11500	2001-2001	self-report

	Centers for Disease Control and Prevention (CDC), 2002.					
169751	Aarhus University, Addiction Switzerland Research Institute, Alcohol Research Group, Public Health Institute, Centre for Addiction and Mental Health (Canada), Centre for Alcohol Policy Research, Turning Point Alcohol and Drug Centre (Australia), Kettil Bruun Society for Social and Epidemiological Research on Alcohol, University of North Dakota. United States Gender, Alcohol and Culture: An International Study (GENACIS) 2001.	USA	National	1108	2001-2001	self-report
191313	World Health Organization Regional Office for Europe (EURO-WHO). Health Behaviour in School-aged Children: WHO Collaborative Cross-National survey/study (HBSC) 2001-2002.	USA	National	1526	2001-2001	self-report
29958	Centers for Disease Control and Prevention (CDC). United States Behavioral Risk Factor Surveillance System 2000. Atlanta, Georgia: CDC, US Department of Health and Human Services.	USA	State + National	343012	2000-2000	self-report
41204	National Center for Health Statistics (NCHS), Centers for Disease Control and Prevention (CDC), US Census Bureau. United States National Health Interview Survey 2000. Hyattsville, United States: National Center for Health Statistics (NCHS), Centers for Disease Control and Prevention (CDC).	USA	National	29287	2000-2000	self-report
152992	Sutton-Tyrrell, Kim, Faith Selzer, MaryFran Sowers, Robert Neer, Lynda Powell, Ellen Gold, Gail Greendale, Gerson Weiss, Karen Matthews,	USA	National	2502	2000-2000	measured

	and Sonja McKinlay. Study of Women's Health Across the Nation (SWAN), 1999-2001: Visit 03 Dataset. ICPSR29701-v1. Ann Arbor, MI: Inter-university Consortium for Political and Social Research [distributor], 2014-02-12. http://doi.org/10.3886/ICPSR29701.v1					
169753	Aarhus University, Addiction Switzerland Research Institute, Alcohol Research Group, Public Health Institute, Centre for Addiction and Mental Health (Canada), Centre for Alcohol Policy Research, Turning Point Alcohol and Drug Centre (Australia), Kettil Bruun Society for Social and Epidemiological Research on Alcohol, University of North Dakota. United States Gender, Alcohol and Culture: An International Study (GENACIS) 2000.	USA	National	7143	2000-2000	self-report
29953	Centers for Disease Control and Prevention (CDC). United States Behavioral Risk Factor Surveillance System 1999. Atlanta, Georgia: CDC, US Department of Health and Human Services.	USA	State + National	299800	1999-1999	self-report
41195	National Center for Health Statistics (NCHS), Centers for Disease Control and Prevention (CDC), US Census Bureau. United States National Health Interview Survey 1999. Hyattsville, United States: National Center for Health Statistics (NCHS), Centers for Disease Control and Prevention (CDC).	USA	National	28080	1999-1999	self-report
52110	National Center for Health Statistics (NCHS), Centers for Disease Control and Prevention (CDC). United States National Health and Nutrition	USA	National	8456	1999-1999	measured

	Examination Survey 1999-2000. Hyattsville, United States: National Center for Health Statistics (NCHS), Centers for Disease Control and Prevention (CDC).					
86849	Panel Study of Income Dynamics, 1999 public use dataset. Produced and distributed by the University of Michigan with primary funding from the National Science Foundation, the National Institute of Aging, and the National Institute of Child Health and Human Development. Ann Arbor, MI, (2011).	USA	National	6708	1999-1999	self-report
152989	Sutton-Tyrrell, Kim, Faith Selzer, MaryFran Sowers, Robert Neer, Lynda Powell, Ellen Gold, Gail Greendale, Gerson Weiss, Karen Matthews, and Sonja McKinlay. Study of Women's Health Across the Nation (SWAN), 1998-2000: Visit 02 Dataset. ICPSR29401-v1. Ann Arbor, MI: Inter-university Consortium for Political and Social Research[distributor], 2014-02-06. http://doi.org/10.3886/ICPSR29401.v1	USA	National	2594	1999-1999	measured
163910	Centers for Disease Control and Prevention (CDC). United States National Youth Risk Behavior Survey 1999. Atlanta, United States of America: Centers for Disease Control and Prevention (CDC), 2000.	USA	National	13249	1999-1999	self-report
29948	Centers for Disease Control and Prevention (CDC). United States Behavioral Risk Factor Surveillance System 1998. Atlanta, Georgia: CDC, US Department of Health and Human Services.	USA	State + National	280089	1998-1998	self-report

41186	National Center for Health Statistics (NCHS), Centers for Disease Control and Prevention (CDC), US Census Bureau. United States National Health Interview Survey 1998. Hyattsville, United States: National Center for Health Statistics (NCHS), Centers for Disease Control and Prevention (CDC).	USA	National	29698	1998-1998	self-report
152988	Sutton-Tyrrell, Kim, Faith Selzer, MaryFran Sowers, Robert Neer, Lynda Powell, Ellen Gold, Gail Greendale, Gerson Weiss, Karen Matthews, and Sonja McKinlay. Study of Women's Health Across the Nation (SWAN), 1997-1999: Visit 01 Dataset. ICPSR29221-v1. Ann Arbor, MI: Inter-university Consortium for Political and Social Research[distributor], 2014-02-05. http://doi.org/10.3886/ICPSR29221.v1	USA	National	2765	1998-1998	measured
14406	Centers for Disease Control and Prevention (CDC). United States Behavioral Risk Factor Surveillance System 1997. Atlanta, Georgia: CDC, US Department of Health and Human Services.	USA	State + National	255405	1997-1997	self-report
41173	National Center for Health Statistics (NCHS), Centers for Disease Control and Prevention (CDC), US Census Bureau. United States National Health Interview Survey 1997. Hyattsville, United States: National Center for Health Statistics (NCHS), Centers for Disease Control and Prevention (CDC).	USA	National	33202	1997-1997	self-report
14421	Centers for Disease Control and Prevention (CDC). United States Behavioral Risk Factor	USA	State + National	233068	1996-1996	self-report

	Surveillance System 1996. Atlanta, Georgia: CDC, US Department of Health and Human Services.					
41126	National Center for Health Statistics (NCHS), Centers for Disease Control and Prevention (CDC), US Census Bureau. United States National Health Interview Survey 1996. Hyattsville, United States: National Center for Health Statistics (NCHS), Centers for Disease Control and Prevention (CDC).	USA	National	43395	1996-1996	self-report
120156	Harris, Kathleen Mullan, and J. Richard Udry. National Longitudinal Study of Adolescent to Adult Health (Add Health), 1994-2008. ICPSR21600-v12. Ann Arbor, MI: Inter-university Consortium for Political and Social Research [distributor], 2013-03-08. doi:10.3886/ICPSR21600.v12	USA	National	8534	1996-1996	measured
152970	Sutton-Tyrell, Kim, Faith Selzer, MaryFran Sowers, Robert Neer, Lynda Powell, Ellen Gold, Gail Greendale, Gerson Weiss, Karen Matthews, and Sonja McKinlay. Study of Women's Health Across the Nation (SWAN), 2001-2003: Visit 05 Dataset. ICPSR30501-v1. Ann Arbor, MI: Inter-university Consortium for Political and Social Research [distributor], 2014-09-02. http://doi.org/10.3886/ICPSR30501.v1	USA	National	15553	1996-1996	self-report
152971	Sutton-Tyrrell, Kim, Faith Selzer, MaryFran Sowers, Robert Neer, Lynda Powell, Ellen Gold, Gail Greendale, Gerson Weiss, Karen Matthews, and Sonja McKinlay. Study of Women's Health Across the	USA	National	3255	1996-1996	measured

	Nation (SWAN), 1996-1997: Baseline Dataset. ICPSR28762-v2. Ann Arbor, MI: Inter-university Consortium for Political and Social Research[distributor], 2014-02-04. http://doi.org/10.3886/ICPSR28762.v2					
196406	Agricultural Research Service, U.S. Department of Agriculture. United States USDA Continuing Survey of Food Intake by Individuals/Diet and Health Knowledge Survey 1994-1996 and 1998. Washington, D.C., United States of America: Agricultural Research Service, U.S. Department of Agriculture.	USA	National	10500	1996-1996	self-report
14426	Centers for Disease Control and Prevention (CDC). United States Behavioral Risk Factor Surveillance System 1995. Atlanta, Georgia: CDC, US Department of Health and Human Services.	USA	State + National	216703	1995-1995	self-report
41114	National Center for Health Statistics (NCHS), Centers for Disease Control and Prevention (CDC), US Census Bureau. United States National Health Interview Survey 1995. Hyattsville, United States: National Center for Health Statistics (NCHS), Centers for Disease Control and Prevention (CDC).	USA	National	70390	1995-1995	self-report
237619	Centers for Disease Control and Prevention (CDC). United States National College Youth Risk Behavior Survey 1995. Atlanta, United States of America: Centers for Disease Control and Prevention (CDC).	USA	National	2875	1995-1995	self-report
14436	Centers for Disease Control and Prevention (CDC). United States Behavioral Risk Factor	USA	State + National	201488	1994-1994	self-report

	Surveillance System 1994. Atlanta, Georgia: CDC, US Department of Health and Human Services.					
40821	National Center for Health Statistics (NCHS), Centers for Disease Control and Prevention (CDC), US Census Bureau. United States National Health Interview Survey 1994. Hyattsville, United States: National Center for Health Statistics (NCHS), Centers for Disease Control and Prevention (CDC).	USA	National	81396	1994-1994	self-report
14440	Centers for Disease Control and Prevention (CDC). United States Behavioral Risk Factor Surveillance System 1993. Atlanta, Georgia: CDC, US Department of Health and Human Services.	USA	State + National	195210	1993-1993	self-report
40766	National Center for Health Statistics (NCHS), Centers for Disease Control and Prevention (CDC), US Census Bureau. United States National Health Interview Survey 1993. Hyattville, United States: National Center for Health Statistics (NCHS), Centers for Disease Control and Prevention (CDC).	USA	National	77477	1993-1993	self-report
14444	Centers for Disease Control and Prevention (CDC). United States Behavioral Risk Factor Surveillance System 1992. Atlanta, Georgia: CDC, US Department of Health and Human Services.	USA	State + National	183366	1992-1992	self-report
40749	National Center for Health Statistics (NCHS), Centers for Disease Control and Prevention (CDC), US Census Bureau. United States National Health Interview Survey 1992. Hyattsville, United States:	USA	National	89930	1992-1992	self-report

	National Center for Health Statistics (NCHS), Centers for Disease Control and Prevention (CDC).					
14448	Centers for Disease Control and Prevention (CDC). United States Behavioral Risk Factor Surveillance System 1991. Atlanta, Georgia: CDC, US Department of Health and Human Services.	USA	State + National	167706	1991-1991	self-report
40330	National Center for Health Statistics (NCHS), Centers for Disease Control and Prevention (CDC), US Census Bureau. United States National Health Interview Survey 1991. Hyattsville, United States: National Center for Health Statistics (NCHS), Centers for Disease Control and Prevention (CDC).	USA	National	84625	1991-1991	self-report
48604	National Center for Health Statistics (NCHS), Centers for Disease Control and Prevention (CDC). United States National Health and Nutrition Examination Survey 1988-1994. Hyattsville, United States: National Center for Health Statistics (NCHS), Centers for Disease Control and Prevention (CDC).	USA	National	20798	1991-1991	measured
14451	Centers for Disease Control and Prevention (CDC). United States Behavioral Risk Factor Surveillance System 1990. Atlanta, Georgia: CDC, US Department of Health and Human Services.	USA	State + National	155967	1990-1990	self-report
40280	National Center for Health Statistics (NCHS), Centers for Disease Control and Prevention (CDC), US Census Bureau. United States National Health Interview Survey 1990. Hyattsville, United States:	USA	National	84739	1990-1990	self-report

	National Center for Health Statistics (NCHS), Centers for Disease Control and Prevention (CDC).					
14454	Centers for Disease Control and Prevention (CDC). United States Behavioral Risk Factor Surveillance System 1989. Atlanta, Georgia: CDC, US Department of Health and Human Services.	USA	State + National	125780	1989-1989	self-report
40229	National Center for Health Statistics (NCHS), Centers for Disease Control and Prevention (CDC), US Census Bureau. United States National Health Interview Survey 1989. Hyattsville, United States: National Center for Health Statistics (NCHS), Centers for Disease Control and Prevention (CDC).	USA	National	82738	1989-1989	self-report
14464	Centers for Disease Control and Prevention (CDC). United States Behavioral Risk Factor Surveillance System; 1988. Atlanta, Georgia: CDC, US Department of Health and Human Services.	USA	State + National	107746	1988-1988	self-report
40146	National Center for Health Statistics (NCHS), Centers for Disease Control and Prevention (CDC), US Census Bureau. United States National Health Interview Survey 1988. Hyattsville, United States: National Center for Health Statistics (NCHS), Centers for Disease Control and Prevention (CDC).	USA	National	86662	1988-1988	self-report
13320	United States Department of Health and Human Services. Centers for Disease Control and Prevention. National Center for Health Statistics. National Health Interview Survey: Longitudinal Study of	USA	National	7384<br><br>6510	1987-1987,<br><br>1984-1984	measured

	Aging, 70 Years and Over, 1984-1990. ICPSR08719-v7. Ann Arbor, MI: Inter-university Consortium for Political and Social Research [distributor], 2007-07-26. http://doi.org/10.3886/ICPSR08719.v7					
14468	Centers for Disease Control and Prevention (CDC). United States Behavioral Risk Factor Surveillance System 1987. Atlanta, Georgia: CDC, US Department of Health and Human Services.	USA	State + National	95570	1987-1987	self-report
40113	National Center for Health Statistics (NCHS), Centers for Disease Control and Prevention (CDC), US Census Bureau. United States National Health Interview Survey 1987. Hyattsville, United States: National Center for Health Statistics (NCHS), Centers for Disease Control and Prevention (CDC).	USA	National	86659	1987-1987	self-report
14472	Centers for Disease Control and Prevention (CDC). United States Behavioral Risk Factor Surveillance System 1986. Atlanta, Georgia: CDC, US Department of Health and Human Services.	USA	State + National	65512	1986-1986	self-report
40080	National Center for Health Statistics (NCHS), Centers for Disease Control and Prevention (CDC), US Census Bureau. United States National Health Interview Survey 1986. Hyattsville, United States: National Center for Health Statistics (NCHS), Centers for Disease Control and Prevention (CDC).	USA	National	43794	1986-1986	self-report
14492	Centers for Disease Control and Prevention (CDC). United States Behavioral Risk Factor	USA	State + National	47840	1985-1985	self-report

	Surveillance System 1985. Atlanta, Georgia: CDC, US Department of Health and Human Services.					
25274	Centers for Disease Control and Prevention (CDC). United States Behavioral Risk Factor Surveillance System 1984. Atlanta, Georgia: CDC, US Department of Health and Human Services	USA	State + National	23335	1984-1984	self-report
40019	National Center for Health Statistics (NCHS), Centers for Disease Control and Prevention (CDC), US Census Bureau. United States National Health Interview Survey 1984. Hyattsville, United States: National Center for Health Statistics (NCHS), Centers for Disease Control and Prevention (CDC).	USA	National	74427	1984-1984	self-report
39991	National Center for Health Statistics (NCHS), Centers for Disease Control and Prevention (CDC), US Census Bureau. United States National Health Interview Survey 1983. Hyattville, United States: National Center for Health Statistics (NCHS), Centers for Disease Control and Prevention (CDC).	USA	National	74680	1983-1983	self-report
39967	National Center for Health Statistics (NCHS), Centers for Disease Control and Prevention (CDC), US Census Bureau. United States National Health Interview Survey 1982. Hyattsville, United States: National Center for Health Statistics (NCHS), Centers for Disease Control and Prevention (CDC).	USA	National	73229	1982-1982	self-report
39765	National Center for Health Statistics (NCHS), Centers for Disease Control and Prevention	USA	National	73264	1980-1980	self-report

	(CDC), US Census Bureau. United States National Health Interview Survey 1980. Hyattville, United States: National Center for Health Statistics (NCHS), Centers for Disease Control and Prevention (CDC).					

Definition of overweight and obesity

For individuals aged 18 and above, we classified them as overweight if their BMI was 25 kg/m² or above but less than 30 kg/m². Individuals were considered obese if their BMI was 30 kg/m² or above. For individuals below the age of 18 years, we used monthly International Obesity Task Force (IOTF) cutoffs to determine overweight and obesity status when age in months was available. If age in months was unavailable, we assumed the midpoint of the birth year. Observations with a BMI less than 10 kg/m² or greater than 70 kg/m² were excluded, as these values were deemed biologically extreme.

Following the GBD 2021 study design, we used IOTF criteria given its international acceptance, enabling cross-country comparisons. In addition, IOTF cutoffs provide a consistent definition of overweight and obesity across the lifespan. At age 18, the IOTF criteria align with standard adult BMI cutoffs, facilitating continuous tracking through the transition into adulthood. In the USA, the CDC Growth Chart is a common standard for assessing overweight and obesity.[1] Based on BMI for age curves, a BMI at or above the 85th percentile and below the 95th percentile is defined as overweight, and a BMI above the 95th percentile is defined as obese.[2] While international studies have indicated discrepancies between CDC and IOTF definitions in estimating overweight and obesity prevalence,[3] studies in the USA have shown that the two approaches display reasonable correlation.[4,5]

Data Extraction

Individual BMI was calculated using weight and height information from survey data. Information related to location, year, age, and sex, were extracted. Relevant survey-design variables, including primary sampling unit, strata, and survey weights, were also extracted and used to aggregate individual-level microdata and produce accurate sample prevalence estimates and standard errors. In addition, three study-level covariates were captured to assess potential bias. These included 1) whether height and weight data were measured or self-reported, 2) whether the study was predominantly conducted in an urban area, rural area, or both, and 3) the level of representativeness of the study (national or subnational). Finally, to facilitate the quantification of data uncertainties, standard errors of the mean were derived from survey micro-data where available.

Data Standardisation and Adjustment

Self-report bias adjustment

Among all the data included in this analysis, 23.1% provided anthropometric measurements, while the remaining were self-reported data. The relationship between self-reported and measured data was examined across age groups. No consistent bias pattern was observed for child and adolescent data (ages 2 to 14), and only measured data were used for this age group. Consequently, no adjustments were made for the data from the 2-14 age group. For data obtained from individuals aged 15 and above,

a self-reported bias correction was applied. Specifically, a Metaregression-Bayesian, Regularised, Trimmed (MR-BRT) model[6] specific to the United States was developed to estimate the deviation between self-reported and measured values from the NHANES survey series. Self-report and measured NHANES data were matched on sex, 5-year age group, and study year (allowing for 3-year difference by study year). Using the logit difference between matched self-reported and measured data ($\text{logit}(prev_s) - \text{logit}(prev_m)$) as the outcome, bias correction coefficients were derived separately by sex with 5-year age group and decade as covariates and the unique study ID (NID) as the random intercept for the prevalence of overweight and obesity and the proportion of obesity among the overweight. The specific coefficients can be found in SM Tables 2 and 3.

SM Table 2: MR-BRT self-report crosswalk adjustment factors for the prevalence of overweight and obesity

Model	Data input	Reference or alternative case definition	Gamma	Beta coefficient, logit (95% UI) *
**Prevalence of Overweight and Obesity**				
Females	Measured NHANES data	Ref	0.0052	---
	Self-report (intercept)	Alt		0.08 (0.05, 0.11)
	Self-report (5-year age group)	Alt		–0.02 (–0.02, -0.01)
	Self-report data (decade)	Alt		0.00 (–0.02, 0.02)
Males	Measured NHANES data	Ref	0.016	---
	Self-report (intercept)	Alt		-0.42 (-0.46, -0.37)
	Self-report (5-year age group)	Alt		-0.003 (–0.005, -0.001)
	Self-report data (decade)	Alt		0.00 (–0.03, 0.03)

SM Table 3: MR-BRT self-report crosswalk adjustment factors for the proportion of obesity among the overweight

Model	Data input	Reference or alternative case definition	Gamma	Beta coefficient, logit (95% UI) *
**Proportion of Obesity among the Overweight**				
Females	Measured NHANES data	Ref	0.012	---
	Self-report (intercept)	Alt		-0.45 (-0.49, -0.41)
	Self-report (5-year age group)	Alt		0.003 (0.001, 0.004)
	Self-report data (decade)	Alt		0.01 (-0.02, 0.04)
Males	Measured NHANES data	Ref	0.018	---
	Self-report (intercept)	Alt		-0.46 (-0.50, -0.41)
	Self-report (5-year age group)	Alt		0.00 (–0.002, 0.001)
	Self-report data (decade)	Alt		0.01 (–0.02, 0.04)

**MR-BRT bias adjustments can be interpreted as the factor the alternative case definition is adjusted by to reflect what it would have been had it been measured using the reference case definition. If the log/logit beta coefficient is negative, then the alternative is adjusted up to the reference. If the log/logit beta coefficient is positive, then the alternative is adjusted down to the reference.*

Prevalence estimation for overweight and obesity

Spatiotemporal Gaussian process regression (ST-GPR) was used to estimate the prevalence of overweight and obesity by age, and sex at the national and state levels. This modelling approach has been described in detail elsewhere.[7] Briefly, ST-GPR utilises spatial and temporal correlation information and incorporates weighted residuals to synthesise data from disparate sources and generate consistent estimates over time and across geographies. There are two key analytical components in ST-GPR, a mean function and the covariance matrix. In this analysis, the mean function was derived from a linear mixed-effects model with added residual smoothing. Separate models were used for overweight prevalence and for the proportion of obesity among the population with overweight and obesity, which was subsequently used to calculate the prevalence of obesity. The national-level estimates were derived as part of the global model from GBD 2021 analysis. The specific formulas are presented below:

$$\mathrm{logit(overweight)}_{c,a,t} = \beta_0 + \beta_1 \mathrm{educ}_{c,t} + \beta_2 \mathrm{urban}_{c,t} + \beta_3 \mathrm{agriculture}_{c,t} + \sum_{k=1}^{16} \beta_k I_{A[a]} + \alpha_s + \alpha_r + \alpha_c$$

$$\mathrm{logit(obesity/overweight)}_{c,a,t} = \beta_0 + \beta_1 \mathrm{educ}_{c,t} + \beta_2 \mathrm{urban}_{c,t} + \beta_3 \mathrm{agriculture}_{c,t} + \sum_{k=1}^{16} \beta_k I_{A[a]} + \alpha_s + \alpha_r + \alpha_c$$

where educ is the age-standardised level of educational attainment*;* urban is the proportion of the population living in an urban area*; and* agriculture is the proportion of the population working in agriculture. $I_{A[a]}$ is a dummy variable indicating the specific age group a that the prevalence point captures, and α_s, α_r and α_c are the super-region, region and country random intercepts, respectively. Separate models were fitted for each sex. Models with similar structures were used for state-level estimation, except there was only one state-level random intercept.

Covariates included in the models were selected based on a systematic variable selection process. The initial set of covariates consisted of ten-year lag-distributed energy per capita, the proportion of the population living in urban areas, Socio-demographic Index (SDI), lag-distributed income per capita, educational attainment (years) per capita, the proportion of the population working in agriculture, grams of sugar adjusted for energy per capita, grams of sugar not adjusted for energy per capita, and the number of two- or four-wheeled vehicles per capita. Models involving different combinations of these covariates were assessed based on three criteria: 1) model face validity, wherein the direction of coefficients was required to be consistent with the theoretical understanding of the correlations; 2) statistically significant coefficients; and 3) low in-sample Akaike Information Criterion (AIC). The covariate selection process was performed using the *dredge* package in R.

Forecast Modelling

To forecast the prevalence of overweight and obesity, as well as the proportion of obesity among the overweight, we used a generalised ensemble modelling approach (GenEM) which consisted of 12 different submodels.[8] For the submodels, we employed two main approaches: annualised rate of change (ARC) and a two-stage spline model based on the Meta-Regression Bayesian Regularised Trimmed Tool (MR-BRT).[9] Each of these models had 6 different recency-weighting parameters[10] ranging from 0 to 2.5, with higher values giving more weight to recent years.[10]

For the ARC submodels, we calculated the sex-and-location specific annualised rate of change for the logit-transformed age-standardised prevalence of overweight and obesity and the logit-transformed

age-standardised proportion of obesity among the overweight. The annual change values are winsorised by replacing outliers with the closest 2.5th and 97.5th percentile values. As for the two-stage MR-BRT submodels, they involved first fitting a sex-specific logit spline model separately for the age-standardised prevalence of overweight and obesity and the age-standardised proportion of obesity among the overweight against the socio-demographic index (SDI):

$$logit(\text{overweight}_{s,t,m}) = \beta_0 + \beta_1 spline(SDI_{t,m}) + \varepsilon_{s,t,m}$$

$$logit(\text{obesity/overweight}_{s,t,m}) = \beta_0 + \beta_1 spline(SDI_{t,m}) + \varepsilon_{s,t,m}$$

where $logit(\text{overweight}_{s,t,m})$ is the logit of the overweight and obesity age-standardised prevalence and $logit(\text{obesity/overweight}_{s,t,m})$ is the age-standardised proportion of obesity among the overweight, for sex s, year t, and state m. β_0 is the intercept, β_1 is a coefficient matrix; $spline$ is the piecewise polynomial function with five knots evenly placed across the curve and with the assumption of right and left linear tails; and $\varepsilon_{s,t,m}$ refers to the residuals. The second stage of the model involved fitting a logit model of the residuals from the first stage model on time:

$$logit(\varepsilon_{s,t,m}) = \beta'_0 + \beta'_1 t + \epsilon_{s,t,m}$$

where β'_0 is the fixed intercept, β'_1 is the coefficient for time in years and $\epsilon_{s,t,m}$ is an error term. We also ran the models separately for adult (aged 25 +) and child (aged 2-24, 2-14, and 15-24) age groups to differentiate the trends between younger and older populations.

To create the ensemble model, each submodel was weighted based on predictive errors. Specifically, out-of-sample cross-validation was performed where all submodels were trained using data from 1990-2011. Prediction errors, defined by root mean square errors (RMSE), were calculated based on a 10-year holdout period from 2012 to 2021. Subsequently, model weights based on the inverse of their RMSE were derived, with models demonstrating better out-of-sample predictive performance receiving higher weights.

For the final forecast, submodels were trained using the complete dataset from 1990-2021. For each ARC submodel, we used the calculated annualised rate of change with corresponding recency-weighting parameters to derive prevalence estimates for 2022-2050. For the MR-BRT submodels, forecast SDI values for 2022-2050 and recency weights were used to obtain forecasted age-standardised prevalence values. The forecasted age-standardised prevalence draws from all models were combined using predictive performance weights. The age-specific forecasted prevalence results were obtained by applying the age weights to the forecasted age-standardised prevalence. The forecasted prevalence of obesity was then calculated by multiplying the forecasted prevalence of overweight and obesity by the forecasted proportion of obesity among the overweight. The forecasted prevalence of overweight excluding obesity was calculated by subtracting prevalence of obesity from prevalence of overweight and obesity. Population forecasts from 2022 to 2050 were used to compute forecasted count values and the aggregated results by location, age and sex. All final forecast values were calculated as the mean of the 500 draws from the posterior distribution, and uncertainty estimates were derived from the 2.5th and 97.5th percentiles of the distribution (See SM Figure 1). Additional details on these methods can be found in Vollset et al, 2024.[8]

GATHER Checklist

Item #	Checklist item	Reporting location
Objectives and funding		
1	Define the indicator(s), populations (including age, sex, and geographic entities), and time period(s) for which estimates were made.	Main text methods overview, paragraphs 1 and 2
2	List the funding sources for the work.	Main text method section “role of the funding source”
Data Inputs		
For all data inputs from multiple sources that are synthesized as part of the study:		
3	Describe how the data were identified and how the data were accessed.	Main text methods section “Data sources” paragraph 1
4	Specify the inclusion and exclusion criteria. Identify all ad-hoc exclusions.	Exclusion criteria summarised in Methods section “Data sources”: paragraphs 1 and 2
5	Provide information on all included data sources and their main characteristics. For each data source used, report reference information or contact name/institution, population represented, data collection method, year(s) of data collection, sex and age range, diagnostic criteria or measurement method, and sample size, as relevant.	Appendix 1 Table SM 1pp. 3-30; citations also provided via the Global Health Data Exchange (GHDx) (https://ghdx.healthdata.org/);
6	Identify and describe any categories of input data that have potentially important biases (e.g., based on characteristics listed in item 5).	Main text method section “Data standardisation” and Appendix 1 pp 30-31
For data inputs that contribute to the analysis but were not synthesized as part of the study:		
7	Describe and give sources for any other data inputs.	N/A
For all data inputs:		
8	Provide all data inputs in a file format from which data can be efficiently extracted (e.g., a spreadsheet rather than a PDF), including all relevant meta-data listed in item 5. For any data inputs that cannot be shared because of ethical or legal reasons, such as third-party ownership, provide a contact name or the name of the institution that retains the right to the data.	Data inputs in excel format available via the GHDx (https://ghdx.healthdata.org/);
Data analysis		
9	Provide a conceptual overview of the data analysis method. A diagram may be helpful.	Main text methods “overview” and SM Figure 1
10	Provide a detailed description of all steps of the analysis, including mathematical formulae. This description should cover, as relevant, data cleaning, data pre-processing, data adjustments and weighting of data sources, and mathematical or statistical model(s).	Main text methods; Appendix 1 pp. 32-33 and SM Figure 1
11	Describe how candidate models were evaluated and how the final model(s) were selected.	Main text methods with reference to previous publications containing details on model validation; Appendix 1 pp. 33
12	Provide the results of an evaluation of model performance, if done, as well as the results of any relevant sensitivity analysis.	N/A
13	Describe methods for calculating uncertainty of the estimates. State which sources of uncertainty were, and were not, accounted for in the uncertainty analysis.	Main text methods section “Estimation of prevalence of overweight and obesity from 1990 to 2021” and Appendix 1 pp. 33.

14	State how analytic or statistical source code used to generate estimates can be accessed.	See data sharing section
Results and Discussion		
15	Provide published estimates in a file format from which data can be efficiently extracted.	The results can be efficiently extracted at https://vizhub.healthdata.org/gbd-results/
16	Report a quantitative measure of the uncertainty of the estimates (e.g. uncertainty intervals).	UIs given for all findings, including in the text, figures, and tables in the main text and appendix 2; online viz tools (see information above)
17	Interpret results in light of existing evidence. If updating a previous set of estimates, describe the reasons for changes in estimates.	Main text discussion section paragraphs 1-8
18	Discuss limitations of the estimates. Include a discussion of any modelling assumptions or data limitations that affect interpretation of the estimates.	Main text discussion paragraph 9

References

1 Growth Charts - CDC Extended BMI-for-Age Growth Charts - Download. 2022; published online Dec 21. https://www.cdc.gov/growthcharts/Extended-BMI-Charts.html (accessed May 17, 2024).

2 Ogden CL. Defining overweight in children using growth charts. *Md Med* 2004; **5**: 19–21.

3 Llorca-Colomer F, Murillo-Llorente MT, Legidos-García ME, Palau-Ferré A, Pérez-Bermejo M. Differences in Classification Standards For the Prevalence of Overweight and Obesity in Children. A Systematic Review and Meta-Analysis. *Clin Epidemiol* 2022; **14**: 1031–52.

4 Li K, Haynie D, Palla H, Lipsky L, Iannotti RJ, Simons-Morton B. Assessment of adolescent weight status: Similarities and differences between CDC, IOTF, and WHO references. *Preventive Medicine* 2016; **87**: 151–4.

5 Lobstein T, Jackson-Leach R. Child overweight and obesity in the USA: Prevalence rates according to IOTF definitions. *International Journal of Pediatric Obesity* 2007; **2**: 62–4.

6 Zheng P, Afshin A, Biryukov S, *et al.* The Burden of Proof studies: assessing the evidence of risk. *Nat Med* 2022; **28**: 2038–44.

7 Ng M, Fleming T, Robinson M, *et al.* Global, regional, and national prevalence of overweight and obesity in children and adults during 1980–2013: a systematic analysis for the Global Burden of Disease Study 2013. *The Lancet* 2014; **384**: 766–81.

8 Vollset SE, Ababneh HS, Abate YH, *et al.* Burden of disease scenarios for 204 countries and territories, 2022–2050: a forecasting analysis for the Global Burden of Disease Study 2021. *The Lancet* 2024; **403**: 2204–56.

9 Zheng P, Barber R, Sorensen RJD, Murray CJL, Aravkin AY. Trimmed Constrained Mixed Effects Models: Formulations and Algorithms. *Journal of Computational and Graphical Statistics* 2021; **30**: 544–56.

10 Foreman KJ, Marquez N, Dolgert A, *et al.* Forecasting life expectancy, years of life lost, and all-cause and cause-specific mortality for 250 causes of death: reference and alternative scenarios for 2016–40 for 195 countries and territories. *The Lancet* 2018; **392**: 2052–90.
