# Supplementary material for: National-level and state-level prevalence of overweight and obesity among children, adolescents, and adults in the USA, 1990–2021, and forecasts up to 2050
Source: Lancet. 2024 Dec 7;404(10469):2278–98. doi: 10.1016/S0140-6736(24)01548-4 (PMC11694015; doi:10.1016/S0140-6736(24)01548-4)
Supplement: Supplementary appendix 2 [file mmc2.pdf]

THE LANCET

Supplementary appendix 2

This appendix formed part of the original submission and has been peer reviewed. We post it as supplied by the authors.

Supplement to: GBD 2021 US Obesity Forecasting Collaborators. National-level and state-level prevalence of overweight and obesity among children, adolescents, and adults in the USA, 1990–2021, and forecasts up to 2050. *Lancet* 2024; published online Nov 14. https://doi.org/10.1016/S0140-6736(24)01548-4.

Appendix 2: Supplementary Results

SM Figure 1: Ensemble modelling framework for overweight and obesity forecast

SM Figure 2: Estimated age-standardised prevalence of overweight and obesity in 50 US states and Washington, DC, in 2021, for adolescents ages 15-24, both sexes

SM Figure 3: Sex-specific prevalence of overweight and obesity (BMI>=25), by age group, in 2021 in the USA. Shaded areas indicate 95% uncertainty intervals

SM Figure 4: Estimated age-standardised prevalence of obesity in 50 states and Washington DC, in 2021(A) adolescent males, ages 15-24 years, (B) adolescent females, ages 15-24 years, (C) adult males, ages 25+, (D) adult females, ages 25+

SM Figure 5: Changes in obesity prevalence from 1990 to 2021 and from 2021 to 2050 in the USA for (A) adolescent males, ages 15-24 years, (B) adolescent females, ages 15-24 years, (C) both sexes ages 15-24 years

SM Figure 6: Changes in obesity prevalence from 1990 to 2021 and from 2021 to 2050 in the USA for (A) adult males, ages 25+, (B) adult females, ages 25+, (C) both sexes, ages 25+

SM Figure 7: Distribution of adolescents and adults with overweight and obesity across 50 states and Washington DC in 1990, 2021 and 2025

SM Figure 8: Distribution of adolescents and adults with obesity across 50 states and Washington DC in 1990, 2021 and 2025

SM Figure 9: Prevalence of overweight and obesity by age across birth cohorts for (A) males and (B) females in USA. Prevalence for each 5-year birth cohort was deduced from the estimated and projected prevalence of the corresponding 5-year age group for each 5-year period from 1990 to 2050.

SM Figure 10: Estimated age-standardised prevalence of overweight and obesity among (A) adolescents ages 15-24 and (B) adults ages 25+ with the associated relative percentage change at the national level, across 50 states and Washington DC

SM Table 4: Prevalence of overweight and obesity by 5-year age group and sex in 1990, 2021 and 2050 at the national level, across 50 states and Washington DC

SM Table 5: Prevalence of obesity by 5-year age group and sex in 1990, 2021 and 2050 at the national level, across 50 states and Washington DC

SM Table 6: Number of population with overweight and obesity among adolescents ages 15-24 and adults ages 25+ by sex in 1990, 2021 and 2050 at the national level, across 50 states and Washington DC

SM Figure 1: Ensemble modelling framework for overweight and obesity forecast

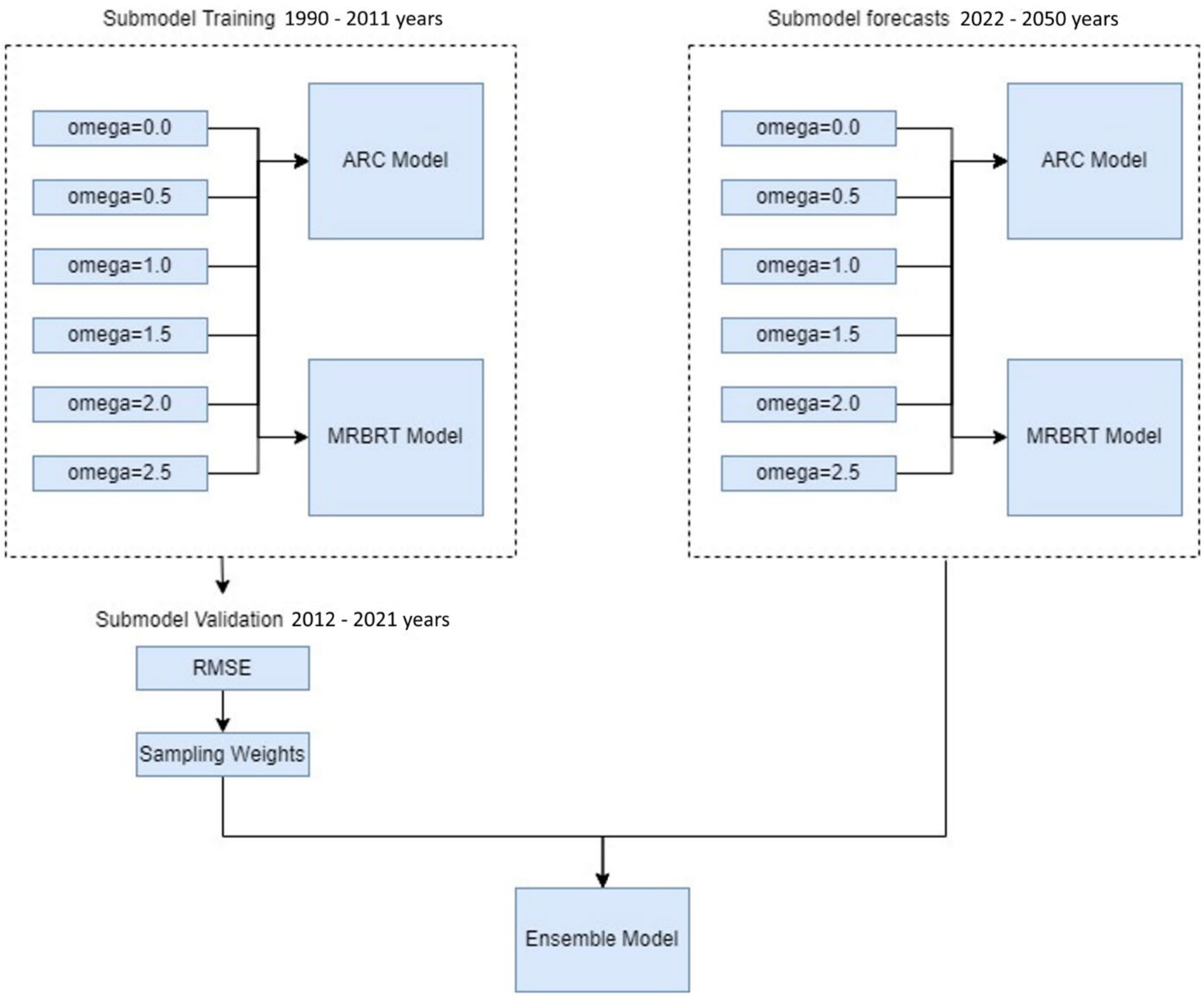

SM Figure 2: Estimated age-standardised prevalence of overweight and obesity in 50 US states and Washington, DC, in 2021, for adolescents ages 15-24, both sexes

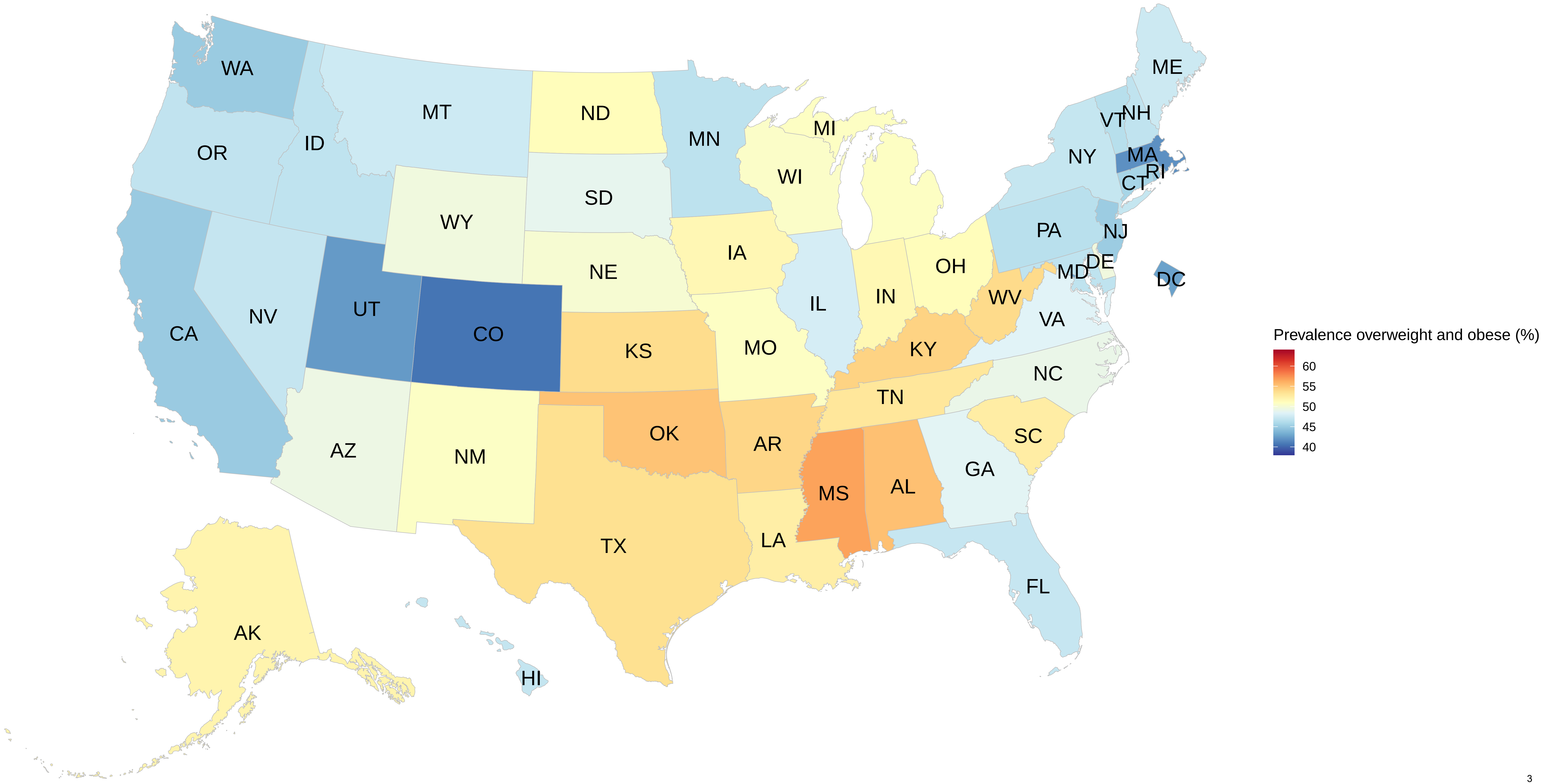

SM Figure 3: Sex-specific prevalence of overweight and obesity (BMI>=25), by age group, in 2021 in the USA. Shaded areas indicate 95% uncertainty intervals

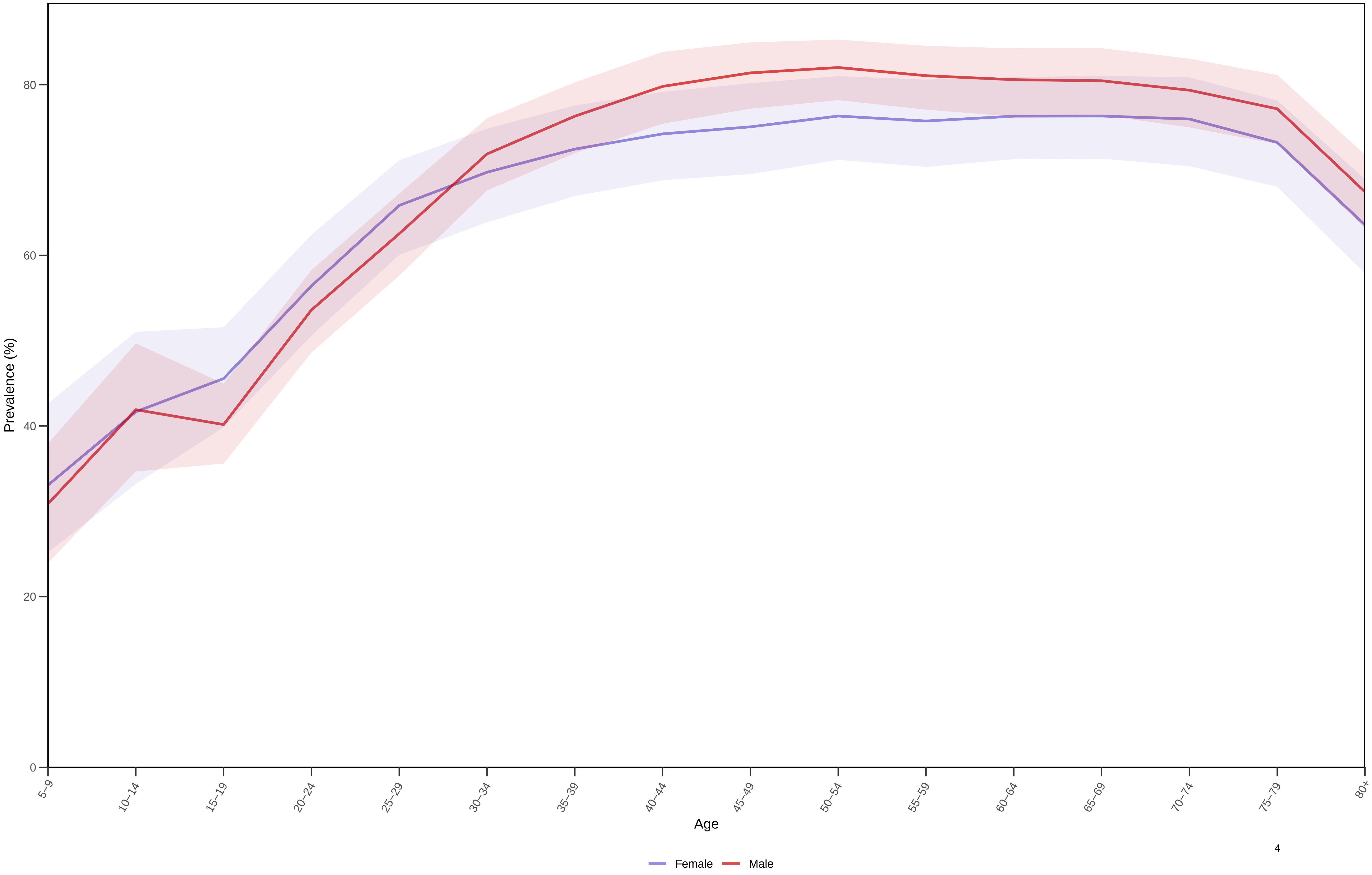

SM Figure 4: Estimated age-standardised prevalence of obesity in 50 states and Washington DC, in 2021 (A) adolescent males, ages 15-24 years, (B) adolescent females, ages 15-24 years, (C) adult males, ages 25+, (D) adult females, ages 25+

(A) Age−standardised prevalence of obesity among adolescent males, ages 15-24 years, 2021

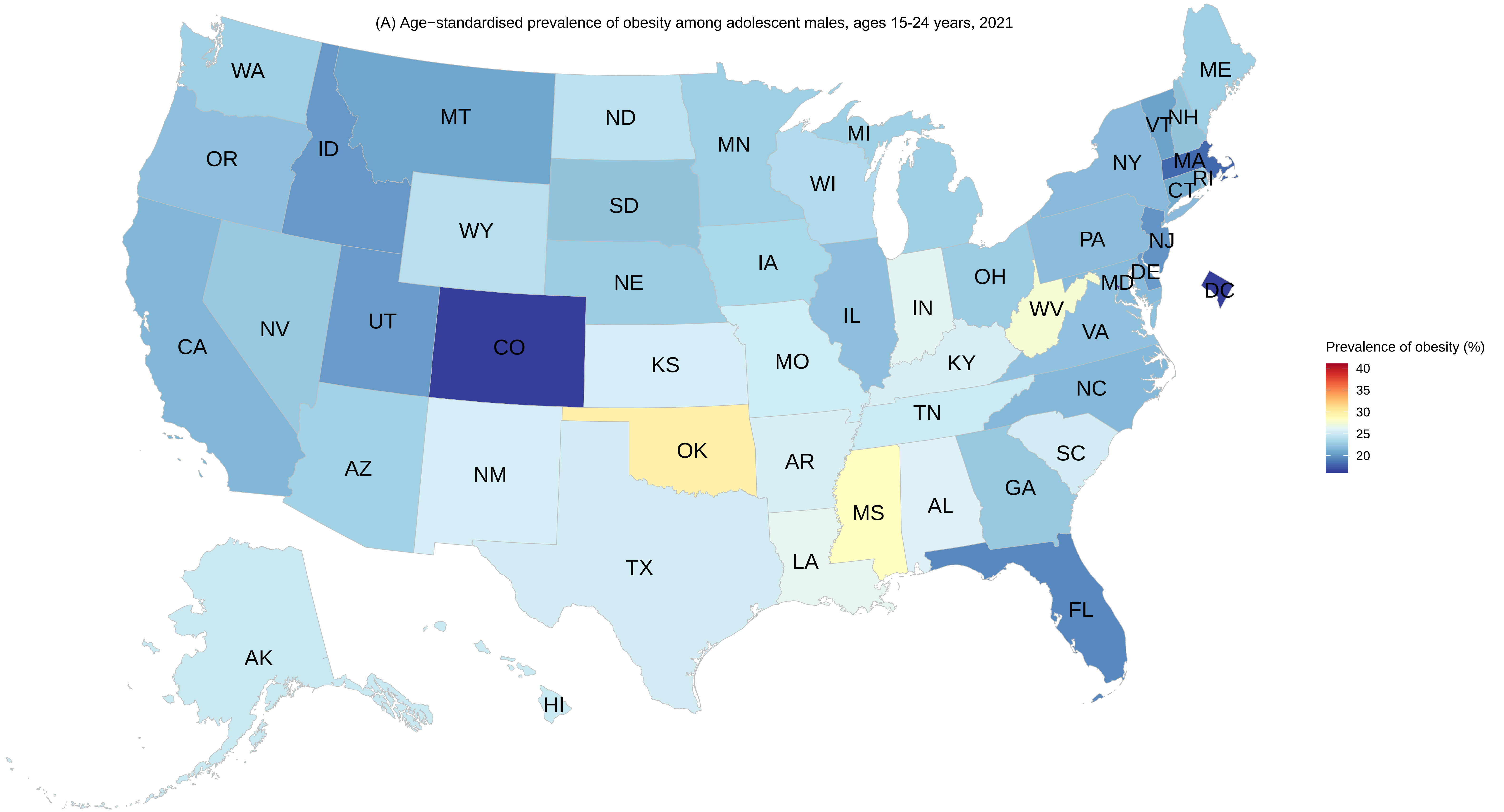

SM Figure 4: Estimated age-standardised prevalence of obesity in 50 states and Washington DC, in 2021 (A) adolescent males, ages 15-24 years, (B) adolescent females, ages 15-24 years, (C) adult males, ages 25+, (D) adult females, ages 25+

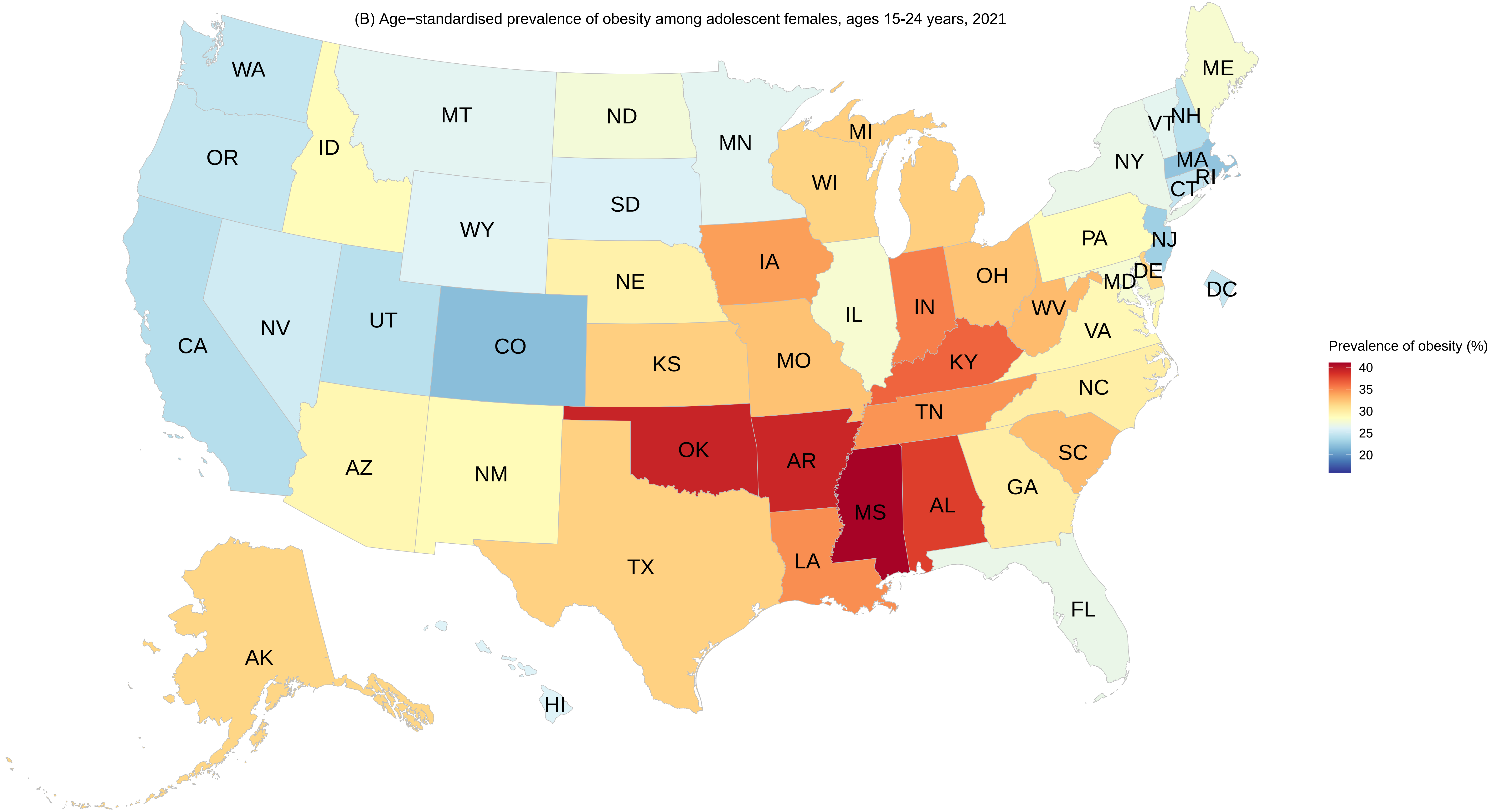

SM Figure 4: Estimated age-standardised prevalence of obesity in 50 states and Washington DC, in 2021 (A) adolescent males, ages 15-24 years, (B) adolescent females, ages 15-24 years, (C) adult males, ages 25+, (D) adult females, ages 25+

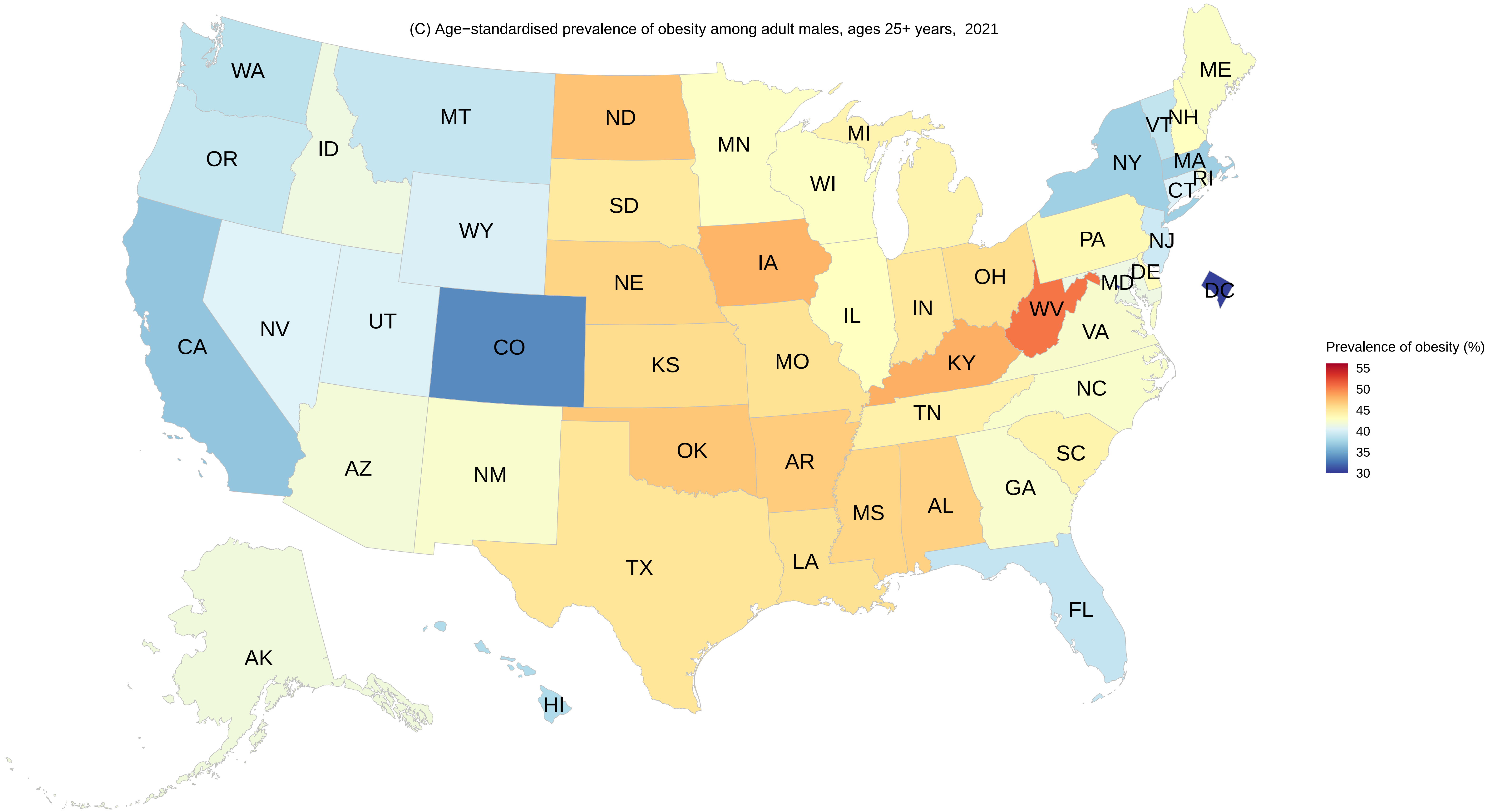

SM Figure 4: Estimated age-standardised prevalence of obesity in 50 states and Washington DC, in 2021 (A) adolescent males, ages 15-24 years, (B) adolescent females, ages 15-24 years, (C) adult males, ages 25+, (D) adult females, ages 25+

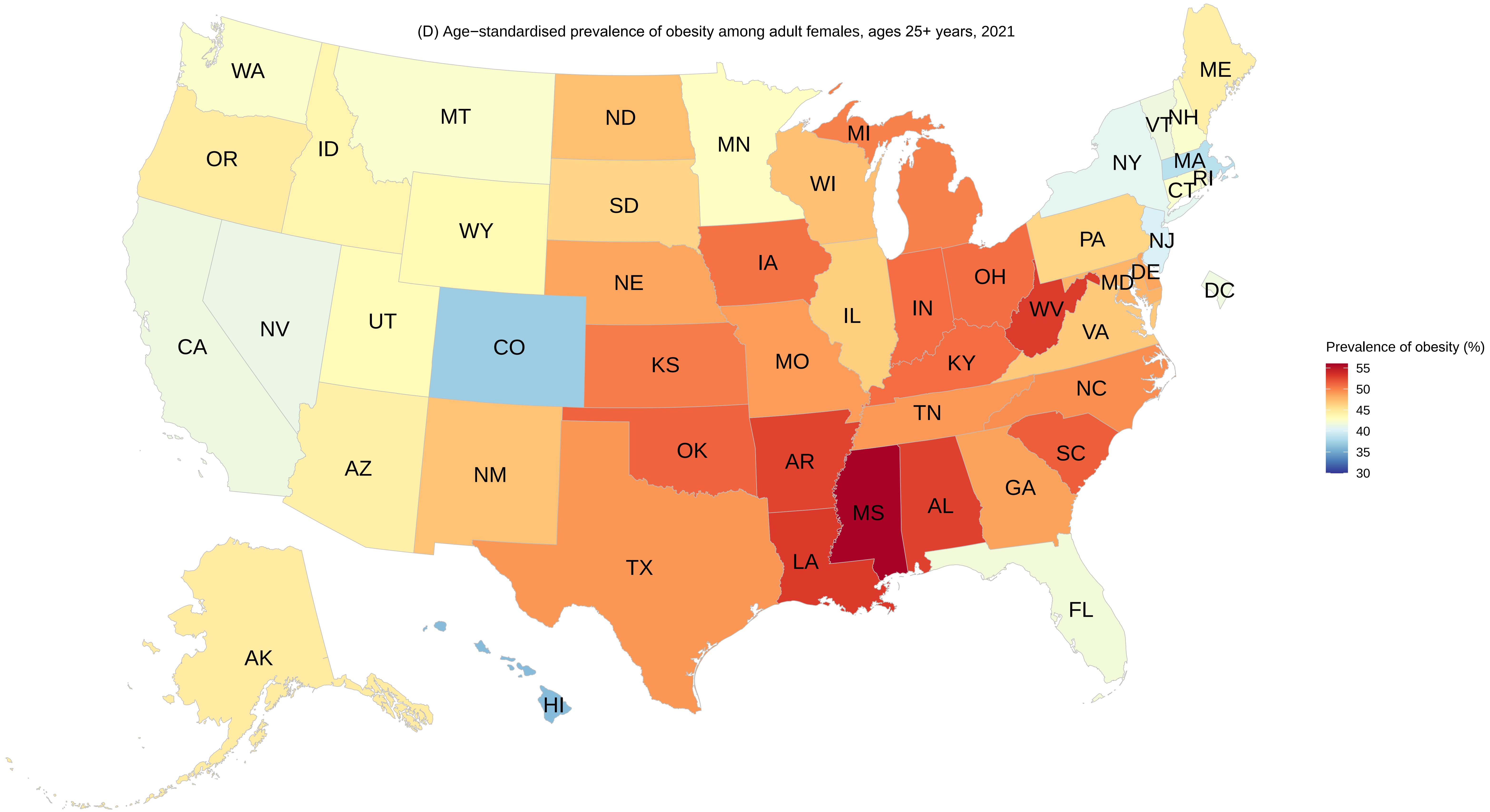

SM Figure 5: Changes in obesity prevalence from 1990 to 2021 and from 2021 to 2050 in the USA for (A) adolescent males, (B) adolescent females, (C) both sexes, ages 15-24 years

(A) Percent change in obesity prevalence between 1990 and 2021 among adolescent males, ages 15-24 years

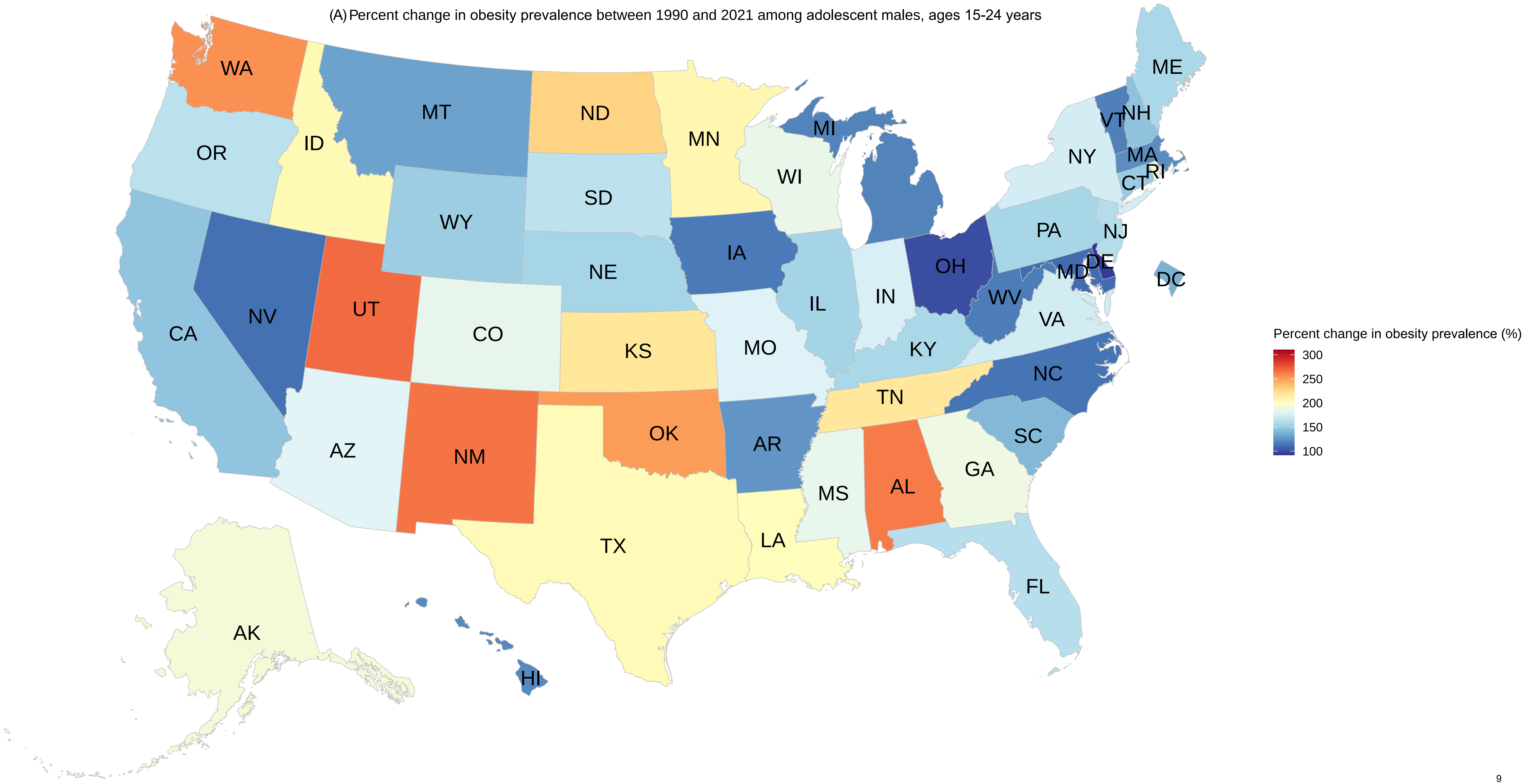

SM Figure 5: Changes in obesity prevalence from 1990 to 2021 and from 2021 to 2050 in the USA for (A) adolescent males, (B) adolescent females, (C) both sexes, ages 15-24 years

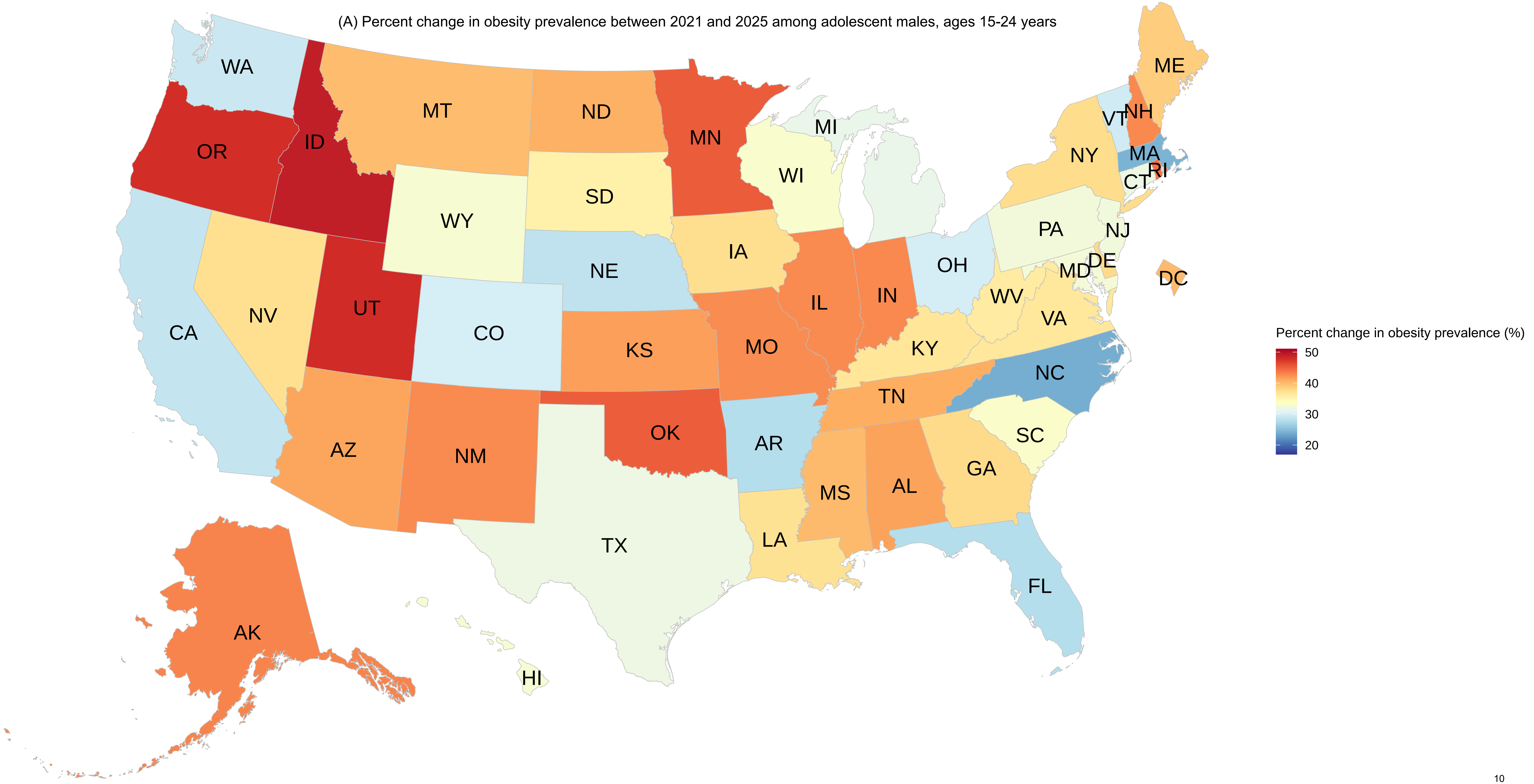

SM Figure 5: Changes in obesity prevalence from 1990 to 2021 and from 2021 to 2050 in the USA for (A) adolescent males, (B) adolescent females, (C) both sexes, ages 15-24 years

(B) Percent change in obesity prevalence between 1990 and 2021 among adolescent females, ages 15-24 years

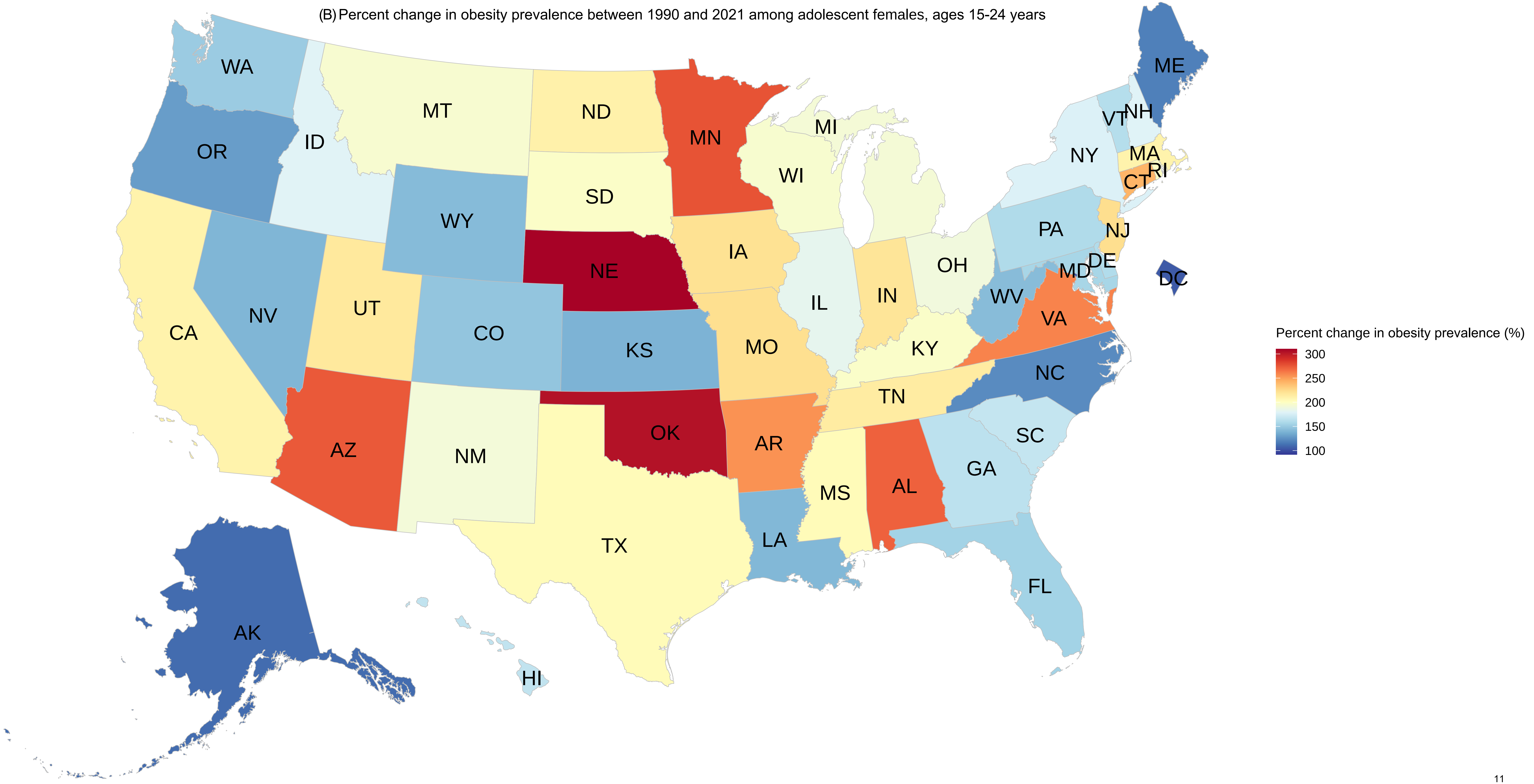

SM Figure 5: Changes in obesity prevalence from 1990 to 2021 and from 2021 to 2050 in the USA for (A) adolescent males, (B) adolescent females, (C) both sexes, ages 15-24 years

(B) Percent change in obesity prevalence between 2021 and 2025 among adolescent females, ages 15-24 years

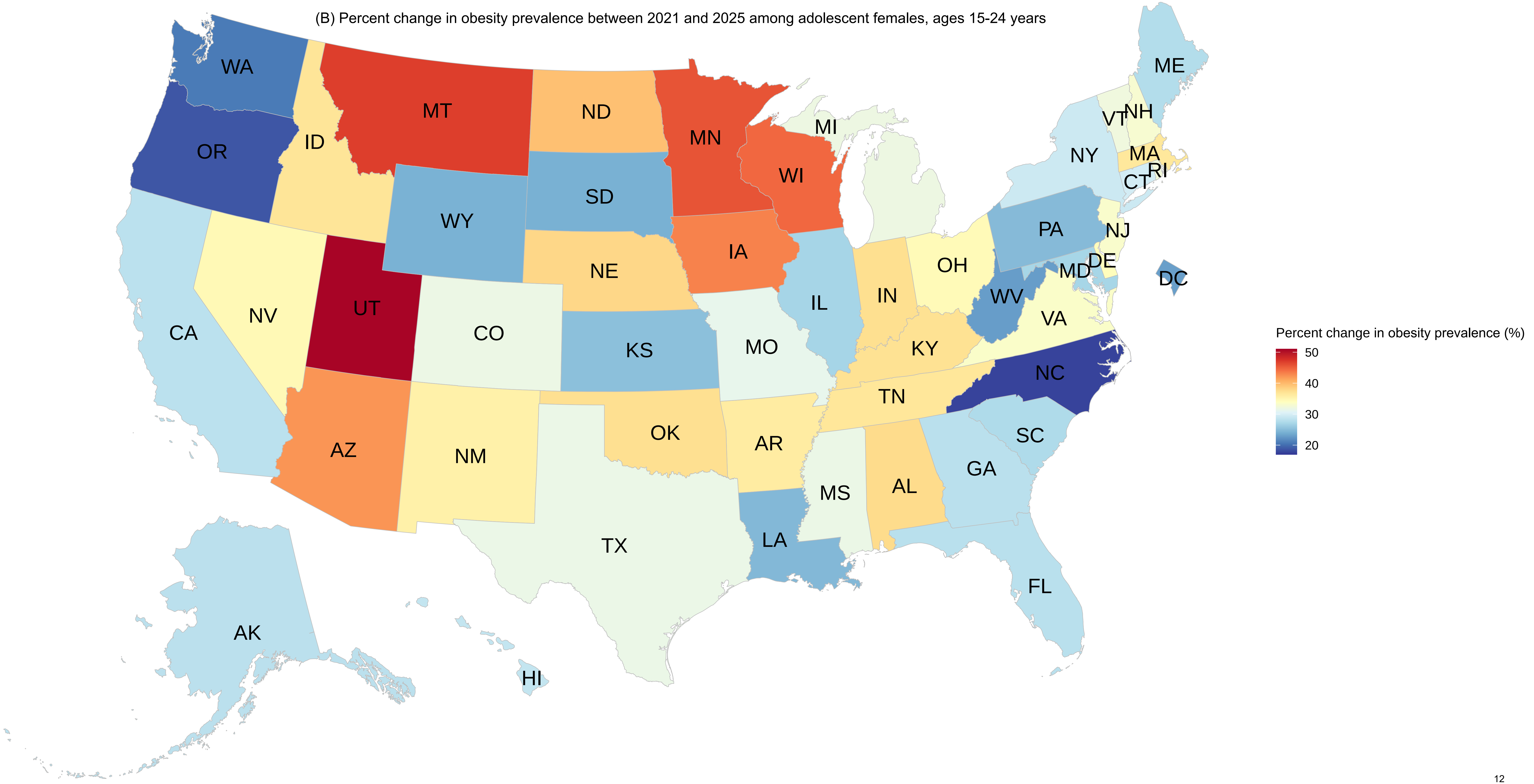

SM Figure 5: Changes in obesity prevalence from 1990 to 2021 and from 2021 to 2050 in the USA for (A) adolescent males, (B) adolescent females, (C) both sexes, ages 15-24 years

(C) Percent change in obesity prevalence between 1990 and 2021 among adolescent, both sexes, ages 15-24

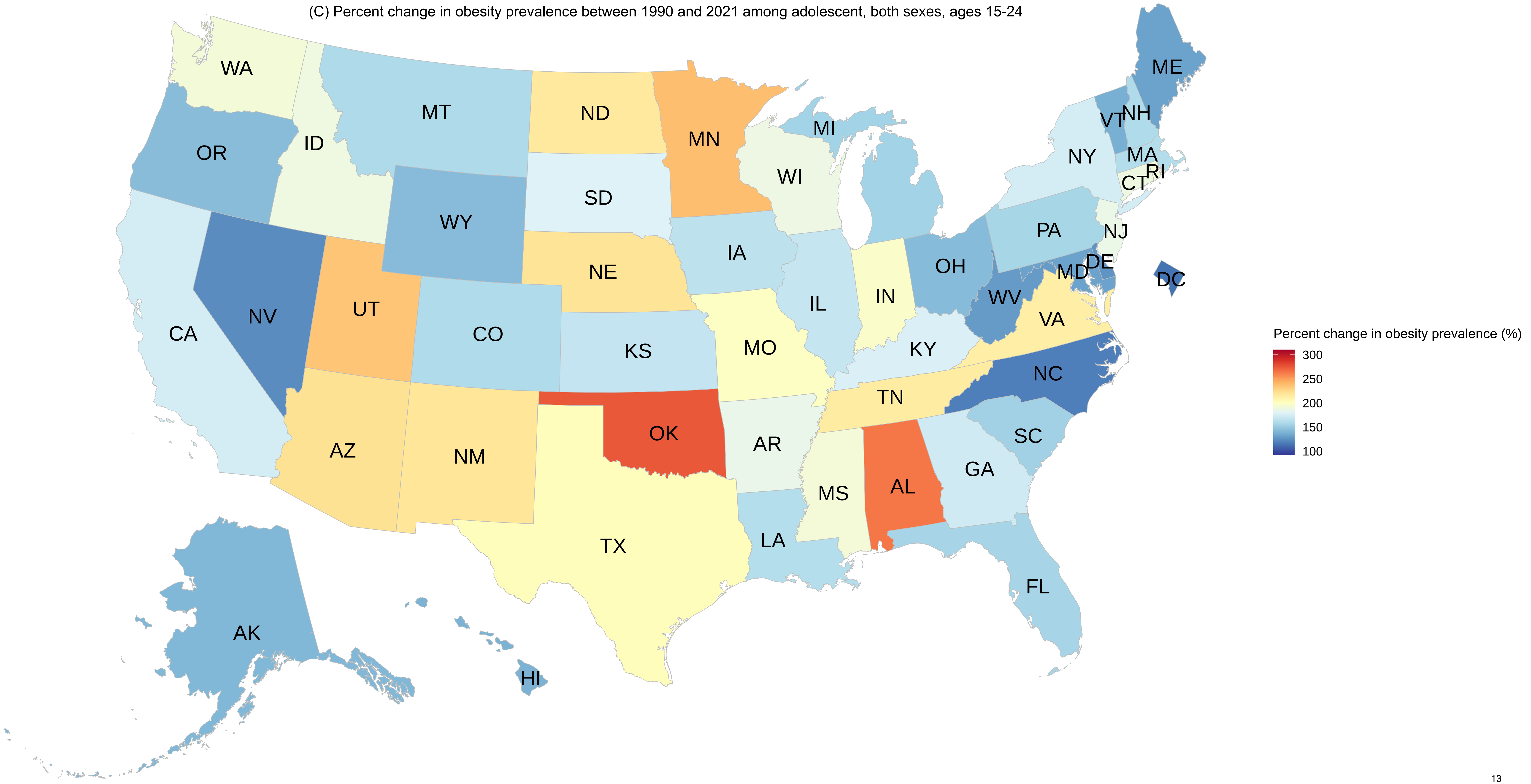

SM Figure 5: Changes in obesity prevalence from 1990 to 2021 and from 2021 to 2050 in the USA for (A) adolescent males, (B) adolescent females, (C) both sexes, ages 15-24 years

(C) Percent change in obesity prevalence between 2021 and 2050 among adolescent, both sexes, ages 15-24

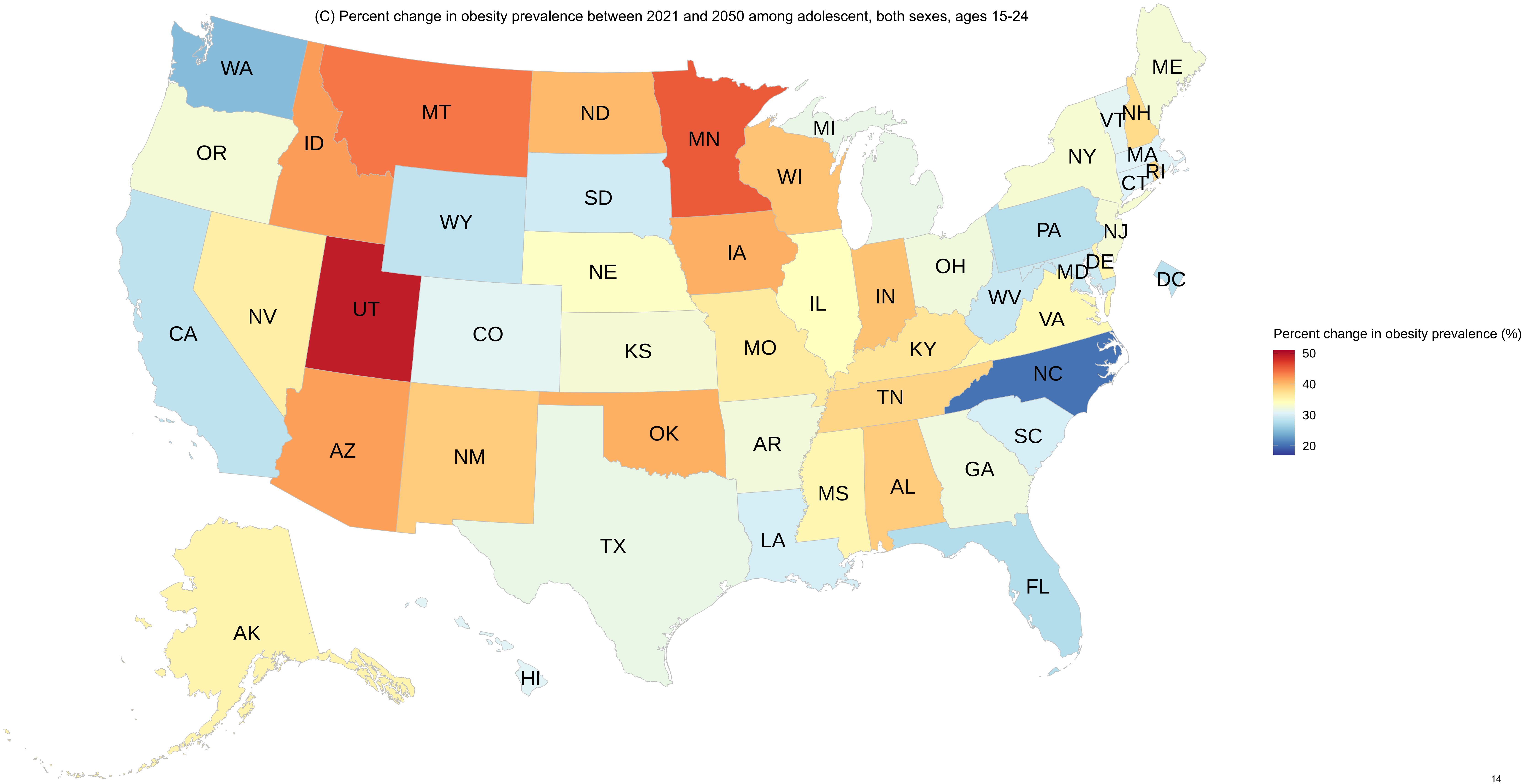

SM Figure 6: Changes in obesity prevalence from 1990 to 2021 and from 2021 to 2050 in the USA for (A) adult males, (B) adult females, (C) both sexes, ages 25+ years

(A) Percent change in obesity prevalence between 1990 and 2021 among adult males, ages 25+ years

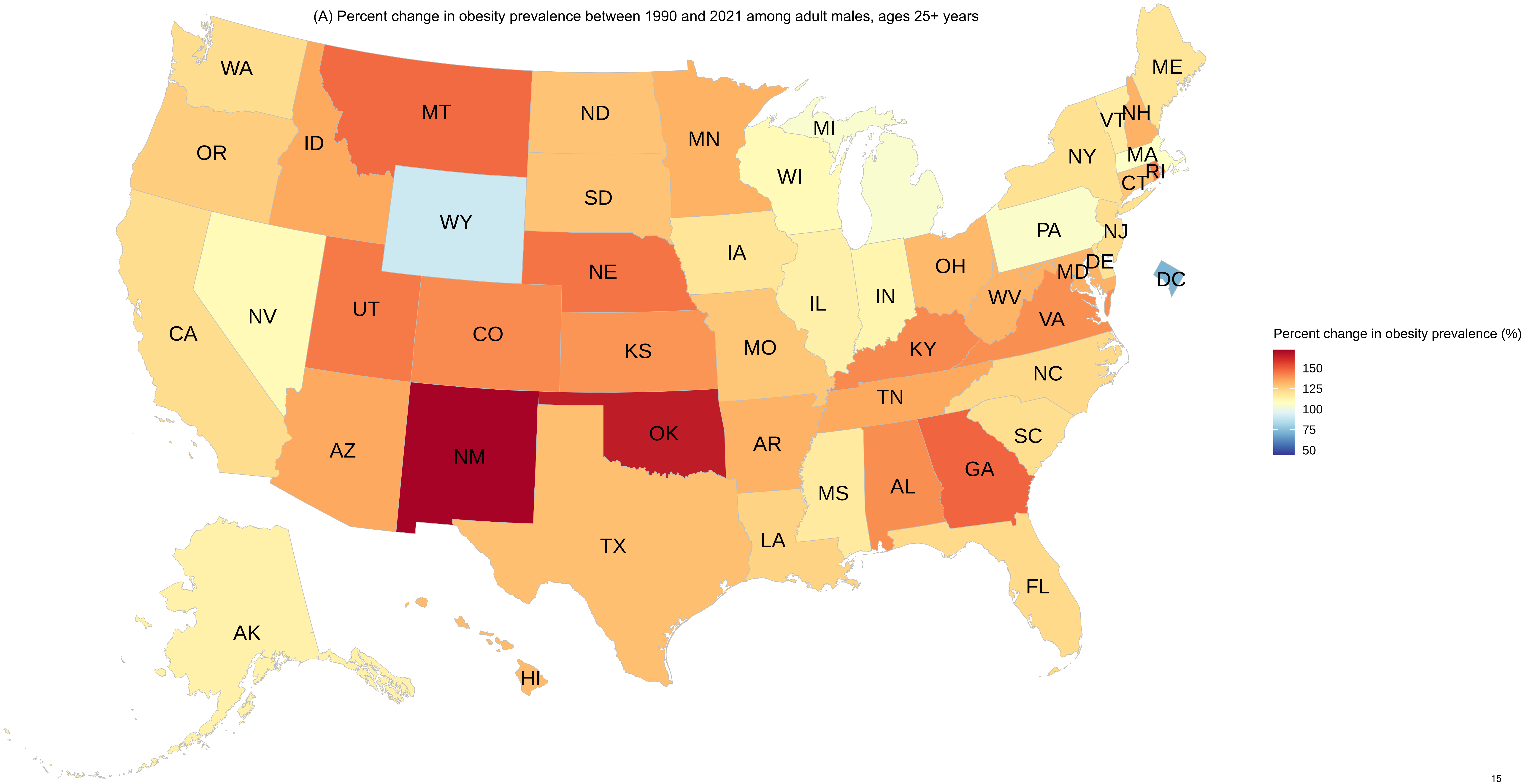

SM Figure 6: Changes in obesity prevalence from 1990 to 2021 and from 2021 to 2050 in the USA for (A) adult males, (B) adult females, (C) both sexes, ages 25+ years

(A) Percent change in obesity prevalence between 2021 and 2050 among adult males, ages 25+ years

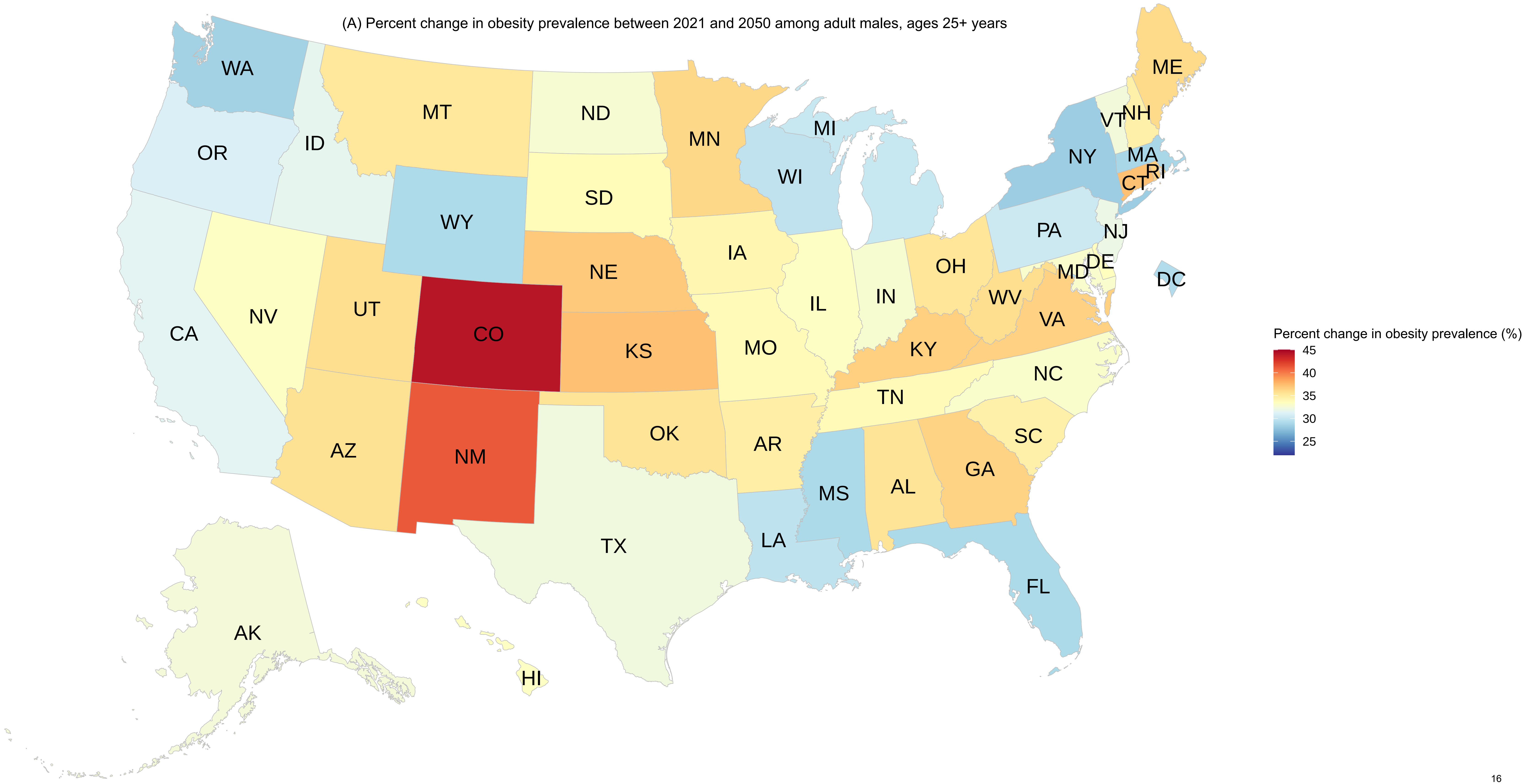

SM Figure 6: Changes in obesity prevalence from 1990 to 2021 and from 2021 to 2050 in the USA for (A) adult males, (B) adult females, (C) both sexes, ages 25+ years

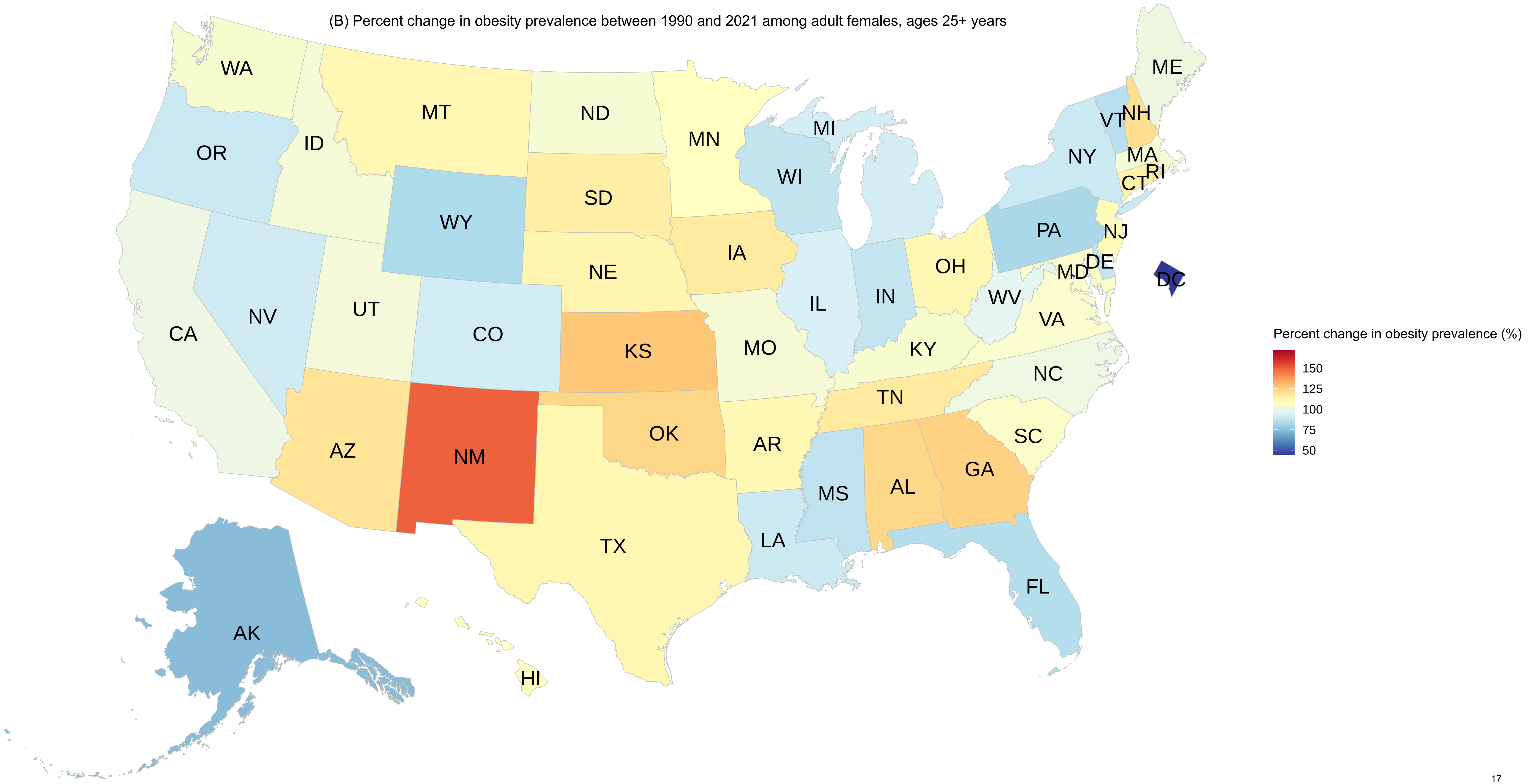

SM Figure 6: Changes in obesity prevalence from 1990 to 2021 and from 2021 to 2050 in the USA for (A) adult males, (B) adult females, (C) both sexes, ages 25+ years

(B) Percent change in obesity prevalence between 2021 and 2050 among adult females, ages 25+ years

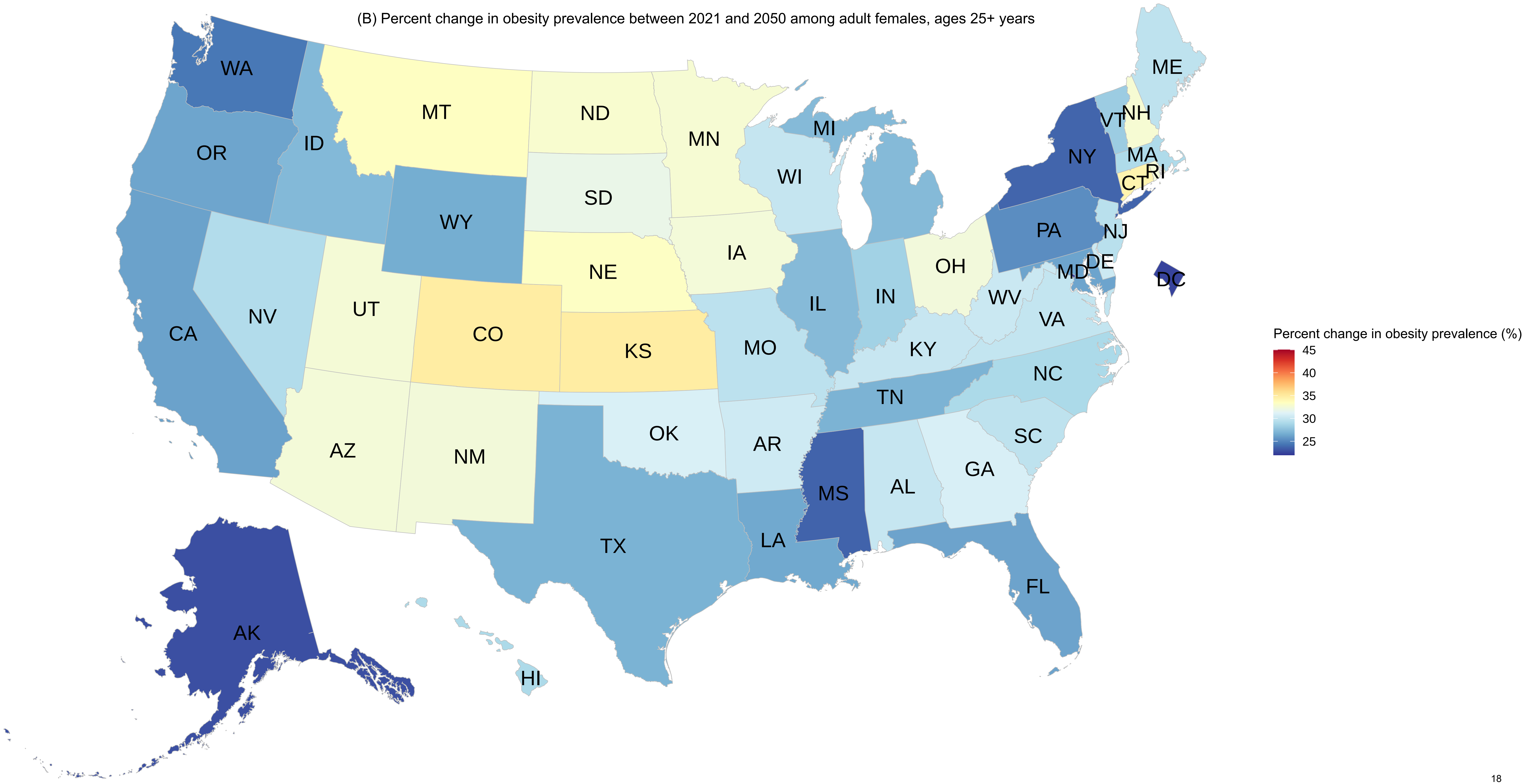

SM Figure 6: Changes in obesity prevalence from 1990 to 2021 and from 2021 to 2050 in the USA for (A) adult males, (B) adult females, (C) both sexes, ages 25+ years

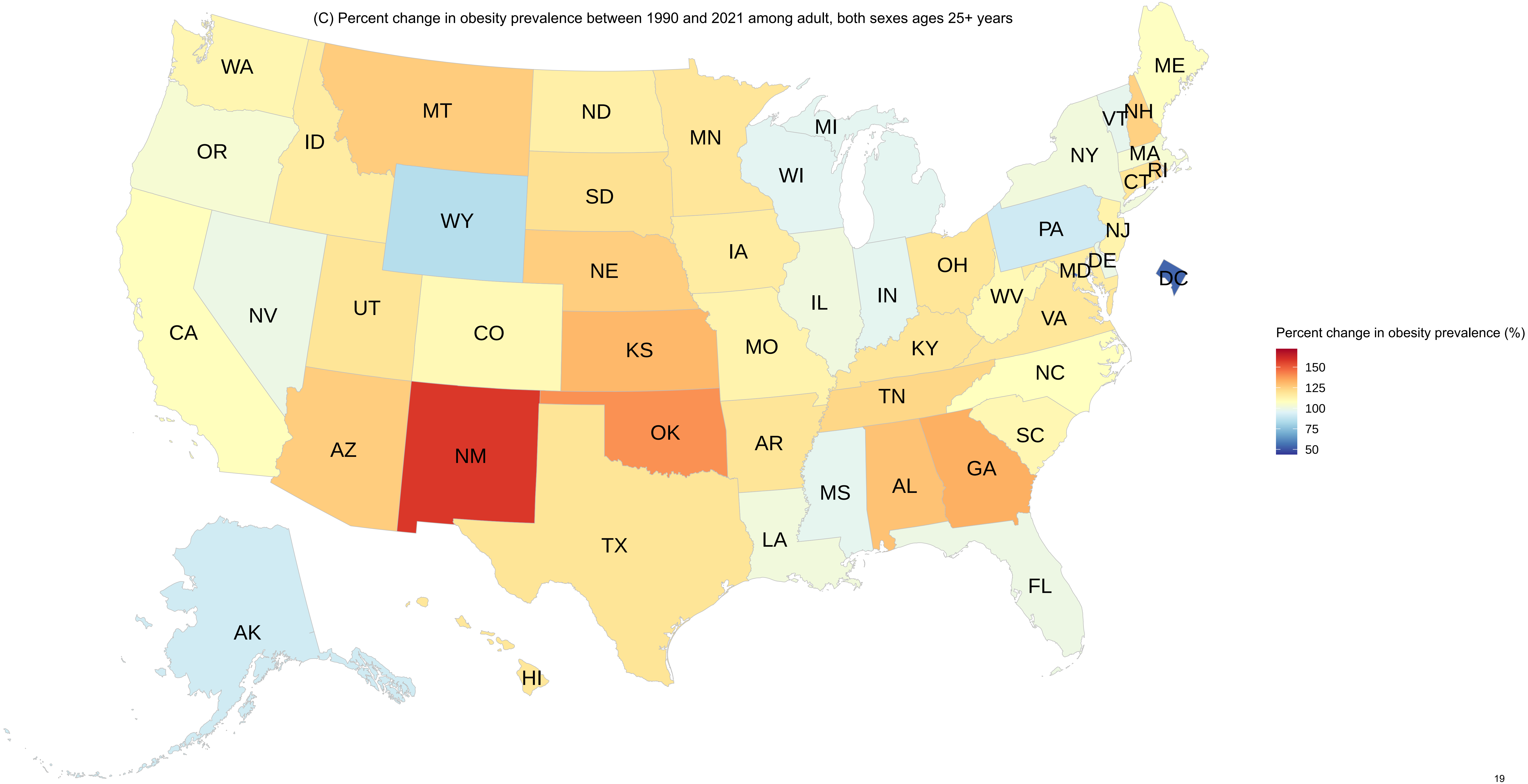

SM Figure 6: Changes in obesity prevalence from 1990 to 2021 and from 2021 to 2050 in the USA for (A) adult males, (B) adult females, (C) both sexes, ages 25+ years

(C) Percent change in obesity prevalence between 2021 and 2050 among adult, both sexes, ages 25+ years

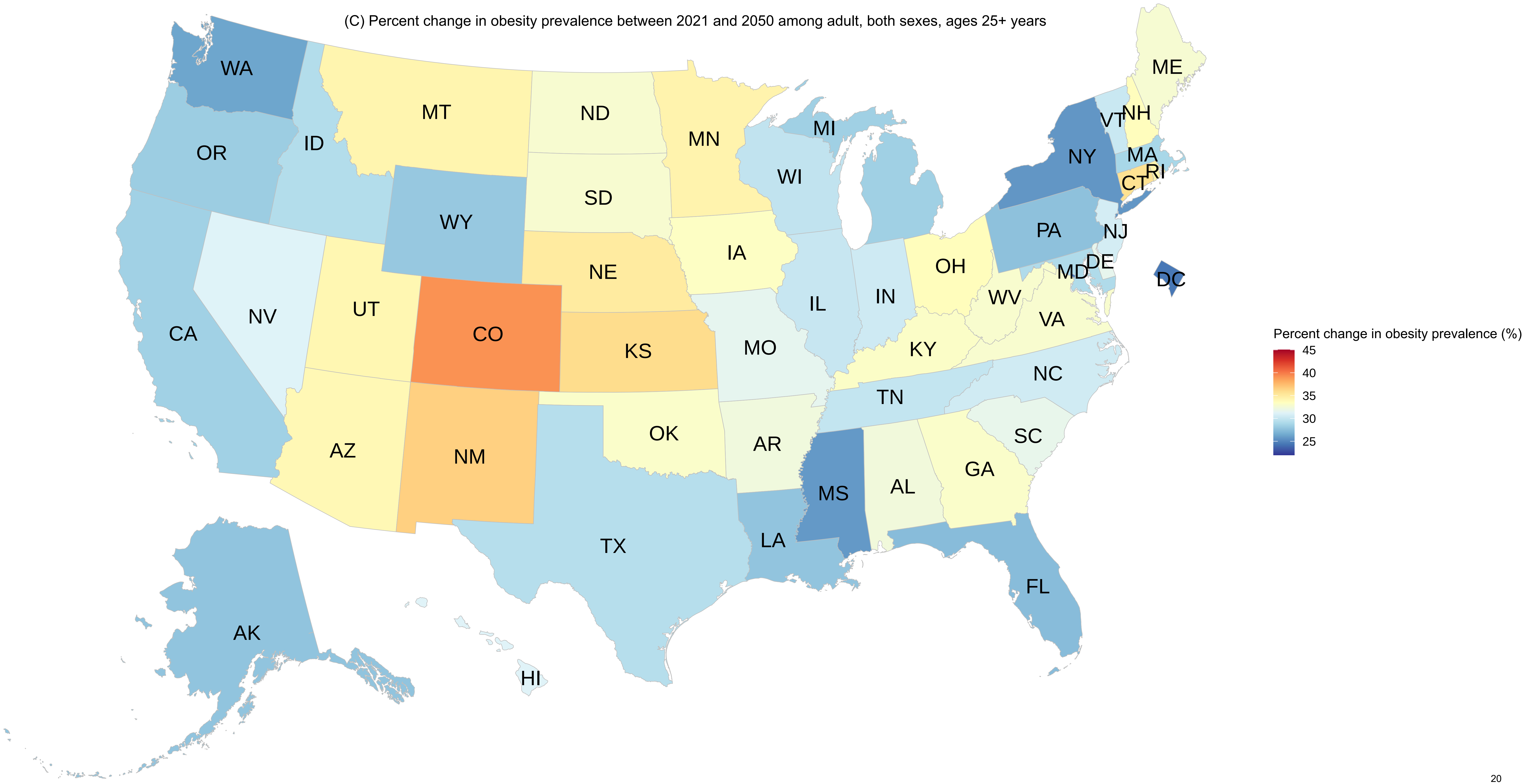

SM Figure 7: Distribution of adolescents and adults with overweight and obesity across 50 states and Washington DC in 1990, 2021 and 2025

Number of adolescents aged 15 – 24 with overweight and obesity, both sexes

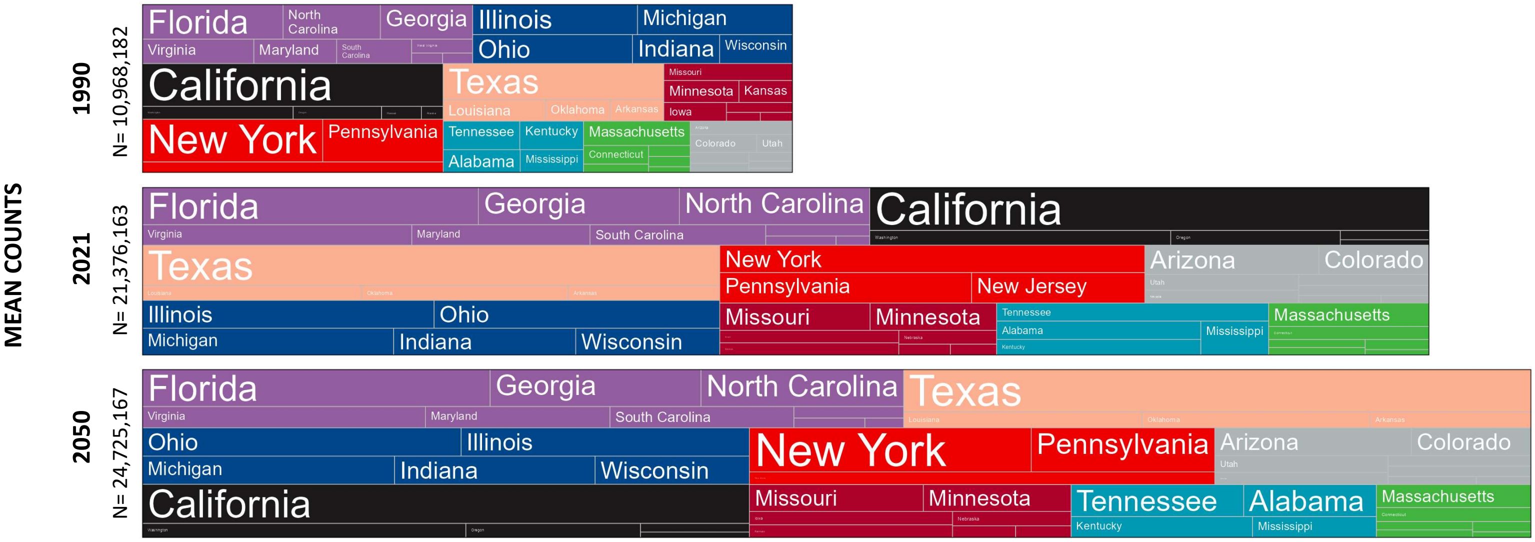

Number of adults 25+ with overweight and obesity, both sexes

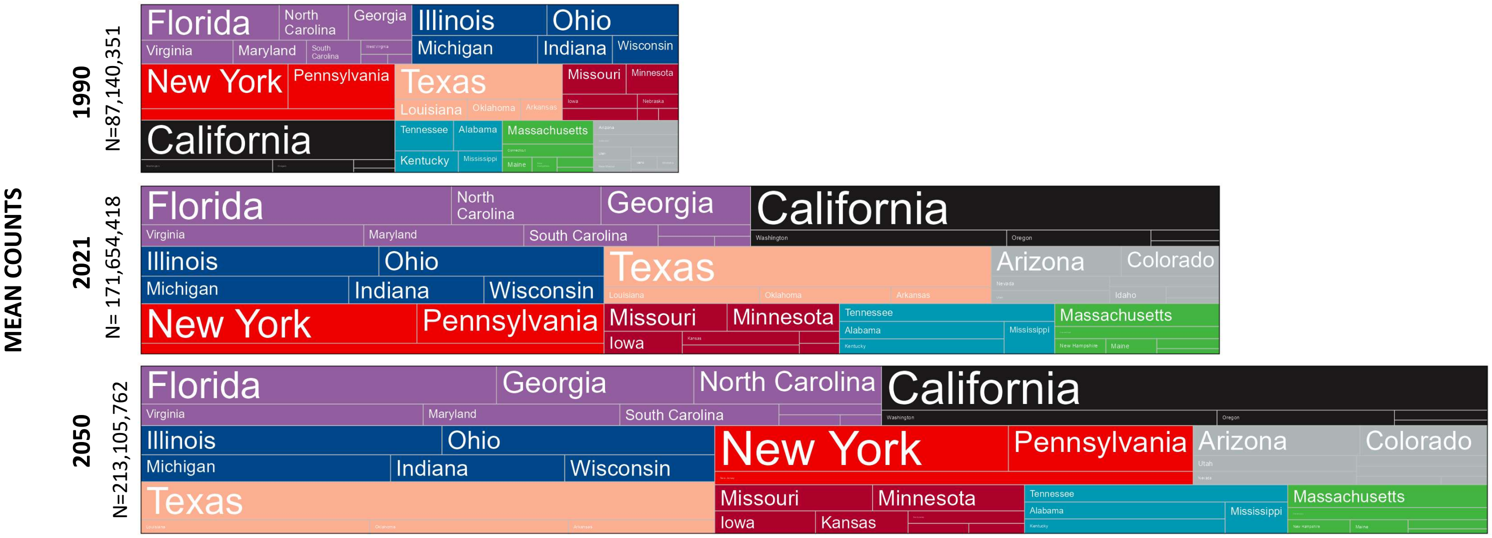

SM Figure 8: Distribution of adolescents and adults with obesity across 50 states and Washington DC in 1990, 2021 and 2025

Number of adolescents aged 15 – 24 with obesity, both sexes

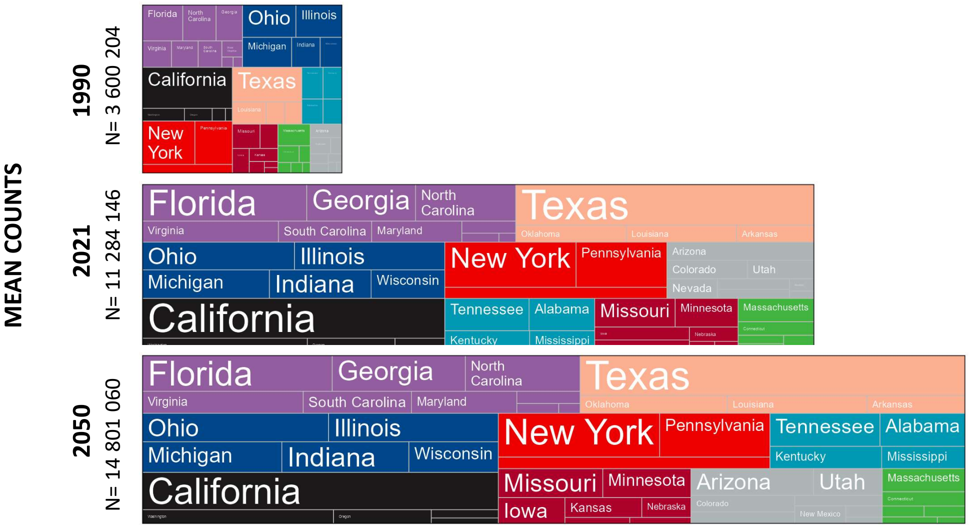

Number of adults 25+ with obesity, both sexes

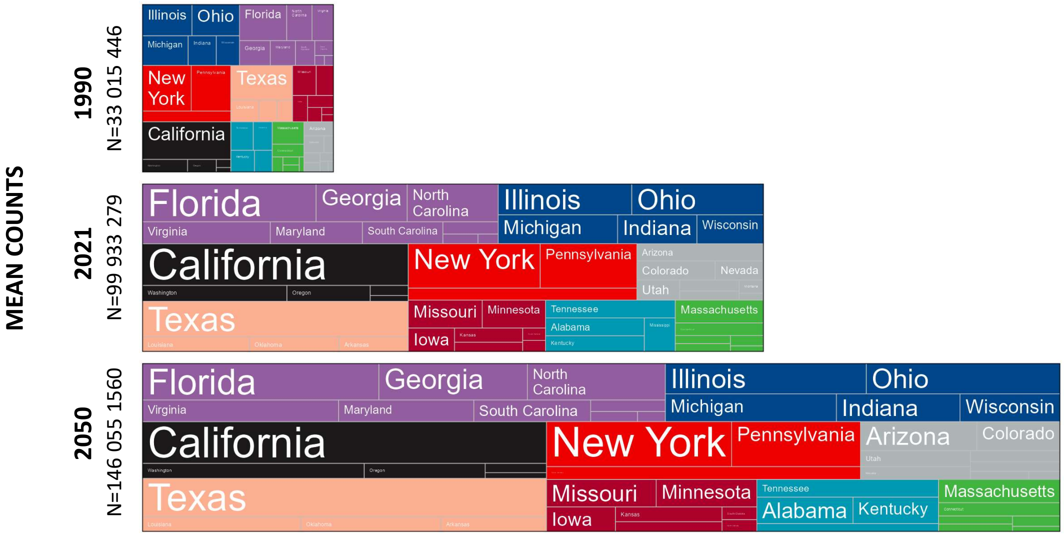

SM Figure 9: Prevalence of overweight and obesity by age across birth cohorts for (A) males and (B) females in USA. Prevalence for each 5-year birth cohort was deduced from the estimated and projected prevalence of the corresponding 5-year age group for each 5-year period from 1990 to 2050.

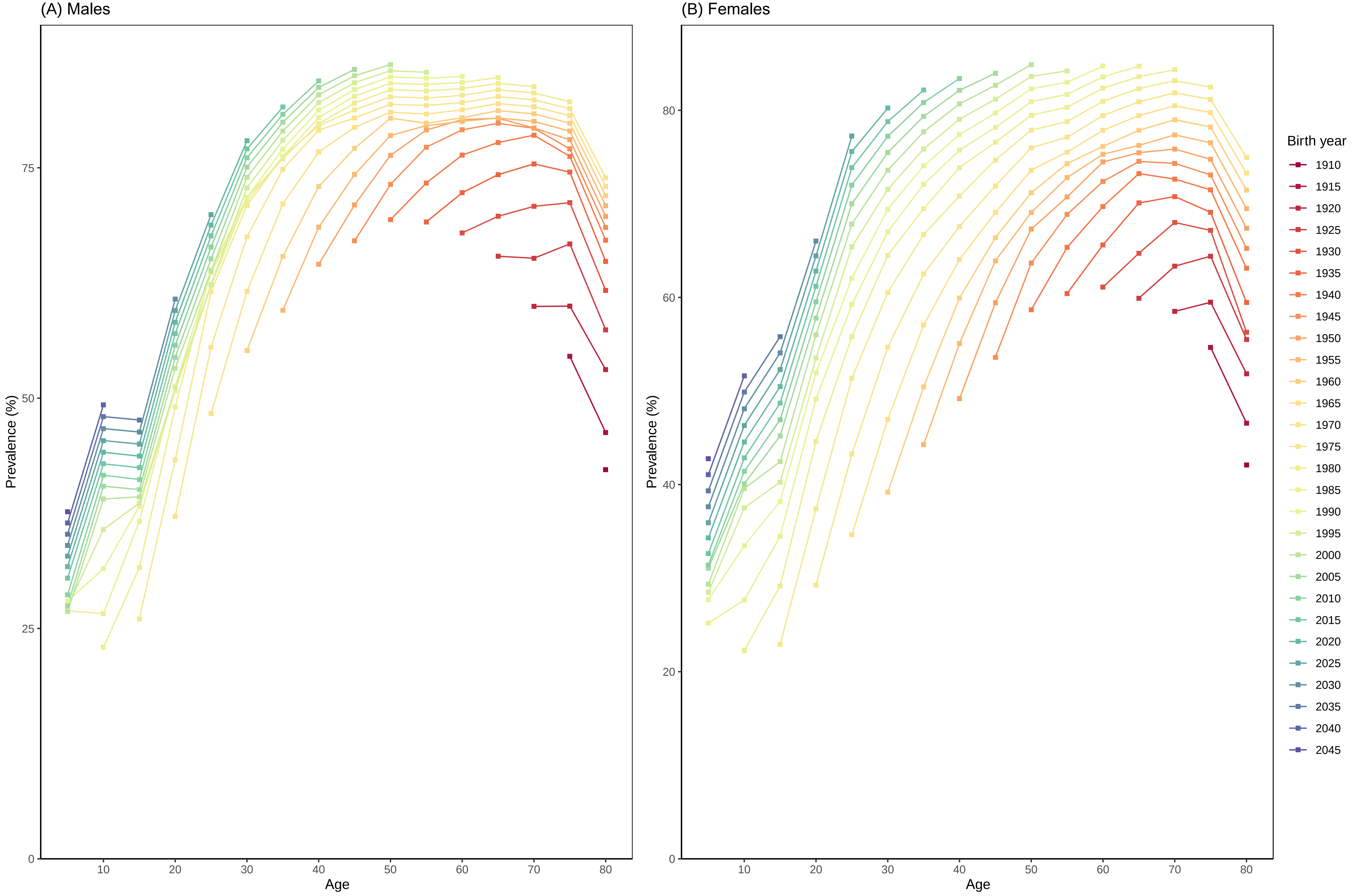

SM Figure 10: Estimated age-standardised prevalence of overweight and obesity among (A) adolescents ages 15-24 and (B) adults ages 25+ with the associated relative percentage change at the national level, across 50 states and Washington DC

(A) Adolescents 15 to 24 years

	Female					Male				
	1990	2021	2050	1990-2021 Percent Change	2021-2050 Percent Change	1990	2021	2050	1990-2021 Percent Change	2021-2050 Percent Change
USA	26.0% (23.9-28.2)	50.8% (46.7-54.9)	60.8% (52.1-67.0)	95.9% (74.52-119.6)	19.9% (7.64-29.5)	31.4% (29.8-33.2)	46.7% (43.3-50.2)	54.0% (46.8-59.3)	48.6% (35.7-63.0)	16.0% (5.5-22.8)
Alabama	24.1% (20.5-28.6)	59.4% (53.5-65.3)	71.8% (62.3-79.2)	148.1% (101.23-203.3)	21.1% (9.66-32.0)	31.1% (27.3-35.3)	51.3% (45.3-57.3)	60.5% (51.3-68.7)	65.7% (39.7-95.9)	18.2% (6.0-30.9)
Alaska	33.0% (26.6-39.8)	54.8% (47.7-62.3)	63.6% (51.4-72.0)	67.9% (32.49-111.3)	16.5% (3.44-25.8)	36.6% (30.8-42.5)	49.3% (43.1-55.6)	56.7% (46.9-64.5)	35.5% (10.9-65.1)	15.4% (2.5-22.7)
Arizona	20.7% (16.4-25.3)	52.4% (45.8-59.2)	66.7% (53.9-76.6)	156.5% (97.18-225.8)	27.3% (9.14-41.9)	28.2% (24.5-32.4)	46.4% (41.3-52.0)	54.9% (44.9-62.5)	65.4% (37.8-95.9)	18.8% (4.7-30.5)
Arkansas	30.2% (24.5-36.8)	58.9% (52.0-65.7)	68.7% (59.5-78.2)	97.1% (54.57-150.5)	16.8% (7.61-30.6)	34.8% (29.2-41.0)	49.5% (43.0-56.3)	55.6% (47.7-64.0)	43.2% (15.6-76.4)	12.8% (4.8-23.1)
California	24.2% (21.1-27.6)	45.2% (39.4-50.6)	51.8% (43.2-60.9)	87.7% (54.75-125.6)	15.0% (1.93-33.4)	29.5% (25.5-33.6)	44.8% (38.9-50.8)	50.9% (41.8-59.3)	52.3% (23.9-83.0)	14.2% (1.7-28.3)
Colorado	22.4% (17.8-27.8)	42.3% (36.0-48.5)	50.7% (39.5-59.4)	91.5% (46.03-149.3)	20.3% (3.18-32.5)	27.1% (23.1-31.6)	39.0% (34.4-44.3)	45.5% (36.6-52.8)	44.7% (15.5-74.3)	16.9% (3.4-23.4)
Connecticut	20.7% (16.0-26.3)	47.2% (40.3-54.4)	57.9% (45.9-68.5)	131.2% (70.32-211.4)	23.4% (5.67-39.5)	29.6% (25.5-34.0)	44.1% (38.9-49.7)	50.3% (42.6-57.9)	50.0% (23.7-79.9)	14.6% (4.2-25.8)
Delaware	28.4% (23.4-34.3)	56.4% (49.7-63.2)	69.3% (57.1-77.7)	100.4% (57.57-149.1)	23.1% (8.31-36.3)	32.8% (27.3-38.8)	42.9% (36.5-49.9)	47.4% (39.4-55.4)	31.8% (3.6-66.3)	11.3% (3.4-19.7)
Florida	25.9% (20.7-32.4)	49.7% (42.1-57.5)	59.0% (47.4-69.3)	94.4% (46.10-154.4)	19.8% (6.49-34.6)	30.8% (27.2-34.6)	44.6% (39.5-49.8)	50.9% (42.0-57.8)	45.7% (22.8-72.0)	14.3% (3.9-22.7)
Georgia	28.3% (24.2-33.0)	52.2% (46.3-58.4)	61.8% (51.9-71.4)	85.6% (53.17-124.8)	18.6% (6.17-31.9)	32.5% (28.2-36.8)	45.1% (40.1-50.7)	50.6% (42.6-57.8)	39.3% (15.9-64.0)	12.6% (3.4-23.6)
Hawaii	24.4% (20.1-29.2)	46.7% (40.2-53.3)	56.1% (45.8-65.8)	92.7% (51.25-141.5)	20.5% (4.94-35.6)	35.1% (30.6-39.9)	47.3% (41.5-53.3)	54.4% (45.1-61.9)	35.4% (11.9-63.2)	15.3% (3.0-23.1)
Idaho	24.6% (20.5-29.1)	51.0% (44.7-57.3)	62.1% (51.4-71.5)	108.8% (68.29-157.2)	22.5% (8.20-38.4)	27.3% (23.6-31.2)	42.7% (37.4-48.6)	49.4% (41.1-57.4)	57.4% (29.8-89.8)	16.2% (5.7-27.1)
Illinois	28.1% (24.1-32.6)	50.3% (44.3-56.6)	58.0% (48.4-66.1)	80.2% (46.91-116.6)	15.9% (4.55-28.4)	29.7% (26.2-33.7)	45.5% (40.1-50.7)	52.8% (43.5-60.4)	53.9% (29.7-81.2)	16.4% (4.6-28.3)
Indiana	26.0% (22.2-30.1)	54.6% (48.8-60.9)	65.6% (55.7-75.1)	111.3% (72.62-156.5)	20.5% (8.15-36.7)	31.6% (27.3-36.7)	48.7% (42.2-55.2)	57.7% (48.7-65.5)	55.1% (25.1-87.5)	19.0% (8.0-27.5)
Iowa	25.4% (21.2-30.0)	55.7% (49.9-61.6)	69.5% (58.3-76.7)	121.3% (81.40-170.6)	25.0% (10.35-35.5)	31.4% (27.2-35.8)	47.7% (42.5-52.9)	55.8% (46.8-62.6)	52.5% (26.9-81.1)	17.4% (6.7-25.8)
Kansas	29.7% (23.9-36.2)	56.7% (50.5-62.5)	68.6% (57.4-75.5)	92.6% (52.47-144.4)	21.3% (6.89-31.4)	34.7% (29.2-40.5)	51.0% (46.4-55.9)	60.4% (51.0-66.7)	48.0% (22.3-78.4)	19.0% (6.5-25.6)
Kentucky	30.8% (26.5-35.7)	58.2% (52.1-63.9)	70.2% (60.1-77.3)	89.8% (57.32-128.9)	20.9% (8.90-31.2)	31.9% (28.3-36.2)	50.6% (45.1-56.3)	58.9% (50.1-66.7)	59.0% (32.8-87.0)	16.8% (6.4-28.5)
Louisiana	32.7% (27.4-39.0)	57.4% (50.8-64.0)	66.6% (58.0-74.0)	76.8% (43.44-115.6)	16.1% (7.43-24.3)	32.0% (27.4-37.1)	47.5% (42.2-53.0)	54.0% (46.2-61.9)	49.0% (22.6-77.0)	14.4% (6.1-25.2)
Maine	28.8% (23.0-35.3)	50.8% (43.2-58.5)	60.4% (49.7-69.8)	78.2% (34.69-129.0)	19.0% (7.39-31.2)	33.7% (28.7-38.5)	44.2% (38.3-50.3)	49.4% (41.6-57.5)	31.9% (8.0-60.6)	12.4% (4.8-21.8)
Maryland	24.5% (20.2-29.2)	49.9% (43.8-55.8)	57.7% (48.0-67.5)	105.0% (63.55-156.5)	16.1% (4.04-30.6)	32.8% (28.4-37.2)	43.7% (38.8-49.2)	49.2% (41.3-55.8)	33.9% (12.6-59.5)	12.7% (3.0-18.4)
Massachusetts	21.5% (17.4-26.0)	42.4% (36.6-48.1)	51.9% (40.8-62.1)	99.7% (57.48-151.5)	22.9% (6.76-41.5)	33.0% (28.9-37.1)	41.5% (36.8-46.8)	44.2% (38.5-51.1)	26.3% (5.0-51.8)	7.0% (1.8-16.4)
Michigan	29.5% (25.2-34.1)	53.7% (47.7-59.5)	63.9% (54.6-71.9)	83.1% (51.24-117.5)	19.3% (8.18-30.8)	35.9% (31.9-40.2)	48.1% (42.9-52.9)	55.6% (46.6-63.3)	34.4% (14.9-55.7)	15.9% (4.5-26.9)
Minnesota	20.6% (17.4-24.3)	47.8% (42.4-53.3)	57.5% (47.6-68.2)	133.1% (90.06-185.3)	20.5% (5.10-40.0)	31.5% (27.7-35.7)	45.6% (40.1-51.4)	52.2% (42.6-59.7)	45.4% (21.1-72.0)	15.4% (3.0-24.0)
Mississippi	33.9% (28.4-40.0)	63.0% (57.0-68.5)	74.1% (64.3-80.9)	86.9% (54.04-124.5)	18.2% (7.26-27.7)	33.9% (29.0-39.0)	50.5% (45.0-56.5)	56.3% (47.9-65.0)	49.9% (23.4-81.9)	11.9% (2.4-24.6)
Missouri	27.9% (22.8-33.3)	54.4% (47.8-60.7)	64.5% (54.4-72.5)	96.7% (56.91-145.2)	18.9% (6.51-29.3)	31.1% (27.1-35.2)	47.3% (42.6-52.6)	55.5% (46.8-62.2)	52.7% (28.6-79.5)	17.7% (6.6-25.8)
Montana	25.6% (20.6-30.9)	50.0% (43.9-56.4)	61.6% (49.4-70.2)	97.3% (54.78-144.3)	23.0% (6.97-36.0)	32.6% (27.8-38.1)	45.1% (39.8-50.8)	51.6% (42.7-58.6)	39.0% (12.7-67.7)	15.0% (4.2-22.5)
Nebraska	22.7% (18.2-27.5)	51.7% (46.2-57.2)	64.5% (52.9-72.7)	129.9% (82.98-188.3)	25.0% (8.04-38.9)	34.8% (30.6-39.5)	48.7% (44.1-53.8)	56.3% (48.1-62.6)	40.5% (19.5-64.6)	16.0% (5.4-21.9)
Nevada	29.1% (23.2-35.8)	47.7% (41.0-54.4)	56.7% (45.9-65.2)	65.7% (27.08-112.8)	19.1% (5.76-29.8)	35.6% (30.1-41.9)	46.4% (40.3-52.6)	52.5% (43.6-59.8)	31.3% (5.0-60.4)	13.6% (3.7-21.2)
New Hampshire	25.2% (20.5-30.4)	45.8% (39.2-52.5)	55.6% (43.3-64.5)	83.5% (44.85-137.9)	21.6% (4.90-33.3)	29.9% (25.4-34.6)	47.7% (41.5-53.8)	56.4% (45.0-64.9)	60.4% (31.0-94.3)	18.4% (4.0-28.5)
New Jersey	26.3% (21.2-31.6)	45.0% (38.9-51.2)	53.6% (43.8-62.0)	73.0% (34.63-119.4)	19.2% (5.85-30.7)	31.4% (26.7-36.1)	45.2% (40.0-50.8)	53.2% (43.5-61.0)	45.0% (19.1-76.4)	18.3% (4.7-28.0)
New Mexico	27.1% (22.4-32.7)	50.4% (43.6-56.7)	61.3% (50.5-69.2)	87.7% (48.55-134.7)	22.3% (7.47-33.1)	29.7% (25.8-34.3)	51.0% (45.5-56.7)	61.1% (51.4-68.1)	72.7% (43.1-106.2)	20.3% (6.9-29.5)
New York	24.0% (19.7-28.7)	47.7% (41.2-54.2)	57.6% (46.4-67.8)	101.0% (59.35-153.9)	20.9% (5.45-38.3)	31.9% (28.0-35.8)	46.4% (41.7-51.1)	54.3% (44.9-61.5)	45.9% (24.3-71.5)	17.4% (5.2-26.0)
North Carolina	29.5% (25.2-33.6)	51.2% (45.2-57.4)	58.4% (49.4-67.9)	74.8% (43.99-109.9)	14.2% (2.63-30.2)	32.8% (29.1-36.9)	47.2% (42.1-52.6)	53.8% (45.4-61.5)	44.4% (21.4-69.2)	14.2% (3.5-23.2)
North Dakota	23.7% (19.3-28.6)	52.4% (45.9-59.1)	67.2% (52.3-76.4)	123.9% (78.09-184.5)	28.2% (8.22-41.9)	33.1% (28.6-37.6)	50.0% (44.1-56.3)	57.1% (47.7-65.8)	52.0% (26.6-85.0)	14.8% (2.8-28.5)
Ohio	27.0% (22.7-32.0)	55.1% (49.3-61.0)	66.9% (57.6-74.0)	105.2% (66.78-150.4)	21.7% (9.66-31.8)	29.5% (25.9-33.3)	47.5% (42.6-52.4)	55.7% (47.5-62.8)	61.4% (35.8-91.3)	17.9% (7.7-28.0)
Oklahoma	25.8% (21.2-30.9)	59.0% (52.9-65.0)	71.8% (61.2-80.5)	130.8% (86.96-182.5)	22.2% (9.00-35.5)	32.8% (28.3-37.9)	51.4% (45.6-56.9)	60.8% (51.4-68.3)	57.4% (30.7-89.3)	18.3% (6.1-28.8)
Oregon	28.7% (24.2-33.7)	49.1% (42.8-55.2)	57.4% (47.0-65.8)	72.2% (40.46-112.7)	17.2% (4.03-27.6)	29.6% (24.9-35.3)	44.7% (38.4-51.1)	54.1% (43.6-62.4)	52.0% (21.0-86.9)	21.3% (5.8-30.9)
Pennsylvania	26.5% (22.2-31.0)	48.1% (42.4-54.0)	56.4% (47.3-65.0)	83.0% (49.63-124.6)	17.5% (6.00-32.4)	32.7% (28.6-36.8)	44.9% (39.7-49.8)	50.8% (43.7-57.3)	37.9% (16.5-63.4)	13.5% (5.1-20.3)
Rhode Island	20.6% (16.7-25.0)	45.1% (38.2-51.6)	53.8% (43.0-65.6)	121.4% (69.61-180.0)	19.3% (3.42-42.1)	31.6% (27.6-36.0)	44.6% (39.0-50.5)	50.5% (42.2-58.5)	41.6% (17.6-71.8)	13.5% (3.6-21.5)
South Carolina	28.1% (23.9-32.9)	55.7% (49.1-62.5)	65.4% (55.8-74.8)	99.3% (62.89-143.0)	18.1% (6.79-32.0)	34.8% (30.4-39.6)	49.4% (43.0-55.4)	57.0% (48.3-64.6)	42.8% (17.7-71.0)	15.7% (6.4-22.8)
South Dakota	25.7% (20.4-31.7)	52.9% (45.6-59.8)	64.0% (51.6-74.4)	108.1% (60.44-164.3)	21.6% (6.93-36.9)	32.5% (28.1-37.1)	45.1% (40.3-50.5)	50.3% (41.5-59.4)	39.4% (17.0-65.0)	12.1% (0.8-27.0)
Tennessee	27.1% (23.1-31.7)	57.9% (51.6-64.3)	69.0% (58.3-78.4)	114.6% (75.70-160.3)	19.4% (6.95-33.6)	30.5% (26.8-34.1)	48.2% (42.9-53.8)	54.9% (47.4-63.6)	58.8% (35.0-87.3)	14.2% (4.8-28.3)
Texas	26.8% (22.7-31.0)	54.8% (49.1-60.3)	66.2% (55.2-74.5)	105.9% (71.26-147.8)	21.3% (5.97-33.4)	31.6% (27.7-35.5)	52.4% (47.4-57.6)	61.9% (52.3-68.8)	66.3% (41.9-93.8)	18.3% (5.1-27.1)
Utah	19.5% (16.0-23.4)	44.1% (38.4-50.2)	56.4% (44.8-65.0)	127.6% (80.74-183.7)	27.8% (9.11-42.7)	23.7% (19.9-27.7)	40.7% (35.4-46.5)	47.7% (39.3-55.7)	73.1% (37.3-114.6)	18.0% (4.9-33.4)
Vermont	26.9% (21.1-32.8)	49.1% (42.7-56.3)	60.6% (48.2-69.5)	84.6% (41.77-140.8)	24.0% (7.08-35.5)	30.2% (25.1-35.3)	43.8% (37.8-50.5)	49.9% (40.8-58.7)	46.4% (16.2-82.6)	14.2% (3.7-24.5)
Virginia	22.9% (18.4-27.9)	50.7% (44.8-56.9)	61.0% (49.8-71.4)	124.5% (73.49-179.7)	20.5% (6.13-37.8)	31.1% (26.3-36.5)	46.3% (40.3-52.5)	54.6% (44.6-62.6)	49.7% (22.0-82.6)	18.5% (5.3-28.2)
Washington	25.1% (20.9-29.9)	46.2% (40.8-51.8)	52.8% (43.6-63.9)	85.9% (50.31-129.5)	14.8% (2.08-36.4)	30.3% (26.3-34.6)	43.8% (38.9-49.0)	48.7% (41.4-56.3)	45.2% (21.6-73.5)	11.5% (2.6-23.7)
Washington, D.C.	28.6% (22.8-34.7)	47.0% (38.3-55.5)	53.2% (40.4-65.2)	66.7% (23.50-116.2)	13.1% (1.97-33.4)	30.2% (25.6-34.9)	38.1% (31.6-45.3)	42.6% (33.4-51.3)	26.7% (-1.1-60.1)	11.7% (1.9-20.9)
West Virginia	28.7% (24.6-33.1)	55.6% (49.2-61.8)	66.4% (57.3-74.9)	94.9% (61.13-132.6)	19.7% (9.44-30.1)	34.9% (31.0-38.8)	52.2% (46.9-57.9)	61.0% (53.7-68.3)	50.2% (27.9-75.8)	17.3% (8.9-24.9)
Wisconsin	24.2% (20.0-29.0)	54.6% (48.2-60.9)	68.3% (56.7-76.8)	127.5% (81.08-179.7)	25.4% (10.54-39.7)	33.2% (29.3-37.5)	46.7% (41.3-52.3)	53.1% (45.0-60.6)	41.2% (17.6-68.0)	14.0% (5.2-24.0)
Wyoming	29.0% (22.8-36.0)	50.5% (43.5-58.1)	59.9% (46.8-68.7)	76.5% (33.56-129.2)	19.0% (4.53-28.4)	31.4% (25.3-37.3)	48.8% (42.5-55.3)	58.6% (46.8-67.1)	56.7% (24.7-100.8)	20.7% (5.0-28.5)

Prevalence (%): 15.0% – 75.0%

Percent Change (%): 5.0% – 160.0%

SM Figure 10: Estimated age-standardised prevalence of overweight and obesity among (A) adolescents ages 15-24 and (B) adults ages 25+ with the associated relative percentage change at the national level, across 50 states and Washington DC

(B) Adults 25 years +

	Female					Male				
	1990	2021	2050	1990-2021 Percent Change	2021-2050 Percent Change	1990	2021	2050	1990-2021 Percent Change	2021-2050 Percent Change
USA	49.1% (48.0-50.1)	72.6% (70.8-74.3)	82.1% (76.7-85.7)	47.9% (43.2-52.4)	13.1% (6.5-17.9)	60.5% (59.6-61.3)	75.9% (74.6-77.2)	81.1% (77.9-84.5)	25.6% (22.7-28.6)	6.9% (3.3-11.1)
Alabama	49.7% (47.9-51.4)	78.1% (76.0-80.2)	88.3% (83.6-91.6)	57.1% (50.6-64.2)	13.1% (7.3-17.5)	61.5% (59.7-63.3)	77.8% (75.8-79.7)	83.2% (79.6-88.3)	26.5% (21.4-31.5)	7.0% (2.9-13.3)
Alaska	53.9% (51.2-56.8)	72.1% (69.4-74.7)	78.9% (72.8-84.6)	33.8% (25.3-42.2)	9.5% (3.1-17.6)	62.1% (59.8-64.4)	75.7% (73.4-77.8)	80.4% (75.7-86.3)	22.0% (16.5-27.6)	6.2% (1.3-13.9)
Arizona	45.0% (43.0-47.1)	71.9% (69.0-74.4)	83.3% (75.3-88.6)	59.9% (50.8-69.0)	16.0% (6.4-23.8)	55.2% (53.6-56.9)	76.4% (74.5-78.3)	83.0% (77.8-88.4)	38.5% (33.0-44.5)	8.5% (2.8-15.7)
Arkansas	51.6% (49.1-54.3)	77.2% (74.9-79.5)	87.2% (81.8-91.1)	49.6% (41.3-58.6)	12.8% (7.1-18.6)	61.8% (59.0-64.5)	77.1% (74.7-79.4)	82.1% (77.8-87.6)	24.8% (18.0-31.8)	6.4% (2.4-13.9)
California	46.3% (44.8-47.8)	69.7% (67.4-72.1)	78.3% (70.7-84.6)	50.6% (44.4-57.7)	12.2% (3.3-21.3)	58.5% (56.5-60.4)	73.3% (71.1-75.6)	77.8% (72.9-84.4)	25.3% (19.6-31.4)	6.2% (1.1-15.2)
Colorado	43.5% (41.4-45.9)	65.2% (62.7-67.8)	76.2% (66.5-81.9)	49.9% (40.0-59.8)	16.7% (4.2-25.2)	54.2% (52.2-56.1)	70.6% (68.4-72.5)	76.3% (70.6-83.0)	30.2% (24.4-36.3)	8.1% (1.4-17.5)
Connecticut	44.2% (41.7-46.8)	69.6% (66.9-72.3)	80.5% (72.8-86.7)	57.7% (47.4-68.7)	15.5% (6.7-24.8)	59.9% (58.0-61.6)	76.2% (74.3-78.2)	81.7% (77.0-87.3)	27.4% (22.3-32.5)	7.1% (2.4-14.2)
Delaware	52.6% (50.4-55.1)	74.2% (71.8-76.5)	83.6% (77.8-88.2)	41.1% (33.7-48.9)	12.6% (5.7-18.7)	62.5% (60.0-65.0)	76.6% (74.2-78.9)	81.7% (77.5-87.1)	22.7% (16.4-29.0)	6.6% (2.5-13.3)
Florida	47.8% (45.1-50.7)	69.4% (66.2-72.4)	78.2% (71.5-84.5)	45.2% (35.0-55.9)	12.7% (5.1-22.1)	59.5% (57.8-61.1)	74.8% (72.7-76.8)	79.4% (75.0-86.1)	25.7% (20.9-30.7)	6.2% (1.5-15.3)
Georgia	48.5% (46.7-50.3)	75.1% (72.8-77.1)	85.0% (78.6-89.4)	55.0% (48.1-61.9)	13.2% (5.5-19.3)	60.4% (58.7-62.2)	75.7% (73.6-77.7)	80.8% (76.3-86.5)	25.2% (20.4-30.3)	6.9% (2.1-14.1)
Hawaii	40.1% (38.3-42.0)	63.7% (61.2-66.4)	73.9% (66.2-81.7)	58.8% (49.2-69.3)	15.8% (6.0-27.6)	52.9% (50.9-54.8)	72.9% (70.4-75.4)	79.5% (74.0-85.9)	37.9% (31.3-45.7)	9.0% (2.8-18.4)
Idaho	48.1% (46.3-50.0)	70.9% (68.5-73.4)	80.2% (74.3-85.9)	47.4% (39.9-55.2)	13.3% (6.1-21.4)	59.8% (58.2-61.4)	77.3% (75.4-79.3)	83.0% (78.8-88.4)	29.4% (24.7-33.9)	7.3% (3.1-14.0)
Illinois	49.7% (48.0-51.4)	73.4% (70.9-75.6)	82.5% (75.6-88.0)	47.7% (41.0-54.4)	12.4% (4.7-19.8)	61.4% (59.9-62.9)	76.3% (74.4-78.2)	81.1% (76.8-86.7)	24.4% (19.7-28.9)	6.4% (1.8-13.9)
Indiana	53.0% (51.4-54.7)	75.7% (73.6-77.8)	85.4% (80.1-89.4)	42.8% (36.7-49.0)	12.7% (6.7-18.2)	63.0% (60.9-65.2)	77.1% (74.7-79.3)	82.4% (78.1-87.0)	22.3% (16.7-28.1)	6.8% (2.5-12.9)
Iowa	51.3% (49.4-53.3)	76.0% (73.7-78.1)	86.6% (80.3-90.2)	48.0% (40.2-54.9)	13.9% (6.6-18.8)	63.9% (62.2-65.6)	80.1% (78.4-81.8)	86.3% (82.1-90.2)	25.5% (21.2-29.8)	7.7% (3.0-12.3)
Kansas	48.6% (46.2-51.2)	75.3% (73.1-77.5)	86.6% (79.1-90.3)	54.9% (45.7-64.0)	15.0% (6.0-20.4)	61.5% (59.3-63.7)	78.9% (77.1-80.6)	85.2% (80.3-89.6)	28.3% (22.8-34.2)	7.9% (2.4-13.3)
Kentucky	51.9% (50.2-53.7)	76.0% (73.8-78.1)	86.0% (81.6-89.8)	46.6% (40.2-53.0)	13.0% (7.9-18.3)	61.0% (59.4-62.6)	78.0% (76.1-79.9)	83.5% (79.7-88.7)	27.9% (23.3-32.3)	7.1% (2.9-13.5)
Louisiana	55.1% (52.9-57.2)	77.8% (75.7-80.0)	86.8% (81.9-90.7)	41.3% (34.5-48.2)	11.4% (6.1-16.7)	61.6% (59.6-63.8)	77.2% (75.3-79.1)	82.2% (78.4-87.9)	25.3% (20.0-30.6)	6.5% (2.4-13.8)
Maine	48.8% (46.0-51.4)	70.8% (67.8-73.8)	80.4% (74.6-85.6)	45.2% (35.4-56.1)	13.4% (6.6-21.2)	61.6% (59.6-63.5)	75.8% (73.7-77.9)	81.4% (77.4-85.9)	23.1% (18.0-28.4)	7.4% (3.2-13.3)
Maryland	49.0% (47.2-50.7)	74.4% (72.2-76.6)	82.9% (77.1-89.2)	52.0% (44.9-59.3)	11.4% (4.8-19.8)	58.9% (57.0-60.6)	75.6% (73.5-77.5)	80.8% (76.3-86.8)	28.4% (23.4-33.8)	6.8% (2.3-14.9)
Massachusetts	43.9% (42.2-45.6)	66.4% (64.0-68.8)	76.4% (69.2-82.9)	51.4% (43.6-59.1)	15.0% (6.2-24.9)	61.1% (59.4-62.7)	73.0% (71.0-74.9)	77.2% (73.3-83.3)	19.5% (14.9-24.5)	5.7% (1.6-13.4)
Michigan	53.2% (51.4-55.0)	75.7% (73.6-77.8)	85.1% (79.2-89.1)	42.2% (35.6-48.7)	12.4% (5.7-17.9)	63.8% (62.2-65.5)	77.3% (75.4-79.2)	81.9% (78.3-86.5)	21.1% (17.1-25.4)	6.0% (1.9-12.2)
Minnesota	47.5% (45.9-49.1)	70.7% (68.4-73.0)	80.3% (72.9-85.9)	48.9% (42.1-56.4)	13.6% (4.7-22.0)	62.6% (60.9-64.3)	77.4% (75.3-79.5)	82.2% (77.5-87.8)	23.7% (19.2-28.5)	6.1% (1.6-13.0)
Mississippi	56.5% (54.2-58.6)	79.9% (77.8-81.8)	88.5% (83.7-92.0)	41.5% (35.6-48.3)	10.7% (5.0-15.5)	62.0% (59.9-64.2)	78.0% (76.0-79.8)	83.0% (79.1-88.1)	25.7% (20.7-31.2)	6.5% (2.3-13.2)
Missouri	50.7% (48.6-52.7)	73.8% (71.5-76.3)	83.5% (78.1-88.2)	45.6% (38.3-53.8)	13.0% (6.4-19.4)	61.4% (59.6-63.1)	78.2% (76.4-80.0)	83.9% (80.0-88.5)	27.3% (22.8-32.0)	7.3% (3.0-13.5)
Montana	46.3% (44.6-48.2)	69.8% (67.4-72.3)	80.1% (73.0-85.7)	50.6% (43.0-58.5)	14.8% (5.6-22.8)	58.7% (56.8-60.6)	75.4% (73.3-77.7)	81.0% (75.9-86.7)	28.6% (23.1-34.2)	7.3% (2.0-15.5)
Nebraska	51.4% (49.5-53.3)	75.6% (73.6-77.6)	86.4% (79.3-90.0)	47.2% (41.2-53.8)	14.2% (5.8-19.3)	62.6% (60.9-64.3)	79.9% (78.1-81.6)	86.2% (81.4-90.1)	27.7% (23.4-32.2)	7.8% (2.5-12.9)
Nevada	47.8% (45.1-50.4)	71.0% (68.4-73.6)	82.1% (75.0-87.0)	48.8% (39.3-59.6)	15.5% (7.2-22.6)	60.6% (58.0-63.1)	76.1% (73.8-78.2)	81.7% (77.0-86.9)	25.6% (18.8-32.0)	7.4% (2.9-13.8)
New Hampshire	44.6% (42.6-46.6)	70.0% (67.3-72.7)	80.7% (72.7-87.0)	57.2% (48.1-66.5)	15.1% (5.7-24.3)	60.4% (58.5-62.3)	77.0% (74.9-79.2)	82.5% (77.3-87.8)	27.6% (22.0-33.8)	7.1% (2.2-14.2)
New Jersey	46.4% (44.1-48.8)	68.8% (66.2-71.2)	78.4% (71.3-84.7)	48.3% (39.1-58.2)	14.0% (5.6-22.9)	60.6% (58.5-62.9)	76.1% (74.1-77.9)	81.5% (77.1-86.6)	25.5% (20.1-31.0)	7.1% (2.5-14.0)
New Mexico	45.7% (43.7-47.9)	74.8% (72.5-77.2)	85.8% (78.6-90.4)	63.7% (55.1-73.0)	14.5% (6.1-20.7)	54.7% (52.6-56.5)	75.9% (74.0-77.7)	83.5% (77.9-88.4)	38.7% (32.8-44.9)	10.0% (3.6-16.5)
New York	47.9% (45.9-49.9)	68.6% (66.1-71.2)	76.5% (70.5-83.7)	43.5% (35.6-51.8)	11.4% (4.5-21.3)	59.4% (57.9-60.9)	73.4% (71.5-75.2)	78.3% (74.1-84.0)	23.7% (19.5-28.3)	6.7% (2.2-14.5)
North Carolina	50.7% (49.1-52.3)	75.7% (73.5-77.7)	86.0% (80.1-90.1)	49.2% (42.9-55.9)	13.7% (6.4-19.0)	60.9% (59.4-62.4)	76.5% (74.6-78.4)	82.0% (77.6-87.1)	25.8% (21.4-30.2)	7.1% (2.5-13.7)
North Dakota	51.1% (49.1-53.1)	74.0% (71.5-76.5)	83.7% (76.3-88.3)	45.1% (38.2-52.7)	13.0% (4.2-19.5)	64.7% (62.8-66.5)	80.6% (78.5-82.6)	85.9% (81.4-90.8)	24.7% (20.0-29.6)	6.5% (1.6-12.3)
Ohio	50.9% (49.2-52.7)	75.7% (73.6-77.9)	86.6% (80.8-90.2)	48.7% (42.0-55.1)	14.3% (7.3-19.2)	62.3% (60.7-64.0)	77.8% (76.0-79.6)	83.8% (79.8-87.9)	24.9% (20.4-29.4)	7.7% (3.1-12.9)
Oklahoma	49.6% (47.6-51.6)	77.1% (74.9-79.2)	87.7% (81.5-91.6)	55.5% (47.8-62.9)	13.8% (6.4-19.5)	60.6% (58.5-62.4)	78.8% (76.8-80.7)	84.7% (80.6-89.7)	30.1% (25.1-35.6)	7.6% (2.9-14.0)
Oregon	48.3% (46.5-50.2)	71.2% (69.0-73.5)	80.5% (73.5-86.1)	47.4% (40.3-55.1)	13.0% (4.5-21.3)	60.6% (58.2-63.0)	73.9% (71.5-76.2)	78.5% (73.6-84.2)	21.9% (15.7-28.5)	6.2% (1.4-13.8)
Pennsylvania	53.3% (51.5-55.1)	73.2% (71.0-75.3)	82.1% (76.5-86.6)	37.3% (31.0-43.5)	12.2% (5.6-18.0)	62.0% (60.3-63.5)	76.5% (74.8-78.3)	81.1% (77.3-86.8)	23.5% (19.1-28.0)	5.9% (1.9-13.3)
Rhode Island	44.3% (42.5-46.0)	71.1% (68.5-73.6)	82.1% (74.8-87.9)	60.4% (51.8-69.5)	15.4% (6.6-24.0)	61.5% (59.9-63.1)	76.6% (74.4-78.6)	82.0% (77.4-86.8)	24.6% (19.8-29.2)	7.0% (2.4-13.5)
South Carolina	52.6% (50.4-54.7)	76.3% (73.8-78.5)	86.0% (80.2-90.1)	45.1% (38.1-52.9)	12.7% (6.4-17.9)	61.7% (59.7-63.6)	76.6% (74.5-78.9)	81.8% (77.4-86.7)	24.2% (19.3-29.5)	7.0% (2.6-13.6)
South Dakota	50.2% (47.9-52.2)	74.0% (71.2-76.6)	83.9% (76.9-88.7)	47.5% (39.0-56.7)	13.4% (5.0-20.3)	63.6% (61.9-65.4)	78.2% (76.1-80.1)	83.1% (78.4-88.4)	23.0% (18.4-27.7)	6.4% (1.4-13.1)
Tennessee	49.2% (47.3-51.1)	76.0% (73.7-78.0)	86.1% (80.2-90.5)	54.5% (47.3-61.8)	13.2% (6.1-19.3)	59.3% (57.8-60.9)	76.3% (74.2-78.3)	81.7% (77.1-87.4)	28.7% (24.0-33.6)	7.1% (2.0-15.0)
Texas	49.7% (48.0-51.4)	75.5% (73.5-77.4)	84.6% (78.1-89.4)	51.9% (45.8-58.6)	12.0% (4.1-19.0)	60.4% (58.5-62.3)	78.5% (76.6-80.3)	84.1% (79.6-89.3)	30.0% (24.7-34.9)	7.1% (2.1-13.6)
Utah	47.0% (45.2-48.8)	70.3% (67.8-72.6)	80.5% (72.9-85.8)	49.7% (42.1-57.8)	14.4% (5.3-21.9)	57.9% (55.9-59.9)	75.2% (72.9-77.5)	81.2% (76.1-86.6)	29.8% (24.2-35.7)	8.0% (2.5-15.2)
Vermont	48.0% (45.7-50.5)	67.1% (64.3-69.8)	75.7% (69.1-81.9)	40.0% (31.3-49.1)	12.8% (4.8-21.4)	59.9% (57.7-62.2)	73.5% (71.1-75.9)	78.7% (73.8-83.8)	22.8% (16.5-29.6)	7.0% (2.5-13.4)
Virginia	47.0% (45.1-48.9)	73.9% (71.9-76.0)	84.6% (77.2-89.5)	57.4% (49.6-65.5)	14.4% (5.6-21.2)	59.1% (56.7-61.6)	76.4% (74.0-78.8)	82.3% (76.9-88.0)	29.5% (22.5-36.4)	7.7% (2.5-15.0)
Washington	47.0% (45.3-48.8)	70.0% (67.8-72.2)	78.1% (71.2-85.6)	48.8% (41.8-56.6)	11.6% (3.6-21.9)	59.0% (57.3-60.5)	74.5% (72.5-76.3)	78.8% (74.3-85.8)	26.3% (21.5-31.1)	5.8% (1.1-14.9)
Washington, D.C.	56.0% (53.4-58.7)	66.7% (63.5-69.8)	73.2% (66.1-78.0)	19.1% (12.0-27.4)	9.7% (1.8-14.8)	54.3% (52.3-56.3)	65.3% (62.7-68.0)	70.4% (64.7-75.1)	20.3% (13.8-26.9)	7.8% (2.0-14.0)
West Virginia	54.0% (52.4-55.8)	77.6% (75.6-79.7)	87.4% (83.1-90.7)	43.7% (38.4-49.9)	12.6% (7.6-17.0)	62.6% (61.0-64.2)	79.6% (77.7-81.4)	85.5% (81.9-89.8)	27.2% (22.8-31.6)	7.4% (3.4-12.8)
Wisconsin	52.0% (50.3-53.9)	73.2% (70.8-75.7)	83.1% (77.1-87.2)	40.8% (34.2-47.5)	13.5% (6.0-19.2)	64.8% (63.2-66.4)	78.0% (76.1-79.8)	82.9% (79.1-87.0)	20.4% (16.5-24.4)	6.3% (2.3-11.8)
Wyoming	50.4% (47.2-53.7)	71.2% (68.5-73.7)	80.0% (73.3-85.8)	41.3% (31.7-51.8)	12.3% (4.6-20.3)	62.9% (60.2-65.7)	75.9% (73.8-78.1)	80.8% (76.3-85.6)	20.7% (14.6-27.8)	6.4% (2.0-12.6)

Prevalence (%) 40.0% – 90.0%

Percent Change (%) 5.0% – 65.0%

SM Table 4: Prevalence of overweight and obesity by 5-year age group and sex in 1990, 2021 and 2050 at the national level, across 50 states and Washington DC

2-4 years	Male			Female		
Location	1990	2021	2050	1990	2021	2050
USA	23.8 (15.7-33.6)	21.1 (15.2-27.9)	26.5 (18.4-35.2)	19.3 (11.7-29.1)	22.1 (15.4-30.4)	30.1 (19.3-41)
Alabama	23.8 (12.1-40.3)	23.4 (12.7-36.9)	31 (18.3-48.5)	17.9 (8.1-32.7)	25.5 (12.8-42)	37 (20.3-56.6)
Alaska	27.5 (14.1-44.4)	22.5 (12-35.6)	28.3 (15.3-44.6)	24.8 (11.4-42.6)	24.7 (13.2-41.2)	32.6 (17.1-52)
Arizona	21.8 (11.3-37.2)	20.5 (11.3-32.2)	26.9 (14.7-41.2)	15.7 (6.6-28.9)	22.6 (12.2-38.4)	34.9 (17.4-55.9)
Arkansas	26.7 (14.7-42.6)	22.7 (12.5-34.9)	27.7 (15.9-40.2)	20.8 (9.4-36.4)	25.4 (13.6-40.3)	34.2 (18.8-56.1)
California	21.9 (12-35)	19.4 (10.9-31.5)	23.6 (12.9-38)	18.4 (8.6-31.5)	19.2 (10-33.4)	23.6 (11.5-43.4)
Colorado	23.2 (12.3-38.4)	18.1 (9.5-30.6)	22.4 (11.6-36.5)	18.9 (8.2-33.9)	18.3 (8.9-30.6)	24.1 (11.1-39.4)
Connecticut	23.7 (13.1-37.9)	20.4 (11-31.9)	25 (13.8-39.1)	17 (7.5-30.9)	20.8 (10-34.4)	28.9 (13.5-47.1)
Delaware	25.4 (12.7-41.9)	19.6 (10.1-32.1)	22.8 (11.6-36.5)	20.3 (9.2-36.3)	24.8 (12.6-41.8)	36.3 (17.4-57.9)
District of Columbia	23.3 (12.1-38.2)	17.2 (9.2-29.1)	19.6 (10.6-32.8)	20.5 (9.4-36.4)	21.7 (10.6-37.2)	26.3 (12.5-43.5)
Florida	23.4 (12.6-38.9)	19.6 (11-31.1)	23.9 (14.2-37.9)	19.3 (8.7-34)	21.8 (11-36.9)	29.6 (15.8-49.7)
Georgia	23.1 (12.4-38.6)	20.6 (11.6-33.1)	24.4 (13.8-37.1)	19.7 (9.4-35)	23.2 (11.9-39.9)	30.7 (15.9-50.1)
Hawaii	27.4 (14.6-44.4)	21.8 (11.4-35.2)	27.3 (14.1-42.9)	17.9 (8.4-31.8)	20.3 (9.8-34.2)	27 (12.9-43.7)
Idaho	21.9 (11.2-36.2)	19.1 (10.2-31.7)	23.6 (12.8-39.2)	18.1 (8.5-32.1)	21.9 (11.3-35.9)	30.7 (15.1-49.8)
Illinois	23 (12.5-37)	21.1 (10.9-33.4)	26.5 (13.2-41.9)	21 (10-37.2)	22.1 (11-36.3)	28.3 (13.8-46.2)
Indiana	23.7 (12-39.3)	21.8 (12.2-35.4)	29.1 (16.1-47.1)	18.4 (8-32.1)	23.6 (12.2-39.2)	32.9 (17.8-52.6)
Iowa	24.3 (12.3-40)	22.3 (12.1-35.5)	28.3 (15.3-44.2)	18.8 (8.8-34.5)	23.6 (11.8-38.6)	35.7 (18.1-54.4)
Kansas	24.6 (12.9-39.6)	22.9 (12.9-35.5)	30.5 (17.4-47)	22.3 (10.7-36.9)	25 (12.8-40.6)	35.8 (17.6-56)
Kentucky	23.6 (13-38.6)	22.8 (12.7-36.3)	29.1 (16.2-43.8)	20.6 (9.3-36.4)	25.1 (12.8-40.8)	36.6 (19.7-56.8)
Louisiana	23 (11.5-38)	21.4 (11.6-34.6)	26.1 (14.1-41.7)	24.4 (11.9-40.3)	24.4 (12.8-40.3)	32 (16.8-51.1)
Maine	26 (13.3-41.7)	21.9 (12.1-34.4)	25.6 (14.4-39.5)	20.9 (9.6-37.9)	22.4 (11.3-37)	29.7 (16.1-47.1)
Maryland	26.1 (13.7-41.9)	21.4 (11.6-34.7)	25.3 (13.1-41.5)	20 (9.6-36.6)	22.2 (11-37.8)	28.3 (13.5-46.3)
Massachusetts	26.5 (14.2-42.9)	20.4 (10.8-32.7)	22.3 (12.5-34.9)	17.2 (8.1-31)	20 (9.9-33.6)	27.2 (12.9-44.6)
Michigan	27.4 (14.4-41.5)	22.1 (12.7-34.7)	27.9 (15.5-43.5)	21.4 (10.1-37.5)	23.8 (12.8-38.7)	32.3 (17.7-50.5)
Minnesota	24.6 (12.7-38.6)	21.2 (11.4-34.5)	26.1 (14.3-42)	17.3 (7.8-30.7)	21.3 (11-35)	28.5 (14.2-45.6)
Mississippi	25.6 (13.5-42.4)	22.9 (12.6-36.2)	27.4 (14-44.3)	23.1 (10.8-40.6)	28.3 (14.4-45.1)	39.8 (21.2-58.4)

2-4 years	Male			Female		
Location	1990	2021	2050	1990	2021	2050
Missouri	23.4 (12.6-38.2)	21.6 (11.9-34.1)	27.6 (14.5-42.8)	20.3 (9.5-35.7)	23.7 (12.1-39.1)	31.8 (16.6-50.9)
Montana	26.3 (14.4-42.5)	21.8 (12-35.3)	26.8 (14.4-42.8)	19.4 (8.8-33.2)	22.3 (10.5-38.1)	31.5 (15.5-50.2)
Nebraska	25.8 (13.9-41.7)	22.6 (12.6-35)	28.3 (15.9-43.3)	17.5 (7.9-32.1)	21.5 (10.8-36.2)	31.8 (16.5-51)
Nevada	25.9 (14.2-42.5)	19.8 (10.3-32.5)	24.1 (13.2-38.4)	19.9 (9.2-35.1)	20.4 (10-35)	26.9 (13-44.2)
New Hampshire	23.3 (12.4-38.3)	21.4 (11.7-34.9)	27.9 (14.8-43.9)	19.1 (8.7-34.3)	19.2 (9.2-33.3)	26.5 (11.2-43.4)
New Jersey	23.1 (12.3-38.1)	21.1 (11.8-35)	27.1 (15.1-41.8)	19.7 (9.2-35.6)	20.2 (9.9-34.8)	26 (12.6-43.6)
New Mexico	23.6 (12.1-40)	22.3 (12.6-35.3)	30.4 (16-46.9)	18.4 (8.5-33.2)	20.6 (10.3-35.6)	29.5 (13.7-49.5)
New York	23.4 (12.2-38.1)	20.8 (11.7-33)	26.9 (15.2-41.8)	17.6 (8-31.8)	21.2 (11.4-36)	28.6 (15.1-46.5)
North Carolina	24.2 (13.2-40.3)	20.7 (11.6-32.6)	25.7 (14.3-40.6)	21.1 (9.7-37.4)	22.3 (11.5-36.9)	27.9 (14.2-45.2)
North Dakota	23.7 (12-39)	23.5 (13-36.1)	29.3 (16.3-44.9)	17.8 (8-32.4)	23.7 (12.4-38.7)	36.8 (17.3-57.3)
Ohio	23.6 (12.2-39.1)	22.1 (12.2-34.5)	28.6 (15.2-43.9)	20.7 (9.8-37.3)	23.2 (11.8-37.6)	33.5 (18.2-50.9)
Oklahoma	24 (12.6-39.9)	23.7 (12.7-37.4)	31.3 (17.2-49.5)	19 (8.5-33.9)	25.7 (13.5-41.4)	38.2 (19.9-58.4)
Oregon	22.3 (11.3-37.4)	20.8 (11.6-34.2)	27.6 (15.6-42.5)	22.2 (10.2-38.7)	21.5 (10.6-36.1)	27.6 (14.5-44.2)
Pennsylvania	24.7 (13.5-39.2)	20.6 (11.6-32.3)	25.1 (14.3-38.2)	19.4 (9.5-35.1)	20.9 (10.4-35)	27.1 (13.6-43.8)
Rhode Island	23.3 (12.3-39.1)	19.7 (10.3-32.6)	23.6 (13.1-38.7)	16.2 (7-29.2)	20.1 (10-33.9)	26.2 (12.9-44.2)
South Carolina	26.1 (13.7-41.7)	22 (12.1-34.8)	27.4 (15.3-43.5)	20.9 (9.3-38.2)	24.2 (12.4-39.8)	32.7 (17.4-51.2)
South Dakota	23.3 (12.6-39)	20.7 (11.1-32.4)	24.9 (13.6-38)	18.8 (8.2-34.1)	23.4 (11.8-38.4)	32.8 (16.1-52.8)
Tennessee	22.7 (11.8-38.1)	21.9 (12.1-35.8)	26.7 (14.8-43.5)	19.7 (8.9-35.8)	25 (12.9-40)	35.3 (18-54.6)
Texas	23.9 (12.8-37.9)	22.4 (12.8-34.9)	30.1 (17-46.1)	18.6 (8.4-33.8)	22.8 (11.4-37.1)	32.6 (16.6-49.8)
Utah	19.9 (9.8-33.3)	18.7 (10-30.7)	23.8 (12.9-39.2)	16.5 (7.6-29.6)	19.3 (9.6-32.7)	28.3 (14.5-48.5)
Vermont	25.4 (13.4-41.2)	21.7 (12.5-34.3)	26.5 (15-41.1)	20.7 (9.3-36.5)	22.3 (11.2-37)	31.5 (15.1-49.9)
Virginia	23.4 (12.6-38.7)	21 (11.3-33.9)	27.3 (14.7-43.3)	18.3 (8.4-32.1)	22.3 (11.4-37.1)	30 (15-48.8)
Washington	24.4 (12.5-39.1)	20.5 (11.1-32.2)	23.8 (12.7-38.9)	19.2 (8.5-33.6)	21.1 (10.8-35)	26.1 (13.5-43.8)
West Virginia	24.6 (12.9-39)	23.4 (13.1-37)	30.6 (18.7-46.7)	20.9 (9.7-36.5)	23.6 (11.9-39.7)	32.9 (16.8-52.6)
Wisconsin	23.7 (12.1-38.8)	21.1 (12.1-34.2)	25.7 (13.7-40.5)	17.5 (8-31.1)	23 (11.7-38.6)	34.8 (17.7-56)
Wyoming	26.3 (13.5-42.6)	22.3 (12.3-35.1)	30.4 (15.9-45.4)	22.4 (10.6-39.3)	22.4 (11.1-36.8)	29.6 (14.5-45.2)

SM Table 4: Prevalence of overweight and obesity by 5-year age group and sex in 1990, 2021 and 2050 at the national level, across 50 states and Washington DC

5-9 years	Male			Female		
Location	1990	2021	2050	1990	2021	2050
USA	26.9 (18.7-36.4)	30.9 (24-37.9)	37.7 (27.2-46.7)	25.2 (16.1-35.5)	33.1 (25.2-42.6)	42.8 (29.8-54.5)
Alabama	26.6 (14.3-41.7)	33.6 (20.8-47.9)	42.6 (27.5-61)	23.3 (11.7-39.6)	37.7 (22.8-55.4)	51 (32.7-69.2)
Alaska	31 (17.7-47.3)	32.9 (19.2-47.7)	39.9 (23-55.6)	31.9 (15.6-51.5)	36.9 (21.1-54.4)	45.6 (25.5-64.1)
Arizona	24.9 (13.4-40.2)	30.6 (17.9-46)	38.7 (22.4-56.3)	20.5 (9.4-36.6)	34.2 (19-51.1)	48.6 (27.5-68.2)
Arkansas	30.8 (17.6-48)	33.2 (20.7-47.7)	38.8 (24.8-54.5)	27.1 (13.8-44.6)	37.4 (22.2-55.9)	47.6 (28.7-67.8)
California	24.7 (14.2-38.2)	28.9 (18.3-41.8)	34.4 (21.1-49)	24 (12.5-39)	29.1 (16.8-44.8)	34.8 (19.4-51.9)
Colorado	26.2 (14.1-41.9)	26.5 (15.4-40.6)	32.2 (18.8-46.4)	24 (11.5-39.4)	27.9 (14.3-43.4)	35 (18.9-53.8)
Connecticut	26.6 (14.7-41.6)	29.3 (17.3-43.1)	34.9 (20.3-50.7)	22.6 (10.7-38.4)	31.3 (17.1-48.6)	41.1 (23.7-61.8)
Delaware	29 (16.1-44.6)	28.9 (16.6-44.5)	33.2 (19.3-49.7)	26.2 (12.6-43.8)	36.4 (19.8-54.9)	50.7 (28.3-70.9)
District of Columbia	26.5 (14.5-42.5)	26 (14.5-40.8)	29.5 (15.8-45.7)	27.2 (13.3-45.1)	33 (18.7-50.3)	38.5 (21.8-57.8)
Florida	26.9 (14.8-42.3)	29.3 (17.2-42.9)	34.6 (20.7-49.1)	25.7 (13.2-42)	33.1 (19.1-49.3)	42.6 (24.6-61.3)
Georgia	26.5 (13.8-41.5)	30.6 (18.2-44.8)	35.7 (21.7-51.2)	25.6 (12.4-43)	34.2 (19.9-51.6)	43.6 (24.7-63.7)
Hawaii	30.8 (17.1-47.1)	31.6 (19.4-45.9)	38.2 (23.9-54.4)	24.2 (11-41.7)	31.3 (16.2-48.1)	40.1 (20.4-59.5)
Idaho	24.4 (12.5-39.1)	28.1 (16.7-42.4)	33.8 (21-49.3)	23.8 (12-38.2)	33.4 (18-51.6)	44.1 (23.4-65.2)
Illinois	25.3 (14.4-39.5)	30.6 (18.8-45.8)	37.4 (22-52.6)	27.1 (13.1-43.3)	33 (17.8-51.3)	40.6 (22-61.4)
Indiana	26.9 (14.7-43.3)	32.1 (20.1-47)	40.3 (24.4-56.6)	24.1 (11.5-40)	35.5 (20.3-53.2)	46.8 (27.7-66.9)
Iowa	27.4 (14.9-42.8)	32.1 (19.2-47.5)	39.7 (24.7-56.7)	25 (12.1-43.6)	34.6 (20.1-52.4)	49.3 (29.4-67.9)
Kansas	28 (15.6-44.9)	33.6 (20.6-50.2)	43 (27-59.1)	28.6 (14.5-45.4)	36.3 (20-54.4)	48.9 (29.2-68.4)
Kentucky	27.5 (14.7-43.3)	32.8 (19.4-48.6)	40.9 (23.7-58.4)	26.4 (13.1-44)	37.1 (21.1-55.6)	50.1 (29.8-69.5)
Louisiana	26.5 (14.8-42.2)	31.1 (18.7-46.7)	37.4 (22.2-52.9)	31.4 (16.9-49.8)	36.9 (21.8-54.9)	46.2 (28.1-66)
Maine	29.6 (16.8-47)	32.3 (19.6-48.4)	37.1 (22.3-55.5)	27.3 (13.3-45.3)	33.8 (18.8-51)	42.5 (23.3-63.1)
Maryland	29.6 (16.7-46.3)	30.8 (19.1-46)	35.6 (22-51.8)	25.8 (12.2-43.9)	32.6 (18.5-49.4)	40.1 (22.2-58.8)
Massachusetts	29.2 (16.3-46.1)	29.8 (18-44.5)	32.4 (19.8-47.1)	22.7 (11.3-40.8)	30 (16.1-46.3)	38.5 (20.7-59.8)
Michigan	30.8 (17.8-46.8)	32.3 (19.2-47.4)	39.2 (23.7-55.7)	28.4 (14.2-45.6)	35.2 (20.4-52.7)	45.3 (26.5-63.2)
Minnesota	27.8 (15.6-43.6)	31.1 (18.6-45.2)	37.7 (21.9-54.2)	22.3 (10.4-37.4)	31.9 (18.2-49)	41 (22.9-61.4)
Mississippi	28.9 (16.1-45.2)	33.5 (20.1-50)	38.9 (22.8-55.8)	29.7 (14.6-48.1)	41.3 (25.8-58.9)	54.3 (34.5-72.4)

5-9 years	Male			Female		
Location	1990	2021	2050	1990	2021	2050
Missouri	26.9 (14.6-43.8)	31.7 (19.2-46.9)	39.1 (23.3-56.2)	26.4 (13-42.9)	35 (19.4-52.9)	44.9 (25.4-64.5)
Montana	29.7 (16.9-45.4)	31.8 (19.2-46.7)	38 (22.5-54.5)	24.9 (12.2-41.9)	33.5 (17.9-52.2)	44.2 (22.2-65.1)
Nebraska	29.5 (17-47)	33.2 (20.5-48.3)	40.7 (25.6-57.7)	22.7 (10.9-38.2)	32.8 (18.1-50.3)	45 (25.3-63.9)
Nevada	29.7 (16.3-47.5)	29.4 (17-43.3)	34.6 (20.1-50.5)	26 (12.1-43.6)	30.8 (17.6-47.5)	38.8 (20.8-57.4)
New Hampshire	26.4 (13.8-42.6)	31.2 (18.6-45.4)	39.4 (22.3-55.9)	24.7 (10.8-41.1)	28.6 (16.2-45.2)	37.4 (19.1-58.1)
New Jersey	26.2 (14-41)	31 (19.2-46.1)	38.4 (23.5-57)	25.5 (13-41.8)	30.3 (16.5-46.6)	38 (19.9-57.9)
New Mexico	26.8 (14.7-43.1)	32.5 (18.9-48.1)	41.9 (23.7-59.2)	24.5 (11.7-40.6)	31.6 (17.4-49.4)	42.1 (22.2-62.1)
New York	26.4 (14.2-42.1)	30.3 (19-43.9)	37.2 (21-53.3)	23.5 (11.5-39.1)	32.1 (18.6-48.6)	41.3 (23.7-60.7)
North Carolina	28 (15.7-43.6)	30.7 (18.3-45.3)	36.7 (21.2-54.7)	27.4 (13.6-43.9)	33.9 (19.3-51.3)	40.3 (22-60.1)
North Dakota	27 (14.8-42.4)	33.8 (20.9-50.5)	40.7 (24.3-59.5)	23.1 (11.3-39.5)	35.4 (19.9-53.2)	50.8 (27.1-70.3)
Ohio	26.5 (14.6-42)	31.9 (19.7-45.8)	39.5 (24.5-56)	26.8 (14.7-43.3)	34.8 (20.8-51)	46.8 (28.7-64.3)
Oklahoma	27 (14.8-42.9)	34.1 (20.1-49.4)	42.9 (25.5-59.1)	24.5 (12-42)	37.9 (22.3-56.3)	52.3 (29.7-73)
Oregon	25.8 (14-39.8)	30.7 (17.9-45.9)	39 (22.2-55.1)	28.4 (13.4-45.3)	32 (17.7-48.6)	39.4 (21.1-58.8)
Pennsylvania	28 (15.3-44.9)	30.2 (18.1-43.9)	35.4 (21.8-51.1)	24.9 (12.9-41.4)	31.1 (17.5-47)	38.9 (21.6-58)
Rhode Island	26.7 (13.9-42.2)	29.4 (16.8-44.9)	34.6 (20.6-52.8)	21.5 (10.3-36.9)	30.1 (16.7-46.2)	38.2 (21.3-57.5)
South Carolina	29 (16.1-44.9)	32.5 (20.6-47.5)	39.6 (26-55.3)	26.6 (12.7-43.8)	36.1 (20.2-53.8)	46.3 (26.3-66.8)
South Dakota	26.8 (14-42.3)	30.1 (17.8-44.8)	34.8 (21.2-50.9)	24.1 (12.5-41.2)	34.7 (18.5-53.4)	46.2 (25-66)
Tennessee	25.8 (13.4-41.4)	31.8 (19.4-46.9)	38 (23.4-55.4)	25.5 (12.8-42.7)	37 (21.5-53.5)	49 (28.6-68.9)
Texas	27.2 (15.3-42.4)	32.9 (20.6-46.8)	42.3 (25.7-59.6)	24.4 (12.5-40.6)	34.1 (19.8-50.1)	45.5 (25.5-64.4)
Utah	22.8 (11.9-36.3)	27.6 (16.2-41.5)	34 (20.7-49)	21.6 (10.5-36.9)	28.8 (14.7-45.5)	39.6 (20-59.4)
Vermont	28.4 (16-44.1)	31.8 (19.2-47.5)	37.2 (22.7-53.6)	26.5 (12.9-42.8)	33.3 (18.6-52.4)	44.4 (25.1-63.8)
Virginia	26.4 (14.3-42.1)	30.9 (18.8-45.8)	38.8 (22.5-55.4)	23.9 (11-41.1)	33.4 (19.9-49.5)	43.3 (24.8-62.6)
Washington	27.4 (15-43.8)	30.2 (18.6-44.4)	34.5 (20.9-50.3)	24.5 (11.5-40.7)	31.8 (17.2-49.1)	37.9 (19-55.9)
West Virginia	27.9 (15-43.1)	34.6 (22.1-50.3)	43.3 (29.3-60.1)	27.3 (13.9-43.9)	35.5 (20-53.7)	46.7 (26.6-65.8)
Wisconsin	26.9 (15-42.3)	30.8 (18.7-45.4)	36.5 (21.7-51.6)	23.4 (11.1-39.3)	34.2 (19.1-51.4)	48.2 (26.2-68.3)
Wyoming	30 (16.9-46.8)	32.8 (18.6-47.5)	42.4 (25.1-58.1)	29.4 (14.7-48.3)	34.1 (19.6-51.3)	43.3 (23.4-62.8)

SM Table 4: Prevalence of overweight and obesity by 5-year age group and sex in 1990, 2021 and 2050 at the national level, across 50 states and Washington DC

10-14 years	Male			Female		
Location	1990	2021	2050	1990	2021	2050
USA	23 (16.4-30.3)	41.9 (34.7-49.7)	49.3 (39.5-58.6)	22.3 (15.2-30.6)	41.7 (33.2-51)	51.6 (38.6-62.8)
Alabama	22.8 (11.9-35.8)	45.2 (29.8-61.4)	54.2 (37.6-70.6)	20.7 (10.5-35.2)	47 (28.9-65)	60.3 (40-78.1)
Alaska	26.5 (14.2-40.2)	43.6 (29.3-58.9)	51 (33.9-68.2)	28.4 (14.8-45.4)	45.8 (29.3-63.4)	54.9 (35.2-72.3)
Arizona	21.3 (11.1-35.4)	41.6 (27.6-57.5)	50.1 (33.2-66)	17.8 (8.7-30.7)	43 (26.7-60.1)	57.4 (35.4-76.7)
Arkansas	26.1 (14.6-41.4)	45 (30.5-60.3)	51.6 (35.6-66.5)	24 (12.6-41)	47 (31.3-64.3)	57.8 (39.9-75)
California	20.9 (11.2-32.1)	39.2 (26.4-53.7)	45.4 (30.1-61.4)	21.1 (11.3-33.7)	37.5 (24-54.4)	43.7 (27.3-61.5)
Colorado	22.1 (12.6-34.6)	37 (23.5-52.4)	43.4 (28.7-58.6)	21.5 (10.7-36.2)	35.6 (21.3-52.8)	43.5 (25.7-62.2)
Connecticut	22.4 (12.2-35.3)	40.3 (25.8-56)	46.6 (30.2-63.1)	19.6 (9.7-32.4)	39.6 (22.9-57.1)	50.2 (29.2-69.7)
Delaware	24.6 (13.4-37.5)	39.4 (25.9-55.3)	44 (29.7-61.1)	23.4 (11.7-39.4)	45.6 (28-62)	59 (38.1-76.5)
District of Columbia	22.4 (11.7-35.4)	35.5 (22.9-50.6)	39.9 (25.9-56.3)	24 (12.1-40.3)	41.5 (23.9-59.2)	47.5 (26.8-67.1)
Florida	23 (12.7-36.8)	40.3 (26.4-54.3)	46.4 (30.3-62)	22.4 (11.6-37.8)	41.5 (25.3-58.8)	51.3 (33-68.7)
Georgia	22.1 (12.3-35.2)	41.5 (27.7-58)	47.2 (31.9-65.9)	22.6 (11.3-38.3)	43 (26.2-60.3)	52.7 (32.8-71.1)
Hawaii	26 (14.7-41.4)	42.4 (27.5-57.8)	49.5 (32-65.2)	21 (10.2-35.2)	39.2 (24.1-56.6)	48.5 (28.7-66.6)
Idaho	21 (11.6-34.5)	38.8 (24.5-55)	45.5 (29.1-60.6)	20.7 (9.9-36.3)	42 (25-59.6)	53.6 (34.2-73.8)
Illinois	21.9 (12.4-34)	41.8 (27.9-58.3)	49.5 (33-67.3)	24.4 (12.3-40)	41.3 (25.6-58.6)	48.8 (30.3-67.3)
Indiana	23.1 (13.2-36.5)	42.7 (28.4-57.9)	51.3 (35.9-68)	21.1 (10.6-36)	44.5 (28.2-62.7)	55.5 (34.7-75.4)
Iowa	23.4 (13.3-37.1)	43.2 (29.3-59.1)	51.7 (35.8-68.9)	22.1 (10.8-37)	44 (26.9-61.7)	58.4 (35.2-76.9)
Kansas	23.9 (12.6-38.1)	44.5 (30.1-60.2)	54.5 (37.1-70.3)	25.6 (12.9-43.1)	44.8 (28-63.6)	57.4 (36.2-76.1)
Kentucky	23.3 (13-36.6)	44.5 (30.2-61.6)	53.1 (35.6-70.4)	23.8 (12.1-39.3)	46.1 (27.8-63.8)	58.8 (36.7-75.9)
Louisiana	22.4 (11.9-34.8)	42.2 (27-56.3)	49.1 (34.3-64.8)	27.7 (14.2-43.7)	45.1 (28-63.1)	54.3 (35.7-72.5)
Maine	25.4 (13.9-39)	43.3 (28.4-58.2)	48.6 (33.2-64.9)	24 (12.3-39.8)	42.6 (26.9-59.7)	52.5 (32.6-69.9)
Maryland	25.3 (14.4-40.1)	41.8 (26.6-57.4)	47.3 (29.9-63.2)	23.1 (11.8-38.4)	41.1 (25.7-58.4)	49.3 (31.8-68.2)
Massachusetts	25.1 (13.6-38.9)	40.8 (26.6-56.2)	43.5 (28.8-60.8)	19.6 (9.8-34.1)	38.1 (22.6-54.5)	47 (27.3-65.3)
Michigan	26.8 (15-42.1)	43.8 (29.6-59.2)	51.2 (34.5-68.4)	24.8 (13.2-41.5)	44 (27.6-62)	53.8 (33.3-73.7)
Minnesota	23.7 (13.2-36.2)	42.1 (28.2-57.2)	49.1 (32-65.7)	19.9 (9.7-34.1)	40.2 (24.4-58.5)	49.8 (28.8-69.8)
Mississippi	24.8 (13.6-39)	44.8 (29.9-59.1)	50.7 (33.7-66)	26.8 (13.8-44.7)	50.7 (32.7-68.6)	63.3 (43.5-80.8)

10-14 years	Male			Female		
Location	1990	2021	2050	1990	2021	2050
Missouri	22.9 (12.8-36.7)	42.6 (28.2-58.6)	50.8 (34.3-67.6)	23.6 (11.5-38.4)	44 (27.7-61.6)	54.3 (34.1-72.6)
Montana	25.3 (14.4-40.7)	42.6 (27.9-58)	49.4 (32.8-66.1)	22.3 (11.1-37.5)	41.7 (25.1-60.9)	52.7 (31.1-71.2)
Nebraska	25.3 (14.5-40.5)	44.2 (29.3-59.8)	51.8 (35.5-68.5)	20.5 (10.3-35.7)	41.3 (24.4-58)	54.3 (31.6-72.4)
Nevada	25 (14.1-38.5)	40.1 (26.5-55.7)	46.4 (31.5-63.1)	23.1 (11.4-39.7)	39.5 (23.1-56.7)	47.7 (27.6-67.5)
New Hampshire	22.3 (11.8-36)	41.9 (26-58.4)	50.8 (31.7-69.1)	21.8 (10.6-37.6)	36.5 (21.7-54.3)	46.2 (26.7-65.9)
New Jersey	22.2 (12.4-34.1)	42 (28-57.3)	49.8 (33.9-66.1)	23.1 (11.9-37.6)	39.3 (23.9-58)	47.2 (29-67.1)
New Mexico	22.7 (12.6-35.5)	44.1 (30.4-59.3)	54.4 (35.4-70.5)	21.5 (10.9-37.5)	39.8 (24.2-57.1)	51 (29.4-69.2)
New York	22.6 (13.2-34.6)	41.9 (27.6-56.4)	49.5 (33.2-63.7)	20.4 (10.1-34.2)	40.1 (23.4-58.2)	49 (30.2-70.3)
North Carolina	23.8 (13.2-37.8)	41.1 (27.3-57)	47.7 (31-65)	24 (11.9-39.6)	42.9 (27-60.7)	50.1 (30.8-70)
North Dakota	23.1 (12.4-35.8)	45.1 (30.8-60.1)	52.7 (35.6-66.6)	20.7 (10-35.4)	43.9 (26.9-61.6)	58.9 (35.1-77.2)
Ohio	22.5 (12.6-34.6)	42.7 (28.1-57.9)	51.2 (34.3-67.2)	23.4 (11.9-39)	43.7 (27.5-61.7)	56.6 (37.1-73.9)
Oklahoma	23.1 (12.8-37.1)	45.4 (30.1-60)	55.2 (37.6-69.8)	21.8 (10.8-37.1)	46.8 (29.9-65.1)	60.5 (40.7-78.7)
Oregon	21.7 (11.9-35)	41.5 (27-56.8)	50.7 (32.9-67.7)	25.2 (13.4-42.9)	40.5 (24-58.5)	48.6 (30.2-67.6)
Pennsylvania	24 (13.3-37.3)	41.6 (26.7-57.1)	47.4 (31.3-64.5)	22.3 (11.3-37.2)	39.5 (24.1-57)	48 (30.8-66.6)
Rhode Island	22.7 (12.4-34.9)	40 (25.9-55.1)	46.1 (30.7-62.4)	19 (9.2-33.9)	38.8 (23.4-56.4)	47 (28.5-65.7)
South Carolina	25 (13.7-40)	43.6 (27.8-59)	51.5 (34.4-67.6)	23.5 (12.4-38.9)	44.8 (28.6-62.2)	55.1 (35-74.2)
South Dakota	22.6 (12.2-35.9)	41.1 (27-57.5)	46.6 (30.9-62.9)	21.7 (11-37.8)	43.7 (26.9-61.4)	54.7 (33.6-75.2)
Tennessee	21.8 (11.8-34.7)	42.5 (28.4-57.8)	49.2 (33.4-65.6)	22.8 (10.9-37.7)	46.6 (29.5-62.9)	58.7 (38.6-76.9)
Texas	23.2 (13.6-35.5)	44.7 (31.2-58.8)	54.4 (37.8-69.2)	21.4 (10.6-35.4)	42.7 (27.3-58.4)	54.4 (33.4-73.4)
Utah	19.4 (10-31.6)	38.1 (24.6-52.9)	45.3 (28.4-61)	18.8 (9.1-33)	37.1 (22.1-54.1)	48.5 (28.6-68.2)
Vermont	24.4 (13.3-38.6)	42.8 (28.1-58.4)	49.2 (32.1-66)	24 (12.1-40.5)	42.1 (26-60.4)	53.5 (31.3-72.6)
Virginia	22.4 (12.6-34.9)	41.7 (27.4-56.3)	50.1 (33.8-65.6)	21.3 (10.5-36.9)	42.2 (25.9-59.7)	52.4 (32.8-71.1)
Washington	23.7 (13.2-37.1)	41.2 (27.5-57.5)	45.8 (30.4-62.5)	22 (11.6-37.2)	40.1 (24.7-58.5)	46.2 (28-65.7)
West Virginia	24.2 (13-38.4)	45.4 (30.7-61.6)	54.8 (38.5-71.6)	23.9 (12.1-39.9)	44.7 (27.1-62.4)	56.2 (36.7-73.4)
Wisconsin	23 (12.7-36.7)	41.9 (26.8-57.8)	47.9 (31.3-65.1)	20.5 (9.8-36.7)	43 (27.3-59.9)	57.1 (35.7-75.6)
Wyoming	25.3 (13.4-39)	43.8 (29.6-58.9)	54.4 (36.2-69.5)	26 (13.2-43.1)	42 (25.9-60.5)	51.4 (33.1-69.7)

SM Table 4: Prevalence of overweight and obesity by 5-year age group and sex in 1990, 2021 and 2050 at the national level, across 50 states and Washington DC

15-19 years	Male			Female		
Location	1990	2021	2050	1990	2021	2050
USA	26.1 (23.7-28.7)	40.2 (35.6-45)	47.6 (40-54.6)	22.9 (19.9-26.3)	45.6 (39.9-51.6)	55.8 (45.5-64.3)
Alabama	25.3 (20.1-31.7)	44.9 (36.7-53.3)	54.6 (42.6-66)	20.4 (14.7-27.1)	56.9 (47.7-65.5)	69.8 (58.2-79.5)
Alaska	32.1 (24-41.2)	41.5 (32.5-51)	49.3 (37-59.9)	32.4 (23-42.6)	51.7 (40.9-63.1)	60.6 (46.2-71.7)
Arizona	22.9 (17.5-28.9)	38.9 (31.6-46.8)	47.5 (36.6-57.7)	18.5 (12.6-25.6)	48.5 (39-58.6)	63.2 (47.9-75.9)
Arkansas	30.2 (21.6-39.1)	42.7 (33.1-52.6)	48.9 (38.2-60)	24.1 (16.7-33.7)	53.6 (42.6-64.7)	64.1 (51.2-76.6)
California	24.2 (18.5-30.4)	37.6 (30.1-46.1)	43.8 (33.6-54.6)	22.1 (17.2-27.2)	38.8 (30.5-47)	45.3 (32.5-57.5)
Colorado	23.7 (17.7-30.7)	32.8 (26.1-39.9)	39.1 (30.2-48.1)	21.7 (15.1-29.8)	35.5 (27.2-44.8)	43.8 (31.2-55.6)
Connecticut	24.3 (18.9-30.7)	36.3 (28.6-44.1)	42.6 (32.7-52.8)	18.6 (12.1-26.5)	40.8 (30.8-51.1)	51.9 (37.3-65.6)
Delaware	27.7 (19.9-36.6)	36.5 (27.8-46.2)	41 (31.1-52.7)	22.7 (15.9-31.2)	53 (43.1-63.9)	66.3 (50.9-77.6)
District of Columbia	27.7 (20.9-35.2)	32.5 (23.1-43.8)	37 (25.6-49.6)	24.9 (16.7-34.4)	46 (33-59.1)	52.3 (36.5-66.9)
Florida	25.2 (19.9-31.5)	38.2 (31-45.7)	44.6 (34.8-54.1)	23.1 (16.5-31.8)	44.5 (33.8-56.3)	53.9 (40.2-66.9)
Georgia	27.9 (22-34.6)	38 (30.6-45.8)	43.6 (34.3-53)	23.1 (16.9-30.7)	47.7 (38.9-57.2)	57.7 (45.6-70.1)
Hawaii	30.8 (24-38.3)	39.8 (31.8-48.5)	47.2 (35.4-57.7)	23.1 (16.8-30.6)	40.7 (31.3-50.2)	50.2 (37.4-62.6)
Idaho	22.3 (17.2-28.2)	34.9 (27.3-42.9)	41.5 (31.6-51.5)	20 (13.8-26.7)	45.9 (36.2-55.7)	57.3 (44.4-69.4)
Illinois	23.3 (18.3-28.8)	39.5 (32.3-47.5)	47.1 (36.1-57)	26.7 (20.5-33.3)	43.8 (34.7-53.8)	51.8 (40-64)
Indiana	26.8 (20.8-34)	42.5 (33.1-51.2)	51.9 (39.7-62.8)	21 (15.4-27.8)	48.5 (39.7-57.7)	60.1 (48.1-73)
Iowa	24.8 (18.7-31.9)	38.7 (31.4-45.5)	47.2 (37.3-56)	20 (14.4-26.5)	48.2 (40-56.6)	63.1 (49.5-73.1)
Kansas	24.4 (17.5-33.1)	42.3 (35.6-49.1)	52.3 (41.3-61)	26.2 (17.7-35.6)	50.7 (42-59.7)	63.4 (49.2-72.9)
Kentucky	27.9 (22.2-33.9)	42.2 (34.6-50.6)	50.9 (40.5-61.5)	26 (19.9-33.3)	51.6 (42.2-60.3)	64.6 (50.8-74.7)
Louisiana	26 (19.7-33.4)	38.4 (30.5-46.8)	45 (35.3-55.3)	33.2 (25.8-42.6)	50.6 (40.5-60.7)	60.5 (48.5-70.9)
Maine	28.2 (21.2-35.6)	37.2 (29.5-45.8)	42.6 (32.8-53.2)	24.5 (16.5-34)	46 (34.9-56.9)	55.8 (41.3-68.2)
Maryland	28.2 (22.4-34.4)	38.1 (30.7-46)	43.5 (34.8-52.5)	24.1 (17.7-31.8)	42.9 (34.5-52.4)	50.9 (40.1-64.2)
Massachusetts	30.3 (23.7-36.8)	34.6 (28-42.1)	37.3 (29.8-46.3)	19 (13-26.8)	42.9 (33.5-51.9)	52.4 (38.2-64.4)
Michigan	31.7 (25.6-38.3)	44 (37-51.2)	51.6 (41.3-61.8)	24.8 (18.9-32)	49.5 (40.8-57.9)	60.1 (48.6-70.2)
Minnesota	26.4 (20.8-32.8)	39.4 (31.3-47.7)	45.9 (35.3-55.9)	18.6 (14.2-24.4)	40.8 (33-48.9)	50.9 (38.6-63.2)
Mississippi	30.2 (23.3-37.7)	42.1 (33.4-50.6)	48.1 (37.6-59.2)	28.9 (21-37.3)	60.6 (51.4-69.2)	71.9 (59.8-81.1)

15-19 years	Male			Female		
Location	1990	2021	2050	1990	2021	2050
Missouri	26.5 (20.9-33.1)	42.5 (35.2-49.9)	51 (41.3-60)	24.5 (17.4-32.7)	48.9 (38.9-58.2)	59.3 (45.3-70.4)
Montana	31.3 (23.4-41)	39.4 (31.5-47.4)	45.9 (36.2-55.8)	23.3 (15.5-32.5)	46.6 (37.2-56.1)	58.5 (44.2-70.2)
Nebraska	30.6 (24-38.1)	43 (36-50.4)	50.8 (41-59.6)	19.1 (12.9-26.2)	46 (37.6-54.2)	59.3 (44.2-69.5)
Nevada	30.2 (22.7-39.1)	38.9 (30.3-47.6)	45 (33.6-54.9)	24.9 (16.2-34.7)	43 (32.8-53.3)	52.1 (38.1-64.5)
New Hampshire	22.6 (16.8-29.3)	40.2 (31.1-50)	49 (34.9-61.6)	21.5 (14.7-29.3)	36.7 (26.6-47.9)	46.6 (30.5-60)
New Jersey	22.7 (16.8-29.4)	40.8 (33.4-49)	48.9 (37.2-59.1)	21.8 (15-30.1)	41.1 (32.3-50.5)	49.8 (37.2-62)
New Mexico	25.6 (19.4-32.4)	45.5 (37.6-53.6)	56 (44.8-65.5)	22 (15.4-29.7)	42.2 (32.5-52.5)	53.5 (40.1-64.7)
New York	26.2 (20.7-32.1)	41 (34.6-48.4)	49.1 (38.2-58.2)	20.9 (15.2-27.8)	44.3 (35.6-53.2)	54.3 (40.1-67.3)
North Carolina	29.9 (24.5-36.2)	38.3 (31.1-46.3)	45.1 (34.1-55.9)	27.7 (21.1-34.5)	44.4 (34.8-53.8)	51.9 (39.7-64.5)
North Dakota	26 (20-32.6)	41.1 (32.7-50.7)	48.4 (36.4-60.6)	20.4 (13.6-28.2)	47 (37.4-57.4)	62.3 (44.8-75.1)
Ohio	25 (19.6-31.2)	40.2 (33-47.8)	48.7 (38.4-58.2)	26.2 (19.2-34.2)	48.7 (40.2-57.2)	61.4 (48.8-71.9)
Oklahoma	24 (17.8-30.8)	46.4 (37.6-54.7)	56.1 (44.1-66.6)	20.6 (14.4-28)	53.6 (44.9-62.4)	67.3 (54.5-77.8)
Oregon	22.6 (16.4-30.1)	40.8 (32.5-49.6)	50.3 (38.3-61)	26.6 (20.1-34.3)	43.9 (34.9-53.4)	52.5 (39-63.4)
Pennsylvania	27.8 (21.7-34.3)	37.5 (30.3-44.7)	43.4 (34.2-52.7)	22.4 (16.5-29.1)	39.1 (30.8-47.1)	47.7 (36.2-58.8)
Rhode Island	27.2 (21.4-33.8)	35.7 (27.9-44.3)	41.6 (31.2-52.7)	18 (12.9-24.7)	39.1 (28.8-49.1)	47.8 (33.6-63)
South Carolina	29 (22.8-36.4)	42.5 (33.5-51.5)	50.3 (39.4-61.8)	24.6 (17.9-32.1)	51.5 (41.7-61)	61.7 (48.9-73.5)
South Dakota	24.4 (18.6-31.3)	33.9 (26.4-41.1)	39.2 (29.1-51)	22.1 (15.3-30.1)	48 (36.9-58.5)	59.6 (44.8-72.6)
Tennessee	23.3 (18.5-28.5)	40.4 (32.6-48.6)	47.3 (37.4-58.5)	24.8 (18.3-32)	53.1 (43.2-62.6)	64.8 (51.8-76.4)
Texas	26.2 (21.5-31.9)	47.3 (40.3-54.7)	57.1 (46-66.3)	21.2 (15.6-28)	50.7 (42.4-59)	62.5 (48.5-73.5)
Utah	19.8 (15-25.7)	32.9 (25.2-40.9)	39.8 (29.6-50.8)	17.3 (12.1-23.1)	39.5 (31.8-48.4)	51.8 (38.1-62.8)
Vermont	25.4 (18.9-32.9)	38.7 (30.2-47.9)	45 (34.5-55.4)	26.8 (19-36.4)	45.4 (36.2-56)	57.1 (42.2-67.7)
Virginia	24.7 (18-31.9)	39.1 (31-47.8)	47.6 (35.7-57.8)	20.2 (13.6-28.2)	45.8 (37.3-54.1)	56.4 (43.5-68.6)
Washington	24.4 (19.2-30.2)	39.5 (32.3-46.6)	44.4 (35.5-54)	20.6 (14.9-27.5)	41 (33.4-49.5)	47.6 (36.6-60.2)
West Virginia	28 (22.8-34)	44.1 (36-52.5)	53.4 (44.2-64.1)	26 (20.3-33.2)	48.5 (39-58.9)	60.1 (48.7-72.6)
Wisconsin	26.3 (20.3-33.1)	38.3 (30.6-46.1)	44.9 (35.3-54.6)	21.1 (14.8-28.4)	49.4 (40-59.2)	63.8 (48.6-75.5)
Wyoming	25.1 (17.9-33.9)	40.2 (31.3-49.9)	50.4 (36.3-62.4)	24.6 (16.5-34.8)	44.9 (34.4-55.9)	54.7 (38.4-67.4)

SM Table 4: Prevalence of overweight and obesity by 5-year age group and sex in 1990, 2021 and 2050 at the national level, across 50 states and Washington DC

20-24 years	Male			Female		
Location	1990	2021	2050	1990	2021	2050
USA	37.1 (34.6-39.8)	53.6 (48.6-58.3)	60.7 (52.2-66.8)	29.3 (26.2-32.7)	56.4 (50.6-62.4)	66 (56.6-73.8)
Alabama	37.2 (31.6-42.9)	58 (49.8-65.9)	66.7 (55.6-75.8)	28.1 (22.4-33.7)	62 (53.5-69.7)	74 (64.2-82.1)
Alaska	41.5 (33.3-49.3)	57.5 (49.7-65.5)	64.6 (52.3-73.3)	33.7 (25.2-43.1)	58.2 (48.5-67.3)	66.8 (54.1-77.1)
Arizona	33.8 (28.9-39.3)	54.4 (47.5-61.2)	62.7 (51.1-71.1)	23 (17.2-29.6)	56.6 (47.6-66.2)	70.3 (56-81)
Arkansas	39.7 (31.6-47.9)	56.6 (47.4-65.2)	62.6 (52-71.7)	36.7 (29.1-45.7)	64.6 (55.7-72.5)	73.5 (62.5-82.2)
California	35.3 (29.4-41.5)	52.3 (43-60.5)	58.4 (46.5-68.4)	26.4 (22.4-31)	51.9 (44.4-59.5)	58.6 (48.4-68.5)
Colorado	30.8 (25-36.7)	45.6 (39-52.3)	52.2 (42.3-60)	23.1 (17-30.2)	49.5 (40.2-58.5)	57.9 (44.7-68)
Connecticut	35.1 (29.3-41.3)	52.4 (45.8-59.3)	58.6 (49.4-66.8)	23 (16.9-30.5)	53.9 (43.9-64.7)	64.4 (50.2-76.3)
Delaware	38.2 (29.9-47.6)	49.7 (40.1-59.3)	54.2 (44.3-64.3)	34.5 (27.3-42.1)	60 (51.1-68.7)	72.4 (58.2-81.9)
District of Columbia	32.9 (27.2-39.2)	44 (35-53.1)	48.5 (37-59.1)	32.4 (25.1-41)	48.1 (37.3-59)	54.1 (39-68.4)
Florida	36.7 (31.5-42)	51.5 (44.5-58.5)	57.6 (47.9-66)	28.9 (21.1-38.3)	55.2 (44.5-65.7)	64.4 (52-76.5)
Georgia	37.5 (32.2-42.6)	52.7 (45.8-60.3)	58 (48.5-66.8)	33.9 (27.8-40.1)	56.9 (49.3-65.4)	66.1 (55.6-76.2)
Hawaii	39.7 (34-46.2)	55.3 (46.6-63.2)	62.1 (51.3-70.6)	25.8 (20.1-32)	53 (44-62.2)	62.3 (50.3-72.5)
Idaho	32.5 (27.4-37.9)	51 (43.5-58.7)	57.7 (48.2-66.5)	29.6 (23.7-35.9)	56.4 (47.6-64.7)	67.1 (55.5-77)
Illinois	36.5 (31.7-41.5)	51.8 (44.4-58.6)	58.8 (48.2-67.7)	29.6 (24.4-35.1)	57.2 (48.9-65.1)	64.7 (53.7-74)
Indiana	36.8 (30.3-43.5)	55.3 (47-63.9)	64 (52.8-73)	31.3 (26-36.6)	61 (53.6-68.4)	71.5 (61.6-80.2)
Iowa	38.5 (32.6-44.5)	57.3 (50.1-63.9)	65 (55.7-72.7)	31.1 (24.9-37.5)	63.7 (56.2-71.4)	76.2 (64.6-83.6)
Kansas	45.5 (38.2-53.6)	60.2 (53.9-66)	69.1 (59.1-75.4)	33.5 (25.6-42)	63 (55.4-70.8)	74.2 (62.5-82.1)
Kentucky	36.2 (31.3-41.6)	59.4 (52.1-66.9)	67.3 (57.7-75.3)	35.9 (30.4-41.7)	65.2 (57.2-72.3)	76.2 (66.1-83.4)
Louisiana	38.4 (31.9-45.2)	57.1 (49.9-64.3)	63.6 (55.4-72.3)	32.2 (25.2-39.5)	64.7 (56.3-72.7)	73.1 (64.2-81.1)
Maine	39.6 (33-46.6)	51.6 (43.9-59.1)	56.7 (47.1-65.3)	33.4 (25.4-42.1)	55.9 (45.3-66.3)	65.3 (52.5-77.1)
Maryland	37.7 (32-44)	49.8 (42.8-56.9)	55.3 (45.8-63.4)	25 (19.8-31)	57.3 (49.3-65.3)	64.9 (54.7-75.7)
Massachusetts	35.8 (31-41.2)	48.7 (41.9-55.5)	51.6 (43.7-58.7)	24 (18.9-29.7)	41.9 (34.6-49.7)	51.5 (38.9-63)
Michigan	40.4 (35.3-45.5)	52.4 (45.3-59.4)	59.7 (48.8-68.3)	34.5 (28.7-40.4)	58.1 (50.3-65.7)	68 (57.4-77.2)
Minnesota	37 (31.6-42.6)	52.3 (44.7-60.1)	58.9 (46.8-67.8)	22.7 (18.4-27.4)	55.1 (47.8-63.2)	64.6 (53.3-75.9)
Mississippi	37.8 (31-45)	59.5 (51.9-66.7)	65 (54.6-73.4)	39.3 (32.1-47.3)	65.5 (57.9-72.8)	76.3 (66.3-84)

20-24 years	Male			Female		
Location	1990	2021	2050	1990	2021	2050
Missouri	36 (30.5-41.7)	52.3 (45.5-58.7)	60.3 (50-67.9)	31.5 (25.3-38.5)	60.3 (51.7-68.5)	69.9 (59-78.7)
Montana	34 (28.5-39.9)	51.1 (43.7-58.3)	57.6 (46.7-65.7)	28 (22-34.3)	53.7 (44.7-62.3)	64.9 (51-74.4)
Nebraska	39.3 (33.6-45.5)	54.7 (47.8-61.1)	62.2 (53.5-69.3)	26.5 (21.4-32.7)	57.7 (50-65.4)	69.9 (58-79.2)
Nevada	41.4 (33.4-50)	54.5 (45.9-62.3)	60.4 (49.7-68.8)	33.6 (25.1-42.2)	52.6 (43.1-62.2)	61.5 (49.4-72.2)
New Hampshire	37.7 (31.5-44.4)	55.8 (47.8-63.1)	64.3 (52-73.4)	29.2 (22.9-36.4)	55.5 (46.5-64.5)	65.1 (52.1-75.3)
New Jersey	40.6 (33.4-47.9)	49.8 (42-56.6)	57.8 (45.6-66.3)	31.1 (23.8-38.5)	49.1 (40.6-57.2)	57.6 (46.5-67.3)
New Mexico	34 (28.8-39.7)	56.8 (49.7-64.4)	66.6 (54.8-74)	32.5 (25.2-40.4)	59.1 (50.2-68.1)	69.6 (57.2-78.9)
New York	38.1 (33.4-43.4)	52.2 (45.8-58.5)	59.9 (49.3-68.1)	27.2 (21.2-33.4)	51.4 (42.6-60.3)	61 (47.9-73.1)
North Carolina	35.8 (31.2-40.5)	56.5 (49.2-63)	63 (53.6-70.9)	31.4 (26.4-36.9)	58.5 (50.2-66.7)	65.4 (54.5-75)
North Dakota	40.6 (34.6-46.3)	59.5 (51-67.4)	66.4 (56-75.5)	27.1 (21.6-33.1)	58.2 (49.6-67.1)	72.3 (56.8-82.1)
Ohio	34.4 (29.5-39.5)	55.1 (48.3-61.9)	63.2 (54.1-70.7)	28 (23-33.9)	61.8 (53.8-68.9)	72.7 (63-80.7)
Oklahoma	42.2 (35.6-49.1)	56.7 (49-63.8)	65.8 (55-73.9)	31.4 (24.8-38.8)	64.8 (56.8-72.2)	76.6 (65-85)
Oregon	37.1 (30.1-44.9)	48.9 (40.3-57.7)	58.1 (46.8-67)	30.8 (25.2-37)	54.5 (46.4-62.4)	62.6 (51.4-71.5)
Pennsylvania	38 (32.7-43.9)	52.8 (45.6-59.5)	58.5 (49.9-66.1)	30.8 (25.4-37)	57.7 (49.4-65.3)	65.6 (56.2-74.8)
Rhode Island	36.4 (31.2-42)	54.1 (46.1-62)	60 (50.2-68.9)	23.3 (18.1-29.5)	51.6 (42-60.6)	60.2 (48.2-72.1)
South Carolina	40.9 (35.1-47.4)	56.8 (48.6-64.5)	64.2 (53.4-72.5)	31.9 (26-38.2)	60 (50.9-68.8)	69.5 (58.6-79.1)
South Dakota	41 (35.2-47.2)	56.9 (49.6-63.8)	62.1 (51.8-71.9)	29.5 (22.7-37.3)	58 (48.2-67.7)	68.7 (53.9-80.7)
Tennessee	38.1 (33.1-42.9)	56.6 (48.7-64.2)	63 (53.5-71.8)	29.6 (24.3-35.5)	62.9 (54.8-71.2)	73.5 (61.8-83)
Texas	37.4 (31.8-42.8)	57.8 (50.9-64.5)	66.9 (56-74.6)	32.7 (26.9-38.6)	59.1 (51.6-66.5)	70.2 (58.3-79.2)
Utah	27.8 (22.4-33)	48.9 (41-57)	56.1 (45.5-65.7)	22 (17.5-27)	48.9 (40.7-57.8)	61.1 (49.1-72.1)
Vermont	35.2 (27.8-42.5)	49.2 (40.7-57.7)	55.2 (44.5-65.2)	27.1 (20.3-35)	53 (44.3-61.8)	64.3 (50.5-75.7)
Virginia	37.9 (30.6-45.6)	53.9 (45.1-62.6)	62.1 (49.6-71.5)	25.7 (20.2-31.8)	55.9 (47.9-64.1)	65.8 (54.1-76.5)
Washington	36.6 (31-42.2)	48.4 (41.8-55.4)	53.2 (44.1-62.1)	29.8 (24.4-35.8)	51.7 (44.1-59.5)	58.3 (47-69.4)
West Virginia	42.2 (36.7-47.6)	60.8 (53.5-67.5)	69.2 (60.8-76.1)	31.5 (26.3-37.5)	63.1 (54.4-70.8)	73 (63.2-81.3)
Wisconsin	40.6 (35.4-45.6)	55.7 (48.2-62.7)	61.8 (52.4-69.9)	27.5 (21.9-33.5)	60.2 (52-67.8)	73.2 (61.2-82.1)
Wyoming	38.1 (29.8-47)	57.9 (49.8-65.6)	67.3 (55.3-75.9)	33.7 (25.3-43.6)	56.5 (46.8-66.1)	65.4 (51.8-76)

SM Table 4: Prevalence of overweight and obesity by 5-year age group and sex in 1990, 2021 and 2050 at the national level, across 50 states and Washington DC

25-29 years	Male			Female		
Location	1990	2021	2050	1990	2021	2050
USA	48.3 (45.7-50.8)	62.5 (57.6-67.2)	69.9 (63.7-76)	34.6 (31.5-37.7)	65.9 (60-71.1)	77.2 (68.6-83.6)
Alabama	50.8 (45.2-56.4)	65.3 (57.5-72.5)	72.8 (63.5-82.1)	36 (30.8-41.5)	73.9 (67.1-80.4)	85.9 (78.3-91)
Alaska	50.7 (43.1-57.8)	62.9 (54.8-70.4)	69.3 (59.2-79.9)	42.2 (34-50.9)	68.6 (59.8-76.9)	76.1 (64.9-85)
Arizona	45.4 (40.6-50.4)	62.8 (56.1-69.4)	72.1 (61.3-81.1)	33.6 (27.9-39.8)	66.6 (57.1-74.8)	79.6 (67.2-87.9)
Arkansas	53.7 (44.8-62)	63.7 (55-71.7)	71 (60.8-80.6)	40.9 (33.2-48.9)	72.1 (64.3-78.9)	83.8 (74.6-90.2)
California	45.9 (39.8-51.9)	60.5 (51.8-68.4)	66.4 (56.5-77.5)	30.2 (26-34.7)	62.5 (54.8-69.8)	72.3 (60.7-82)
Colorado	41.3 (35.8-47.4)	53.8 (46.9-60.4)	61.4 (51-71.9)	31 (24.5-38.4)	55.9 (47.5-64.1)	68.6 (53.7-79.1)
Connecticut	47.3 (41.6-52.5)	62 (54.7-68.8)	69.6 (59.6-79.1)	32.6 (25.2-40.3)	63.2 (54.2-72.1)	75.7 (61.5-85.5)
Delaware	50.1 (42.1-57.8)	63.1 (54.4-71.6)	70.3 (60.3-79.9)	36.9 (30.1-44.9)	64.7 (55.7-73.1)	76.6 (65.1-85.1)
District of Columbia	40.7 (35-46.8)	48.5 (39.6-57.2)	54.5 (44.3-64.1)	40.7 (33.1-48.5)	55.6 (44.5-65.7)	63.6 (50.8-74.2)
Florida	48.3 (43.6-53.5)	61.1 (53.9-67.9)	67.5 (57-78.2)	33.2 (25-41.9)	62.4 (51.4-72.3)	72.6 (59.3-83.1)
Georgia	48.3 (43.2-53.6)	61.4 (53.7-68.3)	68.5 (59.1-78.1)	34.2 (28.9-39.6)	70.7 (63.2-77.5)	82.1 (72.8-88.8)
Hawaii	47.1 (41.2-52.9)	64.1 (56.2-71.6)	72.2 (61.8-81.3)	29.9 (24.4-35.7)	58.6 (49.8-66.5)	69.7 (57.1-80.4)
Idaho	47 (41.9-52.5)	66.1 (58.8-72.4)	73.8 (64.8-81.9)	31.8 (26.4-37.8)	66.5 (57.9-74.4)	76.8 (66-85.5)
Illinois	49.5 (44.5-54.2)	62.3 (54.9-69.2)	69.1 (59.6-78.2)	32.7 (27.8-38.1)	65.3 (57.1-72.7)	76.6 (65.2-85.4)
Indiana	50.3 (43.9-56.8)	64.5 (56-72.1)	72 (63.4-80.6)	38.4 (33.5-43.6)	71.1 (64.4-77.8)	82.3 (73.8-88.7)
Iowa	51.6 (45.6-57.3)	67.2 (59.9-73.6)	76.5 (67.5-83.8)	37.3 (30.8-43.7)	73.4 (66.4-79.6)	85 (76-90.8)
Kansas	50 (42.7-57.4)	68.7 (62.6-74.6)	77.3 (68.7-85.1)	37.5 (29.6-45.4)	71.3 (63.8-78)	84.2 (73.2-90.1)
Kentucky	49.2 (44.1-54.4)	66.4 (59.7-73.1)	74.2 (66.3-82.1)	39.4 (33.7-45.3)	70.8 (63.6-77.7)	82.5 (74.6-88.8)
Louisiana	49.2 (43.3-55.4)	65.5 (58.4-72.2)	72.4 (64.1-81.4)	46.6 (39.4-53.7)	70.1 (62.3-77.4)	81.4 (71.2-89)
Maine	53.6 (47.7-59.6)	63.1 (56.2-70)	70.8 (62.1-79.3)	36.7 (28.6-45.9)	64.2 (53.5-73.8)	75.2 (63.4-84.7)
Maryland	49.5 (43.8-55.1)	62 (54.6-68.7)	69.2 (59-78.9)	34.3 (28.9-39.6)	67.7 (59.9-75)	77.9 (66.9-87.6)
Massachusetts	49.1 (44.2-54.1)	58.4 (51.5-65.2)	64.1 (55.1-73.5)	27.5 (22.7-32.2)	55.5 (47.3-63.2)	67.4 (54.3-78)
Michigan	51.2 (46.1-56.2)	64.3 (57.6-70.8)	70.8 (63.2-78.9)	39 (33.5-44.9)	68.8 (61.5-75.3)	80.3 (71.3-87.2)
Minnesota	49 (43.6-54.7)	63.2 (55.7-70.7)	70.1 (59.6-79.1)	30 (25.5-34.5)	61.4 (53.6-68.6)	73.1 (60.2-82.5)
Mississippi	53.3 (46.5-59.9)	65.1 (57.4-71.9)	72.3 (63-81.3)	45.2 (37.8-52.2)	75.5 (68-82.1)	85.7 (77-91.6)

25-29 years	Male			Female		
Location	1990	2021	2050	1990	2021	2050
Missouri	49.3 (43.7-54.6)	66.2 (59.4-72.3)	74.4 (66.2-81.9)	36.4 (30.2-42.8)	65.8 (57-73.2)	77.7 (67.5-85.6)
Montana	43.3 (37.6-48.6)	66.6 (58.9-73.4)	73.7 (63.5-82.7)	30.8 (24.9-37)	66.6 (58-74)	77.7 (66.6-86.2)
Nebraska	46.2 (40.8-51.7)	67.9 (61.8-73.6)	77.1 (67.9-84.4)	35.8 (30.2-41.9)	69.4 (62.2-75.9)	82.5 (71.3-88.6)
Nevada	50.5 (42.3-57.7)	61.1 (53-69.4)	69.1 (58.2-78.6)	37.1 (29.1-46.3)	65.1 (56.2-73.6)	77.9 (65.7-86.6)
New Hampshire	49.8 (43.8-56)	63.3 (55.3-70.4)	71 (59.8-80.3)	30.6 (24.9-36.9)	68.2 (60.1-76)	79.4 (67.6-88)
New Jersey	50.9 (44.2-58)	64.8 (57.9-71.4)	72 (63.4-80.2)	35.2 (28.1-42.7)	60.9 (52.2-68.6)	72.3 (59.9-81.7)
New Mexico	43 (37.4-48.7)	66.7 (59.8-73.3)	76.5 (66.7-84.1)	34.8 (28.5-42.2)	71 (62.6-78.4)	83.4 (71.8-90.6)
New York	44.4 (39.6-49)	56.7 (50-63)	63.4 (53.8-73.7)	30.7 (25.3-36.4)	58.7 (49.6-66.7)	68.1 (56.7-78.9)
North Carolina	53.2 (48.6-57.8)	63.5 (56.6-70.3)	71.1 (62.8-80.7)	36 (30.5-41.2)	71.1 (63.5-77.6)	83.1 (74.3-89.1)
North Dakota	52.2 (46.1-57.6)	69.4 (61.3-76.7)	77.1 (67.3-85.9)	36.8 (30.9-43.2)	66.2 (57.1-74.4)	78.1 (65.2-86.4)
Ohio	52.8 (47.4-57.8)	64.5 (58-70.7)	73.1 (65.4-80.3)	37.1 (31.7-42.5)	70.2 (63-76.5)	83.1 (73.5-89)
Oklahoma	49.4 (43.5-55.6)	66.9 (59.6-74.2)	75.3 (65.7-83.8)	37.9 (31.4-44.2)	71.9 (64.9-78.7)	84.5 (74.2-90.7)
Oregon	48.4 (41-55.6)	58.9 (49.9-67.3)	65.2 (54.2-75.7)	35.1 (29.5-40.9)	65.9 (58-73)	76.4 (65.1-84.5)
Pennsylvania	49.2 (43.9-54.5)	63.4 (56.5-69.6)	69.8 (61.6-79)	39.4 (33.9-45.2)	65.8 (58.1-72.8)	76.5 (66.5-84.4)
Rhode Island	51.5 (46-57)	64.2 (56.6-71.5)	71.6 (61.6-80.4)	27.6 (21.7-33.2)	66 (57.4-73.6)	78.5 (66.4-87)
South Carolina	49.9 (43.8-56.3)	63.9 (55.5-71.6)	71.1 (61.4-80)	40.3 (34.2-46.3)	69.8 (61-77.7)	81.7 (71.3-89)
South Dakota	49.2 (44.2-55.2)	64.7 (57.9-71.4)	71.9 (62.7-80.7)	38.1 (31.2-45.6)	69 (59.7-76.8)	80.5 (68.8-88.1)
Tennessee	47.9 (42.9-52.9)	64.3 (56.7-71)	71.6 (62-80.1)	35.8 (29.8-41.6)	71 (63.3-77.8)	82.9 (73.3-89.7)
Texas	47.6 (42.4-53)	65.4 (58.5-71.6)	73.4 (64.1-82.2)	36 (31-41.6)	69.3 (62.1-76)	80.1 (68.5-87.4)
Utah	41.6 (35.8-47.5)	60.9 (52.7-68)	69.2 (58.2-78.6)	33.4 (27.9-38.7)	63.4 (55.4-70.8)	75.2 (62.7-84)
Vermont	47.1 (39.9-54.6)	61.2 (51.9-69.1)	68 (58.3-77.2)	35.4 (28.5-42.4)	64.4 (56.1-72)	73.6 (63-82.4)
Virginia	45.9 (37.9-53.6)	64.2 (55.3-72.2)	72.3 (61.1-82.3)	33.3 (27.6-39.1)	67.9 (60.7-74.8)	80.6 (68.7-88.7)
Washington	46.8 (41.7-51.6)	60.6 (53.5-66.9)	66.5 (57.2-77.3)	32.4 (27.3-38)	62.8 (55.6-69.8)	72.3 (60.8-82.6)
West Virginia	53.9 (48.5-59.1)	68.9 (61.6-75.2)	77.2 (69-85.2)	39.2 (33.6-44.3)	72.5 (64.7-79)	84.2 (77.3-89.8)
Wisconsin	49.6 (44.4-54.5)	63 (56.1-69.6)	70.3 (62-78.2)	38.1 (32.9-44)	67.1 (59-75.1)	78.8 (68-86)
Wyoming	50.7 (41.8-59.6)	62.1 (54-70.4)	68.9 (58.7-78.5)	41.1 (31.2-51.2)	64.1 (54.3-72.4)	74.4 (61.6-84.1)

SM Table 4: Prevalence of overweight and obesity by 5-year age group and sex in 1990, 2021 and 2050 at the national level, across 50 states and Washington DC

30-34 years	Male			Female		
Location	1990	2021	2050	1990	2021	2050
USA	55.2 (52.6-57.6)	71.9 (67.6-76.1)	77.9 (72.2-83.5)	39.2 (36.1-42.7)	69.7 (63.9-74.9)	80.2 (71.7-85.8)
Alabama	60.2 (54.5-65.4)	73.6 (67.2-79.8)	79.8 (72.4-87.1)	44 (38.6-49.4)	77 (70.1-83)	87.7 (80.2-92.3)
Alaska	54 (46.7-61.2)	70 (62.6-76.9)	75.7 (66.7-84.4)	45.2 (37.2-53.3)	69.6 (59.8-77.8)	77 (65.4-85.4)
Arizona	48.8 (43.9-53.8)	72.3 (65.9-78.4)	79.7 (70.9-87.8)	36.3 (29.8-43)	69.7 (60.4-77.7)	81.9 (69.5-89.9)
Arkansas	56.2 (47.3-64.9)	74 (65.9-80.9)	79.5 (71.1-87.8)	41.9 (33.5-49.9)	77.2 (69.7-83.6)	87.3 (79-92.7)
California	54 (47.6-60.6)	70.6 (62.8-77.6)	75.7 (67.1-85.1)	38.9 (34.4-43.6)	65.8 (58.6-72.5)	75.3 (63.4-83.8)
Colorado	47.2 (41.6-53.2)	65.4 (58.9-72.2)	71.9 (62.7-81.1)	35.8 (29.3-43)	63.2 (54.3-71)	74.8 (60.8-83.6)
Connecticut	56.4 (50.9-61.3)	73.7 (67.3-79.5)	79.6 (71.6-86.9)	34.3 (27.4-41.8)	69.6 (60.7-77.8)	80.4 (68.1-89.3)
Delaware	57.8 (49.7-65.1)	70.5 (62-77.7)	76.7 (67.2-85.3)	43.9 (36.9-51.1)	72.9 (64.3-79.9)	82.8 (72.7-89.6)
District of Columbia	47 (41-53.3)	60.5 (51.8-68.7)	66.1 (56.5-74.6)	45.9 (37.8-54.3)	58.7 (48.2-69.2)	66.2 (52.6-77.1)
Florida	52.5 (47-57.5)	71.3 (64.7-78)	76.5 (68.5-85.1)	37.6 (29.6-46.1)	66.6 (55.7-76.7)	75.9 (63.5-85.9)
Georgia	55.7 (50.4-60.4)	70 (63.2-76.3)	76.1 (68.5-84.5)	39 (33.9-44.4)	73.1 (65.2-79.4)	83.9 (74-90)
Hawaii	51.2 (45.4-57.1)	73.3 (66.1-79.9)	79.8 (71.3-88.1)	36 (30.7-41.8)	64.7 (56.1-72.4)	74.9 (63.3-84.3)
Idaho	49.6 (44.5-54.9)	71.7 (64.8-78.2)	78.4 (70.7-86.7)	38.1 (32.5-43.8)	68 (58.6-75.9)	78 (67.6-86.3)
Illinois	55 (50.4-59.6)	72.1 (66-78.3)	77.7 (69.9-85.8)	39.6 (34.7-44.6)	71.4 (63.1-78.3)	81.2 (69.9-88.4)
Indiana	58 (51-64.7)	71.9 (64.5-78.8)	78 (69.4-85.7)	41.7 (36.6-46.9)	74.1 (66.9-80.5)	84.3 (75.6-90.2)
Iowa	55.4 (50-60.5)	77.2 (71.4-82.7)	84.2 (77-89.6)	40.3 (34-46.7)	72.9 (64.9-79.6)	84.7 (75.1-90.4)
Kansas	54.7 (47.4-61.7)	75 (69.2-80.4)	82.2 (74.7-88.7)	38.8 (30.9-47.6)	73.7 (66.3-79.9)	85.8 (75.4-91.3)
Kentucky	57.3 (52-62.3)	74.9 (68.4-80.8)	81.2 (74.3-87.8)	44 (38.8-49.7)	74.4 (67.2-80.9)	85 (77.5-90.5)
Louisiana	57.4 (51.3-63.2)	72.9 (66.5-78.9)	78.7 (71.6-87.1)	42.9 (36.2-49.6)	76.9 (69.9-83.3)	86.3 (78-91.9)
Maine	57 (51.4-62.3)	69.4 (62.3-75.7)	76.2 (68.1-83.3)	40.8 (32.9-48)	65.8 (55.5-75.1)	76.7 (65-85.5)
Maryland	52.5 (47.1-57.9)	72.7 (66.2-78.4)	78.3 (70.3-86)	39.5 (34.2-45.1)	71.3 (63.2-78.4)	80.7 (70.6-88.7)
Massachusetts	58.8 (53.8-63.7)	65.2 (58.6-71.5)	70.2 (62-79.9)	32.7 (27.8-38.3)	63.1 (55.2-70.8)	73.9 (61.3-83.9)
Michigan	57.9 (53.2-62.9)	72.5 (66.1-78.7)	77.8 (70.3-85.2)	41.5 (36.5-47.1)	75 (68.3-81.2)	84.7 (76.5-90.2)
Minnesota	55.1 (50.1-60.1)	72 (64.4-78.3)	77.7 (68.7-86.2)	35.7 (31.1-40.6)	67 (59.1-74.1)	77.6 (65.2-85.7)
Mississippi	55.3 (48.7-61.7)	76.4 (69.9-82.3)	81.8 (75.1-88.7)	51.8 (45.3-58.2)	79.4 (72.8-84.9)	88.3 (81-92.9)

30-34 years	Male			Female		
Location	1990	2021	2050	1990	2021	2050
Missouri	54.7 (49.8-59.8)	74.3 (68-79.9)	80.9 (73.4-87.7)	41.3 (35.4-47.3)	71.2 (62.3-78.4)	81.8 (72-88.3)
Montana	53.5 (47.6-59.4)	70.5 (63.6-77.3)	77 (67.9-85.7)	36.1 (30.8-41.8)	66.2 (57.1-73.5)	77.4 (64.5-85.9)
Nebraska	57.8 (52.7-63)	76.1 (70.1-81.5)	83.4 (75.4-89.6)	42.4 (36.9-48.3)	75.4 (68.8-81.6)	86.4 (76.3-91.4)
Nevada	54.3 (46.8-62.5)	75.9 (68.2-82.1)	81.6 (73.7-88.2)	38.8 (30.6-48)	70.3 (61.2-78.3)	81.6 (70.3-89.2)
New Hampshire	54 (48.5-59.7)	72.8 (64.9-78.9)	79 (69.7-86.9)	34.4 (28.5-40.5)	64.4 (55.6-73)	76.6 (63.1-86.6)
New Jersey	52.2 (45.3-59.1)	70.5 (63.9-76.7)	76.9 (68.5-85)	34.1 (27.7-40.7)	63.7 (55.3-71.2)	74.6 (62.5-83.2)
New Mexico	50.2 (44.6-55.9)	75.8 (69.4-81.5)	83.6 (75.4-89.8)	38.4 (32.2-45.2)	74 (66.4-80.7)	85.5 (75.4-91.4)
New York	54.9 (50-59.5)	68.1 (61.7-73.8)	73.7 (65.4-81.8)	34.2 (28.4-40.3)	66.3 (57.7-74.6)	74.7 (64.3-84.2)
North Carolina	54.6 (50.1-59)	72 (65.8-77.8)	78.3 (70.6-85.7)	42.6 (37.4-47.8)	72.8 (65.7-79.5)	84.3 (75.7-90.1)
North Dakota	59.6 (53.9-64.9)	77.1 (70.6-83.1)	83.2 (75.3-90.6)	40.7 (34.5-47.3)	68.7 (59.4-77)	80 (68.2-87.6)
Ohio	54.4 (49.4-59.7)	73.2 (66.7-78.5)	80.3 (73.5-86.2)	38.1 (32.8-43.5)	73.1 (66.2-79.7)	85.1 (76.9-90.3)
Oklahoma	55.2 (49.6-61.2)	77 (70.4-82.6)	83.4 (76.1-90.2)	40.5 (34.7-46.5)	77.6 (70.5-83.7)	88.1 (79.6-93.3)
Oregon	52.7 (44.7-60.5)	68.6 (60.3-76.2)	74 (63.8-82.4)	38.6 (33.2-44.3)	68.6 (60.5-75.7)	78.5 (66.9-86.8)
Pennsylvania	57.4 (52.3-62.3)	68.8 (62.8-74.4)	74.5 (66.8-83.5)	43.8 (38.3-49.2)	68.3 (60-75.6)	78.6 (67.6-86.2)
Rhode Island	56.1 (51.2-61.1)	73 (66.1-79.4)	79.1 (71.1-86.8)	31.4 (26.2-37.1)	70.3 (61.4-77.8)	81.5 (69.8-89.2)
South Carolina	56 (49.8-61.8)	74.5 (67-81.4)	80 (72.1-86.7)	44.2 (38.2-50.1)	75.2 (67-82)	85.4 (76.3-91.2)
South Dakota	58.8 (53-64)	74.3 (67.9-80.1)	79.9 (70.7-87.2)	40.8 (33.3-48.2)	70.3 (61.4-78.8)	81.4 (68.7-89.4)
Tennessee	55.8 (51.1-60.5)	72.1 (65.4-78.2)	78.3 (70.1-86.3)	41.9 (36.2-47.3)	73.5 (65.3-80.6)	84.5 (75.3-90.7)
Texas	58 (52-63.5)	76.8 (71-82)	82.8 (75.8-89.9)	40.5 (35.2-46.1)	71.7 (64.8-78)	82 (72-89)
Utah	52.1 (46-57.9)	69.9 (62-76.8)	76.9 (67.8-85.3)	36.9 (31.7-42.3)	66.9 (59.4-74.1)	78 (66.3-86.1)
Vermont	56.2 (48.8-63.3)	67.7 (59.4-74.9)	73.7 (64.3-82.4)	40.1 (33.2-47.8)	61.1 (51.5-69.6)	70.6 (58.4-81.2)
Virginia	56.7 (49-64.3)	73.3 (65.3-79.9)	79.8 (70.2-87.8)	38.5 (32.9-44.9)	70.4 (63-77.2)	82.4 (71.2-89.6)
Washington	49.8 (44.5-54.9)	68.8 (61.7-75)	73.9 (65.6-83.6)	37.6 (32.3-43.3)	67.7 (59.9-74.7)	76.3 (64.7-85.5)
West Virginia	61.3 (56.5-66)	75.7 (69.4-81.3)	82.6 (75.6-89.2)	44.7 (39.3-49.8)	73 (65-79.6)	84.6 (76-90.5)
Wisconsin	60.5 (55.9-64.9)	73.9 (67.6-79.8)	79.6 (72.4-86.3)	39.1 (33.7-44.3)	70.4 (62.4-77.5)	81.2 (71.6-87.5)
Wyoming	57 (48-65.9)	73 (65.5-79.6)	78.3 (69.3-86.3)	42.1 (33-51.7)	70.1 (61.8-77.6)	79.3 (68.5-87.7)

SM Table 4: Prevalence of overweight and obesity by 5-year age group and sex in 1990, 2021 and 2050 at the national level, across 50 states and Washington DC

35-39 years	Male			Female		
Location	1990	2021	2050	1990	2021	2050
USA	59.5 (56.8-62)	76.3 (72-80.3)	81.6 (76.6-86.3)	44.3 (41-47.5)	72.4 (66.9-77.6)	82.2 (74.2-87.7)
Alabama	64 (59-69.1)	79.9 (73.1-85.3)	84.9 (78.7-90)	46.3 (40.9-51.6)	78.2 (71.7-84.1)	88.5 (81.3-92.7)
Alaska	60.8 (54-68.4)	77.7 (71-83.7)	82.2 (74.3-89.3)	47.6 (39-56.3)	73.1 (64.3-80.6)	79.8 (69.4-87.7)
Arizona	53.3 (48.8-58.1)	77.5 (71.1-82.5)	83.9 (76.5-90.1)	40.2 (33.7-46.9)	73.8 (65.3-81.3)	84.7 (74.1-91.3)
Arkansas	59.8 (50.9-67.4)	78.8 (71.4-85.2)	83.6 (76.6-89.9)	47.7 (40-55.3)	78.8 (71.7-84.9)	88.2 (80.5-93.4)
California	56.7 (50.4-62.8)	74 (66.5-80.7)	78.4 (69.2-87)	43.9 (39.5-48.5)	69.5 (62.2-75.9)	78.1 (67.2-86.1)
Colorado	51.1 (45.2-56.8)	70.3 (63.8-76.5)	76.2 (67.6-84.2)	39.7 (32.8-46.6)	64.6 (55.4-72.2)	75.8 (61.7-84.5)
Connecticut	57.8 (52-63.1)	76.7 (70.5-82.2)	82.1 (74.8-88.8)	39 (31.7-46.6)	69.7 (60.4-78)	80.6 (68.7-89.6)
Delaware	60.8 (52.4-68.8)	76.8 (68.5-83.7)	81.9 (74.1-89)	49.6 (42.4-56.7)	73.5 (65.5-80.9)	83 (73.5-90)
District of Columbia	48.2 (41.9-54.3)	65.8 (57.1-73.6)	70.7 (60.5-79.1)	48.8 (40.6-56.3)	66.1 (54.9-75.7)	72.7 (59.5-82.3)
Florida	58.9 (53.7-64.1)	74.2 (67.4-80.5)	78.8 (71.2-86.6)	42.7 (33.8-51.8)	68.6 (58.1-78.7)	77.6 (66.1-87.3)
Georgia	61.5 (55.8-66.4)	77.1 (70.7-82.9)	82.1 (75.5-88.4)	45.1 (40.1-50)	74.7 (68.5-81.2)	84.9 (75.8-91)
Hawaii	54 (48-59.4)	74 (66.3-80.7)	80.4 (71.7-88.7)	38.7 (33.6-44.5)	65.9 (56.9-74.1)	75.8 (63.9-85.3)
Idaho	59.8 (54.6-64.7)	79.2 (73-84.4)	84.4 (77.8-90)	43 (37-48.9)	70.9 (62.3-78.2)	80.3 (70.4-87.9)
Illinois	58.2 (53.3-62.8)	73.5 (67.1-79.5)	78.8 (71.2-86)	45.3 (40-50.4)	73.7 (66.2-80.4)	82.8 (72.7-89.9)
Indiana	63.7 (57-69.9)	77.1 (69.3-83.6)	82.4 (74.9-88.8)	49.5 (44.4-54.4)	74.7 (68.4-80.7)	84.7 (76.5-90.3)
Iowa	61.3 (55.8-66.7)	80.7 (74.7-85.7)	86.7 (80.5-91.8)	44.4 (37.9-50.4)	76.7 (69.4-82.8)	86.9 (78.7-92.1)
Kansas	59.8 (52.6-67)	78.7 (73-83.7)	85 (78.3-90.5)	41 (33.5-49.2)	74.9 (67.6-81.2)	86.3 (75.7-92)
Kentucky	64.3 (59.2-69)	76.4 (69.4-82.3)	82.3 (75.6-88.8)	48.9 (43.6-54.3)	74.7 (67.6-81)	85.2 (78.1-90.5)
Louisiana	63.8 (57.5-69.5)	79.1 (72.8-84.6)	83.8 (76.9-90.1)	49.3 (42.7-55.8)	80.1 (73.2-85.6)	88.4 (81-93.1)
Maine	59.5 (53.6-65)	75.2 (67.8-81.2)	80.8 (73.5-87.3)	41.5 (33.5-49.6)	70.3 (60.2-79.3)	80.1 (69.4-88.6)
Maryland	55.1 (49.6-60.6)	75.8 (68.9-82)	80.9 (73.3-88.5)	46.1 (40.3-51.3)	75.8 (68.5-82)	84 (74.3-91.1)
Massachusetts	60.6 (55.7-65.4)	73.2 (67-79.4)	77.5 (69.9-84.9)	37.3 (32-42.4)	65.3 (57.9-72.6)	75.5 (64.7-84.9)
Michigan	61.7 (56.8-66.7)	77.7 (72-82.8)	82.2 (76.4-88.5)	46.5 (41-51.9)	75.6 (69-82.1)	85.1 (76.1-90.8)
Minnesota	60.3 (55.1-65.8)	77.9 (70.6-83.9)	82.4 (74.8-89.8)	42.2 (37.7-47.2)	71.5 (64.5-77.8)	81.1 (69.9-87.8)
Mississippi	60.3 (53.4-66.4)	80.1 (74.1-85.3)	84.8 (78.3-90.7)	55.5 (48.8-62)	79 (72.7-84.8)	88 (81-92.8)

35-39 years	Male			Female		
Location	1990	2021	2050	1990	2021	2050
Missouri	61.8 (56.1-67.4)	78.6 (73-84.1)	84.4 (77.3-90)	49 (42.7-55.3)	73.5 (65.5-80.6)	83.3 (74.2-89.6)
Montana	58 (52.9-63.4)	73.4 (66.4-80)	79.3 (70.9-87)	40.3 (34.7-46.3)	65.8 (57.5-73.5)	77.2 (66-85.6)
Nebraska	61.9 (56.8-67.1)	80.2 (74.5-84.9)	86.3 (80-91.3)	46.5 (41.2-51.8)	73.5 (66.6-79.6)	85.1 (74.3-90.9)
Nevada	62.2 (54-69.2)	76.1 (68.5-82.6)	81.8 (74.8-88.2)	44.4 (36.3-52.8)	70.3 (61.1-77.9)	81.6 (70.2-89.1)
New Hampshire	57.9 (52.4-63.5)	76.9 (70-83.3)	82.4 (74.7-89.5)	38.1 (32.5-43.8)	73.5 (65.7-81.1)	83 (71.9-90.7)
New Jersey	62.1 (55.3-68.5)	77.6 (71.4-83.2)	82.7 (75.2-88.7)	38.1 (31.3-45.6)	68.5 (60.8-75.9)	78.2 (67.1-86.4)
New Mexico	53.1 (48-58.5)	75.8 (68.9-81.6)	83.5 (75.1-89.9)	42.9 (36.7-49.5)	73.7 (66-80.5)	85.1 (74.6-91.2)
New York	56.9 (52.4-61.4)	74 (68-79.4)	78.9 (71.4-85.5)	41.6 (35.7-47.8)	68.4 (59.3-76.2)	76.5 (65.7-85.2)
North Carolina	62.1 (57.3-66.5)	76.3 (69.7-82.1)	81.9 (75.2-88.1)	44.5 (39.6-49.3)	77.1 (70.6-83)	87 (78.3-92.2)
North Dakota	65.4 (59.7-71.4)	80.8 (74-86.8)	86 (79.4-92.4)	43 (37.4-49.4)	75.1 (66.8-82.1)	84.5 (74.5-90.6)
Ohio	61.7 (56.5-66.2)	78.4 (73-83.7)	84.3 (78.2-90.2)	43.2 (37.7-48.3)	74 (67-80.3)	85.5 (77-90.8)
Oklahoma	61.1 (55-66.9)	81 (74.8-86.1)	86.5 (80.3-92.2)	46.4 (40.2-52.5)	76.5 (69.1-82.6)	87.3 (78.3-92.8)
Oregon	62.8 (55.1-69.8)	73.7 (65.5-81.4)	78.5 (68.9-86.5)	43.7 (38.4-49.6)	73.3 (66.7-79.5)	82.1 (71.9-88.9)
Pennsylvania	60 (54.9-65)	77.1 (71.1-82)	81.5 (74.5-88)	46.1 (40.5-51.5)	73.5 (66.4-79.7)	82.3 (72.8-88.9)
Rhode Island	59.6 (54.6-64.9)	77.2 (70.1-82.8)	82.5 (74.6-88.9)	37.2 (31.3-43.2)	69.8 (61.1-77.4)	81.3 (71.2-89.1)
South Carolina	62.9 (57.4-68.2)	76.4 (69-83.2)	81.6 (73.6-88.5)	51.1 (45.1-57.1)	76.2 (68.5-83.1)	86.1 (77.3-91.7)
South Dakota	63.7 (58.4-68.7)	78.3 (72-84.2)	83.4 (75.7-89.6)	42.3 (34.1-49.9)	73.1 (63.4-80.9)	83.3 (72.4-90.6)
Tennessee	59.8 (54.8-64.3)	75.2 (68.3-81.4)	80.9 (73.6-88.4)	44.6 (39.1-49.8)	76.6 (68.7-83)	86.5 (78-92)
Texas	60.2 (54.9-65.2)	80 (74.6-85.1)	85.3 (78.9-91.1)	46.4 (41.3-51.7)	75.1 (68.6-80.7)	84.3 (74.9-90.2)
Utah	54.1 (48.3-60)	75.9 (68.3-82.5)	81.8 (73.3-89.1)	42.8 (37.7-48.3)	69.3 (61.9-76.4)	79.8 (68.6-87.7)
Vermont	55.1 (47.5-62.4)	73.4 (64.8-80.4)	78.7 (70.1-86.5)	43.1 (36.2-50.4)	66.7 (58.5-74.8)	75.5 (63.8-84.9)
Virginia	61.8 (53.8-68.8)	76.6 (68.2-84)	82.4 (72.6-89.8)	40.7 (34.7-46.9)	74.3 (67.7-80.5)	84.9 (74.5-90.9)
Washington	58.7 (53.7-63.5)	74.5 (67.8-80.4)	78.8 (70.3-86.4)	45.1 (39.6-50.7)	69.8 (62.9-76.4)	78.1 (68-87.2)
West Virginia	65 (60.3-69.9)	80.1 (74.3-85)	86 (80.4-91.5)	51.7 (46.2-57)	76.1 (68.7-82.2)	86.5 (79.5-91.8)
Wisconsin	60.4 (55.4-65.1)	78.6 (72.5-83.9)	83.5 (77.6-88.8)	45.4 (40.1-50.8)	74.2 (67.1-80.7)	83.9 (75.6-89.6)
Wyoming	62.7 (53.4-70.7)	76 (68.9-82.3)	80.9 (72.1-87.6)	43.5 (34.3-52.8)	71.5 (62.9-79.2)	80.2 (69.2-88.7)

SM Table 4: Prevalence of overweight and obesity by 5-year age group and sex in 1990, 2021 and 2050 at the national level, across 50 states and Washington DC

40-44 years	Male			Female		
Location	1990	2021	2050	1990	2021	2050
USA	64.5 (61.9-67.1)	79.8 (75.4-83.8)	84.4 (79.5-88.7)	49.2 (45.8-52.3)	74.2 (68.8-79.2)	83.4 (75.5-88.3)
Alabama	67.1 (61.6-72.5)	82 (75.6-87.1)	86.6 (80.4-92.1)	47.7 (41.9-53.8)	79.8 (73.5-85.2)	89.4 (83.1-93.7)
Alaska	67.1 (59.7-73.7)	80.5 (73.5-86.5)	84.5 (76.6-90.8)	49.8 (41.3-57.7)	70.3 (61.8-78.2)	77.5 (66.7-86)
Arizona	60.8 (55.6-65.8)	82.5 (77.1-87.4)	87.7 (81.4-93.2)	46.5 (39.9-52.8)	73 (63.9-80.7)	84.1 (72.2-91.2)
Arkansas	66.7 (57.9-74.7)	81.6 (74.8-87.6)	85.9 (79.7-92.1)	52.8 (44.5-61)	79.3 (72.3-85.5)	88.5 (81-93.3)
California	63.7 (57.4-69.6)	77.7 (70.1-84.6)	81.6 (73.3-89.2)	47.7 (43.1-52.5)	72.3 (65.4-78.9)	80.5 (70.3-87.9)
Colorado	56.5 (50.4-62.4)	74.7 (68-80.5)	80 (71.6-87.3)	38.7 (32.1-46)	66.8 (58.4-74.8)	77.5 (63.6-85.5)
Connecticut	60.7 (55.2-66)	78.4 (72.1-83.9)	83.5 (76.3-89.7)	41.6 (34.7-48.8)	70.2 (61.1-78.3)	80.9 (68.7-89.6)
Delaware	67.3 (58.9-74.9)	81.3 (73.6-87.6)	85.5 (78-91.9)	51.1 (44.2-59.1)	74.2 (66.2-81.1)	83.7 (73.9-90.2)
District of Columbia	61.4 (55.1-67.7)	69.8 (61.2-77.5)	74.5 (65.3-81.9)	59.8 (52-67.6)	64.9 (54.1-74.8)	71.6 (57.8-81.5)
Florida	64.1 (58.7-68.9)	77.2 (70.6-82.8)	81.6 (74.3-89.5)	49 (40-57.7)	70.9 (60.2-80.3)	79.4 (66.8-88.1)
Georgia	64.9 (59.7-70.1)	81.2 (74.6-86.5)	85.5 (78.8-91.3)	48.3 (42.7-53.8)	77.4 (70.8-83.4)	86.6 (77.8-91.8)
Hawaii	56.2 (50.2-62.1)	77.6 (70.1-83.9)	83.4 (75.6-90.4)	42.9 (37.1-49.1)	65.9 (57.8-73.7)	75.7 (63.1-85)
Idaho	64.5 (59-69.6)	81.4 (75-86.7)	86.2 (79.6-91.9)	51.1 (45.1-56.7)	70.2 (62.4-77.3)	79.7 (68.7-87.2)
Illinois	63.4 (58.7-68.1)	81.7 (75.5-86.7)	85.6 (79.3-91.3)	49.4 (44.3-54.7)	72.4 (64.6-79.4)	81.8 (71.2-89.3)
Indiana	68 (61.3-74.3)	81.2 (73.6-87.1)	85.9 (79.1-91.5)	55.9 (50.6-61.1)	77.3 (70.2-82.8)	86.4 (78.1-91.6)
Iowa	66.7 (61-72)	83.9 (78.3-88.3)	89.1 (83.8-93.2)	50.6 (44.5-57)	77.6 (70.5-83.3)	87.7 (79.2-92.2)
Kansas	64.3 (57.4-71.2)	82.2 (76.9-86.8)	87.7 (81.8-92.4)	48.1 (40.2-56.4)	77.1 (70.2-83.2)	87.7 (77.7-92.7)
Kentucky	65 (59.6-70.1)	83.3 (77-88.3)	87.7 (82.1-92.9)	52.9 (47.6-58.2)	78.7 (71.9-84.3)	87.7 (81.2-92.2)
Louisiana	63.9 (57-70.1)	79.2 (72.9-84.4)	83.8 (77.6-90.1)	54.4 (47.1-61.2)	79.8 (72.5-85.7)	88.1 (80.1-93)
Maine	69.1 (63.7-74.5)	79.4 (73.1-85.1)	84.4 (78.3-89.9)	46.6 (38.8-54.6)	71.2 (61.4-79.7)	80.8 (70.3-88.2)
Maryland	62.1 (56.8-67.6)	79.5 (72.6-84.8)	84 (77.2-90.3)	47.5 (41.6-53.4)	76 (69.1-82)	84.2 (74.8-91.6)
Massachusetts	61 (56-65.9)	77.4 (71.3-82.8)	81.1 (74.6-87.5)	42.9 (37.3-48.4)	68.4 (60.1-75.2)	78 (66.1-86.1)
Michigan	66.3 (61.3-71.3)	80 (74.2-85)	84.1 (77.8-89.9)	54.1 (48.5-59.7)	77 (70.4-82.9)	86 (77.9-91.5)
Minnesota	67.2 (62-72.2)	81.5 (74.7-87)	85.7 (79.1-91.3)	47.9 (43.2-52.7)	72.7 (65.4-79.1)	81.9 (71-88.8)
Mississippi	63.3 (56.4-69.3)	82.3 (76.2-87.6)	86.6 (80.4-91.9)	53.9 (47.2-60.5)	82.4 (76.8-87.4)	90.1 (83-94.1)

40-44 years	Male			Female		
Location	1990	2021	2050	1990	2021	2050
Missouri	65.2 (59.9-70.4)	82.8 (77.2-87.5)	87.5 (81.7-92.6)	50.7 (44.1-56.7)	76.6 (68.5-83)	85.5 (76.6-91.6)
Montana	63 (57.2-68.7)	77 (70-83.1)	82.2 (74.1-89.3)	47.7 (41.6-53.6)	68.9 (60.3-76.5)	79.4 (67.1-87.6)
Nebraska	68.4 (63.4-73.3)	84 (78.9-88.6)	89.2 (83.7-93.8)	52.3 (46.2-58.4)	77.9 (71.6-83.5)	87.9 (79-92.8)
Nevada	65.1 (57.6-72.5)	80.7 (73.8-86.4)	85.5 (79-91.2)	47.3 (38.7-56.3)	74.3 (66.2-81.8)	84.4 (73.7-91.2)
New Hampshire	63.9 (58.2-69.6)	82.7 (76.2-87.7)	87.1 (80.2-92.5)	45.7 (39.1-52.3)	72 (63.7-79.6)	82.2 (70.1-90.2)
New Jersey	63.6 (57.1-70.9)	79.4 (72.7-85.1)	84.3 (78-90.8)	42.4 (34.8-49.3)	70.5 (62.8-77.6)	79.9 (69-87.6)
New Mexico	57.6 (52-63.4)	80.1 (74-85.6)	86.6 (79-92.3)	45.4 (38.9-51.7)	78.9 (71.5-84.9)	88.4 (79.5-93.3)
New York	63.6 (58.9-68.2)	78.4 (72.6-83.2)	82.6 (76.3-88.8)	49 (42.8-55.2)	69.9 (60.4-77.5)	77.5 (66.1-86.1)
North Carolina	65.8 (61.1-70.1)	80.4 (74-85.3)	85.1 (78.8-90.8)	49.9 (44.8-54.8)	77.3 (70.6-83.1)	87.1 (78.6-92.2)
North Dakota	68.8 (63.2-74.6)	84.8 (78-89.9)	89.1 (82.9-94.2)	52.3 (46.3-58.2)	77.3 (69.2-84.1)	86 (76.5-92.1)
Ohio	67.7 (62.9-72.1)	80.6 (74.4-85.6)	86.1 (80-91.1)	52.9 (47.5-58.1)	78 (71.5-83.9)	88 (80.6-92.6)
Oklahoma	62.3 (56.2-68.4)	82.1 (76.1-87.2)	87.3 (81.7-92.8)	51.5 (45.3-57.6)	78.8 (72.1-84.8)	88.8 (81.2-93.7)
Oregon	65.1 (57.7-71.9)	78.6 (70.9-85.1)	82.7 (74.6-89.7)	47.3 (41.8-53)	73 (65.7-79.4)	82 (71.6-88.5)
Pennsylvania	66.6 (61.2-71.4)	78.5 (72.1-83.8)	82.9 (76.7-89.5)	53.3 (47.8-58.9)	75 (68.1-81)	83.5 (73.9-89.2)
Rhode Island	63.2 (57.9-68.7)	78.7 (71.3-84.2)	83.8 (76.3-89.9)	43.9 (38-49.8)	73.4 (64.6-80.5)	83.8 (72.1-90.8)
South Carolina	66.6 (61.2-72.1)	80.3 (73-86.1)	85 (77.7-91.2)	53.7 (47.4-59.8)	79.2 (71-85.7)	88 (79.6-93.3)
South Dakota	68.4 (63-73.6)	83.2 (77.4-88.2)	87.2 (81.1-92.3)	51.8 (44.4-59.6)	75.3 (65.6-82.6)	84.8 (73.8-91.5)
Tennessee	65 (60.5-69.5)	79 (72-84.8)	83.9 (76.5-90.5)	49.3 (43.4-54.7)	78.1 (70.8-84.6)	87.5 (79.8-92.6)
Texas	66 (60.8-71)	81.9 (75.9-87)	86.8 (80.3-92.3)	50.5 (45.1-55.4)	76.4 (70.1-81.9)	85.2 (75.5-90.9)
Utah	59.6 (53.5-65.8)	78 (71.1-84.2)	83.6 (75.9-90.4)	48 (42.5-53.4)	71.8 (64.1-78.4)	81.6 (70.3-88.5)
Vermont	62.1 (55.3-69.6)	76.9 (69.5-83.6)	81.5 (73-88.3)	42.5 (35.4-49.7)	68.4 (59.9-75.5)	76.9 (65.1-84.7)
Virginia	63.8 (55.7-71.1)	79.7 (71.5-86)	84.8 (76.7-91.3)	43.6 (37.5-49.5)	74.8 (67.5-81)	85.2 (74.5-91.6)
Washington	61.6 (56.1-66.3)	78.9 (72.6-84.5)	82.7 (75.1-89.4)	46.2 (41.1-51.5)	72.5 (65.2-78.9)	80.2 (69.2-88.3)
West Virginia	67 (62.1-71.7)	83.1 (76.5-88.1)	88.1 (82.5-92.9)	57.1 (51.8-62.2)	80.4 (73.7-85.8)	89.2 (83.1-93.6)
Wisconsin	68.6 (63.8-73.3)	81 (75.1-86.2)	85.5 (79.8-90.5)	53.2 (47.5-58.8)	73.4 (65.8-80.1)	83.3 (73.2-89.2)
Wyoming	64.3 (54.7-72.4)	80.5 (73.6-86.2)	84.7 (77.6-90.9)	49.9 (40.7-59.5)	75.1 (67-81.9)	83.2 (72.5-90.3)

SM Table 4: Prevalence of overweight and obesity by 5-year age group and sex in 1990, 2021 and 2050 at the national level, across 50 states and Washington DC

45-49 years	Male			Female		
Location	1990	2021	2050	1990	2021	2050
USA	67.1 (64.6-69.7)	81.4 (77.2-85)	85.7 (80.9-89.6)	53.6 (50.2-56.9)	75.1 (69.5-80.2)	84 (76.7-88.9)
Alabama	67.7 (61.8-73.3)	83 (77.3-88)	87.4 (81.8-91.7)	53.5 (47.7-59.1)	78.9 (71.6-84.7)	88.8 (82-93)
Alaska	70.2 (63.2-76.8)	80.9 (74.4-86.7)	84.8 (76.9-90.7)	58.2 (49.1-66.5)	74.9 (65.9-82.2)	81.2 (71.2-88.8)
Arizona	58.1 (52.1-63.6)	83.3 (77.4-88.1)	88.3 (81.4-93.3)	49.2 (42.4-56.1)	74.9 (66.4-82.4)	85.3 (73.9-92.2)
Arkansas	68.6 (60.1-76.2)	82.8 (75.5-88.5)	86.8 (79.3-92.3)	56.3 (47.8-65)	78.9 (71.6-85)	88.3 (80.7-93.3)
California	65.1 (58.4-71.1)	78.2 (70.7-84.5)	82 (73.3-89)	46.5 (41.8-51.5)	72.8 (65.4-79.2)	80.7 (70.8-88)
Colorado	64.9 (58.7-71)	76.6 (70.3-82.4)	81.6 (72.8-88)	45.1 (37.8-52)	67.1 (58-74.8)	77.7 (63.1-86.5)
Connecticut	65.6 (60-70.9)	83.2 (77.6-88.1)	87.2 (80.6-92.4)	49.1 (41.5-57.2)	71.4 (62.1-79.6)	81.9 (71-90)
Delaware	69 (60.6-76.8)	82 (74.1-88.2)	86.3 (77.9-91.9)	60.4 (51.7-68)	77.5 (70.2-83.8)	85.9 (77.7-91.3)
District of Columbia	54.6 (47.6-61.5)	72.2 (63.9-79.3)	76.8 (65.6-84.1)	60.1 (51.9-68.1)	70.4 (60.1-79.6)	76.6 (64.3-85)
Florida	63.7 (58.7-68.6)	83.3 (77.4-88)	86.7 (80.1-91.9)	52.8 (43.9-61.5)	71.5 (61.6-80.3)	80.1 (69.1-88.6)
Georgia	68.4 (63.2-73.6)	81.6 (75.3-86.6)	85.8 (78.9-91)	53.4 (47.5-59.1)	76.5 (70.2-82.3)	85.9 (77.2-91.5)
Hawaii	59.9 (53.3-65.7)	78.8 (70.7-85.1)	84.2 (74.6-91)	47.3 (41.2-53.7)	68.1 (58.9-75.6)	77.5 (66.3-86.2)
Idaho	66.2 (61.1-71.2)	80.8 (74.6-86.2)	85.7 (79-91.4)	54.1 (47.8-60.1)	72.7 (64.1-80.4)	81.5 (70.7-88.7)
Illinois	68.6 (63.7-73.2)	80.5 (74.8-85.8)	84.8 (77.8-90.3)	55.7 (50.2-60.8)	74.4 (66.9-80.7)	83.3 (72.7-90.5)
Indiana	70.7 (64.7-76.5)	81.3 (73.4-87.1)	85.8 (78.6-91.7)	61 (55.8-65.8)	77 (70.7-82.9)	86.3 (79.1-91.6)
Iowa	72 (66.5-77.2)	84.8 (79.4-89.4)	89.8 (84.2-93.9)	57.4 (50.3-63.5)	77.1 (70.1-83.5)	87.3 (78.8-92.6)
Kansas	73.8 (67-80.2)	84.2 (78.4-88.5)	89.1 (83-93.3)	55.9 (47.1-64.3)	77.9 (71.2-84.1)	88.2 (79.6-92.9)
Kentucky	70.3 (65-75.5)	82.1 (75.8-87.4)	86.8 (80.7-91.7)	56.4 (50.8-61.8)	78.5 (71.6-84.4)	87.6 (80.3-92.4)
Louisiana	67.9 (60.9-74.1)	83.7 (78.2-88.5)	87.5 (82.1-92.9)	62.5 (55.6-69.1)	80.9 (74.3-86.8)	88.8 (81.5-94.1)
Maine	67.5 (61.7-73.2)	83 (76.8-88.2)	87.3 (80.6-92.2)	54 (45.1-62.3)	74.7 (64.9-82.6)	83.4 (74.2-90.2)
Maryland	66.2 (60.2-71.9)	80.9 (74.8-86.1)	85.2 (78.6-91.2)	55.8 (49.9-61.1)	78.2 (71.1-84.2)	85.7 (77-92.1)
Massachusetts	69.3 (63.8-74.2)	76.9 (70.4-82.5)	80.7 (73.5-87.6)	49 (43.2-54.8)	67.3 (59.7-74.4)	77.1 (66.3-86.1)
Michigan	70.8 (65.5-75.6)	82.5 (77-87.1)	86.3 (80.6-90.9)	58.7 (53.3-64.4)	76.9 (70.3-82.8)	86 (78-91.2)
Minnesota	69 (63.6-74.2)	82.1 (75.6-87.1)	86.1 (78.5-91.8)	51.2 (45.8-56.5)	74.3 (66.8-81.1)	83.1 (73.6-89.9)
Mississippi	67.5 (60.7-73.5)	81.3 (75.2-87)	85.8 (78.9-91.3)	58.5 (51.1-65.2)	83.2 (77.1-88.2)	90.6 (83.7-94.9)

45-49 years	Male			Female		
Location	1990	2021	2050	1990	2021	2050
Missouri	66.3 (61-71.6)	84.4 (79.5-88.8)	88.8 (83.1-93)	54.9 (47.9-61.3)	75.8 (67.6-82.6)	85 (76.2-90.8)
Montana	65.9 (59.6-71.9)	80.3 (73.8-86.1)	84.9 (77.2-91.2)	53 (46.7-58.9)	74.4 (66.7-80.9)	83.5 (73.8-90.4)
Nebraska	69.3 (63.7-74.6)	83.5 (78.3-87.9)	88.8 (82.4-92.9)	57.1 (50.9-62.8)	78.7 (72-84.7)	88.4 (79.5-93)
Nevada	64.1 (55.9-72.2)	82.1 (75.2-87.7)	86.6 (79.4-92.3)	53 (44.8-61.2)	73.9 (65-81.6)	84.1 (73.3-91)
New Hampshire	63.9 (57.6-70)	83.4 (76.6-88.5)	87.5 (80.5-92.9)	49.6 (43-55.9)	70.5 (62.3-78.1)	80.9 (68.4-89)
New Jersey	66.3 (58.7-73.1)	80.6 (74.6-86)	85.2 (77.9-91)	49.7 (41.8-57.3)	69.4 (61.8-76.7)	79 (68.7-87.3)
New Mexico	59.7 (53.5-65.6)	82.1 (76-87.2)	88.2 (81.6-93)	49 (42.7-55.9)	79.5 (72-85.9)	88.7 (79.5-94)
New York	68.8 (64-73.7)	78.8 (72.9-83.6)	83 (76.2-88.9)	54.9 (48.4-61.6)	70.1 (61.6-77.8)	77.6 (68-85.8)
North Carolina	70.2 (65.4-74.6)	82.6 (77.4-87.3)	86.9 (81.2-91.6)	58.1 (52.4-63.1)	77.7 (70.6-83.6)	87.4 (79.2-92.5)
North Dakota	69.1 (63.1-75)	84.9 (77.8-90)	89.2 (82.2-94.1)	54.7 (47.9-60.9)	76.5 (68.2-83.2)	85.4 (75.3-91.7)
Ohio	68.1 (62.5-73)	83.9 (78.8-88.3)	88.5 (83-92.3)	54.7 (49.1-60.4)	77.9 (71.2-83.5)	87.9 (80.3-92.7)
Oklahoma	71.1 (65.3-76.4)	82.4 (76.7-87.2)	87.5 (80.5-92.6)	54.6 (48.1-61)	80.9 (74.1-86.7)	90 (82.4-94.6)
Oregon	64.3 (56.4-72)	79.2 (71.8-85.4)	83.1 (74.4-90)	53.5 (47.8-59.3)	72.6 (65.1-79.3)	81.5 (70.7-88.8)
Pennsylvania	66 (60.3-71.3)	82.8 (77.4-87.6)	86.4 (80.7-91.8)	58.7 (52.3-64.8)	75.8 (69-82)	84.1 (75.6-90.1)
Rhode Island	68.1 (62.8-73.1)	81.4 (74.8-86.5)	85.9 (78.6-91.3)	50.1 (43.8-56.7)	75.4 (67.3-82.3)	85 (74.9-91.8)
South Carolina	67.9 (61.7-73.9)	80.1 (72.9-86.5)	84.8 (77.2-90.7)	57.6 (51.2-64.1)	79.7 (72-86)	88.3 (79.9-93.5)
South Dakota	72.2 (66.5-76.9)	83 (76.4-87.7)	87 (80.1-92.2)	52 (44.4-59.4)	76.8 (67.8-84.2)	85.9 (75.9-92.3)
Tennessee	63.8 (58.7-68.8)	81.1 (75-86.6)	85.7 (79.2-91.7)	54 (47.9-59.7)	79.9 (72.9-86)	88.6 (81.1-93.6)
Texas	65.7 (59.5-71.2)	83.5 (77.9-88.1)	88.1 (81.3-92.9)	53.4 (47.3-59.1)	80.1 (74.1-85)	87.8 (79.6-93.2)
Utah	67.9 (61.7-73.8)	81 (73.7-86.7)	86 (78.2-91.9)	51.2 (44.9-57)	72.1 (64.1-78.9)	81.9 (70.4-89)
Vermont	66.7 (58.8-73.9)	79.1 (71.7-85.2)	83.5 (75.5-90)	54.2 (46.8-61.6)	71.4 (63.6-79.1)	79.2 (68.7-86.7)
Virginia	63.7 (55.4-70.9)	81.8 (74.8-87.6)	86.5 (78.8-92.2)	50.3 (43.6-56.9)	75.8 (68.9-81.8)	85.9 (76.4-92)
Washington	64.1 (58.4-69.5)	80 (73.8-85.4)	83.5 (76.1-90)	52.7 (47.2-58.2)	73.5 (66.8-80)	81 (71.4-88.8)
West Virginia	69.3 (64.1-74.3)	84.4 (78.8-88.8)	89.1 (84.1-93.5)	59.9 (54.2-65.2)	82.7 (75.9-87.8)	90.6 (85.4-94.2)
Wisconsin	75.2 (70.1-79.8)	83.6 (78-88.3)	87.5 (81.7-92)	56.8 (51.5-62.5)	73.3 (65.9-80.2)	83.2 (74-89.5)
Wyoming	70.2 (61.7-77.7)	79.7 (72.6-86.2)	84.1 (75.9-90.8)	52.6 (42.2-62.7)	72.6 (64.6-79.7)	81 (70.2-89)

SM Table 4: Prevalence of overweight and obesity by 5-year age group and sex in 1990, 2021 and 2050 at the national level, across 50 states and Washington DC

50-54 years	Male			Female		
Location	1990	2021	2050	1990	2021	2050
USA	69.4 (66.6-71.9)	82 (78.2-85.3)	86.2 (81.8-90)	58.7 (55.3-61.8)	76.3 (71.2-81)	84.9 (78.3-89.3)
Alabama	72.3 (66.7-77.6)	83.6 (77.3-88.5)	87.9 (82.5-92.3)	57.4 (52-63.3)	80.5 (74.3-85.9)	89.8 (84.6-93.9)
Alaska	70.1 (62.6-76.4)	81.3 (74.7-86.6)	85.1 (77.8-91.1)	61.7 (52.2-70.3)	75.9 (67.3-82.9)	82.1 (73.7-88.6)
Arizona	64.5 (59-69.9)	83.4 (77.9-88)	88.4 (82.1-93.4)	53.9 (47-60.6)	75.3 (66.5-83)	85.6 (75.8-92.1)
Arkansas	70.5 (62.3-78.4)	81.6 (74.3-87.5)	85.9 (78.7-92.1)	60.7 (52.8-68.5)	79.7 (72.3-85.7)	88.7 (81.3-93.6)
California	69.9 (63.5-75.4)	79.9 (72.8-86.1)	83.5 (75.9-90)	58.2 (52.9-62.8)	75 (68.3-81)	82.6 (72.9-89.4)
Colorado	63.5 (56.8-70)	78.4 (72.3-83.6)	83.1 (75.8-89.4)	47.4 (40.1-55.5)	68.9 (60.4-76)	79 (66.3-86.8)
Connecticut	68 (62.1-73.1)	81.7 (75.5-86.8)	86.1 (79.8-91.9)	48.8 (41.2-57.1)	72 (62.9-79.9)	82.2 (70.6-90.4)
Delaware	72.9 (64.3-80.1)	81.6 (74.6-87.5)	86.1 (79.1-91.9)	60 (52-67.2)	77.9 (69.9-84.4)	86.3 (78-92)
District of Columbia	65.1 (58.5-71.5)	73.6 (65.7-80.8)	78.2 (69.4-85)	70.9 (62.9-78.3)	74.6 (65.1-82.5)	80 (69.4-87.8)
Florida	65 (59.7-70.3)	80.3 (74.4-85.5)	84.2 (77.5-90.7)	58.5 (49.6-67.1)	72.8 (62.1-81.7)	81 (70.1-88.8)
Georgia	68.2 (62.5-73.6)	82.2 (75.9-87)	86.4 (80.3-91.5)	53.2 (47-58.6)	79.1 (72.7-84.7)	87.8 (79.9-92.7)
Hawaii	64 (57.2-69.9)	78.5 (70.9-85.2)	84.2 (76.3-90.8)	43.1 (37.1-49.1)	68.8 (60.6-76.1)	78.1 (67.9-86.2)
Idaho	71.7 (66.3-77)	83.4 (77.3-88.4)	87.9 (82.1-92.9)	55.9 (50.1-61.6)	75.4 (67.3-82)	83.7 (75.7-90)
Illinois	69.8 (65.2-74.3)	83.1 (77.5-87.8)	86.8 (80.8-91.9)	58.8 (53.5-63.7)	77.1 (70.5-83.1)	85.3 (76.2-91.5)
Indiana	71.7 (65.4-77.5)	82.8 (76.1-88.5)	87.2 (81.1-92.1)	65.9 (61-70.9)	77.8 (71.3-83.3)	86.8 (79.7-91.8)
Iowa	73.2 (67.2-78.6)	84.6 (79.2-88.9)	89.6 (84.1-93.4)	62.2 (55.9-68.5)	78.4 (71.7-84.2)	88.1 (79.6-93)
Kansas	69.3 (61.5-75.8)	83.7 (78-88)	88.8 (83-93.1)	57.9 (49.2-66.5)	77.7 (71.2-83.7)	88.1 (79.4-93.1)
Kentucky	67.2 (61.5-73)	83.8 (77.5-88.6)	88.1 (82-92.6)	60.1 (54.3-65.5)	79.2 (72.3-84.7)	88.1 (82.5-92.3)
Louisiana	67.4 (60.3-73.7)	81.6 (75.6-86.2)	85.9 (79.5-91.5)	62.6 (55.8-69.7)	80 (73.1-85.7)	88.3 (81.3-93.2)
Maine	68.8 (62.3-74.6)	82.3 (75.5-87.4)	86.8 (81.3-91.7)	58.1 (49.7-66.4)	74.3 (65.3-82.2)	83 (74.5-90.3)
Maryland	67.9 (62.1-73.5)	82.5 (76.8-87.4)	86.5 (80.2-91.7)	57.2 (51.1-63.3)	77 (69.7-83)	84.9 (76.4-91.8)
Massachusetts	69.9 (64.1-75.2)	80 (73.8-85.1)	83.5 (77.4-89.7)	54.4 (48.4-60.6)	69.4 (61.8-76.4)	78.8 (68.3-86.8)
Michigan	72.5 (67.3-77.3)	83.3 (77.9-87.9)	86.9 (81.3-91.7)	64.3 (58.3-69.9)	78.5 (72-84.5)	87 (79.4-92.2)
Minnesota	71.9 (66.6-76.8)	83.5 (76.8-88.4)	87.2 (79.9-92.5)	56.5 (51.6-61.1)	73.2 (65.2-79.7)	82.3 (72.9-89.1)
Mississippi	69 (62.2-74.9)	82.1 (75.7-87.1)	86.3 (79.9-91.8)	62.6 (55.5-69.3)	83.6 (77.5-88.3)	90.8 (84.7-94.7)

50-54 years	Male			Female		
Location	1990	2021	2050	1990	2021	2050
Missouri	71.6 (65.6-76.7)	84.6 (79.5-88.7)	89 (84.2-93.4)	58.8 (52.2-65)	77.6 (69.7-83.8)	86.2 (78.1-91.9)
Montana	70.1 (64.4-75.3)	81.5 (75.1-86.7)	86.1 (78.8-91.5)	55.7 (49.7-61.8)	72.6 (65-79.6)	82.3 (73-89.1)
Nebraska	72.4 (66.8-78)	85.3 (80.1-89.4)	90.2 (84.5-93.9)	58.2 (51.6-64.5)	77.7 (71.6-82.9)	87.8 (78.7-92.4)
Nevada	67.9 (59.5-75.3)	79.9 (72.9-85.6)	84.9 (77.6-90.8)	56.7 (47.8-65.5)	76.6 (68.4-83.3)	85.9 (76.9-92)
New Hampshire	70 (64-75.7)	82.6 (76.4-87.9)	87.1 (79.9-92.2)	52.9 (45.9-60.1)	70.5 (62.4-77.9)	81 (69.5-88.7)
New Jersey	64 (56.2-71.1)	82.4 (76.8-87.1)	86.6 (79.7-91.7)	55.4 (48.2-62.9)	71.4 (63.4-78.2)	80.5 (70.4-88.2)
New Mexico	63 (56.3-69.3)	80.9 (73.9-86.2)	87.2 (79.2-92.2)	53.6 (46.7-60)	79.4 (72.4-85.2)	88.7 (80.3-93.7)
New York	67.3 (61.8-72.2)	81.8 (76.4-86.4)	85.5 (78.9-91.1)	60.4 (53.8-66.4)	74 (65.7-80.8)	80.9 (70.9-88.4)
North Carolina	68.3 (62.7-73)	81.9 (76.1-86.6)	86.5 (80.4-91.2)	59.9 (55-65.2)	77.4 (70.3-83.1)	87.1 (79.3-92)
North Dakota	71.7 (65.4-77.3)	84.6 (76.9-90)	89 (82-94.2)	60.1 (53.9-66.3)	75.5 (67.8-82.1)	84.9 (74.7-91.3)
Ohio	72.8 (67.9-77.5)	83.2 (77.5-87.7)	88.1 (83.1-92.5)	59.6 (54.1-65.1)	79.2 (72.7-84.3)	88.7 (82-93)
Oklahoma	71.3 (64.9-76.8)	84 (78.6-88.8)	88.8 (83.1-93.5)	58.9 (52.2-65.5)	80 (72.9-85.7)	89.4 (81.9-94)
Oregon	70.6 (63.5-77.2)	80.4 (72.7-86.7)	84.2 (75.9-90.6)	57 (50.2-63.1)	73.5 (66.5-79.6)	82.2 (72.4-88.7)
Pennsylvania	71.5 (66.2-76.4)	84 (78.6-88.4)	87.4 (81.8-92.4)	63.5 (57.3-69)	77.4 (70.6-83.2)	85.3 (77.5-90.7)
Rhode Island	72.7 (67.2-77.9)	82.1 (75.5-87)	86.5 (80.2-91.4)	54 (47.8-60.5)	72.1 (64.5-79.9)	82.9 (72.6-89.8)
South Carolina	69.1 (62.8-74.9)	82.4 (75.3-87.9)	86.6 (80.3-91.9)	58.6 (52.8-64.5)	79.6 (71.9-86.1)	88.1 (80.6-93.3)
South Dakota	73.7 (68.1-78.8)	81.9 (75.4-87.4)	86.2 (79.7-91.9)	58 (50-65.6)	77.3 (68.2-84.2)	86.3 (77-92.2)
Tennessee	66.2 (60.8-71)	82.9 (77.2-87.6)	87.1 (81-92.1)	56.3 (50.6-62.3)	77.8 (69.9-84.4)	87.4 (78.3-93.1)
Texas	70.8 (65.1-76.1)	83.2 (77.9-87.9)	87.8 (81.4-92.8)	59.5 (53.7-65)	81.2 (75.2-86.2)	88.6 (81.6-93.4)
Utah	69.8 (63.8-75.6)	81.5 (75-86.9)	86.3 (79.5-92.2)	57.1 (51.3-62.9)	74.2 (66.5-80.6)	83.4 (73.2-89.9)
Vermont	69.1 (61.6-75.9)	80.4 (73.7-86.4)	84.6 (77.3-90.8)	56.7 (49.3-63.7)	69.1 (60.2-76.8)	77.5 (66.8-85.5)
Virginia	69 (60.9-76.3)	82.5 (75.2-88.4)	87.3 (80.2-92.4)	57.7 (51.2-64.6)	77.4 (70.9-83)	86.9 (78.1-92.4)
Washington	70.4 (64.8-75)	80.3 (74.1-85.4)	83.9 (76.8-90.5)	55.4 (49.7-61)	72.4 (65-79)	80 (70.1-87.9)
West Virginia	70.2 (65.1-75.3)	84.4 (79-88.9)	89.2 (84.3-93.7)	61.6 (56.1-66.7)	81.8 (75.2-87)	90.1 (84.7-93.9)
Wisconsin	73.5 (68.4-78)	82 (76-87.1)	86.4 (80.5-91.7)	61.9 (55.9-67.8)	77.3 (70-83.3)	86.1 (78.9-91.2)
Wyoming	73.2 (64.7-80.9)	82.7 (75.6-88.1)	86.6 (79.1-91.8)	56.8 (46.4-66.2)	74.9 (67.1-81.6)	82.9 (72.9-90.5)

SM Table 4: Prevalence of overweight and obesity by 5-year age group and sex in 1990, 2021 and 2050 at the national level, across 50 states and Washington DC

55-59 years	Male			Female		
Location	1990	2021	2050	1990	2021	2050
USA	69.1 (66.4-71.5)	81.1 (77.1-84.6)	85.4 (81.2-89.2)	60.4 (56.9-63.8)	75.7 (70.4-80.6)	84.2 (77.2-89.2)
Alabama	62.6 (56.2-68.9)	80.8 (74.5-86)	85.7 (80.2-91.2)	62.5 (56.8-67.8)	81.2 (74.6-86.5)	90.2 (83.5-93.9)
Alaska	69.1 (60.5-76.6)	79.7 (72.7-85.3)	83.9 (76.9-90.3)	66.4 (57-74.7)	73.1 (64.6-80.3)	79.6 (69.2-87.3)
Arizona	64.9 (59.1-70.2)	80.8 (74.6-85.7)	86.4 (79.8-92.2)	54.9 (48.2-62.2)	73.6 (64.9-81)	84.5 (73.1-91.3)
Arkansas	66.9 (57.9-74.8)	80.7 (73.7-87.2)	85.2 (78.4-91.5)	61.4 (52.9-69.7)	79.7 (72.8-85.6)	88.6 (81.5-93.6)
California	68.6 (61.8-74.4)	77.9 (70.5-84.4)	81.9 (73.7-88.9)	57 (51.7-62.2)	72.9 (65.8-79.1)	80.7 (70.8-87.8)
Colorado	66.4 (59.5-73)	77.6 (71.5-82.9)	82.4 (75.3-89.3)	57.5 (50-64.7)	69.5 (60.7-76.6)	79.4 (66.6-87.4)
Connecticut	68.9 (62.6-74.5)	81.6 (76.1-86.7)	86.2 (79.5-91.6)	51.9 (44.6-59.6)	71.9 (62.4-79.6)	82 (68.4-90.6)
Delaware	71.7 (63.8-78.9)	81.8 (74.1-87.5)	86.1 (79-91.7)	62 (53.6-69.6)	78.3 (71.2-84.8)	86.3 (78.1-92.3)
District of Columbia	64.3 (57.5-70.6)	70.1 (61.2-77.7)	74.9 (65.8-83.4)	71.4 (63.2-78.3)	75.4 (65.7-83.6)	80.6 (70.7-88.3)
Florida	71.3 (66.2-76.1)	78.5 (71.9-83.8)	82.7 (75.7-90)	61 (51.8-69.4)	73.2 (62.4-81.6)	81.2 (70-89.5)
Georgia	67.2 (61.2-73)	80 (73.7-85.3)	84.6 (77.6-90.3)	60.1 (54.7-66)	77.9 (71.6-84.2)	86.9 (79-92)
Hawaii	59.1 (53-65.9)	76.8 (68.5-83.2)	82.8 (73.7-89.5)	45.1 (39-51.6)	62.8 (54-70.7)	73.1 (61.3-83.1)
Idaho	68.6 (62.9-73.9)	81.4 (75.1-86.7)	86.4 (80.1-92)	61.8 (55.5-68.1)	74.6 (66.9-81.8)	83 (74.8-89.6)
Illinois	70.6 (65.6-75)	83.1 (77.4-87.9)	87 (81.8-92.3)	61.1 (55.6-66.4)	77.3 (70.1-83.5)	85.3 (76.5-91.7)
Indiana	71.1 (64.9-77.3)	81.7 (74.7-87.7)	86.2 (79.6-91.7)	61.4 (56-66.6)	77.9 (71.6-83.6)	86.8 (79.4-91.9)
Iowa	72.5 (66.4-77.7)	84.3 (78.9-88.7)	89.4 (84.1-93.4)	63.5 (56.4-69.8)	76.9 (69.7-83.2)	87.2 (78.9-92.4)
Kansas	71.4 (64.2-77.8)	83.4 (78.2-87.7)	88.7 (82.5-92.8)	59.2 (50.4-67.6)	77.3 (70.5-83.3)	87.8 (77.7-92.9)
Kentucky	67.8 (61.6-73.2)	83 (77-88.1)	87.4 (81.7-92.2)	61.2 (55.7-66.6)	78.7 (71.8-84.1)	87.7 (81.3-92.4)
Louisiana	70.5 (62.8-76.7)	82 (76.1-86.8)	86.2 (79.7-91.9)	63.7 (56.2-70.2)	79 (71.8-84.6)	87.5 (79.8-92.9)
Maine	68.2 (61.3-74)	81.7 (75.8-86.6)	86.3 (80.6-91.4)	59.9 (51.9-68)	74.7 (65.7-83.3)	83.2 (74.5-90.5)
Maryland	69.1 (63-74.7)	81.7 (75.4-87)	85.9 (79.3-91.6)	59.4 (53.4-65.7)	77.5 (70.7-83.8)	85.3 (76.2-92.2)
Massachusetts	68.1 (62.6-73.7)	81 (75.6-85.5)	84.2 (78.5-89.6)	55.7 (49.9-61.8)	70 (62.4-76.8)	79.2 (68.4-87.5)
Michigan	72.4 (66.8-77.4)	80.9 (75.4-85.7)	85 (79.5-90.1)	64.3 (58.6-69.9)	78.2 (71.6-83.9)	86.8 (78.7-92)
Minnesota	71 (65.5-76)	82.1 (76-87.4)	86.3 (80.2-91.5)	60.7 (55.5-65.7)	74.2 (67.7-80.3)	82.9 (73.6-89.8)
Mississippi	75.3 (69.3-81.2)	82.1 (76.6-87.1)	86.4 (80.5-92)	66.6 (60.1-73.3)	82.3 (76.2-87.3)	90 (83.4-94.2)

55-59 years	Male			Female		
Location	1990	2021	2050	1990	2021	2050
Missouri	68.9 (62.8-74.2)	83.6 (78.4-87.9)	88.2 (83.4-92.7)	62.8 (56-68.8)	78.6 (70.5-84.9)	86.8 (79.3-91.7)
Montana	68.1 (62.2-73.8)	81 (74.7-86)	85.6 (79-91.3)	59.1 (52.8-65.2)	74.1 (66.4-80.9)	83.3 (73.1-90.2)
Nebraska	73.9 (68.2-79)	84 (78.9-88.3)	89.2 (83.1-93.4)	62.4 (56.1-68.3)	77.7 (71.3-83.3)	87.6 (78.6-92.4)
Nevada	67.8 (59.8-75)	80.7 (73.8-86.4)	85.6 (78.4-91.5)	58.9 (50.5-67.8)	72.9 (64.5-80.3)	83.4 (73.1-90.7)
New Hampshire	70.3 (63.9-75.9)	80.3 (74.7-85.7)	85.2 (78.5-91.3)	56.1 (49.4-62.6)	71.8 (63.8-79.3)	81.8 (70.7-90)
New Jersey	71.9 (64.8-78.7)	81.3 (75.7-86.2)	85.8 (79.7-91)	62.1 (55-69.5)	72.5 (65.1-79.1)	81.2 (71-88.3)
New Mexico	62.2 (55.9-68.1)	80.4 (73.9-85.6)	87 (80.3-92.1)	53.7 (46.6-60.2)	76.5 (68.6-82.9)	86.8 (77.4-92.6)
New York	66 (60.4-70.7)	80.4 (74.9-84.9)	84.5 (78.7-89.6)	57.6 (50.9-63.8)	72.9 (64.7-80.3)	80 (70.1-88)
North Carolina	67.6 (62.1-72.4)	82.2 (76.4-86.7)	86.7 (81.2-91.7)	61.7 (56.1-67)	79.2 (72.5-84.8)	88.1 (80.5-92.8)
North Dakota	72.1 (65.6-78.1)	84.9 (78.2-90)	89.1 (82.2-94)	59.6 (52.6-66.6)	77.3 (68.8-84.1)	85.9 (75.3-91.8)
Ohio	70.4 (64.6-75.6)	81.7 (76.2-86.4)	86.9 (81.1-91.4)	65.2 (59.6-70.8)	78.2 (71.7-83.8)	88 (80.1-92.6)
Oklahoma	68.6 (62-74.4)	83.1 (77-88)	88 (81.4-93.2)	60.1 (53.5-67.1)	79.8 (73.5-85.7)	89.3 (80.9-94.1)
Oregon	69.3 (61.5-76.1)	79.4 (71.4-86)	83.3 (75-89.8)	62.5 (56.3-67.9)	73.4 (65.7-79.7)	82.1 (71.7-89.2)
Pennsylvania	70.8 (65.7-75.6)	83.8 (78.6-88)	87.3 (82.3-92.1)	65.4 (59.4-71)	76.4 (69.5-82.2)	84.4 (76.5-90.4)
Rhode Island	71.7 (66.1-77)	82.6 (76.3-87.8)	87 (80.5-92.2)	55.6 (48.9-61.7)	71.6 (63.6-79.1)	82.3 (71.2-90)
South Carolina	70.6 (64.5-76.2)	80.5 (73.7-86.1)	85.2 (78.9-90.9)	61.5 (54.9-67.3)	78.3 (70.7-84.5)	87.4 (79.3-92.5)
South Dakota	71.4 (65.2-76.6)	83.3 (77.2-88.2)	87.4 (81.1-92.7)	63.1 (55.6-70.1)	75.8 (67.2-83.1)	85.2 (74.4-92.1)
Tennessee	66.1 (60.7-71.5)	81.3 (75.2-86.8)	85.9 (80-91.6)	61.5 (55.8-67.7)	79.5 (72.3-85.9)	88.3 (80.4-93.4)
Texas	67.6 (61.4-73.2)	83.8 (78.7-88.6)	88.2 (82.4-93.2)	60.5 (54.3-66.2)	79.7 (73.2-85.2)	87.4 (79.3-92.7)
Utah	67.9 (61.3-73.8)	80.8 (74.3-86.2)	85.9 (79.1-91.5)	59.1 (52.9-64.8)	74.3 (66-80.9)	83.3 (73.7-90.1)
Vermont	68.9 (61.1-75.8)	79.4 (71.9-86.1)	83.8 (76.5-89.9)	57.6 (50.2-65.2)	68.2 (59.7-75.5)	76.4 (65.8-85)
Virginia	66.1 (58.1-73.4)	81.4 (73.7-87.4)	86.3 (78.2-92.8)	57.2 (50.3-64.2)	77 (70.4-83)	86.6 (76.4-92.4)
Washington	71.4 (65.8-76.6)	79.6 (73.9-84.8)	83.3 (76.4-90.1)	57.6 (51.6-63.7)	72.7 (65.6-79.1)	80.1 (70.6-88.2)
West Virginia	68.7 (63.4-73.5)	82.8 (77.3-87.5)	88 (82.5-92.7)	62.7 (57.4-67.9)	80.4 (73.7-85.9)	89.2 (83.1-93.3)
Wisconsin	75.6 (70.5-80.2)	83.4 (78.2-87.9)	87.4 (81.8-91.7)	62.3 (55.7-68.5)	74.7 (67.4-80.9)	84.1 (74.8-89.7)
Wyoming	72.2 (63.2-79.3)	81.2 (74.6-86.3)	85.4 (78.7-91.1)	61.5 (51.1-71.4)	72.8 (64.5-80.3)	81.2 (70-89.1)

SM Table 4: Prevalence of overweight and obesity by 5-year age group and sex in 1990, 2021 and 2050 at the national level, across 50 states and Washington DC

60-64 years	Male			Female		
Location	1990	2021	2050	1990	2021	2050
USA	67.9 (65.4-70.4)	80.6 (76.3-84.3)	84.9 (79.9-88.9)	61.1 (58-64.3)	76.3 (71.3-80.9)	84.7 (77.4-89.4)
Alabama	66.8 (60.1-72.6)	83.1 (77.2-88.2)	87.5 (81.9-92.3)	58.7 (52.8-64.5)	81.8 (75.6-87.3)	90.5 (84.5-94.5)
Alaska	69 (60.3-76.3)	79.9 (72.4-85.4)	84 (76.6-90.7)	67.8 (58.3-75.6)	73.8 (64.5-80.9)	80.5 (70.5-87.8)
Arizona	58.8 (53-64.5)	80.7 (74.6-85.7)	86.4 (80.1-91.9)	55.4 (48.8-62.2)	73.3 (64.7-80.5)	84.5 (74.2-91.5)
Arkansas	68.7 (59.4-76.8)	81.3 (73.5-87.1)	85.5 (78.4-91.4)	60.3 (51.8-68.3)	80 (73.6-85.7)	88.9 (82-93.8)
California	65.7 (59-72.2)	77.3 (69.8-84.5)	81.4 (72.6-89)	60 (55-64.4)	74.5 (67.9-80.5)	82.2 (72.1-88.8)
Colorado	63.1 (55.6-69.9)	76.2 (69.7-82.5)	81.3 (73.2-88.7)	59 (51-66.6)	69.1 (61.5-76.5)	79.4 (66.5-87.2)
Connecticut	68.2 (61.8-73.8)	79.8 (74-85)	84.7 (77.1-90.5)	60.3 (53.1-67.7)	70.5 (60.9-78.8)	81.1 (68.4-89.6)
Delaware	67.7 (59.5-75.2)	82.3 (74.1-88.3)	86.4 (79-92.2)	63.5 (55.7-71)	78.9 (71.7-85.3)	86.9 (78.4-92.1)
District of Columbia	68.9 (62.2-75.1)	70.1 (61.6-78)	74.9 (64.6-82.5)	70.3 (62.4-77.6)	73.8 (64.1-82.4)	79.5 (68.7-86.6)
Florida	68.2 (62.6-72.9)	78.8 (72.2-84.3)	82.9 (76.3-89.9)	59.4 (50.9-67.6)	73.4 (63.1-82.6)	81.4 (69.7-90)
Georgia	67.7 (61.8-73.6)	79.8 (73.4-85.3)	84.4 (77.8-90.4)	60.7 (55.1-66.2)	77.3 (70-82.9)	86.6 (78.2-91.6)
Hawaii	51.9 (45.1-58.6)	74.4 (66.8-81.6)	80.8 (71.2-88.6)	47 (41.2-53.2)	61.7 (52.6-70)	72.3 (59.6-83)
Idaho	66.5 (61.2-71.6)	82.4 (76.6-87.4)	87 (80.4-92.5)	59.5 (53.2-65.2)	74.4 (66.9-81.2)	83 (73.9-89.6)
Illinois	71.4 (66.3-76.4)	81.5 (75.6-86.4)	85.6 (79.1-91.2)	63.2 (58.1-68.4)	77.9 (71.4-84)	85.8 (76.4-91.6)
Indiana	68.9 (62.2-75.1)	82.3 (75.1-88)	86.7 (79.2-92.1)	60 (55.1-64.9)	79.6 (73.3-84.9)	88 (81-92.5)
Iowa	72.7 (66.9-78.2)	83.7 (78.1-88.3)	89.1 (83.1-93.2)	63.4 (56.8-69.3)	77.8 (70.6-84)	87.9 (79-92.4)
Kansas	64.4 (56.3-71.9)	81.9 (76.9-86.6)	87.5 (80.9-91.9)	58.7 (49.9-67.2)	76.8 (69.5-82.7)	87.6 (77.3-92.8)
Kentucky	63.8 (58.2-68.6)	82 (75.9-87.1)	86.8 (80.6-91.9)	60.3 (54.5-66)	80.1 (73.6-85.4)	88.7 (82.1-92.9)
Louisiana	71.6 (65-77.7)	79.8 (73.2-85.1)	84.3 (77.6-90.5)	67.4 (60.6-74.1)	80.4 (73.9-86)	88.5 (81.1-93.3)
Maine	66.4 (59.6-72.4)	80.4 (73.7-85.5)	85.2 (78.4-90.6)	60 (51.4-67.7)	74.3 (64.7-82.6)	82.9 (74.1-90.1)
Maryland	64.3 (58.1-70)	78 (71.3-84)	82.9 (75.3-89.5)	61 (54.4-67.6)	76.4 (69.2-82.5)	84.4 (75.1-91.7)
Massachusetts	67.4 (61.7-72.8)	78.7 (72.5-83.9)	82.3 (74.6-88)	57.5 (51-63.6)	73.9 (67-80.4)	82.4 (72.8-89.7)
Michigan	75.2 (70-79.6)	81.2 (75.3-86.1)	85.4 (79.1-90.5)	64 (58.1-69.5)	79.7 (73.7-85.1)	87.9 (80.6-92.6)
Minnesota	70.1 (64.3-75.1)	82.6 (75.3-88.1)	86.5 (79-92.2)	60.2 (55.2-65.5)	74.2 (66.6-80.4)	83.1 (72.3-89.7)
Mississippi	68.8 (62.1-75.3)	81.4 (74.9-86.7)	85.8 (79.4-91.3)	64 (56.7-70.4)	82.7 (76.6-87.7)	90.3 (83.9-94.5)

60-64 years	Male			Female		
Location	1990	2021	2050	1990	2021	2050
Missouri	70.1 (64.3-75.5)	81.1 (75.4-86)	86.3 (79.7-90.9)	63.8 (57.1-69.8)	77.2 (69.4-84)	85.9 (77.8-91.9)
Montana	65.9 (60-71.7)	78.9 (72.2-84.8)	83.9 (76.4-90.6)	54.8 (48.5-60.6)	73.4 (65.8-79.7)	82.8 (72.8-89.6)
Nebraska	67.3 (61.1-72.6)	84.1 (78.8-88.7)	89.2 (82.8-93.3)	64.6 (58.8-70.2)	78.5 (71.8-83.9)	88.3 (79.6-92.9)
Nevada	65.8 (56.8-74.2)	78.8 (71.6-84.9)	84 (75.7-90.3)	55.9 (46.9-64.3)	72.6 (64-79.8)	83.4 (72.7-90.5)
New Hampshire	68.1 (61.6-74)	81.1 (74.3-86.4)	85.8 (77.3-91.6)	52.8 (45.9-59.8)	69.9 (61-77.5)	80.7 (68.6-89.2)
New Jersey	69.5 (62.6-76)	78.2 (71.8-83.8)	83.3 (76.6-89.3)	59 (51.1-66.4)	74.1 (66.6-80.8)	82.6 (72.9-89.7)
New Mexico	61.6 (55.3-67.7)	77.3 (70.2-83)	84.7 (75.9-90.5)	57.6 (50.7-64.1)	76.2 (68.7-82.8)	86.7 (76.8-92.4)
New York	70 (65.2-74.6)	80.3 (74.7-84.9)	84.3 (77.7-89.5)	62 (54.9-68.2)	73.2 (65.2-80.4)	80.5 (70.7-88.3)
North Carolina	64 (58.8-69.1)	83 (77.1-87.6)	87.2 (81.6-92.2)	63.8 (58.6-68.4)	79.5 (73.2-85.1)	88.5 (81.2-93.1)
North Dakota	69.9 (63.6-75.9)	84.9 (78-90.1)	89.2 (82.9-94)	67.3 (61.3-72.7)	77.7 (70.1-84)	86.4 (76.6-92)
Ohio	71 (65.8-75.9)	82.6 (77.3-87)	87.6 (82.3-92)	65 (59.9-70.2)	79.4 (72.7-85)	88.9 (82-93.3)
Oklahoma	67.5 (60.4-73.5)	83.1 (77.2-88.1)	88.1 (81.8-93)	58.2 (51.8-64.6)	78.6 (71.6-84.3)	88.7 (79.7-93.9)
Oregon	69.7 (62-76.8)	78.5 (70.5-85.8)	82.5 (74.6-89.3)	60.2 (54.1-65.5)	73.7 (65.9-80.2)	82.4 (72.3-89.3)
Pennsylvania	70.1 (64.8-75.2)	83.1 (77.6-87.3)	86.6 (81.2-91.9)	63.9 (58.2-69.5)	77 (70.6-82.6)	85.1 (77-90.8)
Rhode Island	66.5 (61-71.6)	81 (74.6-86.3)	85.6 (78.3-91.4)	59.5 (54-65.1)	72.9 (64.5-79.8)	83.5 (73.1-90.4)
South Carolina	67.8 (61.3-73.3)	81.3 (74.1-86.9)	85.8 (78.5-90.9)	63.2 (56.8-69.1)	79.5 (71.5-85.8)	88.2 (80.2-92.9)
South Dakota	70.2 (64.3-75.6)	83 (77-87.8)	87.1 (80.4-92.3)	61.9 (53.6-69.6)	79 (70.9-86.1)	87.4 (78-93.5)
Tennessee	66.6 (61.4-71.4)	83.1 (76.8-88.1)	87.3 (81-92.5)	59.9 (53.9-65.7)	79.3 (71.5-85.6)	88.3 (80.3-93.6)
Texas	65.3 (59-71.4)	83 (77.3-87.7)	87.7 (81.2-92.8)	59.4 (53.5-65)	79.7 (73.8-84.8)	87.5 (79.5-92.9)
Utah	66.6 (60.2-72.4)	81.1 (74.3-86.6)	86 (78.8-91.5)	57.1 (51.1-63.4)	73.3 (65.9-80.1)	82.9 (73.1-89.7)
Vermont	67.1 (59.2-74.1)	77.7 (69.9-84.3)	82.3 (74-88.8)	59.9 (52.2-67.4)	67.6 (59.2-74.9)	76.2 (65.6-84.8)
Virginia	60.7 (52.2-68.6)	80.7 (73.4-86.8)	85.9 (78-92.3)	61.5 (54.6-67.9)	78.7 (71.9-84.4)	87.8 (78.7-93.3)
Washington	69 (63.8-74)	79.5 (73.4-84.5)	83.2 (75.8-90)	54.7 (48.6-60.6)	71.7 (64-78.4)	79.6 (69-88.3)
West Virginia	64.2 (58.8-69.4)	84.9 (79.5-89.3)	89.4 (84-93.6)	66.5 (61.5-71.5)	81.4 (75.4-86.6)	89.8 (84.2-93.7)
Wisconsin	69.9 (64.2-74.9)	84 (78.5-88.3)	87.9 (82.3-92.1)	64.1 (58.2-69.8)	77.7 (70.9-83.4)	86.4 (78.5-91.4)
Wyoming	69.3 (59.5-76.7)	81.4 (74.5-86.7)	85.4 (78.8-91.3)	61.9 (52.5-71.4)	72.8 (64.7-79.9)	81.4 (70.4-89)

SM Table 4: Prevalence of overweight and obesity by 5-year age group and sex in 1990, 2021 and 2050 at the national level, across 50 states and Washington DC

65-69 years	Male			Female		
Location	1990	2021	2050	1990	2021	2050
USA	65.4 (62.8-68)	80.5 (76.4-84.3)	84.8 (80.4-88.8)	59.9 (56.7-63.2)	76.3 (71.3-81)	84.7 (77.9-89.2)
Alabama	63.9 (57.8-69.5)	82.7 (76.5-87.8)	87.2 (81.1-92.2)	56.4 (50.8-62.4)	81.4 (75.5-86.4)	90.4 (84.1-94.3)
Alaska	69.8 (61.2-77.1)	78.6 (71.5-84.6)	83 (75.7-89.6)	69.8 (60.8-78.4)	76 (67.6-83)	82.2 (73.6-89.4)
Arizona	59.1 (53.6-65)	79.6 (73.6-84.8)	85.7 (79.3-91.5)	54.5 (47.3-61.2)	74.5 (65.3-82.5)	85.1 (74.1-92.1)
Arkansas	64.5 (55.3-72.8)	81.2 (73.5-87.1)	85.5 (78-91.4)	62.3 (53.6-70.5)	80.1 (73.3-85.9)	89 (82.5-93.7)
California	62.1 (55.1-69)	77.4 (69.8-84.1)	81.5 (73.8-88.9)	53.3 (48.2-58.3)	73.4 (66.6-79.8)	81.3 (71-88.9)
Colorado	56.6 (49.5-64.1)	75.8 (70-81.6)	80.9 (72.5-88)	54.8 (46.5-63.3)	70.4 (62.6-77.6)	80.2 (67.5-88)
Connecticut	67.3 (61.2-72.4)	79.7 (73.9-84.9)	84.7 (78.2-90.9)	54.7 (47.4-62)	73.4 (64.1-81)	83.2 (72-90.9)
Delaware	66.4 (58.2-73.9)	82.8 (75.6-88.9)	86.9 (80.1-92.3)	62.3 (54.1-69.9)	79.1 (72-85)	87.2 (79.3-92.6)
District of Columbia	61.4 (54.3-68.6)	69.5 (60.7-77)	74.4 (65.2-82.9)	62.1 (54.3-69.4)	74 (64.1-82.2)	79.5 (68-87.3)
Florida	66.9 (61.9-71.7)	80.6 (74.5-85.9)	84.5 (77.6-90.9)	57.7 (49.1-66.5)	74.2 (63.4-83)	82.1 (71-90)
Georgia	64.1 (57.8-70)	79.2 (72.5-85)	83.9 (77.3-89.9)	58 (52.1-63.8)	78 (71.2-83.5)	87 (79.2-92.1)
Hawaii	53.1 (46.6-59.9)	72.4 (64.5-79.2)	79.2 (70.7-87.7)	41.6 (35.5-47.8)	63.2 (53.9-71.2)	73.5 (61.1-83.5)
Idaho	66.9 (61.5-72)	81.6 (75.4-87)	86.5 (81-91.7)	59 (53-64.8)	74.9 (66.8-81.6)	83.4 (74.4-89.9)
Illinois	66.9 (61.6-72)	79.9 (73.9-85.2)	84.3 (78.7-90)	61.5 (55.9-67)	78.9 (72-84.4)	86.6 (78.2-92.3)
Indiana	65.8 (59.3-72.2)	82.5 (75.1-88.1)	86.8 (79.7-92)	64.1 (59.2-69.3)	79.3 (73.1-84.6)	87.8 (81.2-92.6)
Iowa	71.2 (65.7-76.9)	84.1 (78.4-88.5)	89.3 (83.9-93.3)	61.4 (55-67.4)	78.1 (71.5-84)	88 (79.8-93)
Kansas	67.5 (59.8-74.2)	83.7 (78.6-88)	88.8 (82.8-93.2)	60.2 (51.5-68.2)	78.2 (71.2-84.3)	88.3 (78.9-93.3)
Kentucky	62.2 (56.7-67.7)	82.8 (76.8-87.7)	87.4 (81.6-92.1)	63.1 (57.9-67.9)	80 (73.5-85.6)	88.7 (82.9-93.2)
Louisiana	60.7 (53.1-68.2)	81.4 (75.5-86.6)	85.8 (79.9-91.6)	66.1 (58.9-73.2)	81.4 (75.2-86.9)	89.2 (83-93.8)
Maine	65 (58.8-71.4)	79.7 (73.8-85)	84.7 (78.4-90.3)	59.1 (50.7-67.1)	74.9 (65.2-83.4)	83.6 (74.4-90.5)
Maryland	64.8 (58.8-70.4)	79.6 (73.2-85.2)	84.1 (76.7-90.5)	62.2 (56.6-67.5)	77.4 (70.7-83.5)	85.2 (76.3-92)
Massachusetts	63 (56.9-68.7)	79.9 (74.5-85.1)	83.3 (77-89.1)	58 (52-63.9)	72.7 (65.4-79)	81.5 (72.2-89)
Michigan	68.2 (62.5-73.9)	81.3 (75.7-86.1)	85.3 (79.8-90.4)	65.6 (59.8-71)	77.8 (71.1-83.3)	86.6 (79.1-91.9)
Minnesota	71 (65.4-76.5)	82.8 (76.3-87.7)	86.7 (79.9-91.9)	62.2 (57.1-67.4)	75.1 (68-81.6)	83.7 (74.5-90.2)
Mississippi	69.3 (61.7-75.7)	84.2 (79-88.7)	88.1 (83-92.8)	64.9 (57.9-71.7)	83 (77.5-88)	90.4 (84.5-94.5)

65-69 years	Male			Female		
Location	1990	2021	2050	1990	2021	2050
Missouri	68.5 (62.7-73.8)	81.5 (75.8-86.3)	86.6 (80.3-91.7)	59.9 (53.3-66)	78.2 (69.5-84.9)	86.7 (78.6-92.1)
Montana	61.4 (55.1-67.8)	80.1 (73.9-85.6)	84.8 (78.1-91)	58.2 (52.5-64.1)	71.9 (63.9-79)	81.7 (70.8-89.1)
Nebraska	64.4 (58.4-70.2)	84.3 (79-88.6)	89.4 (83-93.7)	62.8 (57-68.5)	78.6 (72-83.8)	88.4 (79.7-92.8)
Nevada	63 (54.1-71)	81 (74.5-86.8)	85.8 (78.9-91.4)	57.8 (48.6-66.4)	72.5 (63.9-80)	83.3 (72.9-89.8)
New Hampshire	62.3 (55-69.5)	81.2 (75.1-86.6)	86 (78.5-91.6)	56.7 (49.8-63.8)	71.9 (63.5-78.7)	82.1 (71.4-89.8)
New Jersey	72 (65.7-78.3)	80.9 (74.6-86.1)	85.6 (79.7-91.2)	56.5 (48.8-64.1)	75.2 (67.4-81.8)	83.4 (74.8-90.1)
New Mexico	62.3 (55.7-68.7)	77.1 (70.5-83)	84.5 (76.3-90.5)	52.8 (45.8-59.2)	74.7 (66.8-82.2)	85.8 (75.8-92.2)
New York	66.3 (61-71.3)	78.3 (72.7-83.2)	82.6 (76.3-88.9)	60 (53.3-66.5)	74 (65.5-81.1)	81 (71.2-88.6)
North Carolina	63 (57.8-68)	80.8 (75.1-86)	85.6 (79.8-90.9)	61.4 (56.2-66.5)	77.8 (71-83.6)	87.5 (79.3-92.7)
North Dakota	68.9 (62.8-75)	85.5 (78.9-90.3)	89.6 (83.6-94.2)	61.3 (54.6-67.9)	80.4 (72.5-86.8)	88.1 (79.1-93.9)
Ohio	65.2 (59.8-70.3)	84.4 (79.5-88.7)	89 (83.7-93)	65.2 (60.1-70)	80.6 (74.7-85.7)	89.6 (83.4-93.6)
Oklahoma	60.7 (54.8-66.7)	80.1 (73.6-85.4)	85.9 (78.6-91.5)	57.5 (50.9-64.2)	78.7 (71.5-84.3)	88.8 (80.7-93.9)
Oregon	66 (58.7-73.2)	79.1 (71.6-85.5)	83 (75-90)	57.1 (50.7-62.9)	74.8 (67.9-81.2)	83.2 (73.3-90)
Pennsylvania	68.5 (62.9-73.5)	82.2 (77.4-86.7)	86 (80.6-91.2)	66.8 (61.3-72.3)	78.1 (71.8-83.6)	85.8 (78.6-91.2)
Rhode Island	65.9 (60.4-71.1)	80.9 (74.6-86.2)	85.5 (77.7-91.4)	57.3 (51.3-63.7)	73.2 (65.5-80.5)	83.6 (73.1-90.7)
South Carolina	64 (57.2-70.8)	80.9 (73.9-86.8)	85.5 (78.3-91.2)	60 (53.7-66.1)	78.7 (70.8-84.9)	87.7 (79.5-93.1)
South Dakota	66.1 (60.4-71.6)	80.7 (74.2-86.2)	85.3 (78.5-91.2)	63.8 (55.9-70.7)	80 (72.1-86.9)	88 (79.3-93.6)
Tennessee	59.1 (53.8-64.2)	81.2 (74.9-86.4)	85.9 (79.4-91.6)	59.9 (54-65.3)	79.2 (72.1-85)	88.2 (80.1-93.4)
Texas	64.5 (58-70.2)	81.1 (75.3-86)	86.2 (79.2-92)	60.4 (54.4-66)	76.9 (70-82.7)	85.7 (77.1-91.6)
Utah	64.2 (58-70.2)	80.4 (74-85.8)	85.5 (78.6-91.5)	57.7 (51.6-64.1)	76.2 (69.1-82.3)	84.8 (75.5-91.6)
Vermont	68.2 (60.3-75)	78.4 (71.1-84.6)	82.9 (75.8-89.2)	61.1 (53.5-68.6)	71.6 (64.1-78.7)	79.6 (70.2-87.1)
Virginia	64.9 (56.2-73.1)	80 (72.6-86.3)	85.4 (78-91.8)	58.9 (52.5-65.1)	78 (71.3-83.5)	87.4 (78.2-92.9)
Washington	69.5 (63.9-75.1)	79.2 (73.5-84.4)	82.9 (75.5-89.4)	58.3 (52.1-64)	72.3 (64.9-78.8)	80.2 (70.5-88.2)
West Virginia	60.6 (54.9-65.8)	84.1 (79.1-88.6)	88.9 (84.2-93.4)	61.5 (56.1-66.7)	81.1 (74.5-86.1)	89.7 (84.1-93.8)
Wisconsin	71.2 (66-76.3)	84.3 (79.3-88.4)	88.1 (83.2-92.3)	66.6 (61.2-72.3)	77 (70.1-83.1)	85.9 (77.5-91.2)
Wyoming	68.8 (60.5-77)	79.4 (72.4-85.6)	83.8 (76.3-90.2)	60.2 (50.7-69.3)	75.4 (67.4-82.4)	83.3 (73.8-90.5)

SM Table 4: Prevalence of overweight and obesity by 5-year age group and sex in 1990, 2021 and 2050 at the national level, across 50 states and Washington DC

70-74 years	Male			Female		
Location	1990	2021	2050	1990	2021	2050
USA	59.9 (57.4-62.5)	79.3 (75-83)	83.8 (78.6-88.2)	58.5 (55.3-61.7)	76 (70.5-80.9)	84.3 (76.9-89.4)
Alabama	57.1 (50.2-63.7)	80.3 (74.5-85.4)	85.2 (79.3-90.1)	59.1 (53.5-64.5)	78.5 (71.6-84.3)	88.5 (81.8-93.1)
Alaska	63.3 (54-72)	77.2 (69.7-83.2)	81.6 (72.6-88.8)	64.3 (53.9-73.2)	76.8 (68.7-83.6)	82.7 (72.7-89.8)
Arizona	63.3 (58-68.5)	77.5 (71.8-83.3)	84 (76.1-90.4)	49.2 (42.4-56.3)	72.5 (64.1-80.3)	83.7 (72.4-91.3)
Arkansas	62.2 (53.5-70.1)	81.1 (74.2-87)	85.2 (77.5-91.7)	61.1 (52.7-69.2)	77.9 (71.1-83.6)	87.7 (80.7-93)
California	54 (47.7-61.1)	74.9 (66.6-81.5)	79.2 (69.7-87.4)	53.8 (48.6-59.2)	71.1 (64.1-77.6)	79.3 (68.2-87.1)
Colorado	54.9 (47.1-62.7)	75.3 (69.4-80.9)	80.4 (71.9-87.3)	52.8 (44.7-61)	71.5 (63.5-78.8)	80.9 (67.8-88.6)
Connecticut	60.9 (54.4-67.1)	79.2 (73.4-84.2)	84.1 (77.4-90.4)	58.8 (51.3-65.9)	74.2 (64.9-81.6)	83.6 (72.3-91.4)
Delaware	62.3 (53.2-70.5)	82.1 (74.9-88.1)	86.3 (78.9-91.9)	64.7 (57.1-71.8)	79.2 (72.2-85.2)	87.2 (79.4-92.7)
District of Columbia	57.9 (50.2-65.5)	68.9 (60.2-76.4)	73.6 (63.8-81.4)	60.9 (52.4-68.4)	73.2 (63.2-81.6)	78.8 (66.9-87.3)
Florida	60.1 (54.9-65.1)	80.1 (74.4-85.2)	83.9 (78.1-90.4)	55.6 (46.9-64.3)	74.4 (64.7-82.9)	82.2 (71.6-89.7)
Georgia	55.5 (48.6-62.1)	80.6 (74.2-85.7)	84.9 (77.8-90.8)	57.6 (51.5-63.4)	78.4 (72.3-84.4)	87.2 (78.8-92.3)
Hawaii	44.4 (37.5-51.3)	69.6 (61.4-77.1)	76.6 (66.7-86.2)	38.9 (32.9-45.3)	64 (54.8-71.7)	74 (62.6-83.7)
Idaho	61.3 (55.5-67.1)	81.3 (75.5-86.5)	86.2 (80.1-91.5)	54.4 (48.7-60.8)	73.5 (65.7-80.2)	82.4 (73.3-89.1)
Illinois	62.4 (56.9-67.7)	81.4 (75.6-86.2)	85.5 (78.7-91.2)	59.5 (54.1-64.7)	79 (72.6-84.3)	86.6 (77.8-91.9)
Indiana	62 (54.9-68.6)	81.7 (74.7-87.5)	86.1 (79.7-91.3)	59.8 (54.7-65.2)	79.6 (73.3-84.8)	87.9 (81.2-92.6)
Iowa	66.1 (59.8-72.1)	83.5 (78.2-88)	88.8 (83.2-93.1)	63.2 (57.2-69.4)	79.3 (72.7-84.9)	88.6 (80.6-93.3)
Kansas	58 (49.8-65.8)	81.2 (75.9-85.5)	86.8 (80-91.5)	56.6 (47.9-64.9)	78.1 (71.1-84.3)	88.2 (78.3-93.5)
Kentucky	59.3 (53.3-65.2)	81.2 (75.1-86)	86.1 (79.9-91.8)	59 (53.5-64.3)	77.9 (70.9-84)	87.2 (81.1-92.3)
Louisiana	62.2 (54.3-69.2)	82.4 (76.9-87.1)	86.5 (81-92.1)	65 (57.7-71.8)	79.3 (72.5-85.1)	87.7 (80.1-93.2)
Maine	58.6 (51.5-65.3)	78.8 (72.2-84.2)	83.9 (77-89.5)	55.4 (46.9-63.3)	76.1 (66.6-84)	84.3 (74.4-91.3)
Maryland	56.3 (49.8-63)	77.8 (71.7-83.3)	82.5 (75.1-89.4)	56.2 (49.9-62.2)	77.2 (70.6-83.1)	84.9 (76.6-91.6)
Massachusetts	60.4 (53.7-66.7)	79.7 (74.1-84.6)	83.1 (76.7-89.1)	57.1 (51.1-63.2)	73.3 (66.6-79.7)	81.7 (72.2-88.9)
Michigan	66.2 (60.2-71.9)	84 (79.2-88.2)	87.5 (82.2-91.9)	63.7 (57.9-69.4)	79.6 (73.1-85.3)	87.7 (80.7-92.8)
Minnesota	65.7 (59.8-71.3)	82 (75.7-87.2)	86 (78.6-91.7)	60.6 (55.1-65.5)	76.8 (70.1-82.6)	84.9 (75.4-91.2)
Mississippi	61.2 (53.5-68.2)	79.9 (73.7-85)	84.6 (78.3-90.3)	64.7 (58.1-70.9)	79.3 (72.5-84.8)	88.1 (80-93)

70-74 years	Male			Female		
Location	1990	2021	2050	1990	2021	2050
Missouri	63.9 (57.6-69.8)	80.9 (75.4-85.7)	86.1 (80.3-91.2)	58.8 (52.2-64.8)	77 (69.7-83.5)	85.7 (77.6-91.4)
Montana	58.3 (51.5-64.3)	79.7 (73-85)	84.4 (77.1-90.6)	54.6 (48.3-60.4)	73.5 (66.4-80.1)	82.7 (72.9-90)
Nebraska	65.8 (59.6-71.3)	83 (77.6-87.4)	88.5 (82.5-92.8)	60.5 (54.6-66)	79.8 (73.5-85.2)	88.9 (80.9-93.5)
Nevada	63 (55-70.8)	78.2 (70.7-84.2)	83.4 (75.1-89.7)	52.1 (42.8-61.4)	70.5 (61.9-78.3)	81.7 (70.9-89.3)
New Hampshire	62.5 (55.8-69.3)	80.9 (74.9-86.4)	85.6 (78.4-91.5)	55.9 (48.7-62.6)	71.7 (63.4-78.9)	81.9 (70.1-89.8)
New Jersey	56 (48.2-63.9)	79.6 (73.2-84.9)	84.4 (76.9-90.4)	61 (52.9-68.3)	76.7 (69.4-82.9)	84.3 (75.9-90.8)
New Mexico	56 (49.1-62.8)	73.8 (66.5-79.8)	81.9 (72.4-88.7)	56.2 (49.6-62.5)	73.4 (65.6-80.5)	84.8 (74.4-91.4)
New York	61.6 (56.1-66.7)	75.3 (69.5-80.7)	80 (73.3-87.3)	61.4 (54.8-67.9)	74 (65.8-81.1)	80.8 (71.4-88.7)
North Carolina	56.9 (51.2-62.2)	81.2 (75.5-86.1)	85.8 (79.9-90.9)	59.3 (54.1-64.7)	78.2 (71.5-84)	87.6 (79.4-92.6)
North Dakota	66.5 (60.6-72.4)	83.3 (77.2-88.8)	88 (80.8-93.1)	65.3 (59.3-71.3)	80.1 (72.9-86.1)	87.9 (78.3-93.2)
Ohio	58.7 (52.6-64.6)	82.6 (77.5-87.1)	87.6 (82.7-92.1)	64.6 (58.9-70)	79.6 (73.5-84.8)	88.9 (81-93.1)
Oklahoma	58 (50.5-64.9)	80.1 (73.7-85.4)	85.8 (78.4-91.5)	55.4 (48.4-61.9)	79.1 (72.6-84.9)	88.9 (80.4-93.7)
Oregon	62.3 (55-70.3)	78.6 (71.6-84.9)	82.6 (74.8-89.4)	54.7 (48.8-60.6)	75.1 (68.5-81.1)	83.3 (73.9-89.6)
Pennsylvania	63.5 (57.8-69.2)	77.8 (71.8-83)	82.1 (74.7-88.9)	58.7 (52.7-64.2)	78.8 (72.5-84.3)	86.3 (78.5-91.8)
Rhode Island	63.5 (57-68.9)	80.2 (73.7-85.2)	84.9 (78.8-90.7)	57.9 (52.2-64)	74.2 (66.1-81.2)	84.2 (73.3-91.2)
South Carolina	60.5 (53.8-66.9)	80.2 (73.3-85.8)	84.9 (78-90.7)	58 (51.5-64.1)	77.4 (69.9-84.2)	86.8 (79.1-92.4)
South Dakota	60.6 (54.3-66.8)	82.8 (76.9-87.5)	86.9 (81-92)	56.3 (48.5-64.3)	76.5 (68.3-83.6)	85.6 (74.5-91.9)
Tennessee	58.7 (52.6-64.4)	78.4 (72-83.5)	83.5 (76.5-89.8)	57.5 (51.7-63.2)	77.7 (70.5-83.8)	87.2 (79-92.5)
Texas	59.8 (52.9-66.7)	80.5 (74.6-85.3)	85.7 (78.8-91.5)	58.4 (52.3-64.1)	76.2 (69.7-82.2)	85 (75.6-91.5)
Utah	62 (54.4-68.5)	80.2 (73.6-85.8)	85.2 (78.3-91.1)	56.2 (49.9-62.3)	76.4 (69.6-82.6)	84.9 (76.6-91)
Vermont	58.1 (50-66.7)	76.4 (68.7-82.6)	81.2 (73.4-88.3)	58.3 (50.9-65.4)	69.2 (61.3-76.7)	77.4 (66.3-85.8)
Virginia	57.6 (49.2-66)	79.6 (72.5-86.4)	84.8 (76.6-91.2)	55.8 (48.9-62.5)	77.1 (70.5-83)	86.8 (77.8-92.4)
Washington	55.5 (49-61.5)	79.3 (73.3-84.2)	83 (75.9-89.5)	56.4 (50-62.4)	74.1 (67.5-80.8)	81.4 (71.9-89.2)
West Virginia	60.1 (54.2-65.7)	81.8 (76.5-86.7)	87.2 (81.6-92.3)	62.7 (57.4-67.9)	81.1 (75.4-86.3)	89.6 (83.7-93.9)
Wisconsin	67.2 (61.4-72.7)	83.1 (77.6-87.5)	87.2 (81.7-91.7)	64 (58.2-69.9)	77.8 (71.5-83.5)	86.2 (78.2-91.6)
Wyoming	64.9 (55.5-73.4)	78.7 (70.9-85)	83.1 (75.2-89.3)	62 (51.8-71.6)	74.3 (66.6-81.2)	82.3 (72-89.8)

SM Table 4: Prevalence of overweight and obesity by 5-year age group and sex in 1990, 2021 and 2050 at the national level, across 50 states and Washington DC

75-79 years	Male			Female		
Location	1990	2021	2050	1990	2021	2050
USA	54.5 (51.7-57.3)	77.2 (73.1-81.1)	82.2 (77.3-86.8)	54.7 (51.6-57.9)	73.2 (68-78.2)	82.5 (75-87.9)
Alabama	54 (46.3-61.3)	76.8 (69.6-82.6)	82.6 (75.4-88.6)	57.9 (51.5-63.7)	75.4 (68.6-81.8)	86.8 (79.5-91.7)
Alaska	56.7 (44.5-68.4)	79.3 (72.3-85.4)	83.6 (76-90.3)	61.5 (49.7-71.4)	71.9 (63.2-79.6)	78.7 (68.3-87.5)
Arizona	45.7 (38.9-52.9)	76 (69.6-81.5)	82.7 (74.9-89.6)	45.7 (38.5-53.5)	70.9 (62.2-78.6)	82.8 (71-90.7)
Arkansas	56.7 (46.9-66)	78 (70-84.5)	83 (75-89.7)	54.2 (45.5-63.1)	72.3 (64.7-78.9)	83.9 (74.8-90.4)
California	50.1 (41.9-57.9)	72.7 (64.5-80)	77.5 (68.9-86)	51.3 (45.9-56.6)	71 (64.3-77.3)	79.3 (68.8-86.9)
Colorado	50.8 (42.3-59.5)	75.4 (69.8-81)	80.6 (73.3-87.6)	53.7 (45.8-62.2)	69.3 (61.4-76.7)	79.2 (66.1-87)
Connecticut	53.7 (45.6-61)	78 (71.9-83.6)	83.3 (76.5-89.9)	54.4 (46.6-61.5)	70.3 (61.2-78.2)	80.8 (68.7-89.5)
Delaware	54.6 (45.6-63.4)	80.8 (73.3-86.7)	85.2 (77.4-91.7)	59.7 (52.1-67.5)	76.4 (68.8-83.4)	85.2 (76.4-91.6)
District of Columbia	55.6 (46.7-64.1)	67 (58-74.4)	72 (62.5-80.1)	57.2 (48.7-65.3)	70.2 (59.9-79.8)	76.3 (63.4-84.9)
Florida	53.8 (47.2-59.6)	75.5 (69-81.2)	80.1 (72.2-87.5)	51 (41.8-59.8)	70.9 (60.8-80)	79.4 (68.5-88.6)
Georgia	54.7 (46.7-61.9)	77 (70.7-82.9)	82.1 (75.3-88.2)	57.3 (51.1-63.8)	72 (64.6-78.4)	82.9 (72.7-89.1)
Hawaii	37.3 (30.5-44.9)	67.9 (59.6-75.8)	75.4 (64.4-84.5)	39.7 (32.9-46.5)	60 (51.4-68.4)	70.7 (58-81.5)
Idaho	55 (48.4-61.4)	77.8 (70.8-83.5)	83.5 (76.1-90.3)	51.6 (45.4-57.6)	70.1 (61.5-77.6)	79.7 (69.5-87.3)
Illinois	59.4 (53-65.4)	80.4 (74.7-85.6)	84.7 (77.9-91)	58.9 (53.3-64.5)	78.4 (71.6-84)	86 (76.9-91.6)
Indiana	56.6 (48.4-64.4)	79 (71.5-85.2)	84 (76.8-90.5)	53.2 (47.3-58.9)	75.2 (68.5-81.5)	85.1 (76.8-90.9)
Iowa	62 (55-68.4)	83.7 (78.5-88.3)	89 (83.4-93.1)	59.1 (52.7-65.7)	77 (70.4-83.1)	87.3 (78.7-92.2)
Kansas	56.1 (47.3-64.3)	80.3 (75-85.1)	86.3 (80.3-91.6)	56.9 (48.1-65.1)	73.8 (66.2-80.3)	85.6 (74.9-91.3)
Kentucky	53.5 (46.5-60.6)	77.8 (71.2-83.6)	83.5 (76.2-89.7)	55.7 (49.9-61.4)	74.1 (66.5-80.5)	84.6 (76.7-90.3)
Louisiana	56.2 (47.4-64.9)	77.5 (71.1-83.3)	82.5 (75.9-89.3)	58.3 (50.4-66)	77.4 (70-83.5)	86.4 (78.1-92)
Maine	52.3 (44.5-59.6)	77.2 (70-83.3)	82.7 (75.8-89.1)	55.8 (47-64.8)	73.9 (64.6-82.1)	82.6 (72.6-89.9)
Maryland	55 (47.1-62)	76.1 (69.1-81.8)	81.2 (72.9-88.5)	53 (46.3-59.9)	75.5 (68.1-81.6)	83.7 (74.4-91.3)
Massachusetts	60 (53.2-66.6)	74.3 (68.2-80.1)	78.5 (71.2-85.8)	51.7 (44.8-58.6)	70.4 (63-77.1)	79.6 (68.9-87.7)
Michigan	58.9 (51.3-65.4)	81.6 (76.3-86.3)	85.6 (80.3-91)	62.3 (55.7-68.1)	78 (71.9-84.1)	86.7 (79.1-92)
Minnesota	60.8 (54.8-67)	79.8 (72.9-85.2)	84.3 (76.3-90.5)	57.8 (52.9-63)	73.8 (67.2-80)	82.6 (72.6-89.4)
Mississippi	52 (43.4-59.7)	74.1 (67.2-80)	79.9 (72.5-87.2)	57.7 (50.6-64.9)	75.2 (68.6-81.4)	85.3 (77.2-91.2)

75-79 years	Male			Female		
Location	1990	2021	2050	1990	2021	2050
Missouri	57.3 (50.3-64.1)	75.3 (68.6-81)	81.7 (74.3-88.3)	50.8 (44.4-57.4)	73.6 (66.1-81.1)	83.3 (74.5-89.6)
Montana	53.7 (46-60.7)	78.2 (72-84)	83.2 (76.4-90.1)	52.9 (46.7-59.6)	73.3 (66.4-80.1)	82.6 (72.6-89.5)
Nebraska	63.5 (56.6-69.9)	82.4 (76.9-87)	88.1 (81.9-92.5)	59.5 (53.4-65.9)	75.5 (69-81.6)	86.3 (76.5-91.6)
Nevada	58 (48.5-67)	78.4 (71.2-84.7)	83.8 (76.5-90.1)	49.8 (39.5-59.6)	72.1 (63.7-79.4)	82.8 (72.7-89.9)
New Hampshire	59.5 (51.5-67.3)	80.1 (74-85.7)	85.1 (78-91.3)	52.7 (45.1-60.5)	75 (66.9-82.2)	84.1 (73.2-91.4)
New Jersey	54.4 (45.4-63.2)	78.4 (72.2-83.9)	83.5 (76.2-89.4)	54.9 (46.9-63.1)	73.1 (66-80.1)	81.7 (71.5-88.9)
New Mexico	50.1 (42.3-58)	72.9 (66-79.1)	81.4 (72.9-88.3)	46.9 (39.4-54.5)	69.6 (61.1-76.8)	82.4 (70.9-89.3)
New York	55.8 (49.2-62.2)	75.7 (70.1-80.9)	80.4 (74-87.6)	55.9 (49.4-62.2)	71.9 (64.3-79.2)	79.1 (68.8-87)
North Carolina	53 (46.4-59.2)	76.4 (69.6-82.2)	82 (75.3-88.7)	56.6 (51-62.1)	74.5 (67.6-81.2)	85.3 (76.3-90.8)
North Dakota	63 (55.5-69.7)	80.7 (73.6-86.3)	85.9 (77.8-92.5)	61.6 (55-67.6)	76.6 (68.3-83.3)	85.5 (75.8-91.4)
Ohio	54.1 (47.2-61.2)	79.6 (73.9-84.7)	85.3 (79.1-90.5)	54.6 (48.6-60.6)	76.3 (70-82.2)	86.9 (79.2-91.8)
Oklahoma	51.2 (43.3-58.9)	77.6 (71.1-83.3)	83.9 (77-90.6)	52.8 (45.8-59.5)	74.2 (67.4-80.6)	85.9 (76-92)
Oregon	53 (44.7-61.7)	76.3 (68-83.5)	80.8 (72.5-88.2)	52.2 (46.3-58.2)	70.1 (62.9-77)	79.5 (68.9-87.3)
Pennsylvania	56.2 (49.7-62.9)	81.1 (76-85.8)	85 (79.7-90.9)	57.7 (51.2-63.5)	73.3 (66.2-79.7)	82.1 (73.6-89)
Rhode Island	53.7 (46.9-60.6)	76 (69.7-81.8)	81.7 (73.8-88.3)	57.2 (51.3-63)	73.7 (66.5-80.9)	83.9 (73.6-91.2)
South Carolina	51.8 (43.7-59.6)	77.3 (70.3-83.8)	82.7 (75.3-89.3)	57.4 (50.5-63.6)	73.3 (64.7-81.1)	84 (74.1-90.6)
South Dakota	60.2 (53.4-66.9)	81.3 (75-86.4)	85.7 (78.8-91.6)	57.7 (49.8-65.3)	72.2 (63.1-80.3)	82.5 (71.7-89.6)
Tennessee	54.5 (47.8-61.1)	79.7 (73.3-84.9)	84.7 (77.9-90.7)	53.5 (47.5-59.8)	72.7 (64.6-79.8)	83.9 (74-90.7)
Texas	52.1 (44-59.9)	78.2 (72.1-83.8)	83.9 (76.9-90.4)	51.3 (44.5-58.1)	75 (69-80.9)	84.2 (74.9-90.6)
Utah	53.5 (46-60.3)	77.3 (70-83.9)	83.1 (74.9-90.5)	47.9 (41.3-54.4)	73.1 (65.5-79.9)	82.4 (71.4-89.5)
Vermont	58 (48.9-66.9)	73.3 (65.1-80.5)	78.7 (69-86.5)	54.3 (46.7-62.1)	69.5 (62-76.9)	77.7 (67.7-86.1)
Virginia	53.2 (43.5-63.9)	75.7 (67.2-82.9)	81.9 (73.3-89.7)	55.6 (48.2-63.2)	74 (67.6-80.3)	84.7 (74-91)
Washington	47.7 (40.9-54.5)	77.9 (71.8-83)	81.9 (74.9-89.1)	52.2 (45.7-58.3)	71.2 (64.2-77.6)	79.1 (68.4-87.1)
West Virginia	52 (45.1-58.6)	79 (72.7-84.5)	85.2 (78.7-91)	58.1 (52.1-63.7)	75.4 (68.4-81.9)	86.1 (79-91.6)
Wisconsin	59 (51.9-65.7)	82.1 (76.1-87)	86.6 (81.1-91.4)	59.5 (53.2-65.6)	75.7 (69.1-82.4)	84.9 (76.4-90.6)
Wyoming	57.9 (47.5-67.1)	75.7 (68-82.4)	80.7 (71.7-87.5)	55.5 (45.2-65.5)	68.8 (59.5-76.7)	78 (65-87.2)

SM Table 4: Prevalence of overweight and obesity by 5-year age group and sex in 1990, 2021 and 2050 at the national level, across 50 states and Washington DC

80+ years	Male			Female		
Location	1990	2021	2050	1990	2021	2050
USA	42.2 (39.9-44.5)	67.4 (63.2-71.8)	73.9 (68-79.5)	42.1 (39.6-44.4)	63.6 (57.9-68.9)	75 (65.6-81.5)
Alabama	33.8 (26.1-41.3)	66.9 (59.5-73.7)	74.3 (66.1-83.1)	39.9 (34.2-45.7)	66.5 (58.9-73.2)	80.7 (72.3-87.3)
Alaska	45.5 (33.8-57.3)	71 (63.4-78)	76.7 (66.9-85.4)	39 (29.3-49.3)	62.4 (52.4-71.4)	70.5 (59.4-79.7)
Arizona	39.7 (31.7-47.4)	62.7 (56.2-69.2)	72 (62.1-81.6)	37.5 (30.7-44.6)	62.8 (53.6-71.4)	76.9 (63-86)
Arkansas	40.5 (31.6-50.3)	66 (58.5-73.4)	72.8 (64.1-82.7)	37.2 (29.6-44.8)	62.9 (54.7-70.2)	77.3 (66.6-85.5)
California	38.2 (31.1-45.9)	61.4 (53.8-69.4)	67.3 (57.5-77.7)	38 (32.9-43.3)	59.1 (51.9-66.3)	69.3 (56.9-78.9)
Colorado	36.4 (28.6-45.3)	62.5 (55.6-69.1)	69.4 (59.2-79)	39.9 (32.3-47.8)	57.5 (48.7-65.6)	69.8 (54-79.4)
Connecticut	47.6 (39.6-55.6)	70.7 (64.6-76.7)	77.3 (68.7-85.3)	38.8 (31.1-46.9)	64.2 (55.1-73)	76.2 (62.6-85.8)
Delaware	45.1 (35.9-54.3)	68.5 (58.9-76.6)	74.8 (64.5-83.5)	44.5 (36.6-52.5)	67 (58.2-74.3)	78.3 (67.9-85.8)
District of Columbia	40.7 (31.6-49.5)	61.6 (53.6-69)	67.1 (57.6-75.6)	41.1 (33.2-49.7)	61.8 (50.9-71.4)	69 (56.9-78.6)
Florida	41.5 (36.2-47.3)	65.5 (59.1-72.2)	71.5 (63.3-80.8)	38.2 (30-47.5)	59.3 (48.5-69.7)	69.8 (56.3-81.4)
Georgia	42.9 (34.7-51.6)	67.7 (60.8-74.2)	74.2 (66-82.5)	44.1 (37.4-50.6)	62.9 (55.4-70.3)	76.2 (64.5-83.9)
Hawaii	30.8 (23.6-39.1)	54.3 (45.9-62.3)	63.6 (51.3-75)	39 (31.6-46.4)	49.6 (41.2-57.9)	61.1 (47.5-73.2)
Idaho	44.5 (37.2-51)	67.9 (60.8-74.7)	75.4 (67-83.4)	43.1 (37.4-48.7)	61.9 (53.7-69.8)	72.9 (61.2-82.4)
Illinois	47.6 (41.2-54.4)	70.9 (64.2-77.2)	76.7 (68.2-84.7)	42.3 (36.5-47.8)	66.7 (58.8-73.7)	77.5 (65.8-85.4)
Indiana	43.6 (35.9-51.6)	70.9 (63.2-78.5)	77.3 (68.3-85.5)	44.8 (39.7-50)	67.5 (60.1-74.1)	79.6 (71-86.2)
Iowa	48.5 (41-55.6)	74.6 (68.6-80)	82.3 (75.1-88.3)	44.6 (39-51)	66.8 (59.4-73.8)	80.4 (68.9-87.3)
Kansas	47.3 (38.4-56)	70 (64.1-75.5)	78.3 (69.7-85.6)	37.4 (29.3-45.8)	65.1 (57.4-72.4)	79.8 (68-86.9)
Kentucky	41.5 (34.7-48.5)	68.4 (61.5-74.8)	75.7 (67.2-83.6)	39.1 (33.8-44.6)	64 (56.3-71.3)	77.5 (68.5-84.7)
Louisiana	46.5 (37.2-55.5)	69.2 (62.6-75.4)	75.5 (67.4-83.7)	43.8 (36.1-51.7)	69 (61.4-75.8)	80.6 (70.8-87.7)
Maine	41 (32.8-49.2)	70.5 (63.9-76.6)	77.1 (69.2-83.9)	45 (36.7-53.2)	65.3 (54.6-74.7)	76 (64.5-85.2)
Maryland	44.6 (37-52.4)	68.1 (61.2-74.7)	74.6 (66.1-83.7)	38.8 (32.6-45.5)	66.1 (58.7-73.3)	76.5 (65-86.7)
Massachusetts	45.9 (38.9-53.4)	68.2 (61.8-74.1)	73.1 (65.3-81.3)	39.4 (33.9-45.4)	65.4 (58.3-72.3)	75.6 (64.6-84.5)
Michigan	48.1 (40.6-55.6)	73 (67.3-78.3)	78.5 (71.9-84.7)	46.1 (40-52.5)	67.9 (60.9-74.6)	79.5 (69.8-86.6)
Minnesota	48.8 (42.7-55.3)	75.5 (68.8-81.4)	80.7 (72.4-87.6)	44.2 (39.6-49)	64 (56.1-71)	75.1 (63.2-83.1)
Mississippi	46.6 (37.3-56.3)	69.4 (62.9-75.9)	75.9 (67.7-83.8)	47.8 (40.1-55.4)	64.8 (56.4-72.1)	78 (66.9-85.5)

80+ years	Male			Female		
Location	1990	2021	2050	1990	2021	2050
Missouri	38.7 (31.6-45.9)	65.8 (59.6-71.6)	73.9 (65.6-82.3)	42.2 (36-48.8)	65.7 (57.4-73.6)	77.6 (67.4-85.2)
Montana	43.2 (36.1-50.8)	68.5 (61.8-74.9)	75.2 (66.1-83.6)	42 (35.9-48)	64.4 (57-71.3)	76 (65-84.3)
Nebraska	44.4 (37.6-51.6)	74.7 (68.5-80)	82.3 (74.7-88.5)	42.6 (37.6-48.6)	67.8 (60.7-74.5)	81.3 (69.8-87.9)
Nevada	45.5 (35.4-56.2)	67.6 (60-74.6)	74.8 (66.5-83.6)	38 (29-47.5)	58.3 (49.1-66.8)	72.2 (58.5-81.7)
New Hampshire	48 (39-56.6)	69 (62.4-75.1)	76.2 (66.9-83.8)	42.3 (35.3-49.7)	65.3 (57.1-72.9)	77.1 (64.5-86)
New Jersey	43.2 (34.6-52)	67.3 (60.2-73.5)	74.3 (66.5-82.2)	47.9 (39.7-56.1)	64.6 (56.8-71.6)	75.1 (63.6-83.1)
New Mexico	40.7 (32.2-50.2)	60.1 (53.3-66.7)	71.1 (60.7-80.7)	34.3 (27.4-42)	59.8 (51.3-68.2)	75.3 (61.5-84.5)
New York	39 (32.1-45.7)	65.9 (59.8-71.8)	71.9 (63.8-80.7)	43.5 (37.4-49.8)	60.5 (52-68.6)	69.5 (58.3-79.7)
North Carolina	37 (30.1-44.4)	66.5 (59.8-72.9)	73.7 (65.2-81.9)	39.6 (34.2-45.8)	63.9 (55.9-71.2)	77.8 (66.5-85.1)
North Dakota	53.9 (46.8-60.8)	74.6 (67.4-81)	81.3 (71.6-88.8)	46.3 (40.6-52.5)	67.5 (59.3-75.3)	78.9 (66.4-86.4)
Ohio	44.3 (36.2-52.3)	71 (65.2-76.7)	78.7 (71.5-85.2)	43.4 (37.8-49.1)	66.4 (59.4-73)	80.3 (70.2-86.7)
Oklahoma	40 (32.7-48.6)	69.2 (62.5-75.2)	77.3 (68.7-85.3)	36 (29.8-42.4)	63.7 (56.3-70.7)	78.9 (66.7-86.4)
Oregon	41.1 (33.2-49.5)	69.4 (60.8-76.5)	74.6 (65.6-83.1)	41.6 (35.8-47.5)	61.2 (53.2-68.3)	72.4 (60.3-81.3)
Pennsylvania	44.3 (37.7-51.7)	69.7 (63.3-75.4)	75.4 (68-83.1)	49.9 (43.3-56)	66.8 (59.5-73.7)	77.2 (67.2-84.5)
Rhode Island	41.6 (34.8-48.6)	71.1 (64.5-77.3)	77.5 (69.7-84.9)	43.9 (38.1-50.5)	63.7 (55.3-71.4)	76.6 (64-85.4)
South Carolina	47.2 (39.3-55.4)	68.9 (61.4-76.1)	75.6 (67.7-83.9)	43.2 (36.9-50.2)	65.7 (56.3-74)	78.5 (67.9-86.4)
South Dakota	45.1 (38-52.5)	74 (67.8-79.5)	79.8 (71.2-87)	44.8 (37.5-52.5)	65.7 (56.3-74.5)	77.9 (64.4-86.2)
Tennessee	43.4 (36-50.9)	67.1 (60.3-73.7)	74 (65.4-82.8)	36 (30.9-41.6)	61.6 (53.5-69.6)	75.8 (64.1-84.3)
Texas	37.4 (29.1-45.8)	67.9 (61.7-74.2)	75.6 (66.7-84.1)	42.3 (35.2-49.3)	65.6 (58.2-72.2)	77.4 (65.3-85.7)
Utah	45.2 (36.6-53.9)	67.8 (60.1-74.3)	75.2 (65.6-83.7)	37.8 (31.7-43.6)	61.9 (53.2-69.4)	73.8 (62.1-82.4)
Vermont	48.3 (39.6-57.6)	68 (60.3-75.6)	74.3 (65.5-82.1)	39.8 (33-47.5)	65.3 (57.6-72.8)	74.1 (63.5-82.8)
Virginia	36.2 (26.9-46.4)	67.7 (58.8-75.7)	75.2 (64.7-84.3)	42.7 (35.4-50.5)	66.6 (58.9-73.1)	79.5 (67.6-87.3)
Washington	41.3 (34.4-48.8)	69.1 (62.7-75.1)	74.4 (66-83.1)	41.5 (35.6-48.2)	64.3 (56.9-71.2)	73.4 (61.9-83)
West Virginia	38.9 (32-45.5)	71 (64.7-77.1)	78.9 (70.4-85.9)	43.2 (37.9-48.5)	66.7 (59.1-73.7)	80.1 (71.1-86.7)
Wisconsin	50.5 (42.5-58.7)	71.7 (65.7-77.3)	77.8 (70.1-84.5)	47.8 (41.8-53.9)	67.9 (61-74.3)	79.1 (69.1-85.6)
Wyoming	44 (33.6-54.7)	68.3 (61.2-75.3)	74.5 (65.7-83.5)	43.1 (33.6-53.2)	61.8 (53.2-70.1)	72.4 (59.4-82.7)

SM Table 5: Prevalence of obesity by 5-year age group and sex in 1990, 2021 and 2050 at the national level, across 50 states and Washington DC

2-4 years	Male			Female		
Location	1990	2021	2050	1990	2021	2050
USA	7.2 (3.4-12.6)	6.9 (4.3-10.1)	10.7 (6.2-15.6)	6.8 (3.5-11.6)	8.3 (5-12.6)	13.1 (7.7-19.8)
Alabama	6.4 (1.9-14.7)	7.5 (2.7-15.7)	12.9 (4.9-25.2)	6.6 (2.1-14.2)	11.3 (4.5-22)	19.9 (8-35.2)
Alaska	6.8 (2.1-15.4)	7.5 (2.7-14.6)	12.6 (4.2-24.5)	10.3 (3.6-21.2)	9.6 (3.6-19.1)	14.8 (5.7-28.2)
Arizona	6.5 (1.9-14.1)	6.8 (2.5-14)	11.1 (3.8-21.1)	5.2 (1.5-11.2)	6.6 (2.3-13.8)	12.1 (4.2-23.2)
Arkansas	9.2 (2.8-19.1)	8.1 (3.1-16.2)	11.5 (4.4-21.3)	6.9 (2.3-15.2)	10.4 (4.3-20.7)	18.5 (7.7-35.4)
California	6.8 (2.4-14.8)	5.8 (2.2-11.8)	8.2 (3.3-16.4)	5.3 (1.8-11.3)	6.8 (2.6-14.1)	9.7 (3.6-19.8)
Colorado	6.3 (1.9-14.3)	5 (1.8-10.6)	7 (2.3-14.7)	6.6 (2.1-14.7)	6.5 (2.3-14.1)	9.6 (3.3-19.6)
Connecticut	7.6 (2.6-16.7)	6.5 (2.2-13.4)	9.6 (3.3-19.1)	5.8 (1.9-12.9)	6.3 (2.2-13.9)	9.6 (3.2-20.6)
Delaware	8.7 (2.6-18.9)	7.7 (3-15.4)	11.3 (4.6-21.2)	8.5 (2.9-18.2)	9.4 (3.6-19.2)	15.9 (5.5-30.7)
District of Columbia	5.5 (1.6-12.9)	4.5 (1.6-9.5)	6.8 (2.2-14.7)	8.7 (2.9-18.5)	6.7 (2.2-14.9)	9.3 (2.9-19.6)
Florida	6.6 (2-14.4)	5.9 (2.1-12.2)	8.3 (3.2-15.7)	7.5 (2.6-15.7)	8.2 (3.2-17.1)	12.1 (4.4-24)
Georgia	6.5 (1.9-14.9)	7 (2.7-13.7)	10.8 (3.9-20.3)	6.9 (2.4-15.4)	9.3 (3.5-17.5)	13.7 (5.3-25.4)
Hawaii	9 (3-18.7)	7 (2.5-14.9)	10.7 (4.1-22)	6.8 (2.3-14.2)	7.9 (2.9-17)	11.5 (4.5-22.3)
Idaho	5.5 (1.5-13)	4.8 (1.6-10.5)	8.6 (2.9-18.6)	6.8 (2.3-14.8)	6.6 (2.4-13.3)	11.2 (4-22.4)
Illinois	6.6 (2-14.2)	7 (2.6-14.2)	11.6 (4.4-22.2)	7.1 (2.3-15.2)	8.5 (3.2-16.3)	12.2 (4.1-24)
Indiana	7.6 (2.5-16.2)	8 (3.3-15.9)	13.7 (5.8-26.4)	7 (2.5-15.1)	10.1 (3.9-19.6)	17.5 (7.2-33)
Iowa	8.2 (2.7-17.5)	6.9 (2.5-14.5)	10.9 (3.4-21.2)	6.7 (2.1-15.1)	9.1 (3.3-17.9)	17.2 (5.3-31.2)
Kansas	7.1 (2.3-15.5)	7.9 (2.8-15.7)	13.4 (5-26.4)	9.1 (3-18.4)	9.3 (3.6-18.9)	13.8 (4.8-27.7)
Kentucky	7.4 (2.3-16.1)	8.2 (3.2-15)	12.6 (4.9-23.1)	7.2 (2.4-16.2)	10.7 (4.2-20.5)	19 (7.6-34.4)
Louisiana	6.8 (2.1-15.7)	7.5 (2.7-15.4)	12.2 (4.7-23.9)	10.3 (3.3-20.2)	10 (3.9-19.3)	14.6 (5.6-27)
Maine	8.3 (2.8-18.3)	7.6 (2.8-15.7)	11.8 (4.2-22.7)	8.3 (2.8-17.7)	8.8 (3.3-18.1)	12.7 (5.3-25.1)
Maryland	8.9 (2.7-18.6)	6.8 (2.4-14.6)	10 (3.5-19.8)	7.4 (2.6-15.9)	8.4 (3.2-16.6)	12.1 (4.4-23)
Massachusetts	7.4 (2.2-16.1)	6.4 (2.3-13)	8.5 (3-17)	5.3 (1.8-12.4)	7.1 (2.6-14.8)	11.1 (3.8-22.6)
Michigan	8.3 (2.7-18)	7.2 (2.7-14.2)	10.6 (3.7-20.7)	7.7 (2.6-16.2)	9.7 (3.8-19.5)	15.2 (6.4-29.6)
Minnesota	7.3 (2.2-15.6)	7 (2.7-14)	11.8 (4.2-23.5)	5 (1.6-11.6)	7.5 (2.8-15.7)	13.4 (4.6-25.2)
Mississippi	8.5 (2.7-17.8)	11.1 (4.7-20.4)	17.2 (7.5-30.8)	8.8 (2.9-19)	12.3 (4.9-23.4)	20.7 (8.7-35.4)

2-4 years	Male			Female		
Location	1990	2021	2050	1990	2021	2050
Missouri	6.7 (2.2-15.2)	7.9 (3.1-15.5)	13.2 (5.3-24.5)	7.2 (2.3-15.9)	9.4 (3.5-18.3)	14.8 (5.9-27.1)
Montana	7.5 (2.5-16.2)	6 (2.1-12.5)	9.5 (3.2-19.4)	5.9 (1.7-12.6)	6.7 (2.3-14.3)	12.3 (3.8-24.5)
Nebraska	6.9 (2.1-14.8)	7.5 (2.7-15.2)	10.7 (3.8-20.6)	5.7 (1.8-12.2)	7.3 (2.6-15.3)	12.7 (4.6-25.9)
Nevada	9.3 (3.1-19.2)	6.7 (2.4-13.3)	10.5 (3.6-19.4)	6.4 (2.1-14.4)	5.7 (2-11.9)	9.2 (3.1-19)
New Hampshire	7.9 (2.4-16.6)	5.2 (1.7-12.1)	8.9 (2.6-19.5)	6.4 (1.9-14)	6.2 (2.2-12.9)	9.7 (3.3-19.5)
New Jersey	7.2 (2.2-15.7)	6.8 (2.5-13.2)	10 (4-18.5)	5.8 (1.9-13.4)	6.5 (2.3-13.4)	9.7 (3.3-19.1)
New Mexico	7.1 (2.1-15.9)	5.7 (2.1-12.2)	10.2 (3.1-21.9)	5.9 (1.9-12.7)	6.3 (2.3-13.9)	10.7 (3.5-23.2)
New York	6.8 (2-15.3)	6.5 (2.4-13)	10.4 (3.7-19.4)	6.6 (2.3-13.7)	7.7 (3-16.3)	11.6 (4.2-24)
North Carolina	7.3 (2.3-15.6)	6.6 (2.5-13.1)	9.1 (3.4-17.5)	8.2 (2.8-17.3)	9 (3.4-18.1)	11.7 (4.7-22.9)
North Dakota	6 (1.9-13.7)	8.5 (3.3-16.7)	13.7 (4.9-26.7)	6.1 (1.9-12.9)	7.1 (2.4-15)	12.8 (4.8-24.8)
Ohio	8.4 (2.5-18)	7.7 (3-15.5)	11.1 (4.4-21)	7.5 (2.5-16.3)	9.1 (3.4-18)	15.2 (6.2-27.2)
Oklahoma	6.9 (2-15.9)	8.9 (3.4-17.2)	16.2 (5.7-30.3)	7.6 (2.4-16.5)	11.5 (5-22.6)	21.1 (8.2-37.8)
Oregon	7.1 (2-16.5)	6.7 (2.5-13.3)	11.7 (4.3-22.6)	8.9 (2.9-18.6)	7.2 (2.6-14.8)	9.4 (3.3-19.6)
Pennsylvania	7 (2.2-15)	7.4 (2.9-15)	10.8 (4.2-20.7)	7.7 (2.7-16.7)	8.1 (3.2-16.6)	11.6 (4.6-22)
Rhode Island	5.8 (1.7-13)	6.8 (2.5-13.8)	11.3 (4.1-20.9)	5.8 (1.6-13.1)	6.2 (2.2-13.6)	9.6 (3.6-18.9)
South Carolina	8.3 (2.7-17.5)	8.1 (3.2-16.2)	12.2 (4.8-23.6)	8.6 (2.9-18.6)	11.2 (4.7-21.8)	16.5 (7.3-28.6)
South Dakota	6.2 (1.8-13.8)	8.9 (3.4-17.3)	13.2 (5.5-23.6)	6.3 (2-14.6)	8 (3-16.7)	11.7 (4.7-22.7)
Tennessee	5.9 (1.9-12.9)	7.2 (2.6-13.8)	11.9 (4.5-22.6)	7.2 (2.3-15.8)	10.2 (4.2-19.3)	17.8 (7.1-32.2)
Texas	7.1 (2.3-16)	7.2 (2.8-14.5)	11.2 (4.6-22.5)	5.9 (1.8-13.5)	9 (3.5-17.1)	14.1 (5.9-25.2)
Utah	5.4 (1.7-12.4)	5.9 (2.2-12)	10.2 (3.7-20.4)	5.5 (1.7-12.2)	5.9 (2-12.5)	11.5 (3.4-23.5)
Vermont	8.8 (3.1-17.8)	7.3 (2.8-14.5)	10.3 (4-19.4)	7.8 (2.4-16.8)	7.9 (3-16.1)	12.3 (4.5-25.5)
Virginia	7.5 (2.5-17)	6.9 (2.5-14.4)	10.6 (3.6-22.9)	6.5 (2.2-13.9)	9.2 (3.4-17.7)	14.4 (5.2-28)
Washington	7.1 (2.1-16.1)	7 (2.7-14.1)	10.1 (4-19.3)	7.2 (2.3-15.7)	6.7 (2.4-13.9)	8.9 (3.2-18)
West Virginia	9.8 (3.4-19.5)	9.6 (3.6-18.1)	15.3 (6.2-28.2)	9.1 (3.3-18.9)	10.5 (3.8-20.1)	15.1 (5.6-28.8)
Wisconsin	7.1 (2.2-16)	7.2 (2.9-14.1)	10.8 (4.1-20.4)	7.1 (2.2-14.9)	8.3 (2.9-16.8)	15.9 (5.5-32.5)
Wyoming	9.4 (2.9-20.1)	7.5 (2.8-14.6)	11.5 (4.5-21.2)	8.5 (2.9-18.8)	7.2 (2.4-14.4)	10.1 (3.8-19.4)

SM Table 5: Prevalence of obesity by 5-year age group and sex in 1990, 2021 and 2050 at the national level, across 50 states and Washington DC

5-9 years	Male			Female		
Location	1990	2021	2050	1990	2021	2050
USA	10.3 (5.4-16.4)	13.9 (9.9-18.3)	20 (13.4-26.9)	10.5 (5.4-17.2)	15.2 (10.2-21)	22.3 (14.4-30.6)
Alabama	9 (2.8-17.9)	14.8 (7.2-25.8)	23.3 (11.6-37.1)	10.2 (3.6-20.1)	20 (9.6-33.8)	31.7 (16-50)
Alaska	9.9 (3.4-21.1)	15.1 (7-26.6)	23 (10.2-39.2)	15.6 (5.5-29.8)	17.6 (7.4-31.2)	24.4 (10-41.8)
Arizona	9.4 (3.3-19.2)	13.8 (5.9-25.1)	21.4 (9.8-37.8)	8.2 (2.6-17.1)	12.8 (5.1-23.4)	21.6 (8.3-39.3)
Arkansas	13.3 (4.7-26.3)	15.8 (7.2-26.3)	21.1 (10.5-35.1)	10.8 (3.5-21.6)	18.4 (8.4-31.8)	29.6 (13-50.1)
California	9.9 (3.8-19)	12.2 (6.1-20.9)	16.6 (8.8-28.5)	8.3 (2.8-17.1)	12.8 (5.9-23.4)	17.2 (8.3-30.1)
Colorado	9.3 (3.2-19.7)	10.4 (4.6-19.9)	14.3 (6.7-25.6)	10.1 (3.5-20.3)	12.2 (4.9-22.5)	17 (7.1-31.7)
Connecticut	10.8 (3.9-21.9)	12.8 (5.6-22.9)	17.8 (7.9-30.1)	9.2 (3-18.8)	11.9 (4.6-22.6)	16.6 (6.7-30.6)
Delaware	12.6 (4.7-24.4)	15 (7-26)	20.8 (9.6-34.6)	12.7 (4.8-23.8)	17 (7.2-31.1)	26.4 (11.1-46.2)
District of Columbia	8.4 (2.6-17.3)	9.8 (4-19.2)	14.2 (5.8-25.8)	13.4 (4.9-25.6)	12.6 (5.5-23.4)	16.4 (6.1-29.5)
Florida	9.8 (3.6-19.2)	12 (5.4-21.1)	16.2 (7.4-27.8)	11.9 (4.5-22.5)	15.5 (7.4-27.3)	21.6 (9.4-37.2)
Georgia	9.5 (3.2-19.7)	14 (6.4-24)	20.3 (10.1-34.1)	10.8 (3.9-21.6)	16.5 (7.8-28)	23.1 (10.1-37.9)
Hawaii	12.6 (4.6-23.5)	13.8 (5.8-24.2)	19.7 (8.7-32.9)	10.9 (3.4-21.8)	14.8 (6.1-26.4)	20.4 (9.4-34.8)
Idaho	8 (2.6-17.1)	10 (4.1-18)	16.3 (6.5-28.6)	10.6 (3.8-21)	12.5 (5.1-23.6)	19.4 (7.6-36)
Illinois	9.4 (3.2-18.8)	13.7 (6.3-23.8)	20.9 (9.4-33.8)	11.1 (3.9-21.7)	15.5 (6.8-28.3)	21.2 (9.5-36.6)
Indiana	10.8 (3.8-20.9)	16 (7.7-26.6)	24.3 (11.4-38.8)	11 (4-21.2)	18.3 (8.6-32.3)	28.5 (15.7-46.6)
Iowa	11.5 (4.4-21.7)	13.5 (5.8-23.8)	20.1 (8.4-35.4)	10.5 (3.5-21.2)	16.5 (7.3-28.5)	28.4 (11.9-47)
Kansas	10.5 (3.6-21.5)	15.9 (7-27.9)	24.7 (12.2-41.1)	13.7 (5.3-26.5)	16.5 (7.1-29.6)	23.1 (9.5-40.3)
Kentucky	10.9 (3.9-22)	16 (7.5-28.9)	23.3 (11.5-39.5)	11.1 (3.7-21.4)	19 (9-33)	29.9 (14.7-47.9)
Louisiana	10 (3.3-20.5)	14.8 (7.1-26.1)	21.9 (10.5-35.7)	15.3 (6.2-29.4)	18.6 (8.6-32.3)	25.5 (11.5-42.2)
Maine	11.9 (4.3-23.3)	15.4 (7-26.8)	22.2 (10.1-37.7)	12.7 (4.9-25.3)	16 (6.8-28.1)	21.9 (9.4-37)
Maryland	12.5 (4.4-23.8)	13.6 (6.2-24.8)	19 (8.6-33.3)	11.3 (3.8-21.9)	15.2 (6.8-27.2)	20.9 (9.4-38.3)
Massachusetts	10.6 (3.6-23)	12.9 (5.9-23.5)	16.3 (7.5-28.2)	8.4 (3-17.9)	13.2 (5.7-23.8)	19.2 (7.8-36.2)
Michigan	11.9 (4.4-23.6)	14.4 (6.6-24.9)	20.1 (8.8-32.6)	12 (4.5-23.3)	17.3 (8.1-29.8)	25.3 (11.1-41.7)
Minnesota	10.4 (3.9-19.7)	14 (6.2-24.7)	21.8 (9-37.3)	7.9 (2.7-17)	13.9 (6.1-25.4)	22.9 (10-39.6)
Mississippi	12.4 (4.5-24.5)	20.7 (10.5-34.3)	29.2 (15.5-45.7)	13.4 (5-26.6)	21.7 (10.7-34.8)	33 (17.6-50.4)

5-9 years	Male			Female		
Location	1990	2021	2050	1990	2021	2050
Missouri	9.7 (3.3-18.6)	15.8 (7.4-27)	23.8 (12.5-37.9)	11.2 (4.1-22.8)	16.8 (7.5-30.7)	24.5 (10.8-42.6)
Montana	10.9 (4-22.1)	12.2 (5.1-22.3)	18.1 (7.7-31.4)	9 (3-18.9)	12.4 (5.2-23.9)	20.9 (8.3-37.9)
Nebraska	10.1 (3.5-21.3)	15.2 (6.9-26.4)	20.4 (9.5-34.7)	8.8 (3-18.3)	13.6 (5.9-24.7)	21.3 (8.1-38.2)
Nevada	13 (5.2-24)	13.4 (6.1-23.4)	19.6 (8.7-33.1)	10.3 (3.5-21.3)	11.2 (4.1-22.4)	16.6 (6.1-32.1)
New Hampshire	11.3 (4.4-22.2)	11 (4.3-20.9)	17.5 (6.6-32.2)	9.6 (3.1-19.9)	11.3 (4.6-21.5)	16.6 (6.7-29.7)
New Jersey	10.4 (3.5-21.2)	13.8 (6.4-25)	19.1 (8.8-34.6)	9.1 (3.1-18.9)	12.2 (5-24)	17.4 (6.9-32.9)
New Mexico	10 (3.3-19.5)	12.1 (5.3-21.6)	19.3 (7-34.9)	9.4 (3.1-20.4)	12 (4.8-22.1)	18.6 (6.9-35)
New York	9.8 (3.7-19.8)	13 (6-21.9)	19.1 (9-32.8)	10.4 (3.7-21.4)	14.4 (6.5-26.2)	20.2 (8.5-36.3)
North Carolina	10.7 (3.5-20.2)	13.8 (6-24.1)	18 (8.4-30)	12.3 (4.4-24)	16.5 (7.5-28.7)	20.3 (9.1-35.7)
North Dakota	9 (3.1-18.9)	16.4 (7.5-28.1)	24.1 (10.5-39.5)	9.4 (3.3-19.6)	13.5 (5.3-25.9)	21.7 (7.9-39.4)
Ohio	11.8 (4.6-21.9)	15.2 (7.2-26.4)	20.7 (9.3-34.1)	11.5 (4.5-22.3)	16.5 (7.7-27.5)	25.4 (12.9-39.5)
Oklahoma	9.9 (3.5-20.9)	17.3 (8-28.7)	27.9 (12.6-44.2)	11.5 (4.4-22.7)	20.6 (9.9-35.2)	33.5 (15.2-53.3)
Oregon	10.2 (3.7-19.9)	13.7 (6.2-24.4)	21.5 (8.7-36.8)	13.2 (5.1-25.2)	13.4 (5.9-25.4)	16.9 (6.9-30.4)
Pennsylvania	10.2 (3.6-21)	14.2 (6.5-24.5)	19.7 (9.7-32.2)	11.8 (4.8-23.6)	14.6 (6.5-26.4)	19.9 (9.8-34.3)
Rhode Island	8.8 (2.9-18.5)	14 (6.3-25)	21.2 (9.4-38.1)	9 (3-18.7)	11.7 (4.8-22.1)	17.3 (6.4-32)
South Carolina	11.6 (4.4-22)	16.3 (7.6-27.6)	23.3 (12.2-36.6)	12.7 (4.7-23.6)	19.8 (9.4-32.7)	27.3 (13.9-43)
South Dakota	9.2 (3.2-18.9)	16.6 (8.7-27.6)	22.7 (12.4-36)	9.5 (3.1-19.5)	14.6 (6-26.9)	19.8 (7.5-34.3)
Tennessee	8.7 (2.8-18.3)	14.5 (6.1-25.4)	22 (9-36.6)	10.8 (4.1-20.7)	18.2 (8.1-30.5)	28.4 (12.7-45.9)
Texas	10.3 (3.9-19.5)	14.9 (7-25.7)	21.4 (10.6-35.4)	9.3 (3.3-18.4)	16.3 (7.6-27.8)	24.1 (12-39.7)
Utah	8.1 (2.7-16.9)	11.9 (5.3-22.2)	18.9 (7.6-32.7)	8.6 (3.2-18.2)	10.9 (4.3-20.9)	18.7 (7.2-34.3)
Vermont	12.6 (4.3-23.6)	14.5 (6.5-25.5)	19.3 (8.6-33.2)	11.8 (4.3-23.1)	14.4 (6.1-26.6)	20.7 (8.4-37.6)
Virginia	10.5 (3.6-20.9)	14 (6.3-24.3)	20.2 (8.4-33.8)	10.2 (3.6-20.6)	16.4 (7.5-28.5)	23.9 (10.2-40.5)
Washington	10.1 (3.6-20.6)	14.1 (6.5-24.2)	19 (9.1-32)	10.9 (3.8-21.7)	12.6 (5.1-23.6)	16.1 (6.3-29)
West Virginia	13.8 (5.4-25.5)	18.9 (9.8-31.1)	27.4 (15.6-41.5)	13.8 (5.5-25.4)	18.5 (8.6-31.8)	25.1 (11.1-42.4)
Wisconsin	10.2 (3.6-19.9)	14.3 (6.8-24.8)	20.1 (9.7-33.6)	11.2 (4.3-22.2)	15.2 (6.6-26.5)	26.3 (9.9-45.3)
Wyoming	13.5 (5.4-25.7)	15 (6.5-26.5)	21.6 (10.3-35.5)	13.1 (4.8-27)	13.9 (5.8-26.2)	18.5 (7.5-33.6)

SM Table 5: Prevalence of obesity by 5-year age group and sex in 1990, 2021 and 2050 at the national level, across 50 states and Washington DC

10-14 years	Male			Female		
Location	1990	2021	2050	1990	2021	2050
USA	7.7 (4.8-11.5)	18.1 (13.3-23.4)	25.3 (17.7-32.3)	6.9 (4-10.7)	16.3 (11.3-22.8)	23.1 (15.4-31.5)
Alabama	6.8 (2.3-13.9)	19.3 (8.9-32.7)	29.3 (13.7-45.8)	6.9 (2.5-14.1)	21.9 (10.2-36.2)	34.1 (17-51.8)
Alaska	7.2 (2.5-15.3)	19.3 (9-33.1)	28.7 (12.4-47)	10.4 (3.9-19.6)	18.6 (8.6-31.8)	25.4 (12.2-42.6)
Arizona	7.1 (2.7-14.5)	18.4 (8.5-32.1)	26.3 (12.8-42.8)	5.2 (1.9-11.1)	13.1 (5.4-24.7)	21 (8.4-37.5)
Arkansas	10 (3.7-19.8)	20.7 (9.5-35.1)	27 (13.5-43.6)	7 (2.5-14.7)	20 (9.4-34.8)	32.2 (15.9-53)
California	7.3 (2.8-14.7)	15.8 (7.2-26.4)	21.1 (10.3-34.2)	5.3 (1.9-10.9)	14 (6.2-24.9)	18.7 (8.4-31.9)
Colorado	6.8 (2.5-13.6)	14 (6.2-25.2)	18.3 (8-31.3)	6.7 (2.4-14.1)	13.1 (5.6-25.4)	17.6 (7.5-32.1)
Connecticut	8 (2.9-15.5)	17 (8-29.3)	23.1 (10.5-38.6)	5.8 (2-12.2)	12.5 (5.2-24.2)	17 (7.1-30.7)
Delaware	9.5 (3.6-18)	19.9 (9.9-33.8)	27 (13.9-42.9)	8.7 (3.3-17.5)	17.9 (8.1-31.2)	25.9 (11.5-43.1)
District of Columbia	6.2 (2.1-13.3)	12.8 (5.4-23.2)	18.6 (7.3-32.8)	8.9 (3.3-17.7)	13 (4.9-23.9)	16.8 (5.6-32.6)
Florida	7.4 (2.9-15.1)	16.2 (7.3-27.9)	21.1 (8.9-35.5)	7.9 (2.8-16.2)	16.2 (7.4-28.2)	21.8 (10.1-36.2)
Georgia	6.9 (2.6-14.3)	18.1 (8.8-30.6)	25.7 (12.4-42.1)	7 (2.4-14.9)	17.7 (7.9-32.1)	24.5 (11.2-42)
Hawaii	9.4 (3.8-18.2)	18 (8.4-30.6)	25.2 (12.5-40.6)	7.2 (2.5-14.9)	15.7 (6.9-28.2)	21.2 (9.9-35.4)
Idaho	5.9 (2.2-12.8)	13.5 (5.7-25.6)	21.7 (9-37.6)	6.8 (2.4-14.4)	13.2 (5-25.3)	20.1 (7.6-37.3)
Illinois	7.1 (2.6-13.4)	18.2 (8.4-31.1)	26.5 (11.6-42)	7.3 (2.7-14.8)	16.6 (7.7-28.7)	22 (10.1-35.5)
Indiana	8.1 (3.1-16.6)	20.6 (9.9-34.6)	30.2 (15.2-46.6)	7.2 (2.6-14.3)	20 (9.3-34.7)	30.7 (14.8-51.6)
Iowa	8.9 (3.7-17.5)	17.1 (7.5-30.2)	24.7 (9.6-42.1)	6.9 (2.4-14.7)	17.5 (8-31.2)	28.9 (12.7-49.7)
Kansas	7.7 (2.8-15.3)	20.5 (9.6-33.4)	30.3 (13.9-47.7)	9.4 (3.4-18.6)	17.4 (8-30.4)	23 (11.7-39.3)
Kentucky	8.1 (3-16.2)	21 (10.4-34.5)	29.4 (14.6-46.4)	7.4 (2.6-15.4)	20.5 (9.2-34.3)	31.3 (15.9-49.5)
Louisiana	7.4 (2.7-15)	19.1 (9.5-32.5)	28 (14.4-45.2)	10.4 (4.1-20.7)	19.5 (8.5-33.9)	26.2 (11.8-43.1)
Maine	9.1 (3.6-17.8)	19.8 (9.4-32.3)	28.2 (14-45.5)	8.4 (3.2-16.6)	17.2 (7.3-29.5)	23.1 (10.2-38.2)
Maryland	9.7 (3.6-18.8)	17.7 (7.8-30.5)	24.1 (10.3-39.7)	7.4 (2.7-14.4)	16.1 (7.2-29.3)	21.6 (10-38.8)
Massachusetts	7.8 (2.9-15.4)	17.1 (8.1-29.9)	21.2 (9.4-37.5)	5.1 (1.7-11.2)	14.3 (5.8-26.6)	20.1 (8.5-34.9)
Michigan	9.1 (3.5-17.6)	18.8 (8.8-32.3)	25.3 (12.7-41)	7.8 (2.9-16.4)	18.3 (8.1-32.7)	25.8 (12-43)
Minnesota	8 (3.1-15.6)	18.3 (8.5-31.5)	27.5 (12.8-45)	5 (1.7-11.4)	14.6 (6.3-26.4)	24 (10.4-40.8)
Mississippi	9.2 (3.5-18.4)	26.8 (14.7-40.7)	37.2 (21.1-52.8)	9.1 (3.3-18.5)	23.5 (11.5-38.8)	34.5 (17.6-51.5)

10-14 years	Male			Female		
Location	1990	2021	2050	1990	2021	2050
Missouri	7.3 (2.9-14.5)	20.3 (9.5-33.3)	29.9 (15.6-46.8)	7.4 (2.5-15)	17.9 (8.3-31.3)	25.7 (12.4-42.9)
Montana	8 (3-16)	15.8 (7.1-28.2)	22.7 (10.2-39.8)	5.7 (1.9-11.7)	12.7 (4.9-24.7)	21.1 (7.2-39.2)
Nebraska	7.5 (2.8-15.3)	19.5 (9-33)	25.1 (11.6-40.5)	5.8 (2-12.8)	14.3 (5.7-26.3)	21.9 (10.1-38.3)
Nevada	9.9 (4-18.6)	17.9 (8.7-29.7)	25.7 (11.7-42.5)	6.5 (2.3-14.3)	11.8 (5-22.3)	17 (7-31.8)
New Hampshire	8.4 (3.1-16.3)	14.2 (5.8-25.6)	21.7 (8.2-36.6)	6.2 (2.3-13.3)	12.3 (4.9-24.1)	17.7 (6.9-33.8)
New Jersey	7.8 (3.1-15.3)	18 (8.6-30.3)	24.1 (11.3-39.2)	6.1 (2.1-12.8)	13.3 (5.6-25.1)	18.7 (7.7-35.7)
New Mexico	7.6 (2.9-15.5)	15.6 (6.6-28.1)	24.3 (9.6-42)	6.2 (2.2-13.4)	12.7 (5.1-24.2)	19.4 (7.5-36.8)
New York	7.2 (2.8-13.9)	17.3 (8.3-29)	24.3 (11.9-40.1)	6.7 (2.4-13.8)	15.3 (6.4-27.3)	20.2 (9.1-35.1)
North Carolina	8.1 (3-15.6)	17.5 (8.1-29.2)	22.1 (10.7-35.8)	8.3 (3.3-17)	17.9 (8.5-29.8)	21.8 (10.7-35.8)
North Dakota	6.7 (2.4-13.9)	21 (9.8-34.1)	30 (15.1-46.5)	6.1 (2-12.2)	13.6 (5.6-25.8)	20.5 (8-36.8)
Ohio	8.8 (3.7-16.9)	19.8 (9.1-32.7)	26.5 (13.3-41.3)	7.6 (2.8-16)	17.6 (8-30.6)	26.3 (12.7-43.6)
Oklahoma	7.2 (2.7-14.8)	21.9 (10.8-35.8)	34.6 (16.9-51.5)	7.8 (2.8-15.6)	22.1 (10.7-36.9)	34.1 (16.3-53.2)
Oregon	7.6 (2.7-15)	18.1 (8.1-30.7)	27.8 (11.1-44.4)	8.9 (3.6-18)	14 (5.6-25.8)	17 (7.2-30.7)
Pennsylvania	7.7 (2.9-15.1)	19 (9.2-31.2)	25.4 (12.5-39.8)	8 (2.9-16.4)	16 (7-28.1)	21.2 (9.2-35.7)
Rhode Island	6.5 (2.4-12.9)	18.7 (8.1-31.4)	27.5 (12.6-43)	5.8 (1.9-11.7)	12.4 (4.9-23.9)	17.7 (7.3-33.5)
South Carolina	8.9 (3.3-17)	21 (10.3-33.9)	28.6 (14.6-44.4)	8.6 (3.2-17.6)	21.4 (10.8-36.8)	29 (15.9-46.7)
South Dakota	6.6 (2.5-13.7)	22.5 (12.1-36.3)	30.3 (17.7-46)	6.2 (2.1-13.7)	15.3 (6.7-29.1)	19.9 (8.6-35.6)
Tennessee	6.4 (2.4-13)	18.5 (9.2-31)	27.1 (13.3-44.2)	7.2 (2.6-14.6)	19.8 (8.9-33.9)	30.3 (14.1-50.3)
Texas	7.7 (2.8-14.9)	19.2 (9.6-33.2)	26.3 (13.8-42)	6 (2.1-12.6)	17.2 (7.7-29.9)	24.4 (11.4-41.3)
Utah	6 (2.1-12.3)	15.9 (7.2-26.8)	24.5 (10.7-39.6)	5.6 (1.9-11.8)	11.7 (4.6-21.9)	19.7 (8-34.8)
Vermont	9.5 (3.7-18)	19.1 (9.1-32.9)	25.1 (11.9-42.5)	8 (3-16.3)	15.1 (6.2-28.4)	20.9 (8.5-37.1)
Virginia	8 (3.2-15.9)	18.3 (8.6-30.7)	25.6 (11.7-42.7)	6.7 (2.1-14.2)	17.6 (7.9-30.9)	24.9 (11.4-43.9)
Washington	7.6 (2.8-14.9)	18.4 (8.9-32.5)	24 (12.5-39.1)	7.2 (2.7-14.9)	13 (5.4-24.3)	16.1 (7.5-30)
West Virginia	10.7 (4.3-20.7)	24 (11.8-37.9)	33.7 (17.4-51)	9.3 (3.5-18.2)	20.2 (9.1-33.8)	26.4 (12.6-43.7)
Wisconsin	7.8 (2.8-15.9)	18.8 (8.6-32.1)	25.4 (11.4-41.6)	7.5 (2.7-16)	16.3 (7-30.1)	26.8 (10.5-45.8)
Wyoming	10 (3.9-19.1)	19.3 (9.3-32.2)	26.9 (13.4-42.5)	8.8 (3.2-17.9)	14.1 (5.8-25.2)	18.2 (8.3-30.8)

SM Table 5: Prevalence of obesity by 5-year age group and sex in 1990, 2021 and 2050 at the national level, across 50 states and Washington DC

15-19 years	Male			Female		
Location	1990	2021	2050	1990	2021	2050
USA	7.7 (6.7-8.8)	18.6 (15.7-21.7)	26 (19.8-31.4)	8.3 (6.8-9.9)	24.6 (20.2-29)	33.5 (25.6-40.2)
Alabama	5.8 (3.7-8.5)	19.3 (13.5-25.9)	29.1 (20.1-38.8)	7.6 (4.9-11)	36.8 (28.1-45.4)	51.2 (38.1-61.1)
Alaska	7.6 (4.2-12.2)	20.7 (14.3-27.9)	30.5 (18.2-41.7)	13.7 (8.4-20.5)	28.6 (19.9-37.6)	37 (24.3-48.5)
Arizona	7 (4.4-10.5)	19.8 (14.3-25.6)	28.6 (18.7-37.8)	6 (3.1-10)	23.3 (16.3-31.7)	34.8 (22-48.3)
Arkansas	10 (6-14.9)	21.7 (15-29)	28.3 (19.1-37.5)	6.9 (3.8-11)	34.1 (24.9-44)	47.9 (33.2-61)
California	8.6 (6.1-11.5)	16.2 (11.9-21.5)	21.7 (15.6-29.4)	5.8 (3.9-8.2)	21.6 (16.2-27.7)	27.9 (20.4-38.8)
Colorado	6.8 (4-10.2)	12.5 (8.9-17)	16.9 (11.1-23.8)	8.1 (4.9-12.6)	17.4 (12.3-23.4)	23.6 (15.1-31.9)
Connecticut	7.7 (5.1-11.1)	16 (11.2-21.4)	21.8 (14.6-30.1)	6.4 (3.3-10.5)	17.8 (11.7-24.5)	24.1 (14.9-35.6)
Delaware	9.5 (5.7-14.7)	17 (11.3-23.8)	23.7 (15.4-32.8)	10.3 (6.6-15.6)	27.3 (19.3-36.1)	37.6 (25.1-50.6)
District of Columbia	6.5 (3.9-10.4)	12.1 (6.8-18.8)	17.9 (8.7-28.5)	13.1 (8-19.2)	20.9 (12.3-31.2)	26.1 (13.7-40.3)
Florida	6.5 (4.4-9.5)	15.6 (11-20.8)	20.6 (13.9-28.6)	9.7 (6.2-13.9)	24.8 (17.7-33.2)	32 (21-43.2)
Georgia	7.8 (5.2-11.3)	18.6 (13.9-24.7)	26.3 (17.4-34.9)	9.1 (6-12.9)	25.9 (18.7-33.7)	34 (23.7-45.1)
Hawaii	10.7 (7.2-15.1)	21.9 (16.1-28.7)	29.6 (22.2-37.9)	9.8 (6.4-14.1)	22.5 (15.9-30.3)	29.6 (20.2-39.9)
Idaho	4.7 (3-7)	15.9 (11.3-20.9)	24.6 (15-33.8)	8 (5-11.9)	24.9 (17.7-32.5)	35.1 (22.3-46.3)
Illinois	7.6 (5.2-10.8)	18.7 (13.6-24.4)	27.6 (17.4-37.5)	9 (6.1-12.4)	22.9 (16.1-30.7)	29.7 (19.7-40.9)
Indiana	8.3 (5.7-11.8)	23 (16.8-29.8)	33.6 (23.1-44.5)	9.3 (5.9-13.8)	29.7 (22.3-37.5)	42.5 (30.5-54.1)
Iowa	8.5 (5.8-12.1)	18.1 (13.1-23.2)	26.2 (16.7-35.2)	7.1 (4.3-10.7)	27.3 (20.8-34.3)	41.8 (27.6-53.4)
Kansas	5.1 (2.8-8.3)	18.8 (14.3-23.9)	28.6 (18.7-37.8)	11.4 (6.4-17.6)	26.3 (19.8-33.7)	34.3 (24.3-44)
Kentucky	8.9 (6.2-12)	21.5 (15.9-27.4)	30.2 (21.2-38.7)	8.4 (5.4-12.2)	31.4 (23.5-40.1)	44.7 (32-56.3)
Louisiana	7.9 (4.9-11.9)	20.6 (14.9-26.9)	29.4 (19.3-39.1)	15.1 (10.2-21.1)	27.6 (19.9-36.2)	35.9 (26.2-46.1)
Maine	9.2 (5.7-13.3)	19.7 (14.2-26.3)	27.7 (18.5-38.1)	10.6 (6.2-16.8)	22.6 (16-30.4)	29.8 (21-39.9)
Maryland	9.9 (6.6-13.5)	17.3 (12.7-23.1)	23.8 (15.4-32.6)	10 (6.5-14.5)	23.2 (17.4-29.8)	30.1 (20.6-39.4)
Massachusetts	7.7 (4.8-11.2)	14.9 (10.8-19.5)	18.8 (12.5-25.4)	5.6 (3-9.1)	22.3 (16.1-29.7)	30.3 (19.1-40.9)
Michigan	8.3 (5.4-11.7)	18.8 (13.7-24)	25.8 (17.7-35.2)	9.1 (6.1-13)	26.8 (19.9-34.1)	36.6 (25.9-46.5)
Minnesota	7.7 (5.5-10)	19.6 (14.8-25.3)	28.9 (18.8-38.7)	5.4 (3.6-7.6)	21.5 (15.9-28.1)	32.8 (21.1-43)
Mississippi	8.1 (4.8-12.1)	22.6 (16.2-29.6)	32.9 (23.4-42.6)	9.8 (6-14.8)	36.1 (27.8-44.5)	48.7 (36.4-59.4)

15-19 years	Male			Female		
Location	1990	2021	2050	1990	2021	2050
Missouri	7.5 (4.9-10.8)	22.9 (17.3-28.6)	33.3 (24.2-42.3)	8.8 (5.4-12.9)	26.2 (18.9-33.7)	35.9 (24.9-46.7)
Montana	9.2 (5.5-13.5)	18 (13-24.2)	25.5 (16.4-36)	6.2 (3.1-10.6)	24.5 (17.9-32.3)	36.7 (22.4-48.9)
Nebraska	7.6 (4.2-11.7)	19 (14.3-24.3)	25.1 (18.4-32.4)	6.4 (3.5-10.5)	24.6 (19.1-31.1)	35.5 (23.7-46.4)
Nevada	11.3 (7-16.9)	20.7 (14.7-27.5)	28.4 (17.7-39.2)	7.7 (3.9-12.5)	20.2 (13.3-28)	28.2 (16.6-39.4)
New Hampshire	8.2 (5.2-12.1)	17.7 (11.9-24.1)	26.5 (14.1-38.1)	6.3 (3.1-10.4)	17.9 (11.3-25.1)	24.9 (14.6-35.2)
New Jersey	6.5 (3.9-9.9)	17.7 (12.7-23.4)	23.8 (15.4-32.6)	5.9 (3.2-9.7)	19.3 (13.5-26.9)	26.5 (17-37)
New Mexico	6.6 (4.2-9.5)	21.4 (15.5-28.9)	31.7 (19.5-43.4)	6.9 (3.8-11.3)	22.5 (15.8-29.8)	32.1 (19.7-43.7)
New York	7 (4.7-9.8)	18.1 (13.9-23.2)	25.7 (16.6-34.3)	8.5 (5.6-12.4)	22.4 (16.9-28.8)	29.7 (19.4-39.9)
North Carolina	10 (7.1-13.4)	15.4 (10.8-20.5)	19.9 (14.2-26.6)	12.8 (8.9-17)	23.7 (17-31.2)	28.7 (20.5-37.7)
North Dakota	5.2 (3.2-7.9)	20.4 (14.6-27.7)	29.3 (17.9-41.4)	6.5 (3.9-10.2)	19.6 (13.2-27.8)	29.1 (16.3-41.2)
Ohio	10 (6.8-13.5)	18.4 (13.6-23.7)	24.7 (18.1-31.9)	10.4 (6.9-14.8)	26 (19.5-33.5)	37 (28.4-47.3)
Oklahoma	6.3 (3.8-9.6)	26 (19.8-32.9)	39.1 (25.4-50.4)	9 (5.6-13.5)	34.2 (26.2-42.6)	48.8 (34.5-60.5)
Oregon	7.4 (4.5-10.9)	20.4 (14.8-26.9)	30.5 (19.2-40.9)	10.3 (6.8-14.7)	19.2 (13.2-26.8)	23.1 (14.9-32.4)
Pennsylvania	6.2 (3.8-9.2)	16.9 (12.5-21.5)	23.2 (16.2-30.5)	9 (6-12.7)	20.3 (14.9-26.2)	26.8 (19.3-35.8)
Rhode Island	7.3 (4.6-10.4)	20.3 (14.7-27.3)	29 (18.1-39.9)	6.7 (4.1-9.9)	17.9 (11.5-25.4)	25.2 (13.6-37.4)
South Carolina	8.4 (5.9-11.5)	22.6 (16.5-29.5)	30.7 (22.3-39.6)	10.6 (7.3-14.7)	26.8 (19.6-34.7)	35.1 (25.4-45)
South Dakota	6.5 (4.1-9.8)	18.4 (13.1-24.1)	25 (17.7-33.5)	6.1 (3.3-9.8)	19.4 (12.9-27.6)	24.8 (16.1-34.8)
Tennessee	5.1 (3.3-7.3)	19.1 (13.9-25.1)	28.3 (19-39.4)	9 (6.1-12.5)	30.3 (22.5-38.2)	42.8 (29-55.2)
Texas	6.6 (4.5-9.1)	21.7 (16.7-27)	29.5 (21.3-37.9)	6.6 (4.1-9.8)	29.2 (22.8-36.2)	39.1 (29.1-48.3)
Utah	4.5 (2.7-6.9)	16.6 (12-21.9)	25.1 (14.8-34.8)	7 (4.3-10.3)	19.4 (14.2-25.6)	31 (18.1-42.1)
Vermont	7.8 (4.7-11.7)	18.7 (13-24.9)	24.6 (15.5-34)	9.2 (5.4-14.4)	20.5 (13.8-27.8)	28 (16.6-38.9)
Virginia	7.7 (4.8-11.3)	18.3 (12.9-24.5)	25.9 (16.7-34.8)	6.4 (3.6-10.1)	25.8 (19.3-32.9)	35.1 (24.1-46.8)
Washington	5.3 (3.2-7.9)	20.1 (15.2-25.3)	26.4 (19.7-34.8)	9 (6-13.1)	19.4 (14.2-25.7)	23.9 (17.6-32.7)
West Virginia	12.5 (9-16.5)	21 (14.8-27.5)	30.3 (21.1-40.6)	11.5 (8.1-15.6)	24.8 (17.9-32.5)	31.8 (20.9-43.5)
Wisconsin	9.5 (6.3-13.3)	18.1 (13-23.8)	25.1 (16.4-34.3)	9.7 (6-14.5)	26.8 (19-35.2)	40.7 (24.7-54.1)
Wyoming	7.7 (4.1-12.4)	17.1 (11.6-24.1)	24.2 (16.3-33.2)	8.4 (4.4-14)	19.3 (11.9-28.2)	24.7 (15.2-36.6)

SM Table 5: Prevalence of obesity by 5-year age group and sex in 1990, 2021 and 2050 at the national level, across 50 states and Washington DC

20-24 years	Male			Female		
Location	1990	2021	2050	1990	2021	2050
USA	10 (8.8-11.3)	27.1 (23.3-30.9)	35.6 (28.7-41.5)	12 (10.3-13.9)	33.3 (28-38.9)	42.8 (34.5-51)
Alabama	8.8 (6-12.1)	32.8 (25.4-40.2)	44.1 (32.8-54.9)	13.4 (10.1-17)	39.3 (31.1-47.6)	53.3 (41.4-63.4)
Alaska	10.1 (6.4-15.3)	29.3 (21.9-37.2)	40.7 (26.9-52.2)	17 (11.2-23.6)	34.6 (25.9-43.9)	43.6 (29.9-55)
Arizona	9.8 (7.4-12.9)	26.8 (20.9-33)	36.9 (26.1-46)	10.1 (7.1-13.7)	35.4 (27.2-43.5)	48.3 (33.3-60.9)
Arkansas	13.2 (8.8-18.8)	29.7 (22.8-37.6)	37.3 (27.8-47.4)	16 (11.3-21.8)	44.5 (35.9-52.7)	58.1 (44.5-70.4)
California	9 (6.9-11.2)	27.1 (21.3-33.1)	33.9 (26-43.2)	10 (7.9-12.6)	26.5 (20.6-33.4)	33.3 (25-43.6)
Colorado	5 (3-7.5)	20.3 (15.5-25.6)	25.6 (17.3-33.1)	10.1 (6.9-14.4)	26.9 (20.2-34.2)	34.3 (24.1-43.7)
Connecticut	9.3 (6.7-12.5)	26.1 (20.4-32.1)	33.3 (24.4-42.2)	8.5 (5.6-12.4)	31.9 (24.1-40.5)	39.9 (29.2-51.9)
Delaware	12.2 (8.1-17.4)	23.9 (17.5-31.2)	32.1 (22.5-41.5)	14.6 (10.4-19.6)	36.3 (27.8-45.3)	47.2 (33.7-59.9)
District of Columbia	7.5 (5-10.7)	20.7 (14.5-27.4)	28.1 (17.2-38.4)	11.5 (8-16.1)	28.6 (20.7-37.7)	34.7 (22.6-49.2)
Florida	8.6 (6.3-11.1)	23.4 (18.2-28.6)	29.3 (21.1-37.3)	11.8 (8.1-16.6)	28.7 (21-36.7)	35.8 (25.6-45.9)
Georgia	8.1 (5.9-10.8)	26.5 (20.7-33)	35.7 (25-45.8)	13.9 (10.8-17.7)	34.3 (27.4-41.8)	42.8 (32.7-54.1)
Hawaii	12.2 (9.1-15.8)	28.2 (21.1-35.5)	36.6 (27.6-47)	10 (7.3-13.3)	29.6 (22-37.8)	36.9 (27.4-46.3)
Idaho	8.9 (6.3-11.6)	24.7 (19-30.7)	35.6 (24.2-46.2)	13 (9.6-16.9)	32.8 (24.8-41.6)	43.3 (28.9-56)
Illinois	10 (7.4-12.8)	25.6 (20-31.4)	35.5 (24.2-45.1)	10.9 (8.1-14)	32.8 (25.8-40.4)	40.5 (29.8-51)
Indiana	11 (8.1-14.1)	29.7 (23.3-36.6)	41.2 (30.4-51.6)	13.3 (10.4-16.5)	41.3 (34-49)	54.5 (42.6-65)
Iowa	13.6 (10.1-17.4)	29.1 (23-35.5)	38.5 (26.8-49.3)	14.7 (11.1-18.8)	41.4 (34.1-48.9)	55.9 (42.2-67.1)
Kansas	11.5 (7.7-16.2)	32.6 (26.6-38.5)	44 (32.7-53.8)	15.9 (11.3-21.5)	37.7 (30.6-45.4)	45.7 (34.4-54.8)
Kentucky	11.3 (8.6-14.6)	30.1 (23.9-36.8)	39.9 (30-49.3)	16.6 (13.3-20.3)	41.8 (33.9-49.5)	54.9 (43.6-64.4)
Louisiana	9.9 (6.9-13.7)	32.5 (25.9-39.8)	43.3 (32.4-54)	14.2 (10.1-18.8)	42.4 (34.3-50)	51 (40.8-60.3)
Maine	9 (6.1-12.6)	26.4 (20.3-33)	36.1 (25.5-46.3)	15.5 (11-20.3)	33.2 (24.9-41.9)	41.2 (30-52.5)
Maryland	11.1 (8.2-14.5)	26.5 (21.1-32.1)	34.1 (24.9-42.8)	12 (9.1-15.4)	32.5 (25.5-39.9)	39.9 (29.5-49.2)
Massachusetts	8.7 (6.3-11.7)	21.1 (16.1-26.1)	25.9 (18.3-33.2)	9.3 (6.6-12.5)	22.3 (17-28.4)	30.4 (20.3-41.5)
Michigan	13.1 (10.2-16.4)	27.4 (22-33.8)	35 (25.9-43.2)	13.1 (10.1-16.5)	37.3 (30.3-44.9)	47.6 (36.9-57.1)
Minnesota	7.5 (5.5-10.1)	26.5 (20.8-32.7)	37.5 (26.3-47.3)	8.8 (6.7-11.2)	31.4 (24.8-38.7)	44.1 (31-54.3)
Mississippi	12.4 (8.8-16.6)	34.9 (28-41.9)	47.2 (35.9-56.9)	17.8 (13.2-22.8)	46 (37.9-53.8)	58.5 (46.5-68.2)

20-24 years	Male			Female		
Location	1990	2021	2050	1990	2021	2050
Missouri	11 (8.1-14.2)	27.6 (21.8-33.4)	38.7 (28.8-47.7)	11.8 (8.7-15.4)	39.2 (31.6-47.6)	49.4 (38.6-59)
Montana	9 (6.3-12.4)	23.4 (17.5-29.6)	32 (21.6-42.6)	12.2 (8.8-16.1)	28.1 (21-35.5)	40.5 (25.7-53.8)
Nebraska	10.6 (7.5-14.4)	26.7 (21.1-32.3)	33.4 (26.2-41.4)	8.5 (5.8-12.1)	35 (28.2-42.4)	46.5 (34.1-56.7)
Nevada	10.5 (6.3-15.6)	24.6 (17.8-32.3)	33.6 (22.4-45.9)	14.1 (8.9-20.4)	30.6 (22.7-39.4)	39.7 (26.6-51.2)
New Hampshire	10.3 (7-13.9)	27.1 (20.6-34.4)	37.5 (24.4-49.2)	11.6 (8-16)	30.8 (23.2-38.9)	39.3 (26.8-50.5)
New Jersey	9.2 (5.5-13.6)	22.2 (16.5-28.1)	28.8 (20-37.8)	8.8 (5.8-12.8)	26.9 (20.1-33.8)	35 (24.6-45.6)
New Mexico	7.7 (5.2-10.5)	29.9 (23.8-36.8)	41 (27.6-52.8)	13.6 (9.5-18.2)	35.6 (27.1-44.1)	46 (31.1-58.9)
New York	9.2 (6.9-11.7)	25.7 (20.2-31.1)	34.2 (23.7-44.1)	11 (8.1-14.6)	31.3 (24.4-39.1)	39.2 (28.2-51.2)
North Carolina	10.5 (8.1-13.4)	28.4 (22.4-34.9)	34.2 (27-41.5)	14.4 (11.3-18)	36.4 (28.9-44.6)	41.9 (33.4-50.9)
North Dakota	10.1 (7.2-13.5)	28.4 (22.1-35.7)	39 (26.4-50.8)	11.6 (8.7-15.3)	35.7 (28.2-44.2)	47.8 (33-59.4)
Ohio	13.1 (10.2-16.5)	27.4 (21.8-33.4)	34.4 (26.6-42.1)	12.3 (9.4-15.8)	39.3 (32-47)	50.6 (41.1-59.9)
Oklahoma	11.1 (7.8-15.4)	33.5 (26.7-40.3)	47.2 (34.2-57.2)	10.9 (7.6-14.7)	44.6 (36.3-52.8)	58.9 (45.7-69.5)
Oregon	9.6 (6.7-13.1)	23.8 (17.7-30.4)	34.4 (22.3-44.6)	11.5 (8.5-15)	30.6 (23.6-38.5)	35.5 (26.4-44.3)
Pennsylvania	11.4 (8.6-14.7)	27.3 (21.9-33.2)	35 (27.8-43.8)	13.6 (10.3-17.2)	37.6 (30.5-45.1)	45.1 (36.2-54.9)
Rhode Island	8.6 (6.1-11.5)	24.6 (18.3-31.4)	35.6 (22.7-47.1)	10.3 (7.5-13.6)	32.2 (23.9-41.6)	41 (27.6-53.8)
South Carolina	12.9 (9.9-16.6)	28.2 (21.6-35.1)	37 (28.1-46.3)	14.3 (11-17.8)	39.1 (30.7-47.2)	48.4 (39.1-58.3)
South Dakota	10.8 (7.6-14.7)	26.5 (19.7-33.6)	35.4 (27.5-43.6)	11.7 (8.3-15.5)	32.7 (25.1-41.2)	39.3 (28.6-51.2)
Tennessee	11 (8.5-13.8)	31.3 (24.8-38.3)	42.3 (30.9-53)	13.4 (10.3-16.7)	39 (30.6-47.4)	51.7 (38.3-63.1)
Texas	10.4 (7.9-13.2)	29.2 (23.7-35)	37.6 (29-46.4)	14.8 (11.4-18.6)	34.4 (27.8-41.7)	44.2 (34.4-53.9)
Utah	6.8 (4.7-9.1)	24.2 (18.6-30.1)	34.9 (22.8-44.6)	8.6 (6.3-11.2)	29.2 (22.7-36.7)	42.2 (29.4-53.8)
Vermont	11.5 (8.2-15.9)	22.5 (16.7-28.9)	28.8 (19.4-38.4)	11.6 (7.7-16.1)	32.6 (25-41.1)	41.7 (29-53.8)
Virginia	8.8 (6-12.2)	26.3 (20.1-32.7)	34.8 (23.2-44.3)	10.2 (7.4-13.9)	32.5 (25.3-40.1)	42.3 (30.4-54.6)
Washington	8 (5.8-10.7)	26.1 (21.1-31.6)	33.1 (26.7-41)	11 (8.3-14.2)	30.1 (23.6-37)	35.6 (28.1-43.9)
West Virginia	13.3 (10-17.2)	34.8 (27.2-42.1)	45.6 (34.7-54.3)	15.9 (12.4-19.5)	41.4 (33.3-49.6)	49.1 (35.9-59.9)
Wisconsin	7.4 (5.1-10.3)	29.8 (23.7-36.6)	38.2 (27.8-47.2)	12.1 (9.1-15.6)	36.7 (28.8-44.9)	50.8 (35.6-64.2)
Wyoming	12.2 (7.5-18.3)	31.3 (24.2-38.7)	40.1 (31-49.2)	14 (8.6-20.5)	33.3 (24.4-42.4)	40.2 (28.6-51)

SM Table 5: Prevalence of obesity by 5-year age group and sex in 1990, 2021 and 2050 at the national level, across 50 states and Washington DC

25-29 years	Male			Female		
Location	1990	2021	2050	1990	2021	2050
USA	13.2 (11.7-14.7)	33.9 (29.7-38.5)	47.6 (38.6-56.2)	15.4 (13.4-17.3)	41.8 (35.9-47.7)	56.1 (45.6-64.6)
Alabama	14.2 (10.5-18.1)	39.3 (31.9-46.9)	55.5 (43.7-67.2)	16 (12.7-19.7)	49.4 (40.8-56.8)	66.5 (54.1-75.8)
Alaska	10 (5.8-15.4)	31.8 (24.2-39.9)	44.7 (29.7-59.8)	19.7 (14.1-26.3)	44.1 (34.4-53.7)	54.7 (41.3-66.8)
Arizona	13.4 (10.3-16.8)	34.5 (27.2-42)	49.7 (35.6-62.3)	14.1 (10.8-17.9)	43 (33.7-52.2)	58.1 (42.8-70.8)
Arkansas	15.3 (10.1-21.4)	39.4 (31.2-48)	55.2 (41.3-67)	18.9 (13.3-24.7)	50.6 (41.8-58.7)	67.7 (55.1-77.7)
California	10.5 (8-13.4)	31.6 (25.1-38.7)	42.6 (30.2-55.9)	12 (9.7-14.6)	37.4 (30.1-45.1)	48.8 (34.4-60.9)
Colorado	11 (7.7-14.9)	25.7 (19.9-31.6)	39.3 (24.5-51.1)	12.6 (9.1-16.7)	33.1 (25.8-41)	46.6 (32.3-57.9)
Connecticut	12.8 (9.4-16.6)	33.6 (27-40.5)	48.2 (33.8-61.2)	16.6 (12.4-21.3)	39 (30.2-48)	54.2 (37-67.7)
Delaware	15.2 (10.4-21.4)	34.6 (26.1-43.1)	48.9 (36.2-61.8)	20.2 (15.3-26.1)	43.5 (34.2-52.5)	59.3 (44.1-70.3)
District of Columbia	9.8 (6.5-13.8)	21 (14.7-28.4)	28.9 (18.1-39.5)	20.8 (15.7-25.9)	34.6 (26-44)	44.3 (30.3-57.4)
Florida	13.4 (10.5-16.6)	30.9 (24.8-37.5)	41.9 (30-55.3)	15.6 (11.2-20.6)	38.7 (30.3-47.4)	50.5 (37.1-63)
Georgia	12 (9.2-15.1)	32.3 (25.6-39.4)	47.3 (33.5-59.5)	13.8 (10.9-16.8)	44.3 (36.2-52.7)	60 (45.4-71.5)
Hawaii	13.5 (10.1-17.6)	35.9 (27.9-43.8)	48.7 (35.1-62.6)	13.5 (10.2-17.1)	35.5 (27.6-43.9)	46.7 (33.9-59.5)
Idaho	13.2 (10.1-16.5)	36.1 (28.9-43.6)	49.4 (39.3-61.1)	14.4 (11.1-18)	42.9 (34-51.5)	55.3 (45.1-65.2)
Illinois	12.6 (9.8-16)	34.8 (28-41.8)	48.7 (34.8-61.1)	15.7 (12.6-19.2)	40.5 (32.2-48.6)	54.2 (39.8-66.2)
Indiana	14 (10.7-17.7)	37.2 (30-45)	52.1 (40.3-63.7)	18.5 (15.1-22.3)	51.3 (42.6-59.1)	66.4 (54-75.7)
Iowa	20 (15.5-24.8)	39.8 (32.9-47.1)	56.8 (43.3-67.1)	17.1 (13.2-21.8)	52.7 (44.4-60.5)	69.3 (56.3-78.4)
Kansas	12.3 (8.1-17.3)	39.4 (32.8-45.6)	56.8 (43.2-68.3)	15.7 (10.9-20.8)	47.7 (39.4-55.7)	66.2 (50.5-76)
Kentucky	15.8 (12.5-19.4)	42.2 (35.5-49.9)	59.4 (49-69.1)	20.2 (16.3-24)	49 (40.4-57.5)	65.3 (53.7-74.1)
Louisiana	21 (16.1-26.3)	35.7 (29-43)	49.2 (38.1-61.8)	24.3 (19.1-29.8)	47.8 (39.7-56.3)	63.1 (49.7-73.6)
Maine	15.5 (12-19.8)	37 (29.4-43.8)	52 (40.3-62.7)	18.4 (13.5-24)	40.9 (31.9-49.9)	54.7 (41.3-66.1)
Maryland	13.7 (10.3-17.7)	35 (28.3-42)	48.2 (36.2-58.7)	14.7 (11.4-18.1)	43.4 (35.1-51.7)	56.8 (44-68.1)
Massachusetts	14.2 (11-17.6)	30.3 (24-37)	40.3 (31.7-49)	11.7 (9.1-14.9)	32 (24.8-38.7)	43.7 (35-51.8)
Michigan	16.2 (12.7-19.9)	35.9 (29.1-43.2)	48.9 (37.4-60.1)	16.6 (13.3-20.4)	47.5 (40-54.9)	62.2 (50.1-72)
Minnesota	13.2 (10.4-16.4)	33.3 (26.8-40.7)	48.3 (34.3-61.4)	11.5 (9-14.2)	37.5 (30-45)	52.3 (37.6-63.9)
Mississippi	15.1 (10.8-20)	38.3 (31.4-45.6)	51.7 (40.1-63.4)	22.5 (17.6-28.1)	53 (44.4-61.1)	67.3 (55.6-76.1)

25-29 years	Male			Female		
Location	1990	2021	2050	1990	2021	2050
Missouri	15.8 (12.3-19.8)	36.3 (29.9-43.7)	52.2 (39.6-63.9)	16.7 (13-20.8)	46.5 (37.2-54.5)	62 (48.4-72.1)
Montana	10.6 (7.6-14)	33.2 (26.1-41.2)	47 (33.8-58.8)	12.7 (9.5-16.3)	40.2 (31.7-49.5)	54.8 (39.7-67.4)
Nebraska	10.7 (8-14)	36.5 (29.6-43.3)	54.3 (38.8-66.5)	14.6 (11.5-18.4)	44.7 (36.6-52.1)	62.2 (46.3-72.4)
Nevada	14.8 (9.6-21.2)	33.2 (25.4-41.1)	46.5 (31.4-60.1)	15.8 (10.3-22.3)	37 (27.9-46.6)	49.9 (36.4-62.8)
New Hampshire	15.4 (11.7-19.8)	36 (28.6-44.4)	50.6 (35.5-64.3)	11.8 (8.7-15.7)	42.8 (34.1-51.9)	57.1 (41.2-70.8)
New Jersey	15.4 (11-20.6)	36.6 (29.8-43.6)	49.1 (38.8-59.4)	13.2 (9.5-18)	36.1 (27.7-44.6)	48.7 (35.3-59.9)
New Mexico	10.9 (7.8-14.7)	38.1 (31-45.8)	55.9 (41.8-68.1)	14.9 (11-19)	45.2 (36.1-54.2)	61.4 (46.3-72.9)
New York	10.1 (7.8-12.7)	28.9 (23.2-35)	39 (30.3-50.4)	13.5 (10.4-16.8)	34.9 (26.9-42.7)	45.2 (34.7-57)
North Carolina	16.5 (13.1-20.3)	31.9 (25.4-38.9)	45.9 (34-56.2)	17.3 (14.1-20.7)	43.9 (35.9-51.9)	59.3 (46.3-69.2)
North Dakota	13.7 (10.5-17.6)	36.9 (29.5-44.2)	53.1 (37.3-66.4)	16.5 (12.7-20.6)	46.4 (37.5-55.1)	62.6 (45.6-73.3)
Ohio	13.8 (10.4-17.7)	36.2 (29.7-43.2)	52.5 (40.5-62.7)	17.5 (14.1-20.9)	49.2 (41.1-57.2)	66.7 (53.6-76)
Oklahoma	13.2 (10-17.3)	37.8 (30.7-45.4)	55 (41.2-67.8)	18.5 (14.6-22.8)	49.5 (41.2-57.7)	66.5 (51.7-76.6)
Oregon	13.5 (9.6-17.7)	32.2 (25.2-39.8)	43.6 (30.8-56.3)	17.7 (13.9-21.9)	41.8 (33.1-50)	54.2 (41-66.2)
Pennsylvania	17.1 (13.7-21.1)	34.5 (28.1-42)	47.6 (35.6-60)	17.3 (13.7-21.3)	43.2 (35.1-51)	55.9 (43-67.5)
Rhode Island	10.4 (7.6-14)	36.4 (28.3-44.6)	51.4 (37.8-63.7)	9.9 (7.1-12.9)	41.4 (32.5-50.5)	55.9 (39.5-68.2)
South Carolina	13.5 (10.4-17.1)	36.1 (29.3-43.7)	51.1 (38.4-62.7)	18.1 (14.4-21.9)	47.1 (38.3-55.9)	63.5 (49.1-74.2)
South Dakota	12.4 (9-15.8)	37.1 (29.6-44.5)	51.9 (37.9-64.2)	15.2 (11.1-19.6)	40.1 (31-49.7)	55.9 (39.7-68.5)
Tennessee	13.2 (10.3-16.6)	36.8 (29.6-44.2)	51.8 (39.1-63.6)	16.1 (12.7-19.9)	45.1 (36.2-53.7)	59.5 (46.2-70.7)
Texas	13.2 (10.2-16.6)	36.3 (30.1-43.1)	51.2 (37.6-62.7)	17.1 (14-20.7)	44.5 (36.4-52.2)	58.8 (44.1-69.6)
Utah	11.5 (8.6-14.7)	32 (25.4-38.9)	46.1 (31.8-58.6)	12.6 (9.8-15.8)	40.6 (32.8-48.7)	55.5 (41.5-67)
Vermont	11.8 (7.7-16.5)	30 (22.7-37.7)	42.2 (29-55.8)	12.6 (8.8-16.6)	41.8 (33.8-50.2)	53.9 (39.2-66.1)
Virginia	11.5 (8.2-15.5)	34.9 (27.7-42.9)	50.4 (34.4-63.8)	16 (12.5-19.8)	41.5 (33.8-49.1)	56.6 (42-67.8)
Washington	11.5 (8.8-14.7)	34.5 (28.2-41)	45.1 (33.2-57)	13.9 (11-17.1)	40.5 (32.9-48.5)	51.6 (39-62.5)
West Virginia	19.9 (15.7-24.5)	43.2 (35-51.2)	61.8 (50.5-71.8)	19.3 (15.5-23.2)	52.3 (43.4-60.3)	69.1 (58.3-77.1)
Wisconsin	12.3 (9.3-15.5)	30.4 (23.9-37.3)	42.9 (32.1-54.4)	16.5 (13-20.3)	42.3 (33.6-50.4)	57.5 (44.1-68.3)
Wyoming	14.1 (8.7-20.8)	28.1 (20.9-36.3)	39.5 (26.1-52.8)	18.8 (12.2-27)	38.7 (29-48.6)	51.1 (35.2-64.7)

SM Table 5: Prevalence of obesity by 5-year age group and sex in 1990, 2021 and 2050 at the national level, across 50 states and Washington DC

30-34 years	Male			Female		
Location	1990	2021	2050	1990	2021	2050
USA	16.2 (14.5-18)	39.9 (35.2-44.7)	53.9 (43.8-62.5)	18.6 (16.6-20.9)	44.5 (38.3-50.5)	58.4 (47.6-66.9)
Alabama	19.5 (15.1-24.2)	44.4 (36.3-52.8)	60.9 (48.8-71)	19.7 (16.1-23.7)	54.3 (45.2-63.1)	70.5 (57.7-79)
Alaska	17.5 (12-23.5)	42.9 (34.6-51.6)	55.9 (41.9-68.9)	20.2 (14.5-26.8)	40.9 (32-50)	51.6 (37.9-64)
Arizona	16.4 (12.5-20.2)	42.2 (34.4-50.1)	57.3 (42.8-68.9)	17.4 (13.5-21.9)	43.7 (34.6-52.6)	58.8 (43.9-70.7)
Arkansas	18.4 (12.7-25.2)	43.6 (35.6-52.4)	60.1 (46.3-72.8)	22 (16.3-28.5)	52.5 (43.6-61.4)	68.9 (55.3-78.9)
California	14.5 (11.5-17.8)	37.4 (30.6-44.6)	49.1 (35.5-63.5)	18.1 (15.2-21.5)	39.5 (32.3-47)	50.9 (37.1-62.5)
Colorado	10.2 (7-13.9)	32.2 (25.6-38.8)	46.8 (31-60)	17.1 (12.9-21.8)	36.2 (28.1-44.8)	49.5 (32.9-61.2)
Connecticut	17.9 (13.9-22.4)	41.3 (33.9-48.8)	56.1 (41.5-68.6)	13.9 (10.3-18.6)	43.8 (34.9-52.6)	58.3 (44-71)
Delaware	19.2 (13.6-25.2)	39.8 (31-48.9)	54.1 (39.8-67.1)	19.5 (14.5-25)	48.2 (38.5-57.3)	63.3 (49.2-74.1)
District of Columbia	14.6 (10.4-19.4)	24.5 (17.7-32.7)	33 (20.3-45.1)	19 (14.4-24.8)	32.8 (24.2-42)	42.4 (28.3-53.8)
Florida	14.7 (11.6-18.4)	37 (29.8-44.6)	48.4 (36.8-63.1)	19.8 (15.3-25.2)	39.7 (30.9-49.2)	50.9 (38.6-63)
Georgia	14.2 (10.8-18.3)	41.5 (34.4-48.6)	56.5 (42.8-68.5)	19 (15.6-22.6)	49.7 (41.6-58.1)	65.2 (50.7-75.1)
Hawaii	16.4 (12.5-20.9)	39.1 (30.8-47.1)	51.9 (38-64.2)	16.6 (12.9-20.5)	38.6 (29.7-47.5)	49.6 (37.6-62.6)
Idaho	14.5 (11.2-18.3)	36.1 (28.7-44.3)	49.4 (38.8-60.9)	18.1 (14.5-22.2)	45.3 (36.7-53.7)	57.6 (47.6-68)
Illinois	15.2 (12-18.8)	38.8 (31.6-46.7)	53.4 (39-66.3)	19.5 (16.1-23.4)	43.9 (34.8-53.1)	57.1 (41.5-69.1)
Indiana	18.9 (15.2-22.7)	43.1 (35.9-51.2)	57.6 (45.1-69.4)	22.7 (19.2-26.8)	50.1 (41.9-58)	65.2 (53.1-75.1)
Iowa	18 (14.1-22.1)	43.3 (35.7-50.8)	60.3 (47.3-70.8)	17.8 (13.6-22.3)	50.8 (42.3-59.6)	67.9 (53.8-78.8)
Kansas	15.7 (10.8-21.6)	45.5 (39-52.7)	62.5 (48.1-73.5)	19.2 (14.2-24.8)	48.9 (40.8-57.7)	67.1 (51-77.3)
Kentucky	19.3 (15.4-23.5)	47.2 (39.7-54.8)	64.7 (53.2-73.1)	22.1 (18.3-26.2)	51.5 (43.4-59.7)	67.2 (56.5-76)
Louisiana	19.3 (14.5-25.1)	42.6 (35.2-50.2)	56.3 (43.9-68.9)	25.8 (20.7-31.3)	54.7 (45.8-63.1)	69.1 (56.5-78.5)
Maine	17.3 (13.2-22)	37.5 (30-45.8)	53.1 (39.1-63.1)	18.1 (13.6-23.4)	43.1 (33.9-51.9)	57 (43.8-68.6)
Maryland	13.8 (10.5-17.7)	38.8 (31.9-45.9)	52.5 (39.2-62.4)	18.9 (15.3-22.7)	47.5 (39.3-55.6)	60.6 (47.2-71.4)
Massachusetts	16 (12.1-19.9)	34 (27.2-40.7)	44.3 (35-52.9)	13 (10.1-16.4)	37.7 (30-46.2)	49.3 (40.5-57.5)
Michigan	18.2 (14.4-22.4)	40.4 (33.5-47.6)	53.5 (42.1-65.2)	21 (17.5-25.2)	50.3 (42.4-57.8)	64.2 (51-74.3)
Minnesota	15.3 (12.5-18.6)	40.1 (33-47.5)	55.4 (40.2-68.2)	16.9 (13.8-20.1)	41.2 (33.2-49.5)	55.7 (39.7-67.7)
Mississippi	19.3 (14.7-24.5)	43.3 (35.5-51.6)	56.9 (45-68.9)	28.7 (23.4-34.3)	59.3 (51-66.9)	72.5 (62.5-80.3)

30-34 years	Male			Female		
Location	1990	2021	2050	1990	2021	2050
Missouri	16.6 (12.9-20.4)	45.2 (37.3-52.9)	60.5 (46.2-71.8)	21.1 (17-25.8)	47.4 (38.7-56)	62.6 (49.7-73.2)
Montana	14.2 (10.6-18.7)	37.1 (29.6-45)	50.8 (37-62)	16.8 (13.2-20.7)	41.1 (31.9-50.1)	55.7 (41.4-68.4)
Nebraska	15.5 (11.9-19.5)	43 (35.6-50.5)	60.4 (44.7-71.8)	17.8 (14.1-21.7)	46.7 (38.1-54.9)	63.3 (47.3-73.8)
Nevada	17.7 (12-24.5)	42.3 (33.3-51.5)	55.7 (40.4-68.6)	17.7 (12.3-24.2)	41.8 (32.1-52.1)	54.3 (40.7-67.3)
New Hampshire	14.4 (10.9-18.3)	39.4 (31.3-48.2)	54.4 (38.9-68.4)	16.5 (12.6-20.5)	38 (28.7-47.1)	52.5 (36.1-66.3)
New Jersey	14.5 (10-19.6)	35.2 (28-42.5)	47.7 (35.9-59.4)	14.9 (11.1-19.3)	37.9 (29.8-46)	50.5 (38.7-60.9)
New Mexico	15.8 (12-20.1)	46 (38.2-53.9)	63.2 (49.8-75.1)	17.4 (13.6-21.8)	47.5 (38.9-56.1)	63.3 (49.6-74.8)
New York	16.3 (13-19.7)	34.9 (28.6-41)	45.4 (36.6-55.1)	13.6 (10.6-17.2)	40 (31.9-48.5)	50.1 (40.3-61.4)
North Carolina	17.1 (13.9-20.6)	40.1 (33.1-48)	54.2 (41.7-65.4)	23.3 (19.5-27.2)	49.5 (41.2-57.6)	64.7 (51.6-74.7)
North Dakota	19 (14.9-23.3)	43.1 (35.2-51.1)	59 (42.6-72.9)	18.2 (14.2-22.2)	44.6 (35.3-53.3)	60.9 (43.8-71.6)
Ohio	17.8 (14.1-21.7)	44.7 (37.4-51.7)	60.9 (49.2-70.9)	16.8 (13.5-20.6)	49.6 (41.3-58.1)	66.8 (54-75.7)
Oklahoma	18 (13.7-22.7)	44.7 (37.1-52.6)	61.8 (48.2-73.9)	19.6 (15.9-23.7)	50.3 (41.7-58.6)	66.2 (52-76.7)
Oregon	14 (10.1-18.6)	36.9 (29.5-45)	48.9 (36.5-62.2)	17.9 (14.2-21.7)	45.7 (37-53.7)	57.8 (44-68.4)
Pennsylvania	16.4 (12.7-20.5)	38.3 (31.9-45.1)	51.5 (39.6-63.7)	21.3 (17.5-25.7)	41.7 (33.9-50.2)	54.2 (41.6-65.5)
Rhode Island	14 (10.5-18.2)	40.9 (33.1-49)	56.4 (42.6-69.4)	14.2 (11.1-17.7)	45.9 (36.7-55.6)	60.2 (45.5-72)
South Carolina	15.5 (12.1-19.3)	43.6 (35.6-52.1)	58.5 (45.4-69.3)	20.1 (16.5-24)	52.8 (43.5-61)	68.2 (55.4-77.2)
South Dakota	17.3 (12.9-22)	46.1 (37.9-54.4)	60.6 (46.8-72.6)	15.7 (12.1-20)	47.6 (38.5-56.3)	63 (48.6-74.3)
Tennessee	18.6 (15.3-22.3)	40.3 (33.1-47.5)	55.6 (42.5-68.2)	22.1 (18.2-26.3)	49 (40.1-57.7)	62.9 (49.6-73.8)
Texas	17.1 (13.7-21.2)	46.7 (39.6-53.6)	61.1 (47.2-72.5)	17.8 (14.4-21.5)	48.2 (40.4-55.4)	62.1 (49.2-73.4)
Utah	14.2 (10.8-18.2)	38.1 (31.1-45.6)	52.5 (38.9-64.7)	17.9 (14.5-21.5)	40.3 (31.8-48.9)	54.9 (38.8-67.3)
Vermont	17.8 (13-23.3)	38.7 (31-46.1)	51 (37.6-64.1)	18.7 (13.6-24.2)	39 (30.6-47.8)	51 (37.3-64.5)
Virginia	19.3 (14.9-24.7)	41.8 (34-50.1)	57.1 (41.4-70.8)	18.6 (14.7-23.2)	44.9 (36.8-52.7)	59.7 (44.9-70.5)
Washington	12.7 (10-15.9)	34.8 (28.3-42.1)	45.7 (33.4-58.3)	17.9 (14.6-21.6)	39.7 (31.9-47.9)	50.3 (37.1-62.3)
West Virginia	24.8 (20.3-30.1)	49.2 (41.3-56.6)	67.1 (56.7-76.2)	23.5 (19.3-27.9)	50 (41.5-58.1)	67.2 (55.5-76.1)
Wisconsin	18.9 (14.9-23)	39.4 (31.8-47.3)	52.2 (40-63.9)	21.6 (17.4-25.9)	48.2 (38.9-56.4)	62.8 (49.9-72.9)
Wyoming	20.4 (13.3-28.9)	37.8 (29.7-45.8)	49.5 (35.4-62.6)	20.3 (13.7-27.9)	43.5 (33.4-53.4)	55.5 (40.4-69.3)

SM Table 5: Prevalence of obesity by 5-year age group and sex in 1990, 2021 and 2050 at the national level, across 50 states and Washington DC

35-39 years	Male			Female		
Location	1990	2021	2050	1990	2021	2050
USA	18.4 (16.6-20.3)	41.2 (36.4-45.8)	55.3 (45.3-63.3)	21.4 (19.1-23.9)	46.9 (41.2-52.8)	60.5 (49.6-68.8)
Alabama	21.1 (16.7-25.8)	46.4 (38.3-54.3)	63.1 (51.1-72.9)	23.3 (19-27.7)	54.8 (46.9-62.7)	70.7 (60-79.6)
Alaska	20.1 (14.2-26.7)	41.8 (33.1-50.2)	55.9 (40.5-68.3)	24.7 (18.4-32)	44.9 (35.9-54.2)	55.5 (41.9-67.3)
Arizona	15.8 (12.3-19.8)	42.9 (35.1-50.6)	58 (44-69.4)	20.7 (16.6-25.4)	47 (37.6-56.1)	61.3 (46.7-72.6)
Arkansas	20.9 (14.6-28)	46.9 (38.5-55.1)	63.5 (51-74.4)	21.9 (16.3-28)	57.7 (49.4-65.8)	73 (60.6-81.9)
California	15.6 (12.7-18.8)	37.7 (31.3-44.7)	49.3 (35.9-62.2)	20.1 (16.8-23.4)	42 (34.8-49.2)	53.1 (40-64.3)
Colorado	12.1 (8.8-16.4)	33.2 (26.5-40.3)	48.2 (31.8-60.4)	17.9 (13.6-22.5)	36.5 (28.9-44.5)	49.8 (34.8-61.6)
Connecticut	17.4 (13.7-21.7)	36.2 (28.4-44.4)	51.7 (36.7-64.4)	14.7 (11.1-19)	45.5 (36.7-54.3)	60.4 (45.2-72.4)
Delaware	18.5 (12.7-24.5)	42.4 (33.4-51.8)	57.1 (43.7-69.1)	24.9 (19.7-30.7)	50.7 (41.1-59.6)	65.4 (50.3-76.5)
District of Columbia	17.3 (12.4-22.7)	27.4 (19.6-35.9)	36.1 (23.4-48.5)	24.4 (18.9-30.3)	42.4 (32.9-52.4)	52.2 (37.7-64.4)
Florida	15.8 (12.4-20)	36.6 (29.7-44.6)	47.9 (36.7-61.1)	20.4 (15.3-26.1)	41.2 (32.5-50.5)	52.6 (39.9-64.3)
Georgia	15.9 (12.3-20.2)	44.3 (36.5-51.7)	59.9 (46.3-71)	20.6 (17-24.5)	49.4 (41.3-57.8)	64.7 (51.9-74.9)
Hawaii	14.8 (11.3-18.9)	40.7 (32.2-49.2)	53.7 (40-66.4)	18.9 (15-23.4)	40.3 (31.7-49.1)	51.3 (38.3-62.8)
Idaho	17.3 (13.6-21.4)	38.8 (31.2-46.9)	52 (41-64.1)	20.6 (16.5-25.2)	42.9 (34.7-51.3)	55 (44.7-64.9)
Illinois	18.6 (15-22.6)	40.5 (33.1-47.8)	55.1 (40.4-66.9)	22.9 (19-27.1)	50.6 (41.8-58.9)	63.3 (48.8-73.5)
Indiana	24 (19.7-28.4)	42.4 (35-50.2)	57.7 (45.9-68.8)	24.3 (20.3-28.3)	51.2 (42.8-59.4)	65.8 (53.1-76.2)
Iowa	19.1 (14.8-24.3)	47.4 (39.6-55)	63.9 (51.7-73.7)	23.8 (19.1-28.7)	51.7 (44-59.4)	68.1 (55-77.8)
Kansas	18.3 (13.1-24.1)	45.2 (38-51.7)	62.5 (48-72.9)	20.3 (15.6-26.5)	52.6 (45.2-60.1)	70.1 (55.5-79.2)
Kentucky	20.6 (16.5-25.2)	46.3 (37.8-54.1)	64.3 (52.6-73.3)	24 (20-28.3)	51.6 (43.8-59.7)	67.2 (56.4-76.2)
Louisiana	18.7 (13.4-25.3)	47.1 (38.8-54.9)	60.6 (48.2-72.6)	24.7 (19.8-30.1)	55.8 (47.8-63.7)	69.8 (58.6-78.9)
Maine	17.9 (13.9-22.4)	41.5 (33.8-49.2)	57.2 (45.2-67.8)	16.5 (12-21.5)	46.1 (36.8-55)	59.6 (47.6-70.5)
Maryland	17.4 (13.8-21.7)	40.7 (33.3-48.2)	54.7 (41.7-65.8)	21.8 (17.8-26.3)	50.4 (42.3-58.4)	63 (51.1-73.7)
Massachusetts	17 (13.5-21.2)	36.7 (30-43.9)	47.5 (38.1-57.4)	15.6 (12.3-19.1)	37.1 (29.3-45.6)	48.5 (39.9-57.1)
Michigan	20.9 (16.5-25.2)	43.7 (36.5-51.2)	56.8 (45.6-68.3)	22.9 (19-27.2)	52.4 (44.6-60.9)	66.1 (54.1-76.5)
Minnesota	16.5 (13.5-20.1)	41.3 (34-48.4)	57 (41.1-69.5)	20.5 (17-24.1)	44.6 (36.8-52.6)	58.8 (43.3-70.1)
Mississippi	19.9 (14.8-25.5)	45.9 (37.8-53.6)	59.7 (48.2-70.6)	32.2 (26.4-38.4)	56.8 (48.9-64.1)	70.4 (59.3-79.2)

35-39 years	Male			Female		
Location	1990	2021	2050	1990	2021	2050
Missouri	19.5 (15.3-24.4)	44.7 (37-52.3)	60.4 (46.9-71.9)	27.2 (22.2-32)	48.7 (40-56.7)	63.6 (50.4-74.7)
Montana	14.5 (10.5-18.7)	35.1 (27.1-43)	49 (35.1-61.1)	18.6 (14.7-22.9)	42.5 (34.1-51.3)	57.3 (42.6-69.4)
Nebraska	16.1 (12.2-20.3)	46.2 (38.9-53.4)	63.5 (48.3-74.6)	22.9 (18.9-27.3)	49.8 (42.5-57.8)	66.4 (50.9-75.9)
Nevada	19.7 (13.3-27.3)	36.6 (27.6-46.1)	50.3 (35.8-64.2)	19.9 (14.1-26.7)	41.4 (32.3-50.8)	53.7 (40.9-66.3)
New Hampshire	16.3 (12.3-20.8)	44.6 (35.5-53.1)	59.5 (44.2-71.8)	18.8 (14.7-23.1)	47.6 (39.1-56.2)	61 (46.4-73.5)
New Jersey	19.5 (14.1-25.2)	40.5 (32.9-48)	53.1 (40.8-63.8)	14.8 (10.7-19.3)	41.8 (34-49.5)	53.8 (41.7-64.3)
New Mexico	16.4 (12.6-20.8)	45.6 (37.9-52.9)	63 (48.1-74)	20.7 (16.5-25.5)	51.2 (42.6-59.3)	66.6 (53.1-77.8)
New York	17 (13.8-20.7)	36 (29.4-42.6)	46.6 (37.1-58.1)	19.8 (15.8-24.1)	42.3 (34.1-50.4)	52.4 (41.9-63.7)
North Carolina	21.2 (17.4-25.4)	40.9 (34.1-48.2)	55.1 (43.2-66.1)	23.1 (19.3-27)	53.1 (45-60.6)	67.3 (55.3-76.3)
North Dakota	21 (16.8-25.5)	46.9 (39-55.4)	62.4 (46.6-74.5)	20.6 (16.5-25.1)	50.9 (41.6-59.3)	66.3 (51.1-76.4)
Ohio	19.7 (15.8-23.7)	45 (36.5-52.3)	61.4 (48.6-71.5)	23.8 (19.8-28.2)	51.5 (43.5-59.5)	68.3 (55.8-77.6)
Oklahoma	18.9 (14.7-23.5)	48.7 (40.9-56.1)	65.6 (52.4-76.6)	22.8 (18.2-28)	54.1 (45.4-62)	69.9 (56.8-79.5)
Oregon	17.4 (13-22.4)	38.9 (31.2-47.2)	51.1 (37.7-64.9)	23.9 (19.7-28.6)	45.7 (37.6-53.3)	57.6 (43-68.5)
Pennsylvania	23 (18.8-27.5)	43.3 (36.3-50.6)	56.6 (44.8-67.9)	20.7 (16.8-24.9)	49.5 (41.9-57.1)	61.2 (49-72)
Rhode Island	17.2 (13.2-21.7)	44.1 (35.5-52.5)	59.4 (45.2-71.9)	17.4 (13.8-21.6)	41.7 (33.2-50.3)	56.1 (41.7-68.2)
South Carolina	22.7 (18.2-27.6)	43.6 (36.2-51.5)	58.9 (44.9-70.2)	25.2 (20.8-30.1)	52.9 (44.7-60.7)	68.4 (55.7-78.1)
South Dakota	21.9 (17.1-27.3)	41.8 (33.9-49.6)	57.4 (42.5-70.4)	19.7 (15.1-24.9)	47.7 (38.6-56.9)	62.8 (46.8-74.4)
Tennessee	19.9 (16.3-23.9)	44.5 (36.8-52)	59.5 (46.7-71.1)	21.8 (17.7-26)	53.7 (45.2-62.2)	67.1 (55.4-77.4)
Texas	20.8 (17-25.4)	46.5 (39.5-53.7)	61.2 (47.9-72.7)	24.1 (20.4-28.2)	48.8 (41.1-55.9)	62.3 (48.3-72.4)
Utah	16.3 (12.5-20.6)	39.9 (31.6-47.8)	54.4 (39.8-66.6)	19.5 (15.7-23.5)	46.8 (38.7-54.6)	61.1 (46-72)
Vermont	15.6 (11-21)	41.9 (34.2-50.1)	54.4 (40.3-67.4)	21.7 (16.8-27)	41.7 (34.1-49.9)	53.7 (40.2-66.2)
Virginia	18 (13-23.2)	40.8 (33.1-49.5)	56.5 (40.5-69.6)	20.6 (16.3-25.2)	49.3 (40.9-57.3)	63.5 (48.3-74.8)
Washington	17.3 (13.7-21.1)	38.8 (32.4-45.5)	50 (37.2-61.2)	20.7 (17.1-24.8)	43.9 (36.6-51.4)	54.6 (42.1-66.1)
West Virginia	23.6 (19.2-28.6)	53.2 (44.6-61.6)	70.9 (60.1-79.3)	29.5 (25.3-34.1)	52 (43.5-60.1)	68.6 (58.4-77.6)
Wisconsin	17.4 (13.8-21.6)	41 (33.2-48.4)	53.9 (42.2-64.7)	23.2 (19-27.8)	51 (43-59.1)	65.1 (52.7-75)
Wyoming	19.9 (13-28)	36.6 (28.8-44.4)	48.5 (34.7-61.2)	22.6 (15.6-31)	46.7 (36.7-57.2)	58.5 (44.1-72.2)

SM Table 5: Prevalence of obesity by 5-year age group and sex in 1990, 2021 and 2050 at the national level, across 50 states and Washington DC

40-44 years	Male			Female		
Location	1990	2021	2050	1990	2021	2050
USA	21.5 (19.4-23.7)	45.6 (40.3-50.8)	59.5 (49.9-67.6)	24.1 (21.5-26.7)	48.2 (41.9-54.4)	61.3 (50.7-69.8)
Alabama	22.6 (17.9-27.9)	53.7 (45.3-61.5)	69.3 (58.6-78)	25.3 (20.9-30.1)	56.3 (47.4-64.6)	71.7 (60.1-80)
Alaska	22.6 (16.3-30.8)	45.2 (35.9-54.5)	58.9 (44.1-71.5)	21.6 (15.5-28.1)	45.3 (35.6-55.1)	55.8 (42.2-68)
Arizona	18.4 (14.1-23.3)	47.3 (39-55.9)	62 (47.2-74.2)	22.6 (17.6-27.4)	44.5 (34.9-53.5)	59 (43.1-71.9)
Arkansas	22.5 (15.7-30.4)	53.3 (44.1-61.9)	69 (56.9-78.9)	25 (18.1-32.3)	58.4 (49.4-66.3)	73.5 (61-82)
California	20.3 (16.6-24)	39.4 (32-47.2)	51.1 (37.8-64)	23.2 (19.6-26.9)	45.1 (37.6-53.1)	56 (42.7-67.5)
Colorado	15.2 (11.3-20.2)	37.5 (29.6-45.2)	52.8 (37.8-65.4)	19.6 (15.1-24.6)	39.3 (31.2-47.9)	52.3 (35.6-64)
Connecticut	17.6 (13.5-22)	44.7 (36.6-52.7)	59.7 (45.5-71.5)	21.4 (16.7-26.9)	42.9 (33.9-52)	57.5 (42.3-70)
Delaware	24.4 (17.9-31.9)	47.8 (37.7-57.4)	62.3 (47.5-74.5)	29 (23-35.8)	49.6 (40.6-58.6)	64.5 (49.5-74.9)
District of Columbia	17 (11.5-23)	32.8 (24.1-42.1)	41.8 (28.6-53.3)	31.5 (25.1-38.4)	40.5 (30.6-50)	50.1 (34.2-62.2)
Florida	20.4 (16.4-24.8)	40.4 (32.4-48.7)	51.9 (39.1-65.4)	25.3 (20.1-31.4)	45.3 (35.5-54.7)	56.4 (42.9-68.3)
Georgia	20.2 (16.2-24.8)	47.4 (39-55.8)	63 (49.1-74.3)	19.7 (16.1-23.9)	50.9 (42.9-58.7)	65.7 (52.8-75.1)
Hawaii	18.9 (14.7-23.7)	42.1 (33.4-51.3)	55 (41.7-68.4)	21.7 (17.4-26.7)	37.7 (28.6-46.4)	48.3 (34.9-60.6)
Idaho	22.2 (17.8-26.9)	48.5 (39.4-56.4)	61.4 (51.1-72.4)	23.9 (19.6-28.9)	44.7 (35.7-53.8)	56.6 (47.3-66.8)
Illinois	22.9 (18.7-27.6)	47.7 (39.2-55.9)	62.2 (47.6-73.4)	24.9 (20.9-29)	51.6 (42.7-59.2)	64.2 (50.2-74.2)
Indiana	24.8 (20.1-29.8)	50.5 (42.4-58.6)	65 (53.7-75.1)	31.5 (26.9-36.1)	53 (44.6-60.8)	67.3 (55.2-76.8)
Iowa	24.6 (19.6-30.2)	53.2 (45.2-61)	68.9 (57.6-77.8)	24 (19.5-29.4)	55.6 (47.6-63.5)	71.6 (59-80.2)
Kansas	22.4 (16.5-29.4)	49.9 (42.4-57.1)	66.6 (52.8-76.7)	24.6 (18.6-31)	53.4 (45.2-61.4)	70.5 (55.8-79.8)
Kentucky	24.5 (19.9-29.3)	53.1 (44.1-60.9)	70.5 (60.3-78.7)	26.2 (21.9-30.7)	54.5 (46-63.1)	69.3 (58.3-77.8)
Louisiana	22.7 (16.9-29)	49.3 (40.5-57.2)	62.5 (51.2-74.6)	25.4 (19.6-31)	55.5 (46.7-64)	69.3 (56.5-78.3)
Maine	24 (19.2-29.3)	50.2 (41.7-58.2)	65 (52.8-74.8)	21.5 (16.7-27.1)	46.6 (37.2-55.6)	59.8 (46.2-70.4)
Maryland	19.2 (14.9-23.6)	45.7 (37.9-53.6)	59.3 (45.8-70.4)	23.9 (19.5-28.4)	49.6 (41.4-57.9)	62.1 (48.5-73)
Massachusetts	20.3 (16.4-24.9)	43.8 (36.2-51.3)	54.8 (45.6-64)	20.6 (16.6-24.8)	43.6 (35.8-51.9)	54.9 (45.8-63.6)
Michigan	21.9 (17.7-26.7)	46.2 (38.5-53.4)	59.4 (48-70.4)	27.9 (22.9-33)	49.9 (41.6-58.3)	63.6 (50.6-72.9)
Minnesota	21.1 (17.1-25)	46.5 (38-54.1)	62.1 (47.5-73.4)	21 (17.7-24.9)	45.1 (37.1-53.2)	59.1 (43.8-70.3)
Mississippi	21.3 (15.9-27.3)	50.1 (42-58.5)	63.4 (52.7-73.8)	26.3 (20.9-32.1)	57.8 (49.7-66.2)	70.7 (59.4-79.4)

40-44 years	Male			Female		
Location	1990	2021	2050	1990	2021	2050
Missouri	21.8 (17.3-26.8)	50.3 (42.3-58.1)	65.5 (52.7-75.5)	22.8 (18.1-27.6)	51.3 (42-60)	65.6 (52.9-75.9)
Montana	18.5 (14.3-23.5)	44.7 (36.4-52.8)	58.3 (44-70.1)	22.1 (17.7-26.8)	45.1 (35.8-53.9)	59.4 (43.7-70.8)
Nebraska	23.5 (18.6-28.7)	49.6 (41.8-56.7)	66.6 (52.3-77.1)	25.2 (20.7-29.8)	50.4 (42.1-58.4)	66.4 (50.8-76.3)
Nevada	21.1 (14-28.8)	44.3 (35.2-54.3)	57.9 (43.8-71.7)	23 (16.6-30.2)	45.7 (35.4-56)	57.6 (42.8-69.8)
New Hampshire	21.4 (16.8-26.7)	50.3 (41.7-58.8)	64.8 (50-77.8)	18 (13.6-22.8)	46.4 (37.3-55.7)	60.1 (44.2-73.6)
New Jersey	23.5 (17.9-30.1)	43.5 (35.5-51.4)	56.3 (44.6-66.7)	17.6 (13.3-22.5)	44.3 (35.6-53)	56.4 (43.9-66.9)
New Mexico	17.1 (13-22)	45.9 (37.7-53.9)	63.2 (47.6-74.6)	20 (15.6-24.9)	49.1 (40.6-57.8)	64.1 (49.8-76.1)
New York	19.1 (15.4-22.9)	42 (35.2-49)	52.8 (42.7-64.1)	22.5 (18.6-27)	42.2 (33.6-50.5)	51.8 (41.4-62.1)
North Carolina	19 (15.5-23.3)	46.7 (38.9-54.1)	60.6 (48.3-70.6)	24.2 (20.1-28.6)	51.8 (43.1-60)	65.8 (53.5-75.5)
North Dakota	20.7 (16-25.9)	56.2 (47.8-64.4)	70.4 (55.3-80.6)	22.9 (18.3-27.6)	49.5 (40.7-58.2)	64.8 (48-75.8)
Ohio	22.9 (18.5-27.6)	49.7 (41.9-57.2)	65.8 (53.5-75.5)	30.2 (25.7-35)	53.4 (45.1-60.9)	69.5 (56.9-78.3)
Oklahoma	17.6 (13.1-23)	51.6 (42.8-59.3)	67.9 (55.6-79)	25.3 (20.5-30.3)	53.6 (45-62.2)	69.2 (55.8-79.7)
Oregon	20.7 (16-26.1)	41.8 (33.6-50)	54.2 (41.4-67.3)	23.6 (19.3-28.5)	46.2 (37.6-54.7)	57.8 (43.6-68.9)
Pennsylvania	26.7 (22.1-31.5)	48.6 (41.2-55.7)	61.7 (50.5-73.1)	27.4 (22.8-32.5)	47.6 (39.2-55.3)	59.1 (46.5-69.9)
Rhode Island	20.1 (15.8-24.9)	45.4 (36.8-53.3)	60.8 (46.4-73.3)	19.3 (15.4-23.4)	43.2 (34.3-52.1)	56.7 (42-69.1)
South Carolina	21.6 (17.6-26.4)	49 (41-56.9)	64 (51-75.1)	28.6 (24-33.3)	56.1 (47.1-64.4)	70.9 (57.9-80)
South Dakota	22.1 (17.1-27.8)	49.1 (40.4-57.4)	64.3 (50.4-75.6)	21.3 (16.5-26.7)	46.2 (37.1-55.8)	61.1 (44.9-73.4)
Tennessee	23.7 (19.6-28)	48.6 (40.3-57)	63.2 (50.1-73.9)	23.6 (19.1-28.2)	53 (44-61.8)	66.1 (52.9-75.6)
Texas	24.8 (20.3-29.3)	48.9 (41.3-56.3)	63.4 (50-74.4)	23.8 (19.6-28.1)	50.4 (42.4-58.2)	63.5 (49.7-74)
Utah	18.7 (14.6-23.2)	42.6 (34.3-50.3)	57.3 (42.9-70.6)	24.5 (20.1-28.8)	44.1 (35.5-52.5)	58.1 (42.7-69.1)
Vermont	17.7 (12.9-23.3)	39.5 (30.6-48.1)	52.4 (37.6-65.9)	20.8 (16-25.9)	44.2 (35.7-53.1)	55.9 (41.6-68.1)
Virginia	20.1 (15.1-26.2)	45.8 (37.4-54)	61.1 (45-73.9)	21.7 (17.4-26.4)	46.9 (37.7-55.2)	60.9 (45.2-72.4)
Washington	19.3 (15.4-23.7)	39.5 (32.7-46.9)	50.8 (38.4-62.1)	23.5 (19.5-27.9)	46 (38.6-53.6)	56.3 (44.3-68)
West Virginia	25.3 (20.5-30.1)	55.6 (47-63.5)	73.1 (63.2-81)	31.1 (26.4-36.1)	56.8 (47.7-65.2)	72.1 (62.1-79.9)
Wisconsin	22.2 (17.6-27.4)	50.1 (42.3-58.9)	62.4 (51.5-73.4)	26 (21.3-30.9)	49.3 (40.6-58.5)	63.6 (51.5-73.4)
Wyoming	25.1 (17.6-34.5)	47.3 (38.4-56.6)	58.8 (44.6-71.4)	26 (17.6-35.3)	47.5 (37.5-57.4)	58.8 (44.4-71.5)

SM Table 5: Prevalence of obesity by 5-year age group and sex in 1990, 2021 and 2050 at the national level, across 50 states and Washington DC

45-49 years	Male			Female		
Location	1990	2021	2050	1990	2021	2050
USA	22.8 (20.6-24.9)	46.8 (41.4-51.6)	60.4 (50.1-68.6)	26 (23.3-28.8)	48 (42-54)	61 (50.7-69.3)
Alabama	20.8 (16-26)	51.2 (43.1-59.3)	67.3 (55.5-76.5)	26.8 (21.8-31.8)	56.4 (47.6-64.3)	71.8 (60.4-79.8)
Alaska	22.4 (15.8-30.1)	47.7 (39-55.9)	61 (46.1-72.8)	28.1 (20.6-36.3)	49.6 (39.4-59.7)	59.8 (45.5-72.2)
Arizona	23.4 (18.3-28.4)	45.9 (37.1-54.1)	60.7 (45.4-72.1)	19.4 (15-24.6)	45.7 (36.7-55.6)	59.6 (44.2-72.1)
Arkansas	25.2 (18.2-32.9)	53.3 (44.4-62.1)	69 (56.3-78.6)	27.4 (20.7-34.9)	54.6 (45.7-63.4)	70.3 (58.2-79.7)
California	22.3 (18.1-26.4)	39.3 (32.3-46.3)	50.9 (36.5-64.7)	21.7 (18.3-25.3)	41.9 (34.7-49.1)	52.5 (38.2-64.3)
Colorado	17.1 (12.3-22.5)	40.7 (32.9-48.3)	55.8 (37.6-67.2)	20.2 (15.5-25.4)	39.1 (30.9-47.7)	52 (35.1-63.3)
Connecticut	17.7 (13.4-22.7)	45.8 (37.7-53.8)	60.7 (44.7-72.7)	22.9 (17.7-28.5)	42.4 (33.5-51.7)	56.7 (41.3-70.1)
Delaware	22.9 (16.2-30.1)	48 (38.5-57)	62.4 (47.7-73.9)	31.3 (24.2-38.2)	50.9 (42.1-59.9)	65.2 (51.6-75.6)
District of Columbia	14.7 (10.2-20.1)	38.3 (29-47.5)	47.7 (33.6-59.6)	32.4 (25.8-39.5)	45.3 (36-55.1)	54.7 (39.8-67.1)
Florida	22.2 (17.8-26.9)	47.1 (38.9-55.3)	58.3 (45.9-71.2)	26 (20.7-32)	47.3 (37.7-57.1)	58.2 (45.2-70.1)
Georgia	19 (14.9-23.8)	45.7 (37.4-54.1)	61.4 (46.7-71.9)	24.1 (19.6-29)	50.2 (42.2-58.1)	65 (50.9-74.8)
Hawaii	24.5 (19.6-29.9)	43.6 (33.7-51.8)	56.2 (41.7-69.1)	18.1 (13.8-23)	39 (29.9-48)	49.5 (36.4-62.4)
Idaho	22.4 (17.4-27.7)	45.4 (37-53.7)	58.4 (48.3-69.1)	25 (19.9-30.4)	44 (35.2-52.7)	55.8 (44.4-65.9)
Illinois	27 (21.9-32.1)	46.8 (39-55)	61.1 (46.6-72.2)	26.5 (21.9-31.2)	44.6 (35.9-53)	57.3 (42.2-69.5)
Indiana	26.9 (22.3-32.6)	49.4 (41.5-57.3)	63.8 (51.4-74.7)	30.3 (25.7-34.9)	51.4 (43.6-59.8)	66 (53.8-76.1)
Iowa	28.4 (22.6-34.2)	54.1 (45.7-61.5)	69.6 (58-78.9)	24.5 (19.5-30.4)	51 (42.2-59.4)	67.3 (54.5-77.7)
Kansas	28.3 (21.1-36.4)	50.4 (43-57.5)	67 (52.4-76.6)	27.1 (20.5-34.5)	53.1 (45.1-60.5)	70.1 (55.3-79.4)
Kentucky	25.3 (20.2-30.2)	55.4 (47-63.6)	71.7 (61.1-79.7)	27.9 (23.3-33)	54.4 (45.6-62.6)	69.1 (58.4-78.6)
Louisiana	25.4 (18.7-32.2)	53.3 (44.8-61.5)	66.3 (55.3-77)	30.6 (24.2-37.3)	57.7 (48.8-65.8)	71 (59.2-80.4)
Maine	25.3 (19.9-31.1)	51.1 (43.1-59.1)	66.1 (53-76.1)	27.8 (21.5-33.8)	47.2 (37.8-56.4)	60.1 (47.6-72.1)
Maryland	23.9 (18.8-29.1)	46.7 (38.7-54.2)	60.4 (47.4-70.9)	29.8 (24.8-34.9)	50.9 (42.8-58.7)	63 (50.1-73.9)
Massachusetts	20.7 (16.4-25.6)	37.4 (30.2-44.8)	48.4 (37.2-58.3)	22.1 (17.9-27)	39.4 (31.8-47.4)	50.5 (42.1-59)
Michigan	27.2 (22.3-32.8)	50.7 (43.3-57.5)	63.4 (51.9-72.8)	29.6 (24.8-34.7)	51.7 (44-59.4)	65.3 (53.3-74.9)
Minnesota	23 (18.7-27.2)	47.9 (39.9-55.5)	63.1 (47.2-74.4)	22.2 (18.2-26.5)	47.1 (38.9-55.1)	60.8 (45.5-71.5)
Mississippi	22.5 (16.4-29.3)	51.1 (42.7-58.8)	64.1 (52.8-74.8)	32.3 (26.3-38.7)	60.3 (52-67.5)	72.8 (61.8-81)

45-49 years	Male			Female		
Location	1990	2021	2050	1990	2021	2050
Missouri	23.8 (18.9-29.4)	52.9 (44.3-60.9)	67.5 (55.3-76.7)	28.9 (23.8-34.5)	52.5 (43.8-60.8)	66.7 (54.2-77)
Montana	16.4 (11.9-21.7)	40.2 (31.7-49.2)	54.1 (39.6-67.2)	24.7 (19.8-29.8)	45.7 (36.9-55.1)	59.6 (44-72.8)
Nebraska	21 (16.1-26.3)	51.3 (43.3-58.5)	67.6 (52.2-77.6)	26.2 (20.9-31.8)	50.7 (42.6-59)	66.4 (51.4-76)
Nevada	23.5 (16.8-31.3)	47.7 (38.1-56.7)	60.8 (47.2-72.2)	27.8 (20.3-35.7)	41 (31.1-51)	52.5 (38.8-65.7)
New Hampshire	21.6 (16.7-27.4)	48.5 (40.3-57.4)	63 (47.4-75.7)	21.3 (16.4-26.5)	43.8 (35-52.8)	57.4 (41.3-70.5)
New Jersey	16.7 (11.7-23.1)	40.3 (32.4-48.4)	52.9 (39.3-64.2)	25.4 (19.6-31.9)	40.8 (32.4-49.3)	52.7 (40.8-64.1)
New Mexico	20.1 (15.8-25.3)	49.6 (40.5-58.2)	66.4 (52.3-77.7)	18.2 (14-23.2)	51.3 (42.4-60.5)	65.8 (51-77.4)
New York	19.2 (15-23.5)	40.4 (33.4-47.3)	51.2 (40.1-61.6)	28.7 (24-33.8)	40.5 (32.1-49)	50.1 (40-61.6)
North Carolina	25.1 (20.3-30)	49.2 (41.5-56.5)	62.9 (51.3-72.3)	29.5 (25.1-34.4)	52.7 (44.8-60.8)	66.7 (54.5-75.9)
North Dakota	24.5 (19.7-29.7)	53.4 (44.5-61.6)	68.1 (52.8-79.2)	22.8 (17.9-28.6)	50.5 (41.2-59.5)	65.6 (49.1-76.7)
Ohio	24.5 (19.5-29.7)	52.5 (44.9-59.5)	68.1 (56.5-77.2)	25.4 (20.8-30)	52.9 (45.3-61.1)	69 (56.1-78.6)
Oklahoma	21.3 (16.2-26.9)	52.8 (44.2-60.5)	68.8 (55.2-79.5)	24.4 (19.4-30.1)	52.2 (43.6-60.6)	67.6 (53.6-78.4)
Oregon	23.2 (18.3-29.4)	44.5 (36.1-52.3)	56.7 (43.4-69.8)	26.1 (21.1-31.1)	46.9 (38.6-55.6)	58.4 (45.6-69.9)
Pennsylvania	24.6 (19.9-29.5)	50.6 (43.5-58)	63.5 (51.8-74)	25.9 (21.1-30.9)	50.9 (42.8-58.8)	62.3 (50.2-73.3)
Rhode Island	24.6 (19.5-30)	46.4 (37.4-54.6)	61.9 (47-73.8)	24.9 (20-30.2)	43.9 (34.9-53.6)	57.2 (43.1-69.9)
South Carolina	23.8 (19.3-29.4)	50 (41.1-58.6)	64.7 (51.9-74.7)	27.9 (23-33.3)	55.7 (46.9-64.3)	70.3 (57.1-79.8)
South Dakota	26 (20.8-31.8)	50.7 (41.2-59)	65.2 (51.4-76.6)	25.5 (20.2-31.2)	49 (39.4-58.5)	63.4 (48.7-75)
Tennessee	21.2 (17-26)	51.8 (43.8-59.6)	66.2 (53.9-76.2)	21.7 (17.5-25.9)	50.4 (41.6-59.1)	63.1 (51.2-73.9)
Texas	24.6 (19.9-29.7)	52.9 (45.7-59.9)	66.7 (52.8-77.3)	28.4 (23.5-33.2)	55.9 (48-63.6)	68.1 (55.1-77.7)
Utah	22.4 (17.5-27.5)	44.5 (36.4-53.1)	58.9 (43.7-70.7)	25.8 (20.8-31)	44.5 (36-52.7)	58.4 (43.8-69.9)
Vermont	21.4 (15.4-28.3)	42.6 (34.4-50.3)	55.5 (41.4-68)	27.4 (20.6-34.1)	44.8 (35.8-53.1)	56.4 (42.7-68.5)
Virginia	17.3 (12.7-22.9)	47.8 (39.2-55.5)	63 (48.5-74.6)	28 (22.9-34.2)	49.6 (41.7-57.8)	63.5 (49.4-74)
Washington	20.2 (16.2-24.7)	43.6 (36.4-50.9)	54.6 (41.8-66.3)	26.5 (21.8-31.5)	45.5 (37.4-53.5)	55.8 (42.8-67.3)
West Virginia	24.8 (19.7-30.2)	54.4 (45.3-62.8)	72.2 (61.1-79.7)	30.6 (25.6-35.6)	57.7 (48.9-66.4)	72.5 (61.7-80.6)
Wisconsin	28.1 (22.4-34.1)	47.2 (38.3-55.4)	59.8 (47.7-71.2)	27.6 (22.8-33.2)	48.2 (39.4-56.5)	62.4 (48-72.8)
Wyoming	26.2 (17.8-35.4)	46.3 (37.1-55)	57.8 (42.9-70.7)	25.3 (17.1-34.3)	43.8 (33.4-53.3)	55.2 (39.1-69.4)

SM Table 5: Prevalence of obesity by 5-year age group and sex in 1990, 2021 and 2050 at the national level, across 50 states and Washington DC

50-54 years	Male			Female		
Location	1990	2021	2050	1990	2021	2050
USA	23.5 (21.5-25.8)	46.7 (41.6-51.7)	60.2 (50.6-68.3)	28.4 (25.7-31.3)	48.7 (42.3-54.6)	61.5 (51-69.4)
Alabama	23.4 (18.2-28.7)	50.5 (41.6-58.5)	66.9 (54.4-76.2)	28 (22.9-33.3)	57.1 (48.3-65.1)	72.3 (61.1-80.8)
Alaska	23.3 (16.1-30.9)	46.2 (37-54.8)	59.8 (45-71.8)	25.3 (17.5-34.1)	49.1 (39.3-58)	59.3 (45.5-70.7)
Arizona	23.3 (18.3-28.8)	49.6 (41-57.9)	64.1 (50.3-75)	24.9 (20-30.3)	47.4 (38.1-56.5)	61.2 (46.9-72.7)
Arkansas	26.4 (18.9-34.5)	51.2 (42.8-59.7)	67.1 (54.6-77.1)	32.8 (25.2-40.9)	55.1 (46.2-63.5)	70.6 (58.3-79.6)
California	20.9 (16.9-25.2)	40.2 (33.1-47.7)	51.6 (38.2-64.5)	28.4 (24.4-32.8)	45.8 (38-53.8)	56.5 (42.6-68.5)
Colorado	17.2 (12.5-23.3)	36.9 (30.4-44.4)	52.4 (36.7-64.6)	24.2 (18.5-29.8)	39.9 (31.5-48)	52.6 (37.5-63.7)
Connecticut	25.1 (19.5-30.9)	45 (36.6-52.5)	59.9 (44.9-71.3)	24.6 (19.1-30.6)	43.4 (34.5-52.1)	57.6 (41.7-69.5)
Delaware	25.5 (18.5-33.5)	48.5 (38.7-57.5)	63.2 (49.3-74.5)	30.5 (23.7-37.4)	52 (42-60.9)	66.4 (52.5-76.6)
District of Columbia	23.4 (17.6-30)	40.3 (30.9-49.9)	49.9 (36.2-61.8)	42.6 (35.5-50)	45.1 (34.9-54.8)	54.5 (39.9-66.9)
Florida	20.7 (16.4-25.3)	42.8 (34.5-50.8)	54.1 (41.8-66.8)	30 (23.7-36)	44.6 (35-54.1)	55.4 (43.2-67.2)
Georgia	21.9 (17.1-27.3)	50.1 (42.6-58.2)	65.2 (51.8-75.4)	25.3 (20.7-30)	53.5 (44.7-62)	67.8 (53.6-78.3)
Hawaii	22 (17.5-27.5)	44.2 (35.1-52.5)	56.8 (44.1-69.7)	17.6 (13.4-22)	39.6 (31-48.7)	50.1 (36.6-62.6)
Idaho	23.9 (18.7-29.3)	51.7 (43.6-59.8)	64.2 (53.6-73.7)	24.6 (19.6-29.8)	46.4 (37-55.6)	58 (46.9-68.8)
Illinois	26.2 (21.2-31.4)	49 (40.7-56.7)	63.2 (48.7-74)	29.4 (24.7-34.9)	48 (39.3-56.3)	60.5 (46.9-70.8)
Indiana	25.9 (21.3-31.1)	49.5 (41.9-57.2)	64.4 (52.9-73.9)	36.5 (31.6-41.7)	52.1 (43.7-59.9)	66.4 (53-75.4)
Iowa	28.5 (22.5-34.5)	53.5 (45.6-60.7)	69.1 (57.2-78)	31.4 (25.6-37.8)	51.8 (43.4-59.8)	68.1 (54.3-77.9)
Kansas	24.3 (17.3-32.2)	50.9 (43.5-57.9)	67.6 (54.7-77.4)	24.8 (18.3-32)	52.5 (44-59.7)	69.5 (54.7-79)
Kentucky	24.4 (19.6-29.7)	53.9 (45.4-61.8)	71.2 (61-79.7)	27.8 (23-32.5)	53.8 (45-62.5)	68.6 (58.1-77.1)
Louisiana	21.6 (15.9-28.5)	51.1 (42.8-58.7)	64.1 (52.3-75.9)	29.7 (23.2-36.4)	55.1 (46-63.2)	68.9 (57.1-78.1)
Maine	23.6 (18.1-29.6)	49.4 (40.6-57)	64.6 (51.5-74.2)	27.2 (21.6-33.3)	45.9 (36.8-55.6)	59 (46.4-70)
Maryland	23.4 (18.4-28.6)	47.1 (39.4-54.7)	60.8 (46.6-71.4)	30.3 (24.6-35.7)	50.4 (42-58.6)	62.8 (50.2-73.1)
Massachusetts	25 (19.7-31)	41.8 (34.5-49.2)	52.8 (41.9-62.9)	24.2 (19.5-29.5)	38.8 (30.9-46.6)	49.7 (41.3-57.9)
Michigan	28.4 (23.2-34.2)	49.8 (42.1-57.5)	62.8 (51.2-73)	35.7 (30-41.5)	52.7 (44.7-60.7)	65.9 (54.1-75.2)
Minnesota	24.3 (20.2-29.2)	48.1 (40.2-56.1)	63.3 (47.9-74.3)	25.4 (21.2-29.8)	43.8 (35.2-51.8)	57.8 (42.8-69)
Mississippi	29.3 (22.8-36.1)	53.7 (44.7-61.2)	66.2 (56-75.9)	34.9 (28.7-42)	60.3 (51.7-68.4)	72.8 (62.1-81.4)

50-54 years	Male			Female		
Location	1990	2021	2050	1990	2021	2050
Missouri	27.5 (21.9-33.1)	50.5 (42.1-58.1)	65.6 (52.5-76)	27.8 (22.3-33.2)	52.8 (43.5-61.1)	66.8 (53.4-76.7)
Montana	21.1 (16.1-26.7)	43.7 (35-52.1)	57.3 (43.2-69.4)	25.3 (20.7-31.1)	42.9 (33.8-51.9)	57 (42.9-69.2)
Nebraska	28.7 (22.7-34.8)	50.2 (42.3-57.6)	67 (52.5-77.3)	26.1 (20.7-32.1)	51.8 (43.8-60.1)	67.7 (52.7-77.4)
Nevada	24 (16.7-32.8)	45.2 (36.4-53.9)	58.3 (44.4-70.4)	24.9 (17.9-32.7)	46.9 (37.3-56.3)	58.4 (45.5-70.4)
New Hampshire	26.9 (21.5-33)	49.1 (40.9-57.5)	63.6 (49-75.7)	24.3 (19-29.9)	42.2 (33.5-50.4)	56.1 (40-68.8)
New Jersey	19.1 (13.4-25.6)	43 (35.4-50.9)	55.5 (44.2-66.7)	20.4 (14.7-27)	39.6 (31.3-47.7)	51.3 (40-62.4)
New Mexico	18.5 (13.7-24.2)	46.2 (38.1-54.8)	63.5 (49.1-75.5)	26.2 (21.2-32)	54.5 (45.5-63.2)	68.6 (55-79.1)
New York	19.2 (14.9-24)	45.6 (38-52.4)	56.2 (46.9-66.6)	27.7 (22.6-32.8)	45.9 (37-54.7)	55.5 (44.4-66.5)
North Carolina	21.6 (17.1-26.6)	48.4 (40.9-56.2)	62.3 (49.5-72.3)	28.5 (24.3-32.9)	53 (44.3-60.6)	66.9 (54.1-75.8)
North Dakota	26.9 (21.8-32.9)	49.3 (40.4-57.4)	65 (49.5-76.7)	29.9 (24.4-35.6)	46.9 (37.7-55.2)	62.6 (47.1-73.6)
Ohio	24.7 (20.1-29.9)	50.6 (42.6-58.3)	66.6 (54-76.4)	28.6 (23.7-33.7)	52.7 (44-60.3)	68.6 (56.2-77.8)
Oklahoma	21.7 (16.5-27.4)	55.9 (48-63.8)	71.3 (58.2-80.8)	26.8 (21.4-32.1)	57.3 (48.7-65.3)	72 (58.9-81)
Oregon	24.3 (18.8-30.5)	45.4 (36.9-53.2)	57.4 (43.9-69.7)	31.1 (25.5-36.9)	48.4 (39.6-56.4)	60 (46.5-71)
Pennsylvania	25.8 (20.9-31.2)	50.4 (42.6-58.1)	63.4 (51.6-74.3)	31.9 (26.4-37.5)	47.3 (39.1-55.4)	58.5 (47-69.2)
Rhode Island	24.3 (18.8-30.4)	46.9 (38.7-55.2)	62.4 (48.6-74.4)	27.5 (22.3-32.6)	41.9 (32.7-50.7)	55.5 (41.8-67.7)
South Carolina	28.1 (23.2-33.8)	48.4 (40.5-56.2)	63.5 (50.2-74.3)	28.7 (23.9-34.1)	54.8 (45.9-63.4)	69.4 (56.8-78.9)
South Dakota	25.1 (19.3-31.9)	45.5 (36.8-54.3)	61 (46-72.9)	26.8 (20.8-32.4)	51.2 (41.6-59.9)	65.4 (51.3-76.1)
Tennessee	24.6 (20-29.4)	51.4 (43.4-59.1)	65.7 (53.1-77.2)	26.3 (21.9-31.2)	52 (42.4-60.7)	65.1 (52.9-75.5)
Texas	24.9 (19.7-30.9)	47.4 (40.4-54.7)	61.9 (47.5-73.3)	27.6 (22.7-32.6)	54.2 (45.9-61.8)	66.7 (54.1-76.7)
Utah	22.6 (17.4-28.2)	46 (37.8-53.9)	60.1 (46.4-72.1)	27.7 (22.4-33.2)	44.1 (35.6-52.8)	57.9 (43.1-69.8)
Vermont	23.4 (17.1-30.6)	43.7 (35.1-51.8)	56.5 (42.1-70.1)	28.3 (21.9-35.4)	42.6 (33.4-51.7)	54.4 (39.8-66.2)
Virginia	26.9 (20.5-33.2)	49.1 (41.1-56.8)	64 (49.7-75)	27.7 (21.8-33.4)	50.2 (41.8-58.4)	63.8 (49.6-74.3)
Washington	23.7 (19.2-28.7)	41.7 (34.7-48.8)	53 (40-64.7)	25.2 (20.4-30.1)	43.9 (35.7-51.4)	54.1 (41.7-65.5)
West Virginia	24.7 (19.5-30.3)	58 (49.7-65.5)	74.8 (64.5-81.6)	29.3 (24.3-34.1)	58.5 (49.8-66.2)	73.3 (62.6-80.6)
Wisconsin	22.5 (17.1-28.2)	47.4 (39.1-55)	60.1 (48.9-70.9)	31.9 (26.3-37.7)	52 (43.4-60.9)	65.6 (52.5-75.9)
Wyoming	28.1 (19.2-38.6)	48.3 (39.6-57.3)	59.8 (46.7-71.3)	27.5 (19.2-37.1)	47.2 (36.3-56.5)	58.6 (43.6-71.3)

SM Table 5: Prevalence of obesity by 5-year age group and sex in 1990, 2021 and 2050 at the national level, across 50 states and Washington DC

55-59 years	Male			Female		
Location	1990	2021	2050	1990	2021	2050
USA	23 (20.9-25.2)	46 (40.9-51.1)	59.4 (49.6-67.6)	28.5 (25.8-31.4)	47.7 (41.6-53.8)	60.2 (50.5-68.1)
Alabama	23.9 (18.9-29.5)	50.5 (42-58.6)	66.7 (55.8-76.2)	28.1 (22.9-33.6)	55.1 (46.8-63.9)	70.2 (58.6-78.5)
Alaska	24.5 (17.4-33.4)	44.8 (35.4-54.4)	58.6 (43.4-72)	37.3 (28.6-46.4)	45.1 (35.7-54.5)	55.3 (42.1-66.3)
Arizona	25.8 (20.6-31.8)	45.1 (36.3-53.3)	60 (45.8-72.3)	26.9 (21.5-32.4)	47 (38.1-55.9)	61.3 (46.3-73)
Arkansas	26.2 (19.1-34.7)	50.8 (42.1-58.9)	66.9 (53.9-77.5)	34 (26.5-42.3)	51.4 (43-59.7)	67.3 (54.4-76.8)
California	19.1 (15.1-23.9)	39.8 (32.3-47.4)	51.4 (37.1-64.5)	24.8 (20.8-29)	44.4 (36.5-51.6)	55 (41.7-65.4)
Colorado	25.8 (19.8-33)	36.8 (29.6-44.4)	52.2 (35.4-64.4)	27.5 (21.3-34.1)	39.9 (32.1-48.6)	52.5 (37.4-63.5)
Connecticut	24.4 (18.6-30.4)	45 (37.1-53.4)	60.2 (44.8-73)	22.2 (17-28)	43.4 (34.2-52.2)	57.5 (41.7-69.8)
Delaware	22.3 (15.5-29.9)	47.5 (38.7-56.1)	62.2 (48.8-73.8)	27.9 (21.2-35.3)	50.5 (41.7-59.5)	64.7 (52.3-75.9)
District of Columbia	24.8 (18.6-31.6)	37.4 (28.1-46.5)	46.7 (33.4-58.4)	38.6 (31.8-45.8)	51.7 (41.2-61.8)	60.4 (47.1-71.7)
Florida	23.6 (19-28.8)	43.2 (35.7-51.4)	54.5 (41.7-67.4)	28.3 (22.7-34.5)	44.1 (35.1-53.1)	54.8 (41.8-66.6)
Georgia	20.8 (15.8-26.5)	49.2 (41.2-56.6)	64.4 (50.1-74.2)	27.3 (22.4-32.5)	51.2 (43.4-59.5)	65.9 (53.8-75.1)
Hawaii	19.3 (14.5-24.4)	38.1 (29.5-46.7)	51.2 (36.8-65.1)	20.5 (16.2-25.5)	34.5 (27.1-42.4)	45 (32.6-57.3)
Idaho	17.2 (12.4-21.8)	46.9 (38.6-54.8)	60.1 (49.9-71.3)	28.6 (23.1-34)	47 (38.8-55.6)	58.6 (49.1-68.6)
Illinois	27.1 (21.9-32.4)	48.9 (40.9-56.3)	63.3 (49-75)	32.6 (27.8-37.6)	48.1 (39.3-56.4)	60.3 (47.5-71)
Indiana	25.4 (20.5-30.2)	48.9 (41.1-56.4)	63.7 (51.8-74.2)	30.3 (25.7-35.3)	53.5 (45-61.6)	67.5 (56.1-76.9)
Iowa	28.1 (22.2-34.8)	53.3 (45.4-61.1)	68.9 (56.6-78.1)	28.4 (22.5-34.5)	50.9 (42.7-59.2)	67.4 (54.7-77.2)
Kansas	26.7 (19.1-35.3)	50.7 (43-57.7)	67.4 (53.9-76.8)	25.6 (18.8-33)	52.8 (45-60.8)	69.9 (55.2-79)
Kentucky	19.9 (15.4-25.1)	51.5 (42.9-59.8)	69.1 (57.6-77.8)	29.3 (24.7-34.6)	52.6 (44.3-60.2)	67.5 (57.4-76.2)
Louisiana	25 (18.1-32.6)	49.9 (41.9-57.7)	63.2 (51.2-74.4)	34.1 (27.6-41.1)	55 (47.3-63)	68.7 (57.3-77.6)
Maine	25 (19.1-31.5)	46 (37.8-54.9)	61.5 (48.3-73.3)	31 (24.3-37.7)	47.8 (38.9-56.6)	60.6 (48.5-71.6)
Maryland	22.4 (17.2-28.3)	47.5 (39.7-54.9)	61.2 (47.5-71.7)	29.3 (24-35.1)	49.2 (41.2-57)	61.5 (49.8-71.9)
Massachusetts	22.3 (17.2-27.9)	41.8 (33.9-49.1)	52.9 (42.6-63.4)	24 (19.3-29.3)	39.6 (31.6-47.9)	50.5 (42.3-59.2)
Michigan	22.8 (17.6-28.8)	47.3 (39.9-55)	60.5 (48.6-71.2)	33.6 (28.3-39.3)	54 (46.1-61.5)	67 (55.5-76.4)
Minnesota	20 (15.9-24.6)	47.2 (39.8-54.8)	62.7 (48.2-74)	25.9 (21.3-30.7)	45.2 (37.3-53.2)	58.9 (44.3-69.5)
Mississippi	35.2 (27.4-42.8)	52.6 (44.2-60.6)	65.5 (54.6-75.6)	39.8 (33.3-46.2)	58.3 (50.1-66.4)	71.1 (60.2-79.2)

55-59 years	Male			Female		
Location	1990	2021	2050	1990	2021	2050
Missouri	23 (18.2-29)	50.5 (42.1-58.6)	65.6 (53.8-76.3)	25.8 (20.4-31.8)	52.3 (44.1-60.2)	66.1 (53-75)
Montana	19.8 (15.1-26)	43.5 (35-52.2)	57.4 (43.2-70.2)	25.6 (20.3-31.1)	46.8 (37.9-56.1)	60.4 (46.8-72.4)
Nebraska	26 (20.1-32)	52.3 (44.5-59.5)	68.6 (54.6-78.4)	25.3 (20.1-30.7)	51.8 (44.1-59.6)	67.4 (53.5-76.2)
Nevada	23.1 (15.7-31.9)	43 (33.6-52.8)	56.5 (41.8-69.3)	25.2 (17.9-33.6)	44.1 (35.5-53.9)	56 (43.5-68.7)
New Hampshire	21.8 (15.9-28.3)	45.6 (37.3-53.5)	60.6 (45.1-73.6)	23.4 (18.2-29.2)	41.3 (33.3-49.8)	54.9 (40.5-68.1)
New Jersey	24.8 (17.7-32.4)	45.1 (37.1-52.6)	57.8 (45.1-68.1)	24.9 (18.7-31.4)	41.8 (33.9-49.4)	53.2 (42.5-63.8)
New Mexico	19.8 (14.5-25.4)	44.7 (36.6-53)	62.3 (47-74.6)	20.6 (15.7-26)	47.7 (39-56.4)	62.7 (49-73.9)
New York	21.9 (17.5-26.3)	41.8 (34.9-48.9)	52.7 (42.4-63.2)	25.8 (20.7-31.1)	44 (35.8-52.3)	53.4 (43.3-64)
North Carolina	22.9 (18.2-28.1)	46.8 (39.1-54.2)	60.8 (49.2-71.2)	29.8 (25.2-34.6)	52.2 (44.3-60.5)	65.8 (55.2-74.6)
North Dakota	27.4 (21.9-33.9)	51.8 (43.5-60.1)	66.9 (50.9-77.9)	28.6 (22.8-34.9)	45.8 (36.9-54.7)	61.2 (44.8-72.2)
Ohio	23.9 (19-29.6)	49.7 (42.6-57.2)	65.6 (54.2-75.2)	28.1 (23.4-33.1)	53.2 (45.1-61)	69.1 (57.7-77.9)
Oklahoma	26.5 (20.2-33.3)	51.3 (42.8-58.9)	67.7 (54.9-78.2)	27.6 (22.3-33.7)	54.2 (46-62.4)	69.4 (56.8-79.1)
Oregon	20.6 (15.1-26.1)	42.9 (35-50.8)	55.1 (42-67.8)	34.3 (28.4-40.1)	45.7 (37.5-53.2)	57.2 (44.7-68.6)
Pennsylvania	28.7 (23.2-34.2)	51.6 (44.2-58.6)	64.5 (53.3-74.5)	36.1 (30.7-41.9)	46.7 (39.1-54.4)	57.9 (46.5-68.7)
Rhode Island	21.3 (16.2-26.9)	48 (39.5-56.5)	63.3 (48.5-75.7)	25.7 (20.6-31.4)	42.3 (33.4-51.2)	55.9 (42.1-67.9)
South Carolina	25.1 (20.3-30.5)	48.4 (40-56)	63.4 (51-73.6)	31.3 (26.3-36.8)	52.3 (43.6-60.8)	67.4 (54.7-76.7)
South Dakota	21.3 (16-27.3)	48.9 (40.6-57.4)	63.9 (49.8-75.5)	27 (21-33.3)	47.2 (37.7-56.3)	61.7 (47-72.7)
Tennessee	21.1 (16.9-25.3)	48.7 (40.1-56.6)	63.7 (50.4-74.6)	27.5 (22.7-32.7)	51.5 (42.2-60.4)	64.2 (52.3-74.8)
Texas	19.8 (15.2-25)	48.1 (40.4-55.4)	62.7 (48.6-73.3)	31.2 (26-37.1)	49.8 (41.6-58)	62.6 (48.5-73)
Utah	18 (13.2-23.2)	44.7 (36.4-52.8)	59.3 (45-71.4)	27 (22.1-32.3)	48 (39.6-56.4)	61.5 (47.4-71.8)
Vermont	27.2 (20.3-34.3)	45.1 (36.4-53.6)	57.7 (43.4-70.5)	25.9 (19.9-32.6)	42.2 (34.5-50.6)	53.8 (40.6-65.6)
Virginia	20 (14.6-25.7)	46.6 (38.2-55.3)	62 (46.8-73.9)	29.1 (23.5-35.3)	50.2 (41.9-57.4)	63.6 (50-73.3)
Washington	19.8 (15.3-24.8)	42.8 (35.2-50)	54 (40.9-66.1)	23.7 (19.2-28.6)	44.6 (36.9-52.6)	54.7 (42.3-64.3)
West Virginia	25 (19.9-30.4)	55 (46.9-63)	72.5 (61.8-80.1)	30.2 (25.4-35.6)	54.6 (46.1-62.7)	70 (59.8-77.5)
Wisconsin	24.5 (19-30.4)	48.3 (40.1-56.1)	60.8 (49.1-71.4)	30.1 (24.8-35.6)	46.1 (37.7-54.5)	60.2 (47.8-71.1)
Wyoming	25.3 (17.3-34.3)	45.4 (37.1-53.5)	57.3 (43.7-69.7)	27.6 (18.2-38.4)	44.2 (33.6-54.1)	55.5 (40-68.5)

SM Table 5: Prevalence of obesity by 5-year age group and sex in 1990, 2021 and 2050 at the national level, across 50 states and Washington DC

60-64 years	Male			Female		
Location	1990	2021	2050	1990	2021	2050
USA	21.5 (19.4-23.7)	44.8 (40-49.8)	58.4 (48.6-66.4)	28.3 (25.5-31.3)	48.5 (42.1-54.4)	61.1 (50.9-68.9)
Alabama	21 (16.1-26.8)	50.4 (42.3-58.5)	67 (54.6-76.6)	29.2 (23.9-34)	54.3 (45.4-62.4)	69.6 (57.3-78)
Alaska	24.2 (16.8-33)	42.5 (33.7-51.4)	56.4 (40.4-69.8)	34 (25.5-42.7)	45 (35.2-54.7)	55.2 (41.2-67.1)
Arizona	19.3 (15-24.5)	44 (36.5-52.3)	59.2 (44.2-71.5)	24.4 (19-30.1)	45.3 (36.6-54.2)	59.6 (44.9-70.7)
Arkansas	22.7 (15.5-31.1)	50.3 (41.8-58.9)	66.5 (53.5-77.2)	29.3 (22.1-36.8)	54 (45.4-62.4)	69.8 (57.2-79)
California	20.6 (16.6-24.8)	38.4 (31-46.1)	50.3 (36.1-62.2)	27 (22.8-31.6)	47.5 (39.8-55.1)	58.1 (44.1-68.9)
Colorado	17.1 (11.7-23.5)	35.6 (28.4-43.5)	51.1 (34-63.5)	24.5 (18.5-31.3)	39.7 (31.5-48)	52.6 (36.5-63.5)
Connecticut	19.3 (14.1-24.9)	41.9 (34-50.4)	57.2 (42.3-69.3)	29.1 (23.2-35.1)	43.3 (34.3-51.9)	57.7 (40.9-70.4)
Delaware	20.1 (13.6-28.1)	47.4 (37.6-56.8)	62 (47.9-73.5)	28.8 (22.3-36.7)	50.8 (41.7-59.6)	65.2 (51.4-75.9)
District of Columbia	30.2 (23.2-38)	35.1 (26-45.3)	44.6 (30.7-57)	37.2 (30.3-44.5)	48.8 (38.6-58.5)	58 (44-69.9)
Florida	21.7 (17.5-26.1)	42.7 (34.8-50.6)	54.2 (42.9-67)	27.7 (22.3-33.9)	43.3 (34.4-52.8)	54.2 (41.5-66.1)
Georgia	21.8 (16.3-27.8)	42.9 (35.1-51.1)	59 (44.7-70.3)	29.7 (24.3-35.5)	51.9 (43.5-60.4)	66.7 (52.8-76.5)
Hawaii	16.5 (12.1-21.6)	36.4 (28-44.6)	49.3 (35.6-62.4)	20.5 (15.7-25.8)	32.4 (24.5-41.1)	42.7 (29.9-55.5)
Idaho	22.2 (17.4-27.2)	41.6 (33.6-50)	54.9 (44-67)	24.4 (19.4-29.6)	45.8 (37.5-54.9)	57.6 (47.7-68)
Illinois	23.9 (18.7-29.1)	46.9 (39.3-54.6)	61.4 (46.7-72.7)	29.8 (24.6-35)	52.9 (45.1-61.1)	64.9 (52-75.3)
Indiana	23.2 (18.1-28.5)	49.2 (41.2-57.1)	64 (51.3-74.5)	32.3 (27.6-37.3)	53.8 (45.2-61.4)	67.7 (55.1-76.9)
Iowa	22.9 (17.6-28.7)	48.7 (40.9-56.3)	65.3 (52.3-75.3)	24.9 (19-30.8)	49.2 (41.1-57.6)	65.9 (51.6-76)
Kansas	18.3 (12.1-25.8)	48.5 (40.8-55.5)	65.5 (51.3-75.8)	27.1 (20.3-34.4)	52.1 (44.5-59.3)	69.4 (54.7-78.9)
Kentucky	20.9 (16.3-25.7)	50.1 (42-57.7)	68.1 (56.8-77.2)	29.2 (24.3-34.4)	54.6 (46.7-62.6)	69.3 (58.4-77.8)
Louisiana	21.7 (15.2-28.9)	47.9 (40.1-55.5)	61.3 (49.3-73.1)	32.9 (26.1-40.4)	55.3 (46.9-63)	69.1 (57.8-78.1)
Maine	19.6 (14.7-25.6)	44.5 (36.4-52.3)	60.3 (47.7-72.1)	24.2 (18.5-30.4)	45.1 (36.1-54.8)	58.1 (45.9-69.5)
Maryland	21.5 (16.5-27.3)	45.3 (37.6-53)	58.7 (45.8-69.3)	26.6 (21.5-32.1)	50.2 (41.9-58.2)	62.6 (50.3-72.7)
Massachusetts	18.8 (14.3-24.1)	43.6 (35.8-51.1)	54.5 (44.7-64.5)	26.7 (21.3-32.4)	42.2 (33.8-50)	52.9 (44.4-60.6)
Michigan	27.3 (21.8-33.2)	46.3 (39.2-53.5)	59.7 (47.8-69.9)	32.2 (26.7-37.2)	51.4 (43.1-59)	64.6 (52.9-74.6)
Minnesota	21.3 (17-26)	46 (37.5-53.4)	61.8 (46.2-73.2)	26.4 (22.1-31.4)	45.3 (37-53.5)	59 (42.6-70.4)
Mississippi	26 (19.6-33.2)	49.6 (41-57.2)	62.9 (51.2-73.4)	33.8 (27.3-40.8)	58.9 (50.3-66.6)	71.7 (60.4-80.2)

60-64 years	Male			Female		
Location	1990	2021	2050	1990	2021	2050
Missouri	23.3 (18.2-29.1)	47.2 (39.8-55.5)	62.6 (50.2-73.1)	31 (25.3-37)	49.7 (40.6-57.9)	64.1 (50-74.6)
Montana	19.1 (14.2-24.6)	42.5 (34.3-51.3)	56.5 (42.7-69.2)	22.6 (17.4-28.4)	44.5 (35.9-53.4)	58.5 (43.9-69.7)
Nebraska	20.7 (15.7-26.1)	51.1 (43-58.6)	67.7 (53.3-77.6)	31.9 (26.4-37.7)	52.4 (43.9-60.2)	68 (52.9-77.6)
Nevada	19.3 (12.4-27.1)	43 (33.6-52.7)	56.6 (40.9-69.2)	25.2 (17.6-33.6)	40.4 (31.2-50.2)	52.3 (38.7-65.4)
New Hampshire	23.7 (18.1-29.6)	44.4 (36.4-53.2)	59.6 (43.9-72.8)	22.2 (17.2-27.7)	42.2 (33.5-51.1)	56.3 (40.7-69)
New Jersey	20.2 (14.1-27.1)	43.7 (35.8-51.3)	56.3 (45.1-66.3)	27.2 (21.2-34.3)	42.2 (33.3-50.5)	53.9 (40.7-64.5)
New Mexico	16.7 (11.7-21.6)	40.2 (32.3-48)	58.3 (42.8-70.5)	19.4 (14.7-24.9)	47.8 (39.4-56.7)	63.2 (49.4-74.1)
New York	20.1 (16-25.1)	42.2 (35.2-49.4)	53 (43.1-63.9)	26.4 (21.7-31.6)	43.3 (35.6-51.8)	52.9 (42-63.8)
North Carolina	21.4 (17.2-26.3)	49.7 (42.3-57.4)	63.6 (50.6-73.1)	31.9 (27.1-36.8)	53.6 (45.1-61.6)	67.3 (55.8-76.7)
North Dakota	21.3 (16.4-27)	51.1 (42.8-60)	66.3 (50.8-77.7)	32.7 (27-39.5)	47.6 (38.5-57)	63.3 (47.4-74.6)
Ohio	24.1 (18.9-29.4)	47.1 (39.7-54.7)	63.8 (52.6-73.4)	30.1 (25-35.4)	53.4 (45.1-61.3)	69.3 (57.3-77.9)
Oklahoma	19 (13.9-25)	49.4 (41.4-57.5)	66.3 (51.6-77.3)	28.6 (22.9-34.4)	53 (44.5-60.8)	68.5 (55.6-78.2)
Oregon	19.5 (14.6-25.4)	41.6 (34-49.6)	53.9 (41.5-67.7)	25.2 (19.8-30.5)	46 (37.1-53.9)	57.6 (43.9-68.6)
Pennsylvania	22.4 (17.4-27.4)	46.6 (39.6-53.9)	60.1 (48.3-71.2)	32.2 (27-38.2)	48.6 (40.2-56.4)	60.1 (48.3-69.8)
Rhode Island	20.3 (15.6-25.4)	41.5 (33-50)	57.5 (42.9-71.1)	27.7 (22.4-33.2)	43.9 (35.4-52.9)	57.6 (43.2-69.7)
South Carolina	19.3 (14.8-24.5)	45.9 (37.6-54.2)	61.5 (46.9-71.7)	28.3 (23.5-33.6)	52.1 (42.4-60.8)	67.3 (54.4-76.6)
South Dakota	20.8 (15.7-26.9)	47.8 (39-56.1)	63.1 (48.5-74.4)	26.5 (20.3-32.9)	51.2 (41.1-60.9)	65.1 (50-76.5)
Tennessee	19.4 (15-24)	47.1 (38.5-55.4)	62.4 (48.4-73.2)	29.9 (25-34.9)	53.1 (43.8-61.7)	65.9 (54.3-75.9)
Texas	22.1 (17-27.2)	49.6 (42.4-57.1)	64.1 (50.3-74.4)	25.3 (20.5-30.8)	53 (44.8-60.8)	65.6 (53.1-75.8)
Utah	18.2 (13.5-23.4)	45.3 (37.5-53.6)	59.7 (45.1-71.2)	25.9 (20.9-31.6)	44.2 (35.8-52.6)	58.3 (43.5-70.1)
Vermont	24.1 (18.1-31.5)	41.2 (33.9-49.5)	54.2 (40.9-66.9)	31.2 (24.4-38.6)	40.4 (32.4-49)	52.1 (39.2-64.8)
Virginia	16.5 (11.4-22.5)	46.7 (38.6-54.8)	62.3 (47-74)	31.7 (25.4-38.1)	52.3 (43.6-60.3)	65.4 (50.4-75.8)
Washington	21.7 (17.4-26.4)	41.6 (35-48.9)	52.8 (40.3-64.6)	19.5 (15.4-24.2)	42.6 (34.8-50.2)	53 (40.3-64.6)
West Virginia	20.2 (15.9-25.2)	52.4 (44-61)	71.1 (60.4-78.4)	28.9 (24.1-34.2)	55.1 (46.6-63.1)	70.7 (60.3-78.6)
Wisconsin	29.3 (23.8-35.2)	49.3 (41.2-57.3)	62 (49.6-72.4)	31.7 (26.3-37)	48.9 (40.3-57.3)	62.8 (50.2-73.1)
Wyoming	22.9 (15-32.8)	42.6 (33.8-51.3)	54.5 (41.3-67.2)	27 (17.4-38)	44.5 (34.3-53.7)	56.4 (41.5-69.7)

SM Table 5: Prevalence of obesity by 5-year age group and sex in 1990, 2021 and 2050 at the national level, across 50 states and Washington DC

65-69 years	Male			Female		
Location	1990	2021	2050	1990	2021	2050
USA	19.1 (17-21.2)	42.9 (37.7-48)	56.5 (46.5-64.6)	26.9 (24.2-29.9)	47 (40.5-53.3)	59.4 (48.6-67.5)
Alabama	18.2 (13.7-23.6)	43.6 (35.5-51.6)	61.3 (48.5-71.5)	29.3 (24.7-34.8)	53.1 (44.2-61.5)	68.7 (58.1-78)
Alaska	26.3 (18.2-35.3)	43.9 (34.7-52.7)	57.6 (41.9-70.7)	37.2 (28.3-46.5)	47.2 (37.8-55.9)	57.1 (43.1-68.4)
Arizona	17.6 (13.1-22.4)	40.8 (32.7-49.3)	56.3 (40.7-68.8)	24.3 (19.2-29.9)	47.8 (38.5-57.1)	61.7 (46.2-73.5)
Arkansas	18.4 (12.1-26)	47.7 (39-56.2)	64.4 (51.2-74.7)	29 (22.2-37.2)	52.2 (43.1-60.5)	67.9 (54.4-77.6)
California	17.2 (13.6-21.4)	36.4 (29.8-43.7)	48.3 (34.7-61.2)	22.9 (18.9-27.2)	43.7 (35.6-51.4)	54.2 (40.5-66.3)
Colorado	13.5 (9-19.8)	33.8 (26.6-40.8)	49.4 (32.4-62.8)	20.2 (14.7-26.3)	39.1 (31.1-47.7)	51.5 (35.9-62.8)
Connecticut	16.1 (11.2-21.5)	39.6 (32.1-47.2)	55.2 (40.3-67.7)	24.9 (19.6-30.7)	43.5 (34.6-52.9)	57.6 (42.3-70.6)
Delaware	22.4 (16.1-30)	47 (37.7-56.3)	61.9 (46.6-75)	27.9 (21-34.8)	51.7 (42.7-60.5)	65.9 (52.4-75.7)
District of Columbia	22.6 (16.4-29.9)	30.2 (21.7-39.8)	39.4 (25.4-52.5)	31.9 (26-38.3)	46.8 (37.2-56.5)	55.7 (40.6-67.6)
Florida	19.1 (15.1-23.3)	43.2 (35.6-51.1)	54.6 (42.4-67.4)	23.8 (19-29.1)	44 (34.7-53.7)	54.4 (41-66.3)
Georgia	18.9 (14-24.4)	42.3 (34.3-50.3)	58.5 (43.7-69.9)	28.3 (23.4-33.7)	49.2 (41.3-57.7)	64 (50-73.8)
Hawaii	11.5 (7.9-15.5)	34.7 (27.1-42.9)	47.6 (33.7-61.2)	16.6 (12.4-21.2)	32.6 (24-41.8)	42.8 (29.9-54.6)
Idaho	20.9 (16.2-26.1)	43 (34.6-51.2)	56.6 (46-67.4)	27.6 (22.5-32.6)	45.6 (36.7-55)	57.1 (45.6-67.6)
Illinois	22.1 (17.1-27.4)	47.4 (39.6-56)	61.7 (47.9-73.2)	28.9 (24.4-34.1)	50.7 (41.6-59.6)	62.6 (48.8-73)
Indiana	20.1 (15.6-25.1)	47.8 (40-55.9)	62.8 (50.2-73.9)	30.3 (25.4-35.5)	51.8 (43.7-59.5)	66 (53.6-75.4)
Iowa	21.1 (15.6-27.6)	51.8 (43.9-60.1)	67.9 (54.6-77.8)	28.2 (22.9-34.1)	50.6 (42.2-59.5)	67 (54-76.2)
Kansas	19.6 (13.2-27.1)	46.8 (39.6-54.5)	64.1 (49.2-75.1)	25.7 (18.6-33.6)	49.8 (41.6-58)	66.9 (52.1-76.5)
Kentucky	18.1 (13.9-22.7)	50.3 (42.1-58.9)	68.3 (57-77.4)	29.2 (24.5-34.2)	50.8 (42.2-59.3)	65.7 (54.8-74.2)
Louisiana	15.5 (10.2-21.2)	45.8 (37.2-53.7)	59.9 (47.7-71.5)	33.8 (26.9-40.6)	54.5 (45.7-62.4)	68.1 (56-77.7)
Maine	21.3 (15.8-27.1)	40.3 (32.4-48.6)	56.6 (44-68.4)	25.5 (20-31.7)	44.9 (35.5-54.3)	57.7 (45.2-69.3)
Maryland	19.2 (14.7-24.6)	41.5 (34.5-49.4)	55.6 (42.2-67.7)	28.7 (24.1-33.7)	49.3 (41.2-57.5)	61.5 (49.1-72)
Massachusetts	18.3 (13.8-23.7)	40 (32.8-47.6)	51.3 (41-61.8)	23.8 (18.8-28.6)	42.3 (34.3-50.1)	53 (44.1-60.6)
Michigan	22.2 (17.5-27.2)	46.2 (39.1-53.7)	59.6 (48.8-70.4)	31.8 (26.8-37.3)	48.9 (40.8-56.9)	62.4 (49.7-72.6)
Minnesota	21.1 (17-25.7)	46.5 (39.1-53.9)	62.1 (46.5-73.7)	26.5 (22.1-31.1)	44.9 (36.6-53.4)	58.4 (43.4-70.2)
Mississippi	20 (14.1-27.2)	50.8 (42.6-58.5)	64.1 (53.3-75.5)	33.7 (27.2-40.1)	55.6 (47.1-64)	68.5 (56.7-77.2)

65-69 years	Male			Female		
Location	1990	2021	2050	1990	2021	2050
Missouri	20.2 (14.8-26.3)	45.9 (38.4-53.9)	61.9 (48.7-73)	26.6 (21-32.4)	50.7 (41.2-59.8)	64.7 (52.2-75.2)
Montana	17.3 (13-22.4)	42.4 (34.1-50.4)	56.4 (41.6-68.2)	21.3 (16.9-26.3)	39.6 (31.4-48.2)	53.5 (38.4-66.1)
Nebraska	23.1 (18-28.8)	50.3 (42.7-57.7)	67 (52-77.8)	29.8 (24.7-35.6)	50.7 (42.7-58.8)	66.3 (51.7-76.3)
Nevada	20.8 (13.8-29.7)	43.5 (34.1-52.7)	57.1 (42.5-70.3)	29.1 (21-38)	38.7 (29.8-47.9)	50.3 (36.9-62.6)
New Hampshire	20.5 (15-26.3)	42.9 (34.9-51.4)	58.4 (42.1-71.9)	22.8 (17.7-28.7)	41.8 (33.6-50.6)	55.5 (39.7-68.9)
New Jersey	19.7 (13.7-26.2)	40.1 (32.7-47.7)	53.1 (41.4-64.1)	24.7 (18.8-31.4)	44 (34.6-52.4)	55.3 (43-65.2)
New Mexico	13.2 (9-18.3)	38.4 (30.1-46.4)	56.6 (40.4-69.9)	21.3 (16.4-27)	43 (34.1-52.4)	58.6 (42.4-71.7)
New York	20.1 (15.9-24.4)	39 (32.3-45.7)	49.7 (40-60.2)	26.6 (21.6-32.2)	42.6 (33.5-50.7)	51.8 (41.3-63.3)
North Carolina	17.2 (13.4-20.9)	45.5 (38.4-52.9)	59.7 (47.9-70.8)	26.7 (22.3-31.3)	47.2 (39-55)	61.3 (47.7-71.4)
North Dakota	21.5 (16.3-26.7)	49.4 (40.7-57.8)	64.9 (48.8-77)	29.8 (24.3-35.6)	50.9 (42-59.6)	65.6 (50.6-77.2)
Ohio	19.3 (14.8-24.4)	49.7 (41.9-57.5)	65.9 (53.7-76.1)	29.6 (24.7-34.6)	50.9 (42.6-59.2)	66.7 (53.7-76.2)
Oklahoma	15.4 (11.2-20.3)	48.9 (41.3-56.3)	65.7 (52-76.2)	24.3 (18.9-29.7)	49.5 (41.1-57.8)	65.4 (50.5-75.7)
Oregon	19 (14.1-24.3)	39.3 (31.4-48)	51.9 (36.6-65)	24.9 (19.9-29.8)	44.3 (35.9-52.8)	55.6 (42.9-66.2)
Pennsylvania	18.4 (13.8-23.5)	43.8 (36.4-51.2)	57.7 (44.8-69.4)	30.4 (24.9-36.1)	48.2 (39.7-56.3)	59.3 (47-69.4)
Rhode Island	16.7 (12.4-21.8)	40.7 (32.6-49.5)	56.8 (41.1-70.9)	24.1 (19.5-29.2)	44.6 (35.3-53.8)	58.1 (44.3-69.4)
South Carolina	19.8 (15.3-24.8)	45.8 (37.2-54.2)	61.2 (46.8-72.8)	27.8 (23.3-32.7)	51.2 (42.4-60.1)	66.5 (53.2-76.3)
South Dakota	15.3 (11.1-20.4)	47 (38.5-55.3)	62.3 (46.8-74.2)	26.9 (21.3-33.4)	48 (38.8-56.9)	62.1 (47.4-74)
Tennessee	16.9 (13.1-21.1)	45.3 (37.7-53.1)	60.8 (47.6-71.6)	28.2 (23.8-33)	47.5 (38.2-56.1)	60.2 (46.9-71.1)
Texas	20.2 (15.5-25.5)	41 (34-49)	56.3 (41.1-68.3)	27.3 (22.6-32.3)	51.7 (44.2-59.4)	64.7 (50.9-75.2)
Utah	17.5 (13.1-22.6)	41.9 (33.5-50.3)	56.6 (41.6-69.5)	23.7 (19-28.8)	46.4 (38-55.6)	59.9 (46-71.9)
Vermont	16.9 (11.6-23.5)	39.8 (31.8-48.5)	52.8 (38-66.2)	33.9 (27.2-40.4)	41.3 (32.9-50)	53.1 (40.2-65.3)
Virginia	18.8 (13.5-25.2)	44.1 (36.1-52.2)	59.8 (44-72.2)	26.7 (21.6-32.5)	49.7 (41.2-58)	63 (48.2-74.1)
Washington	20.2 (15.7-25.4)	41.3 (34.4-48.6)	52.5 (39.8-64.8)	25 (20.5-30)	41.8 (33.9-49.9)	52 (39.5-62.8)
West Virginia	16.8 (13.1-21.1)	51.6 (43.1-59.6)	70.3 (60.2-78.7)	29.6 (25-34.5)	53.3 (44.5-61.1)	68.9 (58.6-76.8)
Wisconsin	23.5 (18.3-29.1)	48.8 (40.8-57.1)	61.4 (49.7-73)	32.8 (27.7-38.4)	46.9 (37.5-55.9)	60.8 (48.4-71)
Wyoming	20.4 (12.5-29.7)	42.8 (34.1-52)	54.7 (40.7-68.2)	28.1 (19.4-37.7)	44.8 (34.4-54.6)	56.2 (40.6-69.6)

SM Table 5: Prevalence of obesity by 5-year age group and sex in 1990, 2021 and 2050 at the national level, across 50 states and Washington DC

70-74 years	Male			Female		
Location	1990	2021	2050	1990	2021	2050
USA	15.6 (13.9-17.6)	41.2 (36.1-46.6)	54.8 (45.3-63.7)	25 (22.3-27.8)	44.9 (39.1-51.1)	57.4 (47.3-66.2)
Alabama	15.4 (10.9-20.6)	47.9 (39.8-56.4)	64.5 (51.6-74.1)	26.7 (21.8-32.3)	46.7 (38.6-54.8)	63.1 (50.4-73.2)
Alaska	21 (14.1-29.2)	41.4 (33-49.8)	55.3 (39.7-68.4)	24 (15.9-32.6)	45.6 (35.6-55.3)	55.3 (40.4-67.8)
Arizona	13.7 (9.5-18.6)	39.7 (31.8-48.3)	55.3 (40.3-68.3)	17.8 (13.5-22.7)	39.7 (31.1-48.7)	53.7 (39.1-66.3)
Arkansas	15.3 (9.7-22.1)	45.7 (37-54)	62.6 (49-73.8)	25.2 (18.4-32.8)	47.1 (38.8-55.5)	63.8 (50.5-74.5)
California	10.2 (7.5-13.5)	32.7 (26.1-40.3)	44.2 (31-58.7)	23.2 (19.2-27.1)	39 (31.3-46.4)	49.7 (36.2-61.6)
Colorado	11.5 (7.3-16.8)	34.9 (28-42.3)	50.1 (33.6-62.2)	20.5 (14.7-26.8)	37.8 (30.2-46)	50.1 (35.1-62.1)
Connecticut	14.7 (10.2-20.1)	37.5 (29.7-45.6)	52.9 (38.4-65.9)	22.9 (18.1-28.4)	44.4 (35.4-54.2)	58.3 (42.5-71.1)
Delaware	14.7 (9.3-21.5)	46.3 (36.6-55.8)	61.1 (47-73.2)	31.3 (24.2-38.8)	49.5 (40.5-58.7)	63.7 (49.8-75)
District of Columbia	18 (12.8-24.4)	29.8 (21.7-38.3)	39 (26.4-50)	32 (25.2-38.7)	42.3 (33.1-51.6)	51.5 (37.4-64)
Florida	14.3 (10.8-18.3)	43.2 (35.4-51.1)	54.6 (42.3-67.8)	23.2 (18.1-29.1)	41 (32.5-50)	51.5 (39.2-63.5)
Georgia	16.2 (11.6-21.9)	41.4 (33.4-50.3)	57.6 (42.7-69.1)	23.7 (19.1-28.7)	48.4 (40.4-57.1)	63 (49.1-73.5)
Hawaii	11.4 (7.8-15.4)	28.9 (21.1-37.6)	41.3 (28-55.3)	16.1 (12.1-20.7)	28.8 (21.2-37.2)	38.2 (26.4-51.4)
Idaho	15.2 (11.1-20.1)	40 (32-48.7)	53.4 (42.1-65)	22.8 (17.9-27.5)	42.3 (33.6-50.9)	53.9 (44.2-65.1)
Illinois	17.4 (13.2-22.5)	46 (38.1-53.9)	60.6 (46-72.2)	24.2 (19.6-28.9)	46.3 (37.6-54.6)	58.3 (45.1-70.1)
Indiana	18.8 (14.3-23.9)	47.9 (40.5-56.1)	62.8 (51-73.4)	28.7 (23.9-33.7)	50.1 (41.9-58.6)	64.4 (51.9-74.4)
Iowa	19 (14.1-24.7)	47.4 (39.6-55.6)	64 (51.5-73.9)	26.2 (20.6-32.5)	48 (39.7-56.2)	64.3 (51.2-74.9)
Kansas	15.1 (9.7-21.8)	43.1 (35.5-50.7)	60.7 (46-71.7)	23.3 (16.9-30.2)	49 (40.6-57.3)	66.3 (51.1-77.2)
Kentucky	19.2 (14.6-24.1)	43.6 (35.9-52.2)	62.7 (50.9-73.8)	26.3 (21.7-31.1)	45.4 (36.7-53.8)	60.7 (50.1-71.6)
Louisiana	15.2 (9.6-21.9)	48.5 (39.7-57)	62.1 (49.6-74.1)	32.8 (25.7-39.9)	47.9 (39.3-56.2)	62 (49-73.6)
Maine	15.3 (10.9-20.3)	41.4 (33.6-49.6)	57.4 (44.6-69)	21.6 (16.5-27.2)	44.6 (35.2-53.7)	57.5 (44.7-70.1)
Maryland	12.1 (8.7-16.1)	39.8 (32.7-47.3)	53.9 (40.1-66)	25.2 (20.4-30.2)	48.2 (40.2-56.2)	60.6 (48.5-71.5)
Massachusetts	16.8 (12.4-22.2)	38.4 (30.8-46.3)	49.3 (38.7-59.6)	23.1 (18.1-28.4)	42.7 (35.1-50.7)	53.4 (44.7-61.7)
Michigan	17.8 (13.2-23.1)	48.1 (40.2-55.7)	61.3 (50-72.6)	27.4 (22.2-32.9)	51.2 (43.3-58.6)	64.4 (53-73.5)
Minnesota	17.3 (13.4-21.7)	45.9 (37.9-54.4)	61.5 (47-73.6)	24.3 (20.1-28.9)	44.6 (36.7-53.6)	58.4 (43.5-70.7)
Mississippi	15.7 (10.3-21.8)	44.1 (36.3-52.8)	57.9 (46.1-69.5)	29.3 (23.5-35.4)	49.3 (41.3-57.1)	63.3 (51.8-72.6)

70-74 years	Male			Female		
Location	1990	2021	2050	1990	2021	2050
Missouri	16.7 (12.2-21.9)	46.6 (38.7-55)	62.2 (49.6-74)	24.8 (19.9-29.8)	49.2 (40.3-57.9)	63.4 (50.6-74.8)
Montana	13.3 (8.9-18.7)	40.1 (31.9-48.7)	54.1 (39.5-67.6)	20.2 (15.4-25.5)	41 (32.2-50)	55 (40.5-67.8)
Nebraska	19.7 (14.7-25.4)	48.8 (41.2-56.3)	65.8 (51.8-76.7)	23.1 (18.7-28.5)	50 (41.6-57.5)	65.5 (51.2-75.6)
Nevada	21.6 (14-28.8)	38.1 (29.6-46.4)	51.6 (36.6-64.2)	23.9 (17-32.5)	40.8 (31.3-49.9)	52.8 (39.1-65.1)
New Hampshire	16.4 (11.2-22.7)	41.4 (33.5-49.6)	56.8 (41.7-70.3)	22.5 (17.5-28.3)	41.1 (32.5-50.2)	54.6 (39.1-68.1)
New Jersey	11 (6.9-15.6)	37.8 (30.2-45.9)	50.7 (37.6-62.8)	23.9 (17.9-30.5)	44 (35.5-52.8)	55.1 (43.1-65.9)
New Mexico	14.7 (9.8-20.8)	34.3 (26.8-42.1)	52.4 (36.6-66.4)	19.7 (14.7-25.8)	39.6 (30.9-47.9)	55.1 (41.1-68.2)
New York	19 (14.7-24.2)	35 (28.1-41.6)	45.4 (35.1-56.4)	26.7 (21.9-32.2)	43.5 (35.7-52.5)	52.8 (41.8-63.6)
North Carolina	15.2 (11.7-19.2)	43.5 (35.6-51.4)	57.7 (45.5-68.6)	27.8 (23.3-32.5)	48.4 (40-56.1)	62.4 (49.6-72.5)
North Dakota	17.6 (13.2-22.7)	48.7 (41-57.3)	64.3 (49.1-76.5)	30.3 (25-35.7)	50.2 (41.3-59)	65.2 (48.6-75.9)
Ohio	18.7 (14.2-23.7)	46.6 (39.2-54.6)	63.1 (51.8-73.7)	31.5 (26.2-37)	51.2 (43.2-59.1)	67.2 (54.1-76.7)
Oklahoma	11.6 (7.5-16.4)	40.6 (33-48.8)	58.5 (44.4-70.9)	24.4 (19.5-29.7)	49.4 (40.6-57.9)	65 (50.7-75.9)
Oregon	10.3 (7-14.2)	40.3 (31.9-48.7)	52.8 (37.8-66.3)	22.6 (17.8-27.8)	43.2 (35.1-51.7)	54.4 (41.9-66.3)
Pennsylvania	15.9 (12-20.6)	42.3 (35.3-49.8)	55.8 (43-68)	25.1 (20.4-30.3)	48 (40.1-56)	59 (47-69.8)
Rhode Island	13.6 (9.8-18.4)	41.6 (33.1-49.8)	57.4 (42.7-70.6)	24.4 (19.7-29.7)	41.5 (32.7-50)	54.7 (41-67.2)
South Carolina	18.5 (13.9-23.7)	41.9 (33.5-50.3)	57.7 (44-69.4)	25.1 (20.5-30)	47.8 (39.7-57.2)	63.3 (50.8-74.5)
South Dakota	18 (13.2-23.6)	46.5 (37.5-55.2)	62 (47-74.6)	21.8 (16.8-27.7)	48.8 (39.9-57.3)	63.2 (48.2-75.2)
Tennessee	13.1 (9.4-17.2)	43.7 (35.9-52.2)	59.1 (46.8-71.4)	22.8 (18.3-27.6)	46.1 (37.5-55)	59.1 (47.3-70.7)
Texas	18.1 (13.1-24.1)	43.7 (36-51.1)	58.7 (43.9-71.1)	25.3 (20.2-30.8)	46.5 (39.3-54.8)	59.7 (46.1-71.1)
Utah	15.3 (10.8-20.5)	43 (34.8-51.8)	57.6 (43.1-70.5)	22.3 (17.4-27.4)	45.7 (37.1-53.8)	59.2 (45.8-70.1)
Vermont	16.7 (11.2-23.1)	35.8 (27.9-43.9)	48.7 (34.3-62.5)	24.3 (18.1-31.2)	39.4 (31.2-48.4)	50.9 (37.8-65.1)
Virginia	18.4 (12.9-24.7)	40.6 (32.6-48.5)	56.4 (40.6-69.2)	22.9 (17.6-28.7)	45.7 (37.8-54.2)	59.2 (45.1-71)
Washington	20.1 (15.4-25.1)	38 (30.8-44.8)	49.2 (36.1-61.5)	22.2 (17.9-27.1)	43.5 (35.9-51.7)	53.5 (41.3-64.7)
West Virginia	12.9 (9.1-17.5)	46.5 (38.4-55.1)	66.1 (54.5-75.8)	27.6 (22.7-32.7)	51.1 (42.6-59.2)	67 (55.8-75.9)
Wisconsin	19.5 (14.3-25.4)	43.4 (35.4-52.2)	56.4 (44-68.6)	28.3 (22.9-33.9)	46.4 (38-54.9)	60.1 (47.5-71.4)
Wyoming	19 (12.3-28.2)	40.5 (31.8-49.2)	52.1 (38.3-64.7)	27.5 (18.7-38)	43.6 (33.6-52.9)	54.9 (39.4-68)

SM Table 5: Prevalence of obesity by 5-year age group and sex in 1990, 2021 and 2050 at the national level, across 50 states and Washington DC

75-79 years	Male			Female		
Location	1990	2021	2050	1990	2021	2050
USA	12.4 (10.8-14)	37 (32.1-42.1)	51.2 (41.7-59.7)	21.5 (19-24)	40.9 (35-46.8)	53.9 (42.8-62.7)
Alabama	13.6 (9.1-18.7)	37 (29.6-44.8)	54.9 (42.7-65.5)	24.6 (19.5-30.4)	40.2 (32.2-49.1)	57.3 (43.4-68.3)
Alaska	14 (7.5-22.9)	36.5 (27.4-47)	50.8 (35.1-66.2)	32.5 (22.6-43.3)	42.9 (33.5-52)	53.2 (39.5-67)
Arizona	9.3 (5.8-13.7)	33.9 (26.5-42.5)	49.4 (34.8-63.2)	18.7 (13.9-24.2)	36.9 (28-45.7)	51.3 (35.8-64.4)
Arkansas	10.1 (5.7-16.2)	40.3 (32-49)	57.9 (44.2-69.7)	20.8 (14.9-27.3)	43.4 (35.5-51.4)	60.9 (47.3-71.7)
California	11.3 (7.8-15.6)	34.6 (27.8-41.8)	46.4 (33.2-60.4)	18.6 (15-22.3)	37.6 (30.3-44.9)	48.3 (34.8-60.4)
Colorado	8.9 (5.3-13.9)	31.4 (24.7-38.7)	46.8 (30.7-59.2)	21.3 (15.4-27.4)	35.7 (28-43.8)	48.2 (32.9-59.4)
Connecticut	16.3 (11.1-22.5)	37.5 (30-46)	53.3 (38.2-66.7)	20.8 (16-26.9)	40.6 (31.9-49.2)	55.2 (39.3-68.2)
Delaware	11.6 (6.8-18)	37.8 (29-47)	53.1 (39-66.5)	27.6 (21.3-34.9)	43.8 (34.8-52.9)	59 (44.8-70.4)
District of Columbia	19.1 (12.9-26.2)	28.3 (19.9-37.4)	37.1 (24.8-50.3)	25 (18.9-31.5)	45 (35.9-54.9)	54.6 (39.4-66.8)
Florida	11.7 (8.7-15.5)	34.8 (27.6-42.5)	46.6 (34.2-60.6)	18.3 (13.7-23.6)	35.5 (27.1-44.8)	46 (32.8-58.1)
Georgia	14.6 (9.7-20.7)	37 (29.2-45.4)	53.3 (39.4-65.7)	23 (17.9-28.2)	40.7 (33-49.1)	56.5 (42.2-67.1)
Hawaii	9.8 (6.4-14.1)	24.5 (17.6-32.2)	36.4 (23.6-50.2)	15.3 (11.1-20.8)	27.6 (20.7-35.3)	37.6 (25.9-50)
Idaho	9.1 (5.9-13.3)	37 (28.8-45.4)	50.4 (38.8-62.9)	18.1 (14.1-22.9)	38.2 (29.4-47)	50.1 (39.3-61)
Illinois	14 (9.5-19.5)	40.4 (32.2-48.4)	55.5 (40.1-68.4)	23.4 (19-28.2)	43.7 (35-52.5)	55.9 (42.2-67.2)
Indiana	14.2 (9.9-19)	40.3 (32.4-48.1)	56 (44.5-67.8)	25.1 (20.3-29.9)	44.4 (36.3-52.9)	59.5 (45.5-70.6)
Iowa	12.7 (8.7-18.1)	45.4 (36.8-53.4)	62.7 (49.3-73.1)	24.7 (19.2-30.5)	39.2 (30.8-47.6)	56.2 (42-68.5)
Kansas	15.6 (10-22)	39.2 (31.7-46.9)	57.3 (42.5-68.3)	25.7 (18.6-33)	44.6 (36.5-52.6)	63 (46.6-73.4)
Kentucky	10.5 (7-14.9)	37.8 (30.1-46.1)	57.3 (45.4-67.6)	23.5 (19.1-28.4)	38.4 (30.4-46.6)	54.4 (41.9-65.1)
Louisiana	12.3 (7.5-18.1)	39.1 (31-47.3)	53.3 (39.6-66.7)	24.9 (18.9-31.2)	46.9 (38.4-55.3)	61.6 (49-71.6)
Maine	11.7 (7.6-16.6)	34.8 (27-42.3)	51.1 (38.9-62.7)	23.8 (18-30.6)	41.4 (32.8-49.9)	54.5 (41.3-65.8)
Maryland	11.4 (7.4-16.1)	35.3 (27.9-42.8)	49.6 (36.2-61.8)	20.4 (15.7-26)	45 (37.2-52.9)	57.9 (45-69.2)
Massachusetts	13.1 (8.6-18.8)	34.7 (27.5-42.2)	45.7 (35.1-56.6)	21.4 (16.6-26.5)	40.6 (32.2-48.5)	51.6 (43.7-59.8)
Michigan	16.2 (11.4-21.2)	40.7 (33.2-48.3)	54.7 (43.8-65.8)	24.6 (19.4-30.2)	46.4 (38.5-54.9)	60.2 (47.7-70.7)
Minnesota	14.6 (11-18.8)	40.3 (32.8-48)	56.4 (40.2-68.7)	22.5 (18.3-26.8)	41.9 (33.6-50.4)	55.9 (40.2-67.9)
Mississippi	13.6 (8.8-19.6)	37 (29.5-45.2)	51.3 (39.3-63.3)	22.9 (17.6-29.3)	41 (32.8-48.7)	55.7 (41.7-67)

75-79 years	Male			Female		
Location	1990	2021	2050	1990	2021	2050
Missouri	17.3 (12.4-23.1)	35.1 (27.3-42.8)	51.4 (37.2-63.8)	22.5 (18-27.7)	43.2 (34-51.8)	58.1 (44.3-69)
Montana	14.1 (9.5-19.7)	35.7 (28-43.6)	49.7 (35.7-62.4)	19.6 (15-24.7)	39.9 (31.1-48.1)	53.9 (39.9-66.7)
Nebraska	13.1 (9-17.9)	45.9 (38.2-53.4)	63.3 (48.8-73.8)	23.3 (18.4-29.2)	42.9 (34.6-50.8)	59.5 (43.8-70.5)
Nevada	11.7 (6.4-18.7)	33.9 (25.6-42.7)	47.7 (33.8-61.7)	20 (13.2-28)	38.2 (28.9-47.8)	50.1 (36.1-61.7)
New Hampshire	12.1 (7.3-18.2)	34.3 (26.4-42.3)	50 (33.8-65.3)	24.6 (18.7-31)	40.2 (31.2-49.7)	53.7 (38.1-67.8)
New Jersey	13.4 (8.2-20.1)	35 (27-43.1)	47.6 (35.6-59)	17.8 (12.6-24.4)	38.7 (30.7-46.8)	50.2 (37.7-61.1)
New Mexico	11.6 (7.3-16.8)	31.7 (24.3-40.2)	49.7 (34.5-64.4)	15.2 (11-20.3)	35.9 (28.1-44.4)	52 (37.4-65)
New York	12.2 (8.4-17.1)	34.4 (28-41.4)	45.1 (34.9-56.6)	22.1 (17.4-27.1)	40.7 (32.8-48.5)	50.4 (40-61.2)
North Carolina	11.4 (7.8-15.8)	34.4 (26.9-42.5)	49.1 (36.3-61.3)	23.8 (19.5-28)	44.7 (36.4-53)	59.6 (46.5-69.3)
North Dakota	15.6 (11-20.9)	42.8 (35.3-51.2)	58.9 (42.8-72)	25 (19.9-30.6)	43.7 (34.6-52.6)	59.7 (42.9-70.6)
Ohio	12 (8.1-16.6)	42.2 (34.5-49.5)	59.2 (47-70.2)	19.6 (15-24.4)	45.7 (37.8-53)	62.7 (48.6-72.4)
Oklahoma	10.5 (7-15)	40.3 (32.3-47.9)	58.1 (43.7-70.6)	18.7 (14-24.5)	42.1 (34.2-50.6)	59 (44-70.3)
Oregon	8.1 (5.2-12.1)	34.4 (26.3-42.9)	46.7 (33-60.2)	20.4 (16-25.5)	38.5 (30.9-46.7)	50.1 (35.6-61.7)
Pennsylvania	10.8 (7.3-15.3)	40.2 (32.5-48.3)	54.2 (41.8-65.7)	25.8 (20.6-31)	43.9 (36.1-51.8)	55.6 (44.3-66.5)
Rhode Island	11.3 (7.6-16.1)	35.4 (27.7-43.7)	51.5 (36.4-65.4)	24.6 (19.3-29.9)	36.8 (28.3-45.6)	50.1 (36.2-63.5)
South Carolina	11.7 (7.4-17.2)	37.6 (29.6-45.9)	53.7 (40.3-65.1)	27.3 (21.9-33.2)	41.3 (32.6-50)	57.7 (43.6-69.1)
South Dakota	16.7 (11.5-22.9)	39.3 (30.4-47.9)	55.5 (39.3-68.9)	26 (20.3-31.8)	36 (27.8-45.2)	50.9 (35.7-64.3)
Tennessee	9.6 (6.4-13.8)	38.4 (30.7-46.5)	54.7 (41.5-67.1)	23.2 (18.5-27.9)	40.4 (32.2-49.5)	54.3 (41-66.2)
Texas	14.2 (9.2-20.1)	41.2 (33.9-48.7)	56.5 (43.2-68.8)	16 (11.7-20.9)	43.8 (35.9-51.1)	57.4 (42.9-68.3)
Utah	12.1 (8.1-16.7)	38.4 (30.5-46.7)	53.2 (38.4-67.2)	20.3 (15.4-25.2)	40.8 (32.3-49.1)	54.7 (39.5-66.6)
Vermont	13.7 (8.9-19.8)	35.2 (27.4-43.4)	48.1 (34-61.5)	19.9 (14.3-26)	41.1 (33.5-49.8)	53 (40.4-65.4)
Virginia	9.6 (5.5-14.9)	35 (27.4-42.8)	51 (36-65.2)	26.2 (20.3-32.2)	39.9 (32-47.6)	53.8 (38.6-65.7)
Washington	11.4 (7.7-15.9)	36.9 (30.1-44.2)	48.2 (35.9-60.5)	19.4 (15-23.9)	39.1 (31.6-47)	49.4 (37.4-61.4)
West Virginia	9.8 (6.4-14.1)	39.4 (30.9-47.7)	60 (48.5-70.2)	24.9 (20.2-29.8)	46.7 (38.5-54.9)	63.8 (53.4-72.1)
Wisconsin	11.4 (7.3-16.4)	38.8 (30.6-47.2)	52.1 (40.7-64.7)	24.2 (19.2-29.4)	44.2 (35.7-53)	58.5 (44.6-69.2)
Wyoming	15.4 (9-23.6)	36.2 (28-44.3)	48.2 (34.8-61.3)	24.6 (16.2-34.6)	37.7 (28.7-47.3)	49.3 (34.5-63.7)

SM Table 5: Prevalence of obesity by 5-year age group and sex in 1990, 2021 and 2050 at the national level, across 50 states and Washington DC

80+ years	Male			Female		
Location	1990	2021	2050	1990	2021	2050
USA	8.5 (7.4-9.7)	25 (21.4-29.1)	37.8 (28.7-46.4)	14.6 (13.1-16.2)	29.6 (24.6-35)	42.5 (32.4-51.3)
Alabama	6.4 (3.6-10.2)	27.5 (21-35.3)	44.4 (32.1-56.2)	13.2 (9.4-17.7)	30.7 (23.7-38.1)	48.1 (34.2-60.1)
Alaska	9 (4.2-16.5)	29.5 (21-37.6)	43.1 (27.8-57.6)	18.7 (12.1-26.9)	28.8 (20.4-37.6)	38.9 (25.7-52.8)
Arizona	9.9 (5.4-15.4)	19.2 (14-25.2)	32.2 (19-45.1)	11.3 (7.4-15.9)	27.2 (20.3-35)	41.2 (26.4-54.7)
Arkansas	4.8 (2.4-8.7)	25.4 (19.1-32.7)	42.1 (29-55.4)	13.4 (8.9-18.8)	29.5 (22.8-36.7)	47.1 (33.6-58.4)
California	10.6 (7-15)	21.8 (16.1-27.5)	31.9 (20.1-44.9)	12.9 (9.7-16.7)	24.6 (19.2-30.8)	34.7 (23-46)
Colorado	2.1 (1-3.9)	20.1 (14.9-26.1)	33.2 (18.7-45.1)	13 (8.5-18.4)	25.4 (19.2-32.3)	37.6 (23.3-48.9)
Connecticut	8.2 (4.7-13.1)	24.9 (18.9-32.3)	39.3 (24.5-53.2)	14 (9.3-19.8)	30 (22.7-38.1)	44.3 (29.2-58.6)
Delaware	8 (4.2-13.5)	29.7 (21.6-38)	44.4 (30.2-58.5)	19.7 (13.9-26.2)	34.4 (26.4-42.7)	49.7 (35.6-62.6)
District of Columbia	10.5 (5.7-16.5)	23.2 (15.2-31)	31.5 (18.9-42.8)	15 (10.1-20.7)	32.8 (24.4-41.9)	42.4 (28.8-55.5)
Florida	5.7 (3.4-8.6)	24 (18.3-30.3)	34.3 (23.1-48)	12.1 (8.3-16.8)	26.2 (19.5-34.2)	36.7 (25.4-48.9)
Georgia	8.2 (4.6-13.7)	24.8 (18.8-31.5)	39.8 (26.7-52.4)	15.7 (11-21.1)	30.5 (23.6-37.5)	46.4 (31.5-57.7)
Hawaii	4.5 (2.2-7.7)	18.1 (12.5-24.7)	28.9 (16.8-42.7)	7.5 (4.7-11.5)	22.2 (16-29.5)	31.7 (20.6-43.9)
Idaho	4.7 (2.6-7.8)	24.7 (18.4-32)	36.9 (26.4-48.4)	15.1 (10.8-20)	29.9 (22.9-37.7)	41.7 (32-52.4)
Illinois	10.9 (6.7-16.2)	28.4 (21.7-35.5)	42.7 (28.3-57.2)	18.9 (14.8-23.7)	31.6 (25-39.4)	44.5 (30.8-56.6)
Indiana	6.4 (3.6-10.1)	28.7 (22.2-36.2)	43.6 (31.1-56.1)	16.9 (13.1-21.5)	33.5 (26.9-41.2)	49 (35.7-61)
Iowa	6.6 (3.7-10.2)	31.7 (24.5-39.2)	48.8 (35.6-60.1)	17.5 (12.9-22.6)	33.7 (26.8-41.8)	51.5 (37.4-62.9)
Kansas	6.7 (3.5-11.1)	25 (19.8-31.7)	42.1 (28-54.7)	10.8 (6.9-16.2)	31.1 (25-38.3)	50.2 (33.9-62)
Kentucky	6.9 (4.1-10.4)	30.1 (23.4-37.9)	49 (36.1-59.4)	12.5 (8.9-16.4)	30.7 (23.8-37.7)	47.1 (34.9-57.6)
Louisiana	9.4 (5-15.4)	26.2 (19.3-33.6)	39.5 (27.6-54.4)	22.4 (16.2-29.5)	33.8 (26.3-41.4)	49.2 (36.2-61.8)
Maine	5.2 (2.8-8.7)	24.3 (18-31.6)	39.1 (26.2-50.5)	16.2 (11.6-21.8)	30.6 (23-39)	43.6 (30.8-56.7)
Maryland	8.5 (4.9-13.1)	23.1 (17.4-29.7)	35.9 (23.1-48.1)	12.6 (8.8-17.4)	31 (24.3-38.5)	43.7 (30.8-56.2)
Massachusetts	7.1 (4.2-11.5)	20.9 (15.4-27.5)	30.3 (21.2-41.3)	13.1 (9.2-17.2)	29.7 (22.9-37.6)	40.3 (31.6-49.2)
Michigan	15.2 (9.8-21.5)	29.8 (23.2-36.7)	43 (31.6-54.6)	15.8 (11.6-20.7)	33.2 (26.2-40.2)	47.4 (34.7-59.2)
Minnesota	10.8 (7.2-15.3)	30 (23.5-37.6)	45.9 (30.3-59)	17.2 (13.6-21)	29.5 (22.6-36.8)	43.4 (28-55)
Mississippi	10.4 (5.9-16.6)	24.9 (18-32.1)	37.5 (25.4-50.5)	18.8 (13.6-24.8)	30.9 (23.7-38.4)	46 (33.1-57.7)

80+ years	Male			Female		
Location	1990	2021	2050	1990	2021	2050
Missouri	8.1 (4.5-12.7)	25.3 (19.2-32.2)	40.3 (27.2-53.5)	14.7 (10.7-19.7)	32.1 (24.7-39.9)	47.3 (33.4-59.1)
Montana	10.8 (6.6-16.4)	30.2 (23.3-37.5)	43.8 (30.1-56)	15.1 (10.8-19.8)	29.9 (22.6-38.1)	43.8 (29.2-57.2)
Nebraska	6.1 (3.5-9.9)	29.7 (23.1-36.9)	47.5 (32.2-59.2)	17.1 (13-21.7)	32.7 (26.1-40.2)	49.8 (33.5-61.4)
Nevada	4.6 (2.1-9)	21.5 (15.2-29.3)	33.4 (20.6-47.4)	8.2 (4.4-13.1)	28.4 (20.9-36.7)	40.7 (27.2-53.1)
New Hampshire	3 (1.4-5.6)	23.8 (17.2-30.9)	38.2 (23.2-52.7)	16.1 (10.9-22)	30.5 (23.2-38.6)	44.2 (29.2-58.1)
New Jersey	6 (3.1-10.2)	23.5 (17.2-30.4)	35.1 (23.8-47.9)	18.1 (12.4-24.8)	26.5 (20-33.5)	37.3 (26.4-48.6)
New Mexico	4.2 (2.1-7.3)	18.8 (13.4-25)	34.4 (21.2-47.8)	8.6 (5.2-13.3)	24.6 (17.6-32.8)	40 (25.7-54.7)
New York	7.1 (4.3-10.6)	22.2 (16.9-28.1)	31.5 (22.9-42.8)	15.1 (11.3-19.5)	27.2 (20.3-34.5)	36.2 (25.6-47.3)
North Carolina	7.4 (4.5-11.3)	28.5 (22-35.5)	42.2 (30.4-54)	13.7 (10.1-17.9)	30.2 (23.3-37.4)	45.4 (31.4-56.8)
North Dakota	16.6 (11.3-23)	32.2 (24.9-40.1)	48.5 (32.3-61.6)	15.2 (11.3-19.6)	32 (24.3-40)	48.3 (31-61)
Ohio	7.3 (4.3-11.7)	27.8 (21.8-34.8)	44.3 (32-56.2)	10.6 (7.3-14.4)	33.6 (27.5-41.3)	51.5 (38.6-62.6)
Oklahoma	4.5 (2.4-7.7)	26.8 (20.9-34)	44 (30-58.3)	12.2 (8.5-16.5)	30.1 (23.5-37.2)	47.5 (32.9-59.9)
Oregon	2.9 (1.5-5.3)	24.6 (18.2-32.5)	35.9 (23.2-50.7)	13.7 (9.7-18.2)	28.8 (21.7-36.4)	40.2 (27-52.2)
Pennsylvania	10.4 (6.6-15.5)	26 (20.2-32.7)	39 (26.6-51.4)	19.2 (14.6-24.2)	32.4 (24.8-39.7)	44.2 (31.6-55.9)
Rhode Island	7.3 (4-11.5)	22.9 (16.5-30.4)	37.5 (22.8-53.3)	12.1 (8.3-16.4)	30.2 (22.7-38.2)	43.9 (29.3-56.7)
South Carolina	13.3 (8.2-19)	24.9 (18.5-32.3)	39.7 (27.1-53.5)	15.1 (10.8-20)	34.4 (26.5-42.7)	51.2 (36.5-64)
South Dakota	14.9 (9.5-20.7)	32.4 (24.8-40.7)	48.2 (32.6-62.3)	15.5 (11-20.5)	28.8 (21.7-37)	43.6 (28.4-56.9)
Tennessee	12.8 (8-18.8)	22.1 (15.9-28.9)	36.2 (23.7-48.9)	12.1 (8.7-16.1)	26.9 (20-34.3)	40.4 (27.1-53.1)
Texas	9.3 (5.3-14.5)	26.3 (20.8-32.2)	40.7 (27.1-53.3)	13.8 (9.7-18.8)	32.8 (25.9-40.3)	47 (33.2-58.8)
Utah	4.5 (2.3-7.8)	25 (18.7-32.6)	38.8 (25.1-53.2)	13.2 (9.2-17.6)	26.2 (19.7-33.8)	39.7 (25.3-52.2)
Vermont	6 (3.2-10.1)	25.9 (19.3-33.6)	38.1 (24.9-53.2)	12.1 (7.8-17.2)	34.3 (26.6-42.3)	46 (32.4-58.8)
Virginia	6.7 (3.2-11.5)	24.2 (17.5-31.2)	39 (23.9-51.9)	12.8 (8.3-18.1)	33.4 (25.9-40.9)	48.1 (32.5-60.9)
Washington	13.9 (8.8-19.9)	26.2 (20.6-32.5)	36.7 (24.5-49.5)	13.2 (9.4-17.5)	30.9 (24.5-37.7)	41.2 (29.6-52.6)
West Virginia	8.7 (5.3-13.4)	27.4 (20.3-35.2)	47.1 (34.9-58.5)	18 (13.8-22.3)	32.1 (25-40.3)	49.6 (36.6-61)
Wisconsin	11.9 (7-18.1)	30.8 (23.6-38.7)	43.5 (32.7-56)	16 (11.5-21.3)	33 (25.3-40.6)	47.4 (34.5-59)
Wyoming	7.4 (3.6-12.9)	28.5 (21.5-36.2)	39.8 (26.8-52.6)	14.4 (8.6-21.3)	27.7 (20-36.5)	38.9 (24.7-54.7)

SM Table 6: Number of population with overweight and obesity among adolescents ages 15-24 and adults ages 25+ by sex in 1990, 2021 and 2050 at the national level, across 50 states and Washington DC

	Male						Female					
	15-24			25+			15-24			25+		
Location	1990	2021	2050	1990	2021	2050	1990	2021	2050	1990	2021	2050
USA	6,130,337	10,459,862	11,935,500	45,822,389	85,015,697	103,588,829	4,837,844	10,916,300	12,789,666	41,317,961	86,638,717	109,516,932
Alabama	100,191	172,804	200,291	738,167	1,290,601	1,483,390	77,715	195,690	226,815	692,618	1,411,437	1,681,325
Alaska	16,142	24,396	32,727	105,188	196,100	273,548	12,068	24,211	34,717	78,426	167,364	254,619
Arizona	81,998	242,306	272,644	612,972	1,831,179	2,358,925	55,752	259,815	314,010	536,589	1,829,512	2,486,798
Arkansas	61,769	102,330	116,723	437,022	761,732	930,331	52,665	118,134	137,522	418,948	807,373	1,021,385
California	755,456	1,224,535	1,370,140	5,297,892	9,861,708	12,850,387	535,086	1,166,199	1,325,272	4,367,184	9,789,781	13,285,604
Colorado	67,352	157,697	159,547	551,904	1,409,408	1,647,620	51,589	161,252	169,549	468,162	1,353,463	1,694,569
Connecticut	72,645	102,832	116,797	624,788	955,911	1,163,513	48,633	104,534	127,661	522,026	939,229	1,201,967
Delaware	17,135	27,785	31,399	127,746	258,144	296,055	15,132	35,943	43,658	119,706	277,455	328,395
District of Columbia	15,179	13,150	17,583	100,634	157,675	182,746	16,124	17,350	20,736	123,768	183,468	213,482
Florida	272,822	602,111	666,637	2,499,810	5,690,907	6,215,410	219,284	644,971	735,248	2,284,013	5,729,499	6,530,187
Georgia	174,724	351,712	380,079	1,155,657	2,613,053	3,360,075	147,625	394,326	442,200	1,035,328	2,890,315	3,780,217
Hawaii	32,701	42,624	54,277	188,690	368,936	462,131	18,790	38,510	52,835	143,879	327,874	443,110
Idaho	20,875	58,995	68,052	177,291	464,692	588,948	17,829	68,194	81,548	150,051	436,167	574,328
Illinois	265,099	389,205	461,821	2,123,606	3,294,661	4,184,832	236,305	414,924	484,889	1,936,735	3,397,354	4,465,056
Indiana	139,394	233,134	280,789	1,054,301	1,726,447	2,110,849	112,605	253,122	304,614	993,124	1,800,247	2,296,803
Iowa	65,320	104,761	129,642	540,250	857,897	1,054,048	51,277	116,446	153,223	493,240	835,473	1,076,910
Kansas	65,860	106,810	132,452	460,050	757,486	995,497	52,310	112,364	143,006	400,279	749,030	1,039,253
Kentucky	94,349	152,511	182,449	679,954	1,180,345	1,375,163	88,013	168,421	207,342	652,776	1,208,854	1,466,949
Louisiana	108,138	146,380	168,349	739,136	1,181,705	1,413,937	110,382	172,878	197,749	755,094	1,258,107	1,566,788
Maine	30,531	34,979	37,819	236,055	375,347	387,717	25,404	38,165	43,912	207,451	373,421	396,713
Maryland	116,817	170,835	202,217	873,200	1,570,296	1,905,038	84,575	189,177	226,557	805,079	1,696,882	2,098,238
Massachusetts	157,189	186,428	182,548	1,134,117	1,791,761	1,958,558	103,334	185,188	202,713	934,918	1,799,175	2,097,885
Michigan	259,540	323,534	358,491	1,784,330	2,645,455	2,937,512	212,478	348,161	393,838	1,657,790	2,783,700	3,215,797
Minnesota	100,499	170,314	212,025	836,593	1,518,721	1,854,952	64,784	171,880	222,738	687,440	1,432,189	1,859,672
Mississippi	73,023	103,735	112,848	446,221	738,458	902,483	73,325	125,263	140,792	477,072	827,043	1,028,756
Missouri	116,508	192,794	233,840	954,141	1,608,573	1,961,066	103,389	214,022	259,614	899,368	1,627,368	2,058,442
Montana	17,976	30,596	34,710	146,611	292,146	316,146	13,343	31,676	39,337	122,982	270,376	311,254
Nebraska	40,229	66,309	85,753	298,079	515,761	672,500	25,590	67,347	93,550	271,765	503,523	680,975
Nevada	31,491	100,131	114,681	244,553	823,318	1,062,183	23,986	97,080	117,679	186,990	780,083	1,088,705
New Hampshire	24,916	40,326	44,468	209,332	385,960	402,451	20,698	36,985	41,774	165,934	364,577	417,229
New Jersey	179,570	267,637	321,530	1,487,526	2,408,719	2,879,926	144,375	251,517	308,363	1,295,478	2,392,248	2,938,565
New Mexico	33,934	72,982	84,087	243,304	537,119	679,418	30,139	69,251	80,541	218,768	550,622	729,902
New York	444,869	578,990	692,342	3,272,307	5,062,257	6,148,456	326,073	574,399	697,703	3,066,165	5,225,173	6,379,980
North Carolina	185,196	347,341	388,107	1,230,431	2,632,896	3,300,512	154,896	359,245	402,097	1,158,920	2,882,081	3,680,899
North Dakota	17,075	25,711	30,491	126,280	217,341	245,086	10,998	24,838	33,875	106,010	195,489	237,229
Ohio	241,955	370,619	438,715	2,045,639	3,090,984	3,555,278	219,424	416,366	501,857	1,914,768	3,231,660	3,874,514
Oklahoma	79,037	143,798	164,621	572,330	1,021,524	1,262,070	58,969	156,741	185,452	524,693	1,038,861	1,338,550
Oregon	59,505	120,465	132,622	547,342	1,105,753	1,213,672	54,998	126,996	134,298	469,973	1,116,371	1,279,465
Pennsylvania	285,916	370,040	417,311	2,297,479	3,448,238	3,896,886	228,797	385,040	443,273	2,298,264	3,514,702	4,166,190
Rhode Island	26,076	32,321	32,260	189,127	292,803	320,393	16,629	31,891	32,648	160,667	297,072	335,452
South Carolina	102,751	170,376	186,246	635,244	1,319,271	1,520,423	80,100	184,801	203,398	612,094	1,424,680	1,695,234
South Dakota	16,629	26,779	34,927	132,078	232,705	286,059	12,637	29,704	42,169	113,515	220,648	291,529
Tennessee	117,269	224,215	243,075	882,294	1,755,230	1,999,099	101,528	262,875	291,251	836,162	1,904,766	2,234,330
Texas	442,035	1,156,254	1,258,465	2,999,265	7,358,257	9,885,810	358,177	1,150,622	1,282,743	2,644,157	7,487,355	10,305,233
Utah	35,343	115,194	142,469	252,843	741,138	1,148,344	29,321	122,294	160,587	215,576	705,547	1,145,357
Vermont	13,534	17,313	17,798	103,298	170,628	178,013	11,586	18,343	20,444	90,030	162,757	180,497
Virginia	162,710	266,248	322,885	1,134,110	2,239,110	2,808,784	109,205	278,432	342,414	979,093	2,318,548	3,024,046
Washington	110,132	219,615	233,086	897,783	2,008,745	2,312,135	85,266	219,460	240,839	756,684	1,942,106	2,328,714
West Virginia	46,919	57,292	64,955	346,819	501,291	534,157	38,138	58,485	67,416	347,918	507,080	568,279
Wisconsin	123,239	181,112	214,244	963,675	1,594,515	1,896,355	87,838	203,611	262,234	851,042	1,535,268	1,944,369
Wyoming	10,242	18,716	24,556	86,761	154,295	198,849	8,922	17,790	23,791	70,976	136,114	189,110
