# Supplementary material for: National-level and state-level prevalence of overweight and obesity among children, adolescents, and adults in the USA, 1990–2021, and forecasts up to 2050
Source: Lancet. 2024 Dec 7;404(10469):2278–98. doi: 10.1016/S0140-6736(24)01548-4 (PMC11694015; doi:10.1016/S0140-6736(24)01548-4)
Supplement: Supplementary appendix 3 [file mmc3.pdf]

THE LANCET

## Supplementary appendix 3

This appendix formed part of the original submission and has been peer reviewed. We post it as supplied by the authors.

Supplement to: GBD 2021 US Obesity Forecasting Collaborators. National-level and state-level prevalence of overweight and obesity among children, adolescents, and adults in the USA, 1990–2021, and forecasts up to 2050. *Lancet* 2024; published online Nov 14. https://doi.org/10.1016/S0140-6736(24)01548-4.

# Appendix 3: Authorship appendix to "National and state-level prevalence of overweight and obesity in the USA among children, adolescents, and adults, 1990–2021 and forecasts 2022–2050"

This appendix provides further authorship detail for "National and state-level prevalence of overweight and obesity in the USA among children, adolescents, and adults, 1990–2021 and forecasts 2022–2050"

## Table of Contents


Appendix 3: Authorship appendix to "National and state-level prevalence of overweight and obesity in the USA among children, adolescents, and adults, 1990–2021 and forecasts 2022–2050" ..... 1

**GBD 2021 US Obesity Forecasting Collaborators ..... 2**

**Affiliations ..... 3**

**Authors' Contributions ..... 12**

Managing the overall research enterprise ..... 12

Writing the first draft of the manuscript ..... 12

Primary responsibility for applying analytical methods to produce estimates ..... 12

Primary responsibility for seeking, cataloguing, extracting, or cleaning data; designing or coding figures and tables ..... 13

Providing data or critical feedback on data sources ..... 13

Developing methods or computational machinery ..... 13

Providing critical feedback on methods or results ..... 13

Drafting the work or revising it critically for important intellectual content ..... 15

Managing the estimation or publications process ..... 16

## GBD 2021 US Obesity Forecasting Collaborators

Marie Ng*, Xiaochen Dai*, Rebecca M Cogen*, Michael Abdelmasseh, Arash Abdollahi, Auwal Abdullahi, Richard Gyan Aboagye, Hana J Abukhadijah, Temitayo Esther Adeyeoluwa, Aanuoluwapo Adeyimika Afolabi, Danish Ahmad, Noah Ahmad, Ayman Ahmed, Syed Anees Ahmed, Mohammed Ahmed Akkaif, Ashley E Akrami, Syed Mahfuz Al Hasan, Omar Al Ta'ani, Fares Alahdab, Ziyad Al-Aly, Wafa A Aldhaleei, Abdelazeem M Algammal, Waad Ali, Akram Al-Ibraheem, Saleh A Alqahatni, Rami H Al-Rifai, Najim Z Alshahrani, Mohammad Al-Wardat, Hany Aly, Walid A Al-Zyoud, Sohrab Amiri, Abhishek Anil, Jalal Arabloo, Aleksandr Y Aravkin, Ali Ardekani, Demelash Areda, Mubarek Yesse Ashemo, Alok Atreya, Sina Azadnajafabad, Shahkaar Aziz, Peter S Azzopardi, Giridhara Rathnaiah Babu, Atif Amin Baig, Abdulaziz T Bako, Kannu Bansal, Till Winfried Bärnighausen, Mohammad-Mahdi Bastan, Maryam Bemanalizadeh, Azizullah Beran, Habtamu B Beyene, Sonu Bhaskar, Cem Bilgin, Archie Bleyer, Hamed Borhany, Edward J Boyko, Dejana Braithwaite, Dana Bryazka, Raffaele Bugiardini, Yasser Bustanji, Zahid A Butt, Mehtap Çakmak Barsbay, Ismael Campos-Nonato, Francieli Cembranel, Ester Cerin, Pamela Roxana Chacón-Uscamaita, Eeshwar K Chandrasekar, Vijay Kumar Chattu, An-Tian Chen, Guangjin Chen, Gerald Chi, Patrick R Ching, So Mi Jemma Cho, Dong-Woo Choi, Bryan Chong, Sheng-Chia Chung, Zinhle Cindi, Karly I Cini, Alyssa Columbus, Rosa A S Couto, Michael H Criqui, Natalia Cruz-Martins, Omar B Da'ar, Omid Dadras, Zhaoli Dai, Samuel Demissie Darcho, Nihar Ranjan Dash, Hardik Dineshbhai Desai, Samath Dhamminda Dharmaratne, Daniel Diaz, Michael J Diaz, Thanh Chi Do, Mahsa Dolatshahi, Mario D'Oria, Ojas Prakashbhai Doshi, Rajkumar Prakashbhai Doshi, Robert Kokou Dowou, John Dube, Dorothea Dumuid, Arkadiusz Marian Dziedzic, Abdel Rahman E'mar, Rabie Adel El Arab, Ibrahim Farahat El Bayoumy, Muhammed Elhadi, Chadi Eltaha, Luca Falzone, Hossein Farrokhpour, Patrick Fazeli, Valery L Feigin, Ginenus Fekadu, Nuno Ferreira, Florian Fischer, Kate Louise Francis, Muktar A Gadanya, Miglas Welay Gebregergis, Delaram J Ghadimi, Ehsan Gholami, Mahaveer Golechha, Davide Golinelli, Philimon N Gona, Mahdi Gouravani, Ayman Grada, Ashna Grover, Avirup Guha, Rahul Gupta, Parham Habibzadeh, Nils Haep, Aram Halimi, Md. Kamrul Hasan, Md Saquib Hasnain, Simon I Hay, Wen-Qiang He, Jeffrey J Hebert, Mehdi Hemmati, Yuta Hiraike, Nguyen Quoc Hoan, Sorin Hostiuc, Chengxi Hu, Junjie Huang, Hong-Han Huynh, Md. Rabiul Islam, Sheikh Mohammed Shariful Islam, Louis Jacob, Abel Joseph, Sivesh Kathir Kamarajah, Kehinde Kazeem Kanmodi, Rami S Kantar, Yeganeh Karimi, Sina Kazemian, Mohammad Jobair Khan, Muhammad Shahzeb Khan, Praval Khanal, Shaghayegh Khanmohammadi, Khaled Khatab, Moawiah Mohammad Khatatbeh, Moein Khormali, Jagdish Khubchandani, Sylvia Kiconco, Min Seo Kim, Ruth W Kimokoti, Adnan Kisa, Mukhtar Kulimbet, Vijay Kumar, Satyajit Kundu, Om P Kurmi, Hanpeng Lai, Nhi Huu Hanh Le, Munjae Lee, Seung Won Lee, Wei-Chen Lee, An Li, Wei Li, Stephen S Lim, Jialing Lin, Paulina A Lindstedt, Xiaofeng Liu, Justin Lo, José Francisco López-Gil, Giancarlo Lucchetti, Lisha Luo, Jay B Lusk, Elham Mahmoudi, Elaheh Malakan Rad, Yosef Manla, Ramon Martinez-Piedra, Yasith Mathangasinghe, Fernanda Penido Matozinhos, Steven M McPhail, Hadush Negash Meles, George A Mensah, Sultan Ayoub Meo, Tomislav Mestrovic, Irmina Maria Michalek, GK Mini, Mohammad Mirza-Aghazadeh-Attari, Gabriele Mocciaro, Jama Mohamed, Mouhand F H Mohamed, Nouh Saad Mohamed, Ameen Mosa Mohammad, Shafiu Mohammed, Ali H Mokdad, Kaveh Momenzadeh, Sara Momtazmanesh, Fateme Montazeri, Maziar Moradi-Lakeh, Shane Douglas Morrison, Rohith Motappa, Erin C Mullany, Christopher J L Murray, Pirouz Naghavi, Soroush Najdaghi, Delaram Narimani Davani, Gustavo G Nascimento, Zuhair S Natto, Dang H Nguyen, Hau Thi Hien Nguyen, Phat Tuan Nguyen, Van Thanh Nguyen, Yeshambel T Nigatu, Nasrin Nikravangolsefid, Syed Toukir Ahmed Noor, Fred Nugen, Ogochukwu Janet Nzoputam, Bogdan Oancea, Erin M O'Connell, Sylvester Reuben Okeke, Andrew T Olagunju, Omotola O Olasupo, Abdulhakeem Abayomi Olorukooba, Samuel M Ostroff, Abderrahim

Oulhaj, Mayowa O Owolabi, Mahesh Padukudru P A, Romil R Parikh, Seoyeon Park, Sungchul Park, Ava Pashaei, Gavin Pereira, Hoang Nhat Pham, Tom Pham, Anil K Philip, Jalandhar Pradhan, Pranil Man Singh Pradhan, Nicolaas P Pronk, Jagadeesh Puvvula, Seyedeh Niloufar Rafiei Alavi, Catalina Raggi, Muhammad Aziz Rahman, Bita Rahmani, Mohammad Rahmanian, Shakthi Kumaran Ramasamy, Chhabi Lal Ranabhat, Sowmya J Rao, Sina Rashedi, Ahmed Mustafa Rashid, Elrashdy Moustafa Mohamed Redwan, Taeho Gregory Rhee, Monica Rodrigues, Jefferson Antonio Buendia Rodriguez, Cameron John Sabet, Siamak Sabour, Umar Saeed, Dominic Sagoe, Mohamed A Saleh, Vijaya Paul Samuel, Abdallah M Samy, Aswini Saravanan, Monika Sawhney, Susan M M Sawyer, Nikolaos Scarmeas, Markus P Schlaich, Art Schuermans, Sadaf G Sepanlou, Allen Seylani, Mahan Shafie, Nilay S Shah, Muhammad Aaqib Shamim, Mohammad Ali Shamshirgaran, Sadaf Sharfaei, Amin Sharifan, Anupam Sharma, Manoj Sharma, Aziz Sheikh, Rekha Raghuveer Shenoy, Premalatha K Shetty, Kenji Shibuya, Aminu Shittu, Kerem Shuval, Emmanuel Edwar Siddig, Diego Augusto Santos Silva, Jasvinder A Singh, Amanda E Smith, Ranjan Solanki, Sameh S M Soliman, Yi Song, Soroush Soraneh, Kurt Straif, Lukasz Szarpak, Seyyed Mohammad Tabatabaei, Celine Tabche, Manoj Tanwar, Nathan Y Tat, Mohamad-Hani Temsah, Aravind Thavamani, Thang Huu Tran, Domenico Trico, Thien Tan Tri Tai Truyen, Stefanos Tyrovolas, Arit Udoh, Sana Ullah, Seyed Mohammad Vahabi, Sanaz Vahdati, Asokan Govindaraj Vaithinathan, Azin Vakilpour, Jef Van den Eynde, Manish Vinayak, Kosala Gayan Weerakoon, Nuwan Darshana Wickramasinghe, Asrat Arja Wolde, Tewodros Eshete Wonde, Suowen Xu, Lin Yang, Yuichiro Yano, Arzu Yiğit, Dong Keon Yon, Chuanhua Yu, Chun-Wei Yuan, Michael Zastrozhin, Mohammed G M Zeariya, Claire Chenwen Zhong, Bin Zhu, Abzal Zhumagaliuly, Magdalena Zielińska, Sa'ed H Zyoud, Jessica A Kerr†, Stein Emil Vollset†, Emmanuela Gakidou†.

*Co-first Authors

†Co-senior Authors

## Affiliations

Yong Loo Lin School of Medicine (M Ng PhD), Department of Medicine (B Chong MBBS), National University of Singapore, Singapore, Singapore; Institute for Health Metrics and Evaluation (M Ng PhD, X Dai PhD, R M Cogen BA, N Ahmad BS, A Y Aravkin PhD, D Bryazka BA, Prof S D Dharmaratne MD, Prof V L Feigin PhD, Prof S I Hay FMedSci, Prof S S Lim PhD, P A Lindstedt MPH, J Lo BA, T Mestrovic PhD, Prof A H Mokdad PhD, E C Mullany BA, Prof C J L Murray DPhil, E M O'Connell BA, S M Ostroff PhD, T Pham BS, C Raggi MS, A E Smith MPA, A A Wolde MPH, C Yuan PhD, Prof S E Vollset DrPH, Prof E Gakidou PhD), Department of Health Metrics Sciences, School of Medicine (X Dai PhD, A Y Aravkin PhD, Prof S D Dharmaratne MD, Prof S I Hay FMedSci, Prof S S Lim PhD, Prof A H Mokdad PhD, Prof C J L Murray DPhil, Prof S E Vollset DrPH, Prof E Gakidou PhD), Department of Applied Mathematics (A Y Aravkin PhD), School of Medicine (E J Boyko MD), Henry M Jackson School of International Studies (S M Ostroff PhD), University of Washington, Seattle, WA, USA; Department of Surgery (M Abdelmasseh MD), Marshall University, Huntington, WV, USA; Minimally Invasive Surgery Research Center (A Abdollahi MD), Health Management and Economics Research Center (J Arabloo PhD), School of Medicine (M Bastan MD), Gastrointestinal and Liver Diseases Research Center (Prof M Moradi-Lakeh MD), Preventive Medicine and Public Health Research Center (Prof M Moradi-Lakeh MD), Iran University of Medical Sciences, Tehran, Iran; Department of Physiotherapy (A Abdullahi PhD), Department of Community Medicine (Prof M A Gadanya MD), Bayero University Kano, Kano, Nigeria; Department of Physiotherapy (A Abdullahi PhD), Federal University Wukari, Wukari, Nigeria; Department of Family and Community Health (R G

Aboagye MPH), Department of Epidemiology and Biostatistics (R K Dowou MPhil), University of Health and Allied Sciences, Ho, Ghana; Academic Health System (H J Abukhadijah MPH), Hamad Medical Corporation, Doha, Qatar; Department of Pharmacology and Therapeutics (T E Adeyeoluwa PhD), University of Medical Sciences, Ondo, Ondo, Nigeria; Department of Veterinary Medicine (T E Adeyeoluwa PhD), Department of Medicine (Prof M O Owolabi DrM), University of Ibadan, Ibadan, Nigeria; Technical Services Directorate (A A Afolabi MPH), MSI Nigeria Reproductive Choices, Abuja, Nigeria; School of Medicine and Psychology (D Ahmad PhD), Australian National University, Canberra, ACT, Australia; Public Health Foundation of India, Gandhinagar, India (D Ahmad PhD); Institute of Endemic Diseases (A Ahmed MSc), Unit of Basic Medical Sciences (E E Siddig MD), University of Khartoum, Khartoum, Sudan; Swiss Tropical and Public Health Institute (A Ahmed MSc), University of Basel, Basel, Switzerland; Brody School of Medicine (S Ahmed PhD), East Carolina University, Greenville, NC, USA; Department of Cardiology (M Akkaif PhD), Fudan University, Shanghai, China; Chicago College of Osteopathic Medicine (A E Akrami BS), Midwestern University, Downers Grove, IL, USA; Feinberg School of Medicine (A E Akrami BS), Department of Medicine (N S Shah MD), Northwestern University, Chicago, IL, USA; Division of Public Health Sciences (S Al Hasan PhD), Department of Research and Development (Z Al-Aly MD), Department of Surgery (S Azadnajafabad MD), Mallinckrodt Institute of Radiology (M Dolatshahi MD), Washington University in St. Louis, St. Louis, MO, USA; Department of Internal Medicine (O Al Ta'ani MD), Allegheny Health Network, Pittsburgh, PA, USA; Department of Biomedical Informatics, Biostatistics, and Epidemiology (F Alahdab MD), University of Missouri, Columbia, MO, USA; McWilliams School of Biomedical Informatics (F Alahdab MD), McGovern Medical School (A Bleyer MD), University of Texas, Houston, TX, USA; Clinical Epidemiology Center (Z Al-Aly MD), US Department of Veterans Affairs (VA), St. Louis, MO, USA; Division of Gastroenterology and Hepatology (W A Aldhaleei MD), Mayo Clinic, Jacksonville, FL, USA; Department of Bacteriology, Immunology, and Mycology (Prof A M Algammal PhD), Suez Canal University, Ismailia, Egypt; Department of Geography (W Ali PhD), Sultan Qaboos University, Muscat, Oman; Department of Nuclear Medicine (Prof A Al-Ibraheem MD), King Hussein Cancer Center, Amman, Jordan; Department of Diagnostic Radiology and Nuclear Medicine (Prof A Al-Ibraheem MD), School of Pharmacy (Prof Y Bustanji PhD), The University of Jordan, Amman, Jordan; Department of Medicine (S A Alqahatni MD), King Faisal Specialist Hospital & Research Center, Riyadh, Saudi Arabia; Department of Medicine (S A Alqahatni MD), Department of Biostatistics (A Columbus MS), Johns Hopkins University, Baltimore, MD, USA; Institute of Public Health (R Al-Rifai PhD), United Arab Emirates University, Al Ain, United Arab Emirates; Department of Family and Community Medicine (N Z Alshahrani MD), University of Jeddah, Jeddah, Saudi Arabia; Department of Rehabilitation Sciences (M Al-Wardat PhD), Jordan University of Science and Technology, Irbid, Jordan; Department of Pediatrics (Prof H Aly MD, A E'mar MD), Cleveland Clinic, Cleveland, OH, USA; Department of Biomedical Engineering (W A Al-Zyoud PhD), German Jordanian University, Amman, Jordan; Quran and Hadith Research Center (S Amiri PhD), Baqiyatallah University of Medical Sciences, Tehran, Iran; Department of Pharmacology (A Anil MD, A Saravanan MD, M Shamim MBBS), All India Institute of Medical Sciences, Jodhpur, India; All India Institute of Medical Sciences, Bhubaneswar, India (A Anil MD); Health Policy Research Center (A Ardekani MD), Non-communicable Disease Research Center (S G Sepanlou MD), Shiraz University of Medical Sciences, Shiraz, Iran; College of Art and Science (D Areda PhD), Ottawa University, Surprise, AZ, USA; School of Life Sciences (D Areda PhD), Arizona State University, Tempe, AZ, USA; Department of Public Health (M Y Ashemo MPH), Jimma University, Jimma, Ethiopia; Department of Public Health (M Y Ashemo MPH), Wachemo University, Hossana, Ethiopia; Department of Forensic Medicine (A Atreya MD), Lumbini

Medical College, Palpa, Nepal; Leeds Institute of Rheumatic and Musculoskeletal Medicine (S Azadnajafabad MD), University of Leeds, Leeds, UK; Institute of Biotechnology and Genetic Engineering (S Aziz MS), The University of Agriculture, Peshawar, Pakistan; Centre for Adolescent Health (P S Azzopardi PhD, K I Cini MClinEpi, K L Francis MBiostat, J A Kerr PhD, Prof S M M Sawyer MD), Murdoch Childrens Research Institute, Parkville, VIC, Australia; Department of Paediatrics (P S Azzopardi PhD), University of Melbourne, Melbourne, VIC, Australia; Global Adolescent Health Group (P S Azzopardi PhD, K I Cini MClinEpi), Burnet Institute, Melbourne, VIC, Australia; Department of Population Medicine (Prof G Babu PhD), Qatar University, Doha, Qatar; International Medical School (A A Baig PhD), Management and Science University, Alam, Malaysia; Department of Neurosurgery (A T Bako PhD), Houston Methodist Hospital, Houston, TX, USA (M Elhadi MD); Department of Medicine (K Bansal MD), University of Massachusetts Medical School, Worcester, MA, USA; Department of Medicine (K Bansal MD), Saint Vincent Hospital, Worcester, MA, USA; Heidelberg Institute of Global Health (HIGH) (Prof T W Bärnighausen MD, Prof S Mohammed PhD), Heidelberg University, Heidelberg, Germany; T.H. Chan School of Public Health (Prof T W Bärnighausen MD, P M S Pradhan MD), Division of Cardiovascular Medicine (G Chi MD), Department of Orthopedic Surgery (K Momenzadeh MD), Department of Health Policy and Oral Epidemiology (Z S Natto DrPH), School of Public Health (Prof N P Pronk PhD), Beth Israel Deaconess Medical Center (S Sharfaei MD), Division of General Internal Medicine (Prof A Sheikh MD), Harvard University, Boston, MA, USA; Non-communicable Diseases Research Center (M Bastan MD, S Momtazmanesh MD), Department of Pediatric Neurology (M Bemanalizadeh MD), Iranian Research Center for HIV/AIDS (IRCHA) (O Dadras PhD), School of Medicine (H Farrokhpour MD, M Gouravani MD, S Khanmohammadi MD, S Momtazmanesh MD), Cardiovascular Diseases Research Institute (Y Karimi MD), Cardiac Primary Prevention Research Center (S Kazemian MD), Department of Cardiac Electrophysiology (S Kazemian MD), Sina Trauma and Surgery Research Center (M Khormali MD), Department of Cardiology (E Mahmoudi MD), Department of Pediatric Cardiology (Prof E Malakan Rad MD), Digestive Diseases Research Institute (S G Sepanlou MD), Department of Neurology (M Shafie MD), Research Center for Rational Use of Drugs (A Sharifan PharmD), Faculty of Medicine (S Vahabi MD), Tehran University of Medical Sciences, Tehran, Iran; Department of Pediatrics (M Bemanalizadeh MD), Heart Failure Research Center (S Najdaghi MD, D Narimani Davani MD), Neuroscience Research Center (S Najdaghi MD), Isfahan University of Medical Sciences, Isfahan, Iran; School of Medicine (A Beran MD), Indiana University, Indianapolis, IN, USA; Metabolomics Laboratory (H B Beyene PhD), Hypertension and Kidney Disease Laboratory (Prof M P Schlaich MD), Baker Heart and Diabetes Institute, Melbourne, VIC, Australia; Department of Microbiology (H B Beyene PhD), Addis Ababa University, Addis Ababa, Ethiopia; Global Health Neurology Lab (S Bhaskar MD), NSW Brain Clot Bank, Sydney, NSW, Australia; Division of Cerebrovascular Medicine and Neurology (S Bhaskar MD), National Cerebral and Cardiovascular Center, Suita, Japan; Department of Radiology (C Bilgin MD), Neurovascular Research Laboratory (C Bilgin MD), Mayo Clinic College of Medicine, Rochester, MN, USA; Department of Radiation Medicine (A Bleyer MD), Oregon Health and Science University, Portland, OR, USA; Internal Medicine Department (H Borhany MD), School of Medicine (D J Ghadimi MD), Research Center for Social Determinants of Health (A Halimi MSc), Student Research Committee (M Rahmanian MD), Department of Epidemiology (Prof S Sabour PhD), Shahid Beheshti University of Medical Sciences, Tehran, Iran; General Medicine Service (E J Boyko MD), Department of Veterans Affairs, Seattle, WA, USA; Department of Epidemiology (D Braithwaite PhD), College of Medicine (M J Diaz BS), University of Florida, Gainesville, FL, USA; Cancer Population Sciences Program (D Braithwaite PhD), University of Florida Health Cancer Center, Gainesville, FL, USA; Department of Medical and Surgical Sciences (Prof R Bugiardini MD), University of Bologna, Bologna,

Italy; Department of Basic Biomedical Sciences (Prof Y Bustanji PhD), Clinical Sciences Department (N R Dash MD), College of Medicine (Prof M A Saleh PhD), Department of Medicinal Chemistry (S S M Soliman PhD), University of Sharjah, Sharjah, United Arab Emirates; School of Public Health Sciences (Z A Butt PhD), University of Waterloo, Waterloo, ON, Canada; Al Shifa School of Public Health (Z A Butt PhD), Al Shifa Trust Eye Hospital, Rawalpindi, Pakistan; Faculty of Health Sciences (M Çakmak Barsbay PhD), Ankara University, Ankara, Türkiye; Center for Nutrition and Health Research (I Campos-Nonato PhD), National Institute of Public Health, Cuernavaca, Mexico; Department of Nutrition (Prof F Cembranel DSc), Department of Physical Education (Prof D A S Silva PhD), Federal University of Santa Catarina, Florianópolis, Brazil; Mary MacKillop Institute for Health Research (Prof E Cerin PhD), Australian Catholic University, Melbourne, VIC, Australia; School of Public Health (Prof E Cerin PhD), University of Hong Kong, Hong Kong, China; Carolina Health Informatics Program (P Chacón-Uscamaita DDS), University of North Carolina Chapel Hill, Chapel Hill, NC, USA; Department of Anesthesiology and Perioperative Medicine (E K Chandrasekar MD), School of Medicine (Prof S Xu PhD), University of Rochester, Rochester, NY, USA; Temerty Faculty of Medicine (V Chattu MD), University of Toronto, Toronto, ON, Canada; Department of Community Medicine (V Chattu MD), Datta Meghe Institute of Medical Sciences, Sawangi, India; Fuwai Hospital (A Chen PhD), Chinese Academy of Medical Sciences & Peking Union Medical College, Beijing, China; Department of Computer Science (A Chen PhD), University of Texas at Austin, Austin, TX, USA; Department of Stomatology (G Chen DMD), Huazhong University of Science and Technology, Wuhan, China; Hubei Province Key Laboratory of Oral and Maxillofacial Development and Regeneration, Wuhan, China (G Chen DMD); Division of Infectious Diseases (P R Ching MD), Virginia Commonwealth University, Richmond, VA, USA; Program in Medical and Population Genetics (S J Cho PhD), Broad Institute of MIT and Harvard, Cambridge, MA, USA (M Kim MD); Cardiovascular Research Center (S J Cho PhD, A Schuermans BSc), Department of Radiology (X Liu PhD), Division of Cardiology (D H Nguyen BS), Massachusetts General Hospital, Boston, MA, USA (M Kim MD); Cancer Big Data Center (D Choi PhD), National Cancer Center, Goyang, South Korea; Department of Health Informatics (S Chung PhD), University College London, London, UK; Health Data Research UK, London, UK (S Chung PhD); Department of Genetics (Z Cindi PhD), Department of Biostatistics, Epidemiology, and Informatics (J Puvvula PhD), Cardiovascular Medicine (A Vakilpour MD), University of Pennsylvania, Philadelphia, PA, USA; Department of Chemical Sciences (R A S Couto MD), Institute for Research and Innovation in Health (i3S) (Prof N Cruz-Martins PhD), University of Porto, Porto, Portugal; Department of Family Medicine and Public Health (Prof M H Criqui MD), University of California San Diego, La Jolla, CA, USA; Department of Diagnostic and Therapeutic Technologies (Prof N Cruz-Martins PhD), Cooperativa de Ensino Superior Politécnico e Universitário (Polytechnic and University Higher Education Cooperative), Vila Nova de Famalicão, Portugal; Department of Health Systems Management (Prof O B Da'ar PhD), King Saud bin Abdulaziz University for Health Sciences, Riyadh, Saudi Arabia; Department of Global Public Health and Primary Care (O Dadras PhD), Department of Psychosocial Science (Prof D Sagoe PhD), University of Bergen, Bergen, Norway; School of Population Health (Z Dai PhD), International Centre for Future Health Systems (J Lin PhD), Centre for Social Research in Health (S R Okeke PhD), University of New South Wales, Sydney, NSW, Australia; School of Pharmacy and Charles Perkins Centre (Z Dai PhD), Faculty of Medicine and Health (W He PhD), Sydney Medical School (S Islam PhD), University of Sydney, Sydney, NSW, Australia (S R Okeke PhD); Department of Public Health (S D Darcho MPH), Haramaya University, Harar, Ethiopia; Department of Research (H D Desai MD), Gujarat Adani Institute of Medical Sciences, Bhuj, India; Department of Community Medicine (Prof S D Dharmaratne MD), University of Peradeniya, Peradeniya, Sri Lanka; Faculty of Science (Prof D Diaz PhD), National Autonomous University of Mexico,

Mexico City, Mexico; Department of Medicine (T C Do MD), Pham Ngoc Thach University of Medicine, Ho Chi Minh City, Vietnam; Department of Medical, Surgical, and Health Sciences (Prof M D'Oria MD), University of Trieste, Trieste, Italy; Cardio-Thoraco-Vascular Department (Prof M D'Oria MD), Azienda Sanitaria Universitaria Giuliano Isontina, Trieste, Italy; Independent Consultant, South Plainfield, NJ, USA (O P Doshi MSc); Department of Cardiology (R P Doshi MD), Hackettstown Medical Center, Hackettstown, NJ, USA; Newton Medical Center, Sparta, NJ, USA (R P Doshi MD); Office of Institutional Analysis (J Dube MA), University of Windsor, Windsor, ON, Canada; Allied Health and Human Performance (D Dumuid PhD), University of South Australia, Adelaide, SA, Australia; Department of Conservative Dentistry with Endodontics (A M Dziedzic DSc), Medical University of Silesia, Katowice, Poland; Almoosa College of Health Sciences, Al Ahsa, Saudi Arabia (R A El Arab PhD); Department of Public Health and Community Medicine (Prof I F El Bayoumy DrPH), Tanta University, Tanta city, Egypt; School of Public Health (Prof I F El Bayoumy DrPH), Texila American University, Guyana, Guyana; Faculty of Medicine (M Elhadi MD), University of Tripoli, Tripoli, Libya; Department of Pediatrics (C Eltaha MD), Texas A&M University, Dallas, TX, USA; Department of Biomedical and Biotechnological Sciences (L Falzone PhD), University of Catania, Catania, Italy; Epidemiology and Biostatistics Unit (L Falzone PhD), IRCCS Pascale, Naples, Italy; Endocrinology and Metabolism Research Institute (H Farrokhpour MD), Department of Epidemiology (S Khanmohammadi MD, S Rashedi MD), Non-Communicable Diseases Research Center (NCDRC), Tehran, Iran (F Montazeri MD); Department of Biology and Medicine (P Fazeli MSc), Department of Internal Medicine (M F H Mohamed MSc), Brown University, Providence, RI, USA; National Institute for Stroke and Applied Neurosciences (Prof V L Feigin PhD), Auckland University of Technology, Auckland, New Zealand; Research Center of Neurology, Moscow, Russia (Prof V L Feigin PhD); Department of Public Health and Infectious Diseases (G Fekadu PhD), City University of Hong Kong, Hong Kong, China; Department of Pharmacy (G Fekadu PhD), Wollega University, Nekemte, Ethiopia; Department of Social Sciences (Prof N Ferreira PhD), University of Nicosia, Nicosia, Cyprus; Institute of Public Health (F Fischer PhD), Department of Surgery (N Haep MD), Charité Universitätsmedizin Berlin (Charité Medical University Berlin), Berlin, Germany; Department of Community Medicine (Prof M A Gadanya MD), Aminu Kano Teaching Hospital, Kano, Nigeria; Department of Midwifery (M W Gebregergis MSc), Department of Medical Laboratory Sciences (H N Meles MSc), Adigrat University, Adigrat, Ethiopia; Department of Electrical and Computer Engineering (E Gholami PhD), University of California Davis, Davis, CA, USA; Department of Health Systems and Policy Research (Prof M Golechha PhD), Indian Institute of Public Health, Gandhinagar, India; Department of Life Sciences, Health and Healthcare Professions (Prof D Golinelli MD), Link Campus University, Rome, Italy; Health Services Research, Evaluation and Policy Unit (Prof D Golinelli MD), AUSL della Romagna, Ravenna, Italy; Department of Urban Public Health (Prof P N Gona PhD), University of Massachusetts Boston, Boston, MA, USA; Department of Dermatology (A Grada MD), Harrington Heart and Vascular Institute (A Guha MD), Department of Pediatrics (A Thavamani MD), Division of Pediatric Gastroenterology (A Thavamani MD), Case Western Reserve University, Cleveland, OH, USA; Department of Endocrinology (A Grover MD), Center for Translation Research and Implementation Science (G A Mensah MD), National Institutes of Health, Bethesda, MD, USA; Division of Cardiovascular Medicine (A Guha MD), Ohio State University, Columbus, OH, USA; Department of Cardiology (R Gupta MBBS), Lehigh Valley Health Network, Allentown, PA, USA; Department of Medicine (P Habibzadeh MD), University of Pittsburgh Medical Center, Pittsburgh, PA, USA; Clinician Scientist Program (N Haep MD), Berlin Institute of Health, Berlin, Germany; Department of Health Research Methods, Evidence, and Impact (M Hasan MPH, O O Olasupo PhD), Department of Medicine (O P Kurmi PhD), Department of

Psychiatry and Behavioural Neurosciences (A T Olagunju MD), McMaster University, Hamilton, ON, Canada; Department of Biochemistry and Molecular Biology (M Hasan MPH), Tejgaon College, Dhaka, Bangladesh; Department of Pharmacy (Prof M S Hasnain PhD), Marwadi University, Rajkot, India; Faculty of Kinesiology (Prof J J Hebert PhD), University of New Brunswick, Fredericton, NB, Canada; School of Allied Health (Prof J J Hebert PhD), Murdoch University, Murdoch, WA, Australia; Department of Medicine (M Hemmati MD), MedStar Health, Washington, DC, USA; Department of Medicine (M Hemmati MD, C J Sabet MA), Georgetown University, Washington, DC, USA; Graduate School of Medicine (Y Hiraike PhD), University of Tokyo, Tokyo, Japan; School of Dentistry (N Hoan DDS), Hanoi Medical University, Hanoi, Vietnam; Department of Legal Medicine and Bioethics (Prof S Hostiuc PhD), Carol Davila University of Medicine and Pharmacy, Bucharest, Romania; Department of Clinical Legal Medicine (Prof S Hostiuc PhD), National Institute of Legal Medicine Mina Minovici, Bucharest, Romania; Department of Psychology (C Hu PhD), Tsinghua University, Beijing, China; Faculty of Medicine (J Huang MD), Jockey Club School of Public Health and Primary Care (C Zhong PhD), The Chinese University of Hong Kong, Hong Kong, China; International Master Program for Translational Science (H Huynh BS), Taipei Medical University, Taipei, Taiwan; School of Pharmacy (M Islam PhD), BRAC University, Dhaka, Bangladesh; Institute for Physical Activity and Nutrition (S Islam PhD), Deakin University, Burwood, VIC, Australia; Department of Physical and Medicine (L Jacob MD), Université Paris Cité, Paris, France; Research and Development Unit (L Jacob MD), Biomedical Research Networking Center for Mental Health Network (CiberSAM), Barcelona, Spain; Department of Gastroenterology and Hepatology (A Joseph MD), Department of Biomedical Data Science (S Park MD), Department of Radiology (S Ramasamy MD), Stanford University, Stanford, CA, USA; Academic Department of Surgery (S K Kamarajah MD), University of Birmingham, Birmingham, UK; Faculty of Dentistry (K K Kanmodi MPH), University of Puthisastra, Phnom Penh, Cambodia; Office of the Executive Director (K K Kanmodi MPH), Cephas Health Research Initiative Inc, Ibadan, Nigeria; The Hansjörg Wyss Department of Plastic and Reconstructive Surgery (R S Kantar MD), NYU Langone Health, New York, NY, USA; Cleft Lip and Palate Surgery Division (R S Kantar MD), Global Smile Foundation, Norwood, MA, USA; Department of Rehabilitation Sciences (M Khan MPH), School of Nursing (S Tyrovolas PhD), Hong Kong Polytechnic University, Hong Kong, China; Department of Internal Medicine (M S Khan MD), John H. Stroger, Jr. Hospital of Cook County, Chicago, IL, USA; Department of Internal Medicine (M S Khan MD), Dow University of Health Sciences, Karachi, Pakistan; Department of Medical Biochemistry (P Khanal PhD), Pokhara University, Kathmandu, Nepal; College of Health, Wellbeing and Life Sciences (Prof K Khatab PhD), Sheffield Hallam University, Sheffield, UK; College of Arts and Sciences (Prof K Khatab PhD), Ohio University, Zanesville, OH, USA; Department of Basic Medical Sciences (Prof M M Khatatbeh PhD), Yarmouk University, Irbid, Jordan; Department of Public Health (J Khubchandani PhD), New Mexico State University, Las Cruces, NM, USA; Public Health Service (S Kiconco PhD), Department of Health and Human Services, Hobart, TAS, Australia; Millennium Prevention, Inc., Westwood, MA, USA (R W Kimokoti MD); School of Health Sciences (Prof A Kisa PhD), Kristiania University College, Oslo, Norway; Department of International Health and Sustainable Development (Prof A Kisa PhD), Tulane University, New Orleans, LA, USA; Atchabarov Scientific-Research Institute of Fundamental and Applied Medicine (M Kulimbet MSc, A Zhumagaliuly MD), Kazakh National Medical University, Almaty, Kazakhstan; Center of Medicine and Public Health (M Kulimbet MSc), Asfendiyarov Kazakh National Medical University, Almaty, Kazakhstan; Geospatial Information Science and Engineering Hub (V Kumar PhD), Indian Institute of Technology, Mumbai, India; Centre for Studies in Economics and Planning (V Kumar PhD), Central University of Gujarat, Gandhinagar, India; School of Medicine and Dentistry (S Kundu MPH),

Griffith University, Gold Coast, QLD, Australia; Department of Nutrition and Food Science (S Kundu MPH), Patuakhali Science and Technology University, Patuakhali, Bangladesh; Faculty of Health and Life Sciences (O P Kurmi PhD), Coventry University, Coventry, UK; Department of Occupational and Environmental Health (H Lai PhD), Yangzhou University, Yangzhou, China; Department of Respiratory and Critical Care Medicine (H Lai PhD), Northern Jiangsu People's Hospital, Yangzhou, China; Faculty of Medicine (N Le MD), Department of General Medicine (V T Nguyen MD), Department of Internal Medicine (T H Tran MD), University of Medicine and Pharmacy at Ho Chi Minh City, Ho Chi Minh City, Vietnam; Department of Cardiovascular Research (N Le MD), Methodist Hospital, Merrillville, IN, USA; Department of Medical Science (M Lee PhD), Ajou University School of Medicine, Suwon, South Korea; Department of Precision Medicine (Prof S Lee MD), Sungkyunkwan University, Suwon-si, South Korea; Department of Family Medicine (W Lee PhD), University of Texas Medical Branch, Galveston, TX, USA; Center for Dentistry and Oral Hygiene (A Li PhD), University of Groningen, Groningen, Netherlands; Stomatological Hospital (A Li PhD), Southern Medical University, Guangzhou, China; Department of Psychiatry (W Li PhD, T Rhee PhD), Department of Radiology and Biomedical Imaging (X Liu PhD), Yale University, New Haven, CT, USA; One Health Research Group (J López-Gil PhD), Universidad de Las Américas (University of the Americas), Quito, Ecuador; School of Medicine (Prof G Lucchetti PhD), Federal University of Juiz de Fora, Juiz de Fora, Brazil; Center for Evidence-Based and Translational Medicine (L Luo MPH), Department of Epidemiology and Biostatistics (Prof C Yu PhD), Wuhan University, Wuhan, China; Department of Population Health Sciences (J B Lusk MD), Duke University, Durham, NC, USA; Smidt Heart Institute (Y Manla MD), Cedars-Sinai Medical Center, Los Angeles, CA, USA; Department of Non-communicable Diseases and Mental Health (R Martinez-Piedra BSc), Pan American Health Organization, Washington, DC, USA; Department of Anatomy and Developmental Biology (Y Mathangasinghe PhD), Monash University, Clayton, VIC, Australia; Department of Anatomy, Genetics and Biomedical Informatics (Y Mathangasinghe PhD), University of Colombo, Colombo, Sri Lanka; Department of Maternal-Child Nursing and Public Health (Prof F P Matozinhos PhD), Federal University of Minas Gerais, Belo Horizonte, Brazil; Australian Centre for Health Services Innovation (Prof S M McPhail PhD), Queensland University of Technology, Kelvin Grove, QLD, Australia; Digital Health and Informatics Directorate (Prof S M McPhail PhD), Queensland Health, Brisbane, QLD, Australia; Department of Medicine (G A Mensah MD), University of Cape Town, Cape Town, South Africa; Department of Physiology (Prof S A Meo PhD), Pediatric Intensive Care Unit (Prof M Temsah MD), King Saud University, Riyadh, Saudi Arabia; University Centre Varazdin (T Mestrovic PhD), University North, Varazdin, Croatia; National Cancer Registry (I Michalek PhD), Department of Pathology (I Michalek PhD), Maria Sklodowska-Curie National Research Institute of Oncology, Warsaw, Poland; Department of Public Health Dentistry (Prof G Mini PhD), Saveetha Institute of Medical and Technical Sciences (SIMATS), Chennai, India; Global Institute of Public Health (Prof G Mini PhD), Ananthapuri Hospitals and Research Institute, Trivandrum, India; Department of Radiology (M Mirza-Aghazadeh-Attari MD), Tabriz University of Medical Sciences, Tabriz, Iran; Social Determinants of Health Center (M Mirza-Aghazadeh-Attari MD), Student Research Committee (S Soraneh MD), Urmia University of Medical Sciences, Urmia, Iran; Foundation for Liver Research (G Mocciaro PhD), Foundation for Liver Research, London, UK; College of Applied and Natural Science (J Mohamed MSc), University of Hargeisa, Hargeisa, Somalia; Molecular Biology Unit (N S Mohamed MSc), Bio-Statistical and Molecular Biology Department (N S Mohamed MSc), Sirius Training and Research Centre, Khartoum, Sudan; College of Medicine (Prof A M Mohammad MD), University of Duhok, Duhok, Iraq; Health Systems and Policy Research Unit (Prof S Mohammed PhD), Department of Community Medicine (A A Olorukooba MD), Ahmadu Bello University, Zaria,

Nigeria; Department of Ophthalmology & Vision Science (F Montazeri MD), University of California Davis, Sacramento, CA, USA; Division of Plastic and Reconstructive Surgery (S D Morrison MD), University of Washington Medical Center, Seattle, WA, USA; Department of Community Medicine (R Motappa MD), Manipal College of Dental Sciences Mangalore (Prof P K Shetty MDS), Manipal Academy of Higher Education, Mangalore, India; Department of Computer Science (P Naghavi MS), University of Illinois Urbana-Champaign, Urbana, IL, USA; National Dental Research Institute Singapore (G G Nascimento PhD), Duke-NUS Medical School, Singapore, Singapore; Department of Dental Public Health (Z S Natto DrPH), King Abdulaziz University, Jeddah, Saudi Arabia; Department of Medical Engineering (D H Nguyen BS), University of South Florida, Tampa, FL, USA; Gastrointestinal and Liver Diseases Research Center (H T H Nguyen MD), Institute for Research and Training in Medicine, Biology and Pharmacy (H T H Nguyen MD), Duy Tan University, Da Nang, Vietnam; Department of Surgery (P T Nguyen MD), Danang Family Hospital, Danang, Vietnam; Institute for Mental Health Policy Research (Y T Nigatu PhD), Centre for Addiction and Mental Health, Toronto, ON, Canada; Department of Nephrology and Hypertension (N Nikravangolsefid MD), Department of Radiology (F Nugen PhD), Department of Cardiovascular Medicine (H Pham MD), Department of Informatics and Radiology (S Vahdati MD), Mayo Clinic, Rochester, MN, USA; Maternal and Child Health Divisions (S Noor MS), International Centre for Diarrhoeal Disease Research, Bangladesh, Dhaka, Bangladesh; Department of Statistics (S Noor MS), Shahjalal University of Science and Technology, Sylhet, Bangladesh; School of Information (F Nugen PhD), University of California Berkeley, Berkeley, CA, USA; Department of Physiology (O J Nzoputam PhD), University of Benin, Edo, Nigeria; Department of Physiology (O J Nzoputam PhD), Benson Idahosa University, Benin City, Nigeria; Department of Applied Economics and Quantitative Analysis (Prof B Oancea PhD), University of Bucharest, Bucharest, Romania; Department of Psychiatry (A T Olagunju MD), University of Lagos, Lagos, Nigeria; Department of Epidemiology and Population Health (A Oulhaj PhD), Khalifa University, Abu Dhabi, United Arab Emirates; Department of Medicine (Prof M O Owolabi DrM), University College Hospital, Ibadan, Ibadan, Nigeria; Department of Respiratory Medicine (Prof M P P A DNB), Jagadguru Sri Shivarathreeswara University, Mysore, India; Department of Epidemiology and Community Health (R R Parikh MD), University of Minnesota, Minneapolis, MN, USA; Department of Health Policy and Management (S Park PhD), Korea University, Seoul, South Korea; School of Nursing (A Pashaei MSc), University of British Columbia, Vancouver, BC, Canada; School of Population Health (Prof G Pereira PhD), Curtin University, Bentley, WA, Australia; Centre for Fertility and Health (Prof G Pereira PhD), Norwegian Institute of Public Health, Oslo, Norway; Department of Internal Medicine (H Pham MD), University of Arizona, Tucson, AZ, USA; School of Pharmacy (A K Philip PhD), University of Nizwa, Nizwa, Oman; Department of Humanities and Social Sciences (Prof J Pradhan PhD), National Institute of Technology Rourkela, Rourkela, India; Department of Community Medicine (P M S Pradhan MD), Tribhuvan University, Kathmandu, Nepal; Office of the President (Prof N P Pronk PhD), HealthPartners Institute, Bloomington, MN, USA; Department of Radiology (S Rafiei Alavi MD), University of California Irvine, Irvine, CA, USA; Institute of Health and Wellbeing (Prof M Rahman PhD), Federation University Australia, Berwick, VIC, Australia; School of Nursing and Midwifery (Prof M Rahman PhD), La Trobe University, Melbourne, VIC, Australia; Cellular and Molecular Research Center (B Rahmani MSc), Qazvin University of Medical Sciences, Qazvin, Iran; Department of Research (C L Ranabhat PhD), Eastern Scientific LLC, Richmond, KY, USA; Department of Health Promotion and Administration (C L Ranabhat PhD), Eastern Kentucky University, Richmond, KY, USA; Department of Oral Pathology, Microbiology and Forensic Odontology (S Rao MDS), Sharavathi Dental College and Hospital, Shimogga, India; Brigham and Women's Hospital (S Rashedi MD), Harvard Medical School, Boston, MA, USA; Department of Medicine

(A M Rashid MD), Jinnah Sindh Medical University, Karachi, Pakistan; Baylor University, Dallas, TX, USA (A M Rashid MD); Department of Biological Sciences (Prof E M M Redwan PhD), King Abdulaziz University, Jeddah, Egypt; Department of Protein Research (Prof E M M Redwan PhD), Research and Academic Institution, Alexandria, Egypt; Department of Public Health Sciences (T Rhee PhD), University of Connecticut, Farmington, CT, USA; Department of Geography and Demography (M Rodrigues PhD), University of Coimbra, Coimbra, Portugal; Department of Pharmacology and Toxicology (Prof J A B Rodriguez PhD), University of Antioquia, Medellin, Colombia; Warwick Medical School (Prof J A B Rodriguez PhD), University of Warwick, Coventry, UK; Clinical and Biomedical Research Center (Prof U Saeed PhD), Foundation University Islamabad, Islamabad, Pakistan; International Center of Medical Sciences Research (ICMSR), Islamabad, Pakistan (Prof U Saeed PhD); Faculty of Pharmacy (Prof M A Saleh PhD), Mansoura University, Mansoura, Egypt; Department of Anatomy (Prof V P Samuel PhD), Ras Al Khaimah Medical and Health Sciences University, Ras Al Khaimah, United Arab Emirates; Department of Entomology (A M Samy PhD), Medical Ain Shams Research Institute (MASRI) (A M Samy PhD), Ain Shams University, Cairo, Egypt; Indira Gandhi Medical College and Research Institute, Puducherry, India (A Saravanan MD); Department of Public Health Sciences (M Sawhney PhD), University of North Carolina at Charlotte, Charlotte, NC, USA; Department of Paediatrics (Prof S M M Sawyer MD), University of Melbourne, Parkville, VIC, Australia; Department of Neurology (Prof N Scarmeas PhD), National and Kapodistrian University of Athens, Athens, Greece; Department of Neurology (Prof N Scarmeas PhD), Columbia University, New York, NY, USA; Dobney Hypertension Centre (Prof M P Schlaich MD), The University of Western Australia, Perth, WA, Australia; Department of Cardiovascular Sciences (A Schuermans BSc, J Van den Eynde BSc), Katholieke Universiteit Leuven (Catholic University Leuven), Leuven, Belgium; National Heart, Lung, and Blood Institute (A Seylani BS), National Institutes of Health, Rockville, MD, USA; Department of Pathobiology (M Shamshirgaran PhD), Shahid Bahonar University of Kerman, Kerman, Iran; Department of Safety Services (S Sharfaei MD), Baim Institute for Clinical Research, Boston, MA, USA; Department for Evidence-based Medicine and Evaluation (A Sharifan PharmD), University for Continuing Education Krems, Krems, Austria; Department of Hemato-oncology (A Sharma MD), Fortis Hospital, Noida, India; Department of Social and Behavioral Health (Prof M Sharma PhD), University of Nevada Las Vegas, Las Vegas, NV, USA; Centre for Medical Informatics (Prof A Sheikh MD), University of Edinburgh, Edinburgh, UK; Department of Pharmacology (R R Shenoy PhD), Manipal Academy of Higher Education, Manipal, India; Tokyo Foundation for Policy Research, Tokyo, Japan (Prof K Shibuya MD); Department of Veterinary Public Health and Preventive Medicine (A Shittu MSc), Usmanu Danfodiyo University, Sokoto, Sokoto, Nigeria; The Cooper Institute, Dallas, TX, USA (K Shuval PhD); Department of Medical Microbiology and Infectious Diseases (E E Siddig MD), Erasmus University, Rotterdam, Netherlands; School of Medicine (Prof J A Singh MD), Baylor College of Medicine, Houston, TX, USA; Department of Medicine Service (Prof J A Singh MD), US Department of Veterans Affairs (VA), Houston, TX, USA; Department of Systemic Pathology (R Solanki MD), Touro College of Osteopathic Medicine, Middletown, NY, USA; Department of Pathology (R Solanki MD), American University of the Caribbean School of Medicine, Cupecoy, Saint Martin; Institute of Child and Adolescent Health (Y Song PhD), Peking University, Beijing, China; School of Medicine (S Soraneh MD), Babol University of Medical Sciences, Babol, Iran; Global Observatory on Pollution and Health (Prof K Straif PhD), Boston College, Chestnut Hill, MA, USA; ISGlobal Instituto de Salud Global de Barcelona, Barcelona, Spain (Prof K Straif PhD); Collegium Medicum (Prof L Szarpak PhD), John Paul II Catholic University of Lubin, Lublin, Poland; Department of Clinical Research and Development (Prof L Szarpak PhD), LUXMED Group, Warsaw, Poland; Department of Medical Informatics (S Tabatabaei PhD), Clinial

Research Development Unit (S Tabatabaei PhD), Mashhad University of Medical Sciences, Mashhad, Iran; Department of Primary Care and Public Health (C Tabche MSc), Imperial College London, London, UK; Department of Radiology (M Tanwar MD), University of Alabama at Birmingham, Birmingham, AL, USA; Department of Economics (N Y Tat MS), Rice University, Houston, TX, USA; Department of Research and Innovation (N Y Tat MS), Enventure Medical Innovation, Houston, TX, USA; Department of Business Analytics (T H Tran MD), University of Massachusetts Dartmouth, Dartmouth, MA, USA; Department of Clinical and Experimental Medicine (D Trico MD), University of Pisa, Pisa, Italy; Faculty of Medicine (T T Truyen MD), Nam Can Tho University, Can Tho, Vietnam; Department of Nutrition and Food Studies (S Tyrovolas PhD), George Mason University, Fairfax, VA, USA; Faculty of Health and Life Sciences (A Udoh PhD), University of Exeter, Exeter, UK; Department of Zoology (S Ullah PhD), Division of Science and Technology (S Ullah PhD), University of Education Lahore, Lahore, Pakistan; College of Health and Sport Sciences (A G Vaithinathan MSc), University of Bahrain, Zallaq, Bahrain; Department of Cardiology (M Vinayak MD), Icahn School of Medicine at Mount Sinai, New York, NY, USA; Department of Parasitology (Prof K G Weerakoon PhD), Department of Community Medicine (N D Wickramasinghe MD), Rajarata University of Sri Lanka, Anuradhapura, Sri Lanka; National Data Management Center for Health (NDMC) (A A Wolde MPH), Ethiopian Public Health Institute, Addis Ababa, Ethiopia; Department of Public Health (T E Wonde MPH), Debre Markos University, Debre Markos, Ethiopia; Department of Endocrinology (Prof S Xu PhD), University of Science and Technology of China, Hefei, China; Department of Cancer Epidemiology and Prevention Research (L Yang PhD), Alberta Health Services, Calgary, AB, Canada; Department of Oncology (L Yang PhD), University of Calgary, Calgary, AB, Canada; Faculty of Medicine (Y Yano MD), Juntendo University, Tokyo, Japan; Department of Health Management (A Yiğit PhD), Süleyman Demirel Üniversitesi (Süleyman Demirel University), Isparta, Türkiye; Department of Pediatrics (Prof D Yon MD), Kyung Hee University, Seoul, South Korea; Department of Bioengineering and Therapeutical Sciences (Prof M Zastrozhin PhD), University of California San Francisco, San Francisco, CA, USA; Department of Administration (Prof M Zastrozhin PhD), PGxAI, San Francisco, CA, USA; Department of Public Health (M G M Zeariya PhD), University of Hail, Hail, Saudi Arabia; Department of Zoology and Entomology (M G M Zeariya PhD), Al-Azhar University, Cairo, Egypt; School of Public Health and Emergency Management (B Zhu PhD), Southern University of Science and Technology, Shenzhen, China; Department of Biochemistry and Pharmacogenomics (M Zielińska MPharm), Medical University of Warsaw, Warsaw, Poland; Department of Clinical and Community Pharmacy (Prof S H Zyoud PhD), An-Najah National University, Nablus, Palestine; Clinical Research Centre (Prof S H Zyoud PhD), An-Najah National University Hospital, Nablus, Palestine; Department of Psychological Medicine (J A Kerr PhD), University of Otago, Christchurch, New Zealand; GBD Collaborating Unit (Prof S E Vollset DrPH), Norwegian Institute of Public Health, Bergen, Norway.

## Authors' Contributions

### Managing the overall research enterprise

Peter Azzopardi, Emmanuela Gakidou, Simon I Hay, Paulina Lindstedt, Ali H Mokdad, Christopher J L Murray, Susan Sawyer, Amanda E Smith, and Stein Emil Vollset.

### Writing the first draft of the manuscript

Karly Cini, Xiaochen Dai, Emmanuela Gakidou, Marie Ng, and Jessica Kerr.

### Primary responsibility for applying analytical methods to produce estimates

Dana Bryazka, Rebecca Cogen, Xiaochen Dai, and Justin Lo.

## Primary responsibility for seeking, cataloguing, extracting, or cleaning data; designing or coding figures and tables

Noah Ahmad, Karly Cini, Rebecca Cogen, Xiaochen Dai, Dorothea Dumuid, Jessica Kerr, Justin Lo, and Marie Ng.

## Providing data or critical feedback on data sources

Auwal Abdullahi, Richard Gyan Aboagye, Hana J Abukhadijah, Danish Ahmad, Noah Ahmad, Ayman Ahmed, Fares Alahdab, Abdelazeem M Algammal, Najim Z Alshahrani, Mohammad Al-Wardat, Jalal Arabloo, Alok Atreya, Atif Amin Baig, Abdulaziz T Bako, Kannu Bansal, Till Winfried Bärnighausen, Mohammad-Mahdi Bastan, Sonu Bhaskar, Archie Bleyer, Hamed Borhany, Dejana Braithwaite, Mehtap Çakmak Barsbay, Francieli Cembranel, Pamela Roxana Chacón-Uscamaita, Vijay Kumar Chattu, Bryan Chong, Alyssa Columbus, Rosa A S Couto, Michael H Criqui, Natalia Cruz-Martins, Xiaochen Dai, Samuel Demissie Darcho, Hardik Dineshbhai Desai, Samath Dhamminda Dharmaratne, Michael J Diaz, Thanh Chi Do, Ojas Prakashbhai Doshi, Rajkumar Prakashbhai Doshi, Robert Kokou Dowou, Abdel Rahman E'mar, Rabie Adel El Arab, Ibrahim Farahat El Bayoumy, Chadi Eltaha, Hossein Farrokhpour, Ginenus Fekadu, Muktar A Gadanya, Emmanuela Gakidou, Ehsan Gholami, Mahaveer Golechha, Avirup Guha, Nils Haep, Aram Halimi, Wen-Qiang He, Jeffrey J Hebert, Mehdi Hemmati, Nguyen Quoc Hoan, Chengxi Hu, Hong-Han Huynh, Abel Joseph, Rami S Kantar, Mohammad Jobair Khan, Khaled Khatab, Adnan Kisa, Nhi Huu Hanh Le, Munjae Lee, Seung Won Lee, An Li, Stephen S Lim, Justin Lo, Jay B Lusk, Irmina Maria Michalek, GK Mini, Ameen Mosa Mohammad, Shafiu Mohammed, Ali H Mokdad, Kaveh Momenzadeh, Sara Momtazmanesh, Rohith Motappa, Christopher J L Murray, Zuhair S Natto, Dang H Nguyen, Phat Tuan Nguyen, Van Thanh Nguyen, Syed Toukir Ahmed Noor, Fred Nugen, Ogochukwu Janet Nzoputam, Bogdan Oancea, Andrew T Olagunju, Mayowa O Owolabi, Mahesh Padukudru P A, Romil R Parikh, Sungchul Park, Gavin Pereira, Hoang Nhat Pham, Tom Pham, Anil K Philip, Jalandhar Pradhan, Jagadeesh Puvvula, Catalina Raggi, Bita Rahmani, Mohammad Rahmanian, Shakthi Kumaran Ramasamy, Chhabi Lal Ranabhat, Sowmya J Rao, Sina Rashedi, Ahmed Mustafa Rashid, Monica Rodrigues, Jefferson Antonio Buendia Rodriguez, Cameron John Sabet, Siamak Sabour, Umar Saeed, Vijaya Paul Samuel, Abdallah M Samy, Monika Sawhney, Susan M M Sawyer, Muhammad Aaqib Shamim, Amin Sharifan, Aminu Shittu, Jasvinder A Singh, Lukasz Szarpak, Seyyed Mohammad Tabatabaei, Manoj Tanwar, Domenico Trico, Sana Ullah, Jef Van den Eynde, Kosala Gayan Weerakoon, Tewodros Eshete Wonde, Suowen Xu, Yuichiro Yano, Dong Keon Yon, Chuanhua Yu, Michael Zastrozhin, Abzal Zhumagaliuly, and Magdalena Zielińska.

## Developing methods or computational machinery

Noah Ahmad, Aleksandr Y Aravkin, Dana Bryazka, Rebecca M Cogen, Xiaochen Dai, Emmanuela Gakidou, Simon I Hay, Justin Lo, Ali H Mokdad, Christopher J L Murray, Catalina Raggi, Amanda E Smith, Stein Emil Vollset, and Chun-Wei Yuan.

## Providing critical feedback on methods or results

Michael Abdelmasseh, Arash Abdollahi, Auwal Abdullahi, Richard Gyan Aboagye, Hana J Abukhadijah, Temitayo Esther Adeyeoluwa, Aanuoluwapo Adeyimika Afolabi, Danish Ahmad, Ayman Ahmed, Syed Anees Ahmed, Mohammed Ahmed Akkaif, Ashley E Akrami, Syed Mahfuz Al Hasan, Omar Al Ta'ani, Fares Alahdab, Ziyad Al-Aly, Abdelazeem M Algammal, Waad Ali, Akram Al-Ibraheem, Saleh A Alqahatni, Rami H Al-Rifai, Najim Z Alshahrani, Mohammad Al-Wardat, Hany Aly, Walid A Al-Zyoud, Sohrab Amiri, Jalal Arabloo, Demelash Areda, Mubarek Yesse Ashemo, Alok Atreya, Sina Azadnajafabad, Shahkaar Aziz, Peter S Azzopardi, Giridhara Rathnaiah Babu, Atif Amin Baig, Abdulaziz T Bako, Kannu Bansal, Till

Winfried Bärnighausen, Mohammad-Mahdi Bastan, Maryam Bemanalizadeh, Habtamu B Beyene, Sonu Bhaskar, Cem Bilgin, Archie Bleyer, Hamed Borhany, Edward J Boyko, Dejana Braithwaite, Dana Bryazka, Raffaele Bugiardini, Yasser Bustanji, Zahid A Butt, Mehtap Çakmak Barsbay, Ismael Campos-Nonato, Francieli Cembranel, Ester Cerin, Pamela Roxana Chacón-Uscamaita, Eeshwar K Chandrasekar, Vijay Kumar Chattu, An-Tian Chen, Guangjin Chen, Gerald Chi, So Mi Jemma Cho, Dong-Woo Choi, Bryan Chong, Sheng-Chia Chung, Zinhle Cindi, Karly I Cini, Alyssa Columbus, Rosa A S Couto, Michael H Criqui, Natalia Cruz-Martins, Omar B Da'ar, Omid Dadras, Xiaochen Dai, Zhaoli Dai, Samuel Demissie Darcho, Nihar Ranjan Dash, Hardik Dineshbhai Desai, Samath Dhamminda Dharmaratne, Daniel Diaz, Michael J Diaz, Thanh Chi Do, Mahsa Dolatshahi, Mario D'Oria, Ojas Prakashbhai Doshi, Rajkumar Prakashbhai Doshi, Robert Kokou Dowou, John Dube, Dorothea Dumuid, Arkadiusz Marian Dziedzic, Abdel Rahman E'mar, Rabie Adel El Arab, Ibrahim Farahat El Bayoumy, Muhammed Elhadi, Chadi Eltaha, Hossein Farrokhpour, Patrick Fazeli, Valery L Feigin, Ginenus Fekadu, Florian Fischer, Kate Louise Francis, Muktar A Gadanya, Emmanuela Gakidou, Miglas Welay Gebregergis, Delaram J Ghadimi, Ehsan Gholami, Mahaveer Golechha, Davide Golinelli, Philimon N Gona, Ashna Grover, Avirup Guha, Rahul Gupta, Parham Habibzadeh, Nils Haep, Aram Halimi, Md. Kamrul Hasan, Md Saquib Hasnain, Simon I Hay, Wen-Qiang He, Mehdi Hemmati, Yuta Hiraike, Nguyen Quoc Hoan, Chengxi Hu, Hong-Han Huynh, Md. Rabiul Islam, Sheikh Mohammed Shariful Islam, Louis Jacob, Abel Joseph, Sivesh Kathir Kamarajah, Kehinde Kazeem Kanmodi, Rami S Kantar, Yeganeh Karimi, Sina Kazemian, Jessica A Kerr, Mohammad Jobair Khan, Muhammad Shahzeb Khan, Shaghayegh Khanmohammadi, Khaled Khatab, Moawiah Mohammad Khatatbeh, Moein Khormali, Jagdish Khubchandani, Sylvia Kiconco, Min Seo Kim, Ruth W Kimokoti, Adnan Kisa, Vijay Kumar, Satyajit Kundu, Om P Kurmi, Hanpeng Lai, Nhi Huu Hanh Le, Munjae Lee, Seung Won Lee, Wei-Chen Lee, Wei Li, Stephen S Lim, Jialing Lin, Xiaofeng Liu, Justin Lo, José Francisco López-Gil, Giancarlo Lucchetti, Lisha Luo, Jay B Lusk, Elham Mahmoudi, Elaheh Malakan Rad, Yosef Manla, Ramon Martinez-Piedra, Yasith Mathangasinghe, Fernanda Penido Matozinhos, Steven M McPhail, Hadush Negash Meles, George A Mensah, Sultan Ayoub Meo, Tomislav Mestrovic, Irmina Maria Michalek, GK Mini, Mohammad Mirza-Aghazadeh-Attari, Gabriele Mocciaro, Jama Mohamed, Mouhand F H Mohamed, Nouh Saad Mohamed, Ameen Mosa Mohammad, Shafiu Mohammed, Ali H Mokdad, Kaveh Momenzadeh, Sara Momtazmanesh, Fateme Montazeri, Maziar Moradi-Lakeh, Shane Douglas Morrison, Rohith Motappa, Christopher J L Murray, Pirouz Naghavi, Soroush Najdaghi, Delaram Narimani Davani, Zuhair S Natto, Marie Ng, Dang H Nguyen, Hau Thi Hien Nguyen, Phat Tuan Nguyen, Van Thanh Nguyen, Yeshambel T Nigatu, Nasrin Nikravangolsefid, Syed Toukir Ahmed Noor, Fred Nugen, Ogochukwu Janet Nzoputam, Bogdan Oancea, Andrew T Olagunju, Omotola O Olasupo, Abdulhakeem Abayomi Olorukooba, Samuel M Ostroff, Abderrahim Oulhaj, Mayowa O Owolabi, Mahesh Padukudru P A, Romil R Parikh, Seoyeon Park, Sungchul Park, Ava Pashaei, Gavin Pereira, Hoang Nhat Pham, Anil K Philip, Jalandhar Pradhan, Pranil Man Singh Pradhan, Jagadeesh Puvvula, Seyedeh Niloufar Rafiei Alavi, Muhammad Aziz Rahman, Bita Rahmani, Mohammad Rahmanian, Shakthi Kumaran Ramasamy, Chhabi Lal Ranabhat, Sowmya J Rao, Sina Rashedi, Ahmed Mustafa Rashid, Elrashdy Moustafa Mohamed Redwan, Taeho Gregory Rhee, Monica Rodrigues, Jefferson Antonio Buendia Rodriguez, Cameron John Sabet, Siamak Sabour, Umar Saeed, Mohamed A Saleh, Vijaya Paul Samuel, Abdallah M Samy, Monika Sawhney, Susan M M Sawyer, Nikolaos Scarmeas, Markus P Schlaich, Art Schuermans, Sadaf G Sepanlou, Mahan Shafie, Nilay S Shah, Muhammad Aaqib Shamim, Mohammad Ali Shamshirgaran, Sadaf Sharfaei, Amin Sharifan, Anupam Sharma, Aziz Sheikh, Rekha Raghuveer Shenoy, Kenji Shibuya, Aminu Shittu, Kerem Shuval, Emmanuel Edwar Siddig, Diego Augusto Santos Silva, Jasvinder A Singh, Amanda E Smith, Sameh S M Soliman, Yi Song, Soroush Soraneh, Kurt Straif, Lukasz Szarpak, Seyyed Mohammad

Tabatabaei, Celine Tabche, Manoj Tanwar, Mohamad-Hani Temsah, Aravind Thavamani, Thang Huu Tran, Domenico Trico, Stefanos Tyrovolas, Arit Udoh, Sana Ullah, Seyed Mohammad Vahabi, Sanaz Vahdati, Azin Vakilpour, Jef Van den Eynde, Manish Vinayak, Stein Emil Vollset, Kosala Gayan Weerakoon, Nuwan Darshana Wickramasinghe, Asrat Arja Wolde, Tewodros Eshete Wonde, Lin Yang, Arzu Yiğit, Dong Keon Yon, Chuanhua Yu, Michael Zastrozhin, Mohammed G M Zeariya, Claire Chenwen Zhong, Bin Zhu, Magdalena Zielińska, and Sa'ed H Zyoud.

### Drafting the work or revising it critically for important intellectual content

Michael Abdelmasseh, Arash Abdollahi, Auwal Abdullahi, Hana J Abukhadijah, Aanuoluwapo Adeyimika Afolabi, Danish Ahmad, Ayman Ahmed, Syed Anees Ahmed, Mohammed Ahmed Akkaif, Ashley E Akrami, Omar Al Ta'ani, Fares Alahdab, Wafa A Aldhaleei, Abdelazeem M Algammal, Waad Ali, Akram Al-Ibraheem, Saleh A Alqahatni, Rami H Al-Rifai, Najim Z Alshahrani, Mohammad Al-Wardat, Hany Aly, Walid A Al-Zyoud, Sohrab Amiri, Abhishek Anil, Jalal Arabloo, Ali Ardekani, Alok Atreya, Sina Azadnajafabad, Shahkaar Aziz, Peter S Azzopardi, Giridhara Rathnaiah Babu, Atif Amin Baig, Abdulaziz T Bako, Kannu Bansal, Till Winfried Bärnighausen, Mohammad-Mahdi Bastan, Azizullah Beran, Habtamu B Beyene, Sonu Bhaskar, Archie Bleyer, Hamed Borhany, Edward J Boyko, Dejana Braithwaite, Raffaele Bugiardini, Yasser Bustanji, Mehtap Çakmak Barsbay, Ismael Campos-Nonato, Francieli Cembranel, Ester Cerin, Pamela Roxana Chacón-Uscamaita, Eeshwar K Chandrasekar, Vijay Kumar Chattu, An-Tian Chen, Guangjin Chen, Patrick R Ching, Dong-Woo Choi, Bryan Chong, Karly I Cini, Rebecca M Cogen, Alyssa Columbus, Rosa A S Couto, Michael H Criqui, Natalia Cruz-Martins, Xiaochen Dai, Zhaoli Dai, Samuel Demissie Darcho, Nihar Ranjan Dash, Hardik Dineshbhai Desai, Samath Dhamminda Dharmaratne, Daniel Diaz, Michael J Diaz, Thanh Chi Do, Mahsa Dolatshahi, Mario D'Oria, Rajkumar Prakashbhai Doshi, Robert Kokou Dowou, John Dube, Dorothea Dumuid, Arkadiusz Marian Dziedzic, Abdel Rahman E'mar, Rabie Adel El Arab, Ibrahim Farahat El Bayoumy, Muhammed Elhadi, Chadi Eltaha, Nuno Ferreira, Florian Fischer, Kate Louise Francis, Muktar A Gadanya, Emmanuela Gakidou, Miglas Welay Gebregergis, Delaram J Ghadimi, Ehsan Gholami, Davide Golinelli, Philimon N Gona, Mahdi Gouravani, Ayman Grada, Ashna Grover, Avirup Guha, Rahul Gupta, Parham Habibzadeh, Nils Haep, Aram Halimi, Md. Kamrul Hasan, Md Saquib Hasnain, Simon I Hay, Wen-Qiang He, Jeffrey J Hebert, Mehdi Hemmati, Yuta Hiraike, Nguyen Quoc Hoan, Sorin Hostiuc, Junjie Huang, Hong-Han Huynh, Md. Rabiul Islam, Sheikh Mohammed Shariful Islam, Louis Jacob, Abel Joseph, Sivesh Kathir Kamarajah, Kehinde Kazeem Kanmodi, Rami S Kantar, Yeganeh Karimi, Jessica A Kerr, Muhammad Shahzeb Khan, Praval Khanal, Shaghayegh Khanmohammadi, Khaled Khatab, Moawiah Mohammad Khatatbeh, Jagdish Khubchandani, Min Seo Kim, Adnan Kisa, Mukhtar Kulimbet, Vijay Kumar, Satyajit Kundu, Om P Kurmi, Hanpeng Lai, Nhi Huu Hanh Le, An Li, Wei Li, Justin Lo, José Francisco López-Gil, Giancarlo Lucchetti, Jay B Lusk, Elham Mahmoudi, Elaheh Malakan Rad, Ramon Martinez-Piedra, Yasith Mathangasinghe, Fernanda Penido Matozinhos, Steven M McPhail, Hadush Negash Meles, George A Mensah, Sultan Ayoub Meo, Tomislav Mestrovic, Irmina Maria Michalek, Mohammad Mirza-Aghazadeh-Attari, Gabriele Mocciaro, Mouhand F H Mohamed, Nouh Saad Mohamed, Ameen Mosa Mohammad, Shafiu Mohammed, Ali H Mokdad, Kaveh Momenzadeh, Sara Momtazmanesh, Fateme Montazeri, Maziar Moradi-Lakeh, Shane Douglas Morrison, Rohith Motappa, Christopher J L Murray, Soroush Najdaghi, Delaram Narimani Davani, Gustavo G Nascimento, Zuhair S Natto, Marie Ng, Dang H Nguyen, Hau Thi Hien Nguyen, Phat Tuan Nguyen, Van Thanh Nguyen, Yeshambel T Nigatu, Nasrin Nikravangolsefid, Fred Nugen, Ogochukwu Janet Nzoputam, Bogdan Oancea, Sylvester Reuben Okeke, Andrew T Olagunju, Omotola O Olasupo, Abdulhakeem Abayomi Olorukooba, Samuel M Ostroff, Mayowa O Owolabi, Mahesh Padukudru P A, Romil R Parikh, Gavin Pereira, Hoang Nhat Pham, Jalandhar Pradhan, Pranil Man Singh Pradhan, Nicolaas

P Pronk, Jagadeesh Puvvula, Seyedeh Niloufar Rafiei Alavi, Bita Rahmani, Mohammad Rahmanian, Shakthi Kumaran Ramasamy, Chhabi Lal Ranabhat, Sowmya J Rao, Ahmed Mustafa Rashid, Elrashdy Moustafa Mohamed Redwan, Taeho Gregory Rhee, Monica Rodrigues, Jefferson Antonio Buendia Rodriguez, Cameron John Sabet, Umar Saeed, Dominic Sagoe, Vijaya Paul Samuel, Abdallah M Samy, Aswini Saravanan, Susan M M Sawyer, Nikolaos Scarmeas, Markus P Schlaich, Art Schuermans, Sadaf G Sepanlou, Allen Seylani, Mahan Shafie, Nilay S Shah, Muhammad Aaqib Shamim, Amin Sharifan, Anupam Sharma, Manoj Sharma, Rekha Raghuveer Shenoy, Premalatha K Shetty, Kenji Shibuya, Aminu Shittu, Emmanuel Edwar Siddig, Diego Augusto Santos Silva, Jasvinder A Singh, Amanda E Smith, Ranjan Solanki, Sameh S M Soliman, Yi Song, Lukasz Szarpak, Celine Tabche, Manoj Tanwar, Nathan Y Tat, Mohamad-Hani Temsah, Thang Huu Tran, Domenico Trico, Thien Tan Tri Tai Truyen, Stefanos Tyrovolas, Arit Udoh, Sanaz Vahdati, Asokan Govindaraj Vaithinathan, Azin Vakilpour, Jef Van den Eynde, Manish Vinayak, Stein Emil Vollset, Kosala Gayan Weerakoon, Nuwan Darshana Wickramasinghe, Tewodros Eshete Wonde, Suowen Xu, Lin Yang, Arzu Yiğit, Michael Zastrozhin, Claire Chenwen Zhong, Bin Zhu, Abzal Zhumagaliuly, Magdalena Zielińska, and Sa'ed H Zyoud.

### Managing the estimation or publications process

Xiaochen Dai, Emmanuela Gakidou, Simon I Hay, Paulina A Lindstedt, Ali H Mokdad, Erin C Mullany, Christopher J L Murray, Marie Ng, Erin M O'Connell, Samuel M Ostroff, and Amanda E Smith.
